# FUT10 and FUT11 are protein *O*-fucosyltransferases that modify protein EMI domains

**DOI:** 10.1038/s41589-024-01815-x

**Published:** 2025-01-07

**Authors:** Huilin Hao, Youxi Yuan, Atsuko Ito, Benjamin M. Eberand, Harry Tjondro, Michelle Cielesh, Nicholas Norris, Cesar L. Moreno, Joshua W. C. Maxwell, G. Gregory Neely, Richard J. Payne, Melkam A. Kebede, Ramona J. Bieber Urbauer, Freda H. Passam, Mark Larance, Robert S. Haltiwanger

**Affiliations:** 1 Complex Carbohydrate Research Center, University of Georgia, Athens, GA USA; 2 Charles Perkins Centre, School of Medical Sciences, Faculty of Medicine and Health, The University of Sydney, Sydney, New South Wales Australia; 3 Central Clinical School, The University of Sydney, Sydney, New South Wales Australia; 4 Charles Perkins Centre, School of Life and Environmental Sciences, Faculty of Science, The University of Sydney, Sydney, New South Wales Australia; 5 School of Chemistry, Faculty of Science, The University of Sydney, Sydney, New South Wales Australia; 6 Australian Research Council Centre of Excellence for Innovations in Peptide and Protein Science, The University of Sydney, Sydney, New South Wales Australia; 7 Present Address: Regional Fish Institute, Ltd., Kyoto, Japan

**Keywords:** Glycobiology, Post-translational modifications

## Abstract

*O*-Fucosylation plays crucial roles in various essential biological events. Alongside the well-established *O*-fucosylation of epidermal growth factor-like repeats by protein *O*-fucosyltransferase 1 (POFUT1) and thrombospondin type 1 repeats by POFUT2, we recently identified a type of *O*-fucosylation on the elastin microfibril interface (EMI) domain of Multimerin-1 (MMRN1). Here, using AlphaFold2 screens, co-immunoprecipitation, enzymatic assays combined with mass spectrometric analysis and CRISPR–Cas9 knockouts, we demonstrate that FUT10 and FUT11, originally annotated in UniProt as α1,3-fucosyltransferases, are actually POFUTs responsible for modifying EMI domains; thus, we renamed them as POFUT3 and POFUT4, respectively. Like POFUT1/2, POFUT3/4 function in the endoplasmic reticulum, require folded domain structures for modification and participate in a non-canonical endoplasmic reticulum quality control pathway for EMI domain-containing protein secretion. This finding expands the *O*-fucosylation repertoire and provides an entry point for further exploration in this emerging field of *O*-fucosylation.

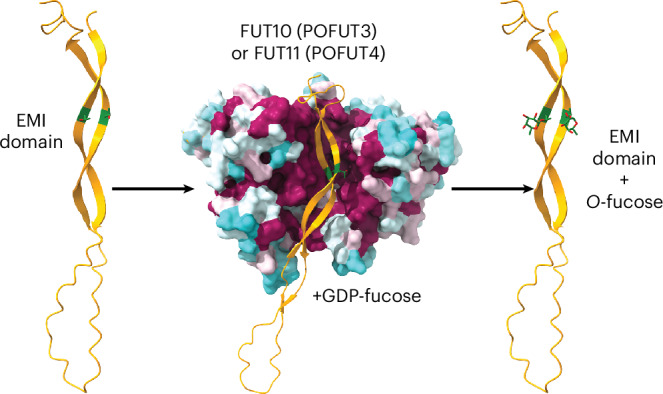

## Abstract (web summary)

FUT10 and FUT11, originally annotated as α1,3-fucosyltransferases, are actually protein *O*-fucosyltransferases participating in a non-canonical ER quality control pathway for EMI domain-containing protein secretion.

## Main

Fucosylated glycan structures are frequently found on cell membranes and extracellular matrix proteins in mammals. They are involved in a broad range of physiological and pathological processes, such as development, selectin-mediated leukocyte adhesion, ABO blood group histocompatibility, cellular signal transduction, host–microbe interactions and tumor metastasis^1–3^. Fucosyltransferases (FUTs) are the enzymes that transfer fucose residues from guanosine diphosphate (GDP)-fucose to acceptor molecules, including glycoproteins, glycolipids and oligosaccharides^1,3^. Thirteen *FUT* genes have been identified in the human genome. Based on the linkage of fucose addition, these FUTs are classified into α1,2-, α1,3/4- and α1,6-FUTs and protein *O*-FUTs (POFUTs). α1,2-FUTs (CAZy GT11 family) include FUT1, FUT2 and Sec1 (non-functional protein encoded by a pseudogene)^4,5^. FUT1 and FUT2 transfer fucose in α1,2-linkage to the terminal galactose of lactosamine to make the H antigens. α1,3/4-FUTs (CAZy GT10 family) include FUT3–7 and FUT9, participate in the synthesizing of the complex series of Lewis antigen epitopes and have distinct acceptor specificity^6,7^. Additionally, based on sequence homology to known α1,3/4-FUTs, FUT10 and FUT11 (CAZy GT10 family) were postulated to be α1,3-FUTs, but a comprehensive investigation is needed to elucidate their function and acceptor specificity^8–11^. The only α1,6-FUT identified in mammals is FUT8 (CAZy GT23 family). It catalyzes *N*-glycan core fucosylation that occurs at the innermost GlcNAc moiety of the chitobiose unit^12^.

In contrast to the Golgi-localized FUT1–9 that add fucose to glycan structures, POFUTs catalyze the direct transfer of fucose to proteins via *O*-linkage to the hydroxyl groups of either serine or threonine in the endoplasmic reticulum (ER)^13–15^. To date, only two POFUTs have been identified in mammals: POFUT1 (CAZy GT65 family) and POFUT2 (CAZy GT68 family). Both enzymes require the recognition of a specific consensus sequence within a certain cysteine-rich domain for modification: POFUT1 mediates *O*-fucosylation of epidermal growth factor (EGF)-like repeats containing the consensus sequence C^2^XXXX[S/T]C^3^ (where C^2^ and C^3^ are the second and third cysteine in the EGF), and POFUT2 adds *O*-fucose to thrombospondin type 1 repeats (TSRs) with the consensus sequence C^1^XX[S/T]C^2^ in group 1 TSRs and C^2^XX[S/T]C^3^ in group 2 TSRs^15,16^. Both POFUT1 and POFUT2 are highly specific for their substrates due to the complementary features displayed between the interface of enzyme binding pockets and the three-dimensional (3D) fold of their respective substrates^17,18^. Disrupted *O*-fucosylation is associated with many human congenital disorders and various types of cancer^19–21^. *O*-fucose glycans modulate protein function through both intermolecular and intramolecular interactions^13^. In co-crystal structures of portions of the NOTCH1 ligand-binding domain with its ligands (NOTCH1–DLL4 (ref. ^22^) and NOTCH1–JAG1 (ref. ^23^)), *O*-fucose residues on NOTCH1 EGF8 and EGF12 were found to directly interact with the ligands, regulating NOTCH1 ligand binding and signaling^23,24^. In the crystal structure of BAI1/RTN4-receptor complex, the *O*-fucosylation of TSR3 on BAI1 directly interacts with the RTN4 receptor and regulates neuronal development^25^. Additionally, *O*-fucose forms intramolecular interactions that stabilize folded EGF repeats and TSRs^26,27^. This stabilizing effect drives the domain into an energy well, preventing the re-entry of the domain into the folding cycle and, thereby, aiding in protein folding and secretion. The requirement of both POFUT1 and POFUT2 to modify only properly folded substrates, along with the stabilizing effects of *O*-fucose, suggests that the ER-localized POFUTs may function in a non-canonical quality control pathway designed to ensure the efficient folding of proteins containing cysteine-rich domains, such as EGF repeats and TSRs^27,28^.

**Fig. 1:**
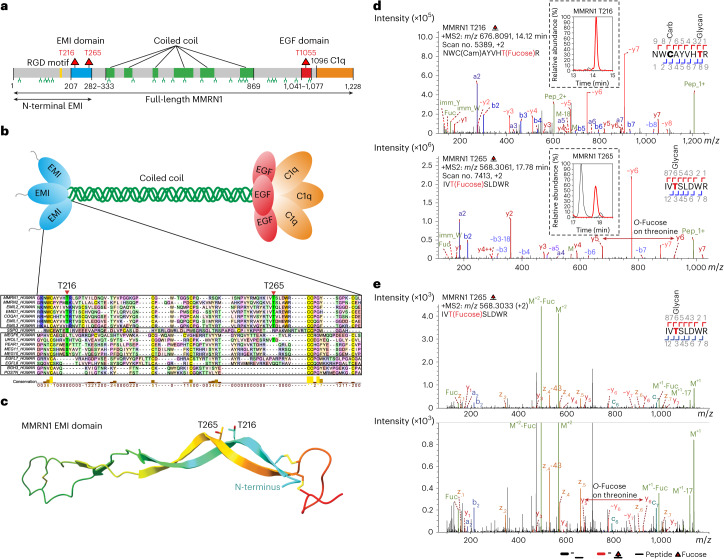
Two *O*-fucose sites were identified on the EMI domain of MMRN1. **a**, Domain organization of human MMRN1 protein. The positions of *O*-linked fucosylation and *N*-glycan sequons are shown as red triangles and green branches, respectively. Protein domains are illustrated: yellow, RGD motif (cell attachment site); blue, EMI domain; green, coiled coil region; red, EGF-like domain; orange, C1q domain. Black arrows indicate the expression constructs used in this study. **b**, Cartoon of the homotrimer MMRN1 structure with key domains shown. Clustal Omega alignment of all human EMI domain protein sequences colored by residue type. Subgroups with high similarity are bound by black boxes. The *O*-fucosylated residues in MMRN1 are labeled. **c**, AlphaFold2 structure of the EMI domain from human MMRN1 protein colored with a rainbow style from the N-terminal side (blue) to the C-terminal side (red). Disulfide bonds are shown, and the *O*-fucosylated residues are labeled. **d**, HCD–MS/MS spectra of fucosylated peptides ^209^NWCAYVHTR^217^ and ^263^IVTSLDWR^270^ that contain the T216 and T265 *O*-fucose sites in MMRN1 EMI domains. EICs of different glycoforms were inserted for each peptide: red lines, *O*-fucose modified; black lines, unmodified. The EIC and spectra for T1055 *O*-fucose modification in MMRN1 EGF domain are in Supplementary Data 1. **e**, Annotated EAD MS/MS spectra for the *O*-fucosylated human MMRN1 peptide derived from the platelet releasate containing T265. Human platelet releasates were digested into peptides and analyzed by targeted LC–MS/MS with EAD fragmentation. Ions in the MS/MS spectrum are annotated as c-ions, z-ions, y-ions, b-ions, a-ions, ~y-ions with a neutral loss (for example, whole glycan loss), M (intact precursor), M-Fuc (intact precursor with neutral loss of fucose) or Fuc (fucose oxonium ion). Mass error for fragment matching is less than 20 ppm using the Byonic software package.

In addition to EGF repeats and TSRs, a recent platelet proteome study identified an uncharacterized *O*-fucose site on the elastin microfibril interface (EMI) domain of Multimerin-1 (MMRN1), a major platelet protein that supports platelet adhesion and thrombus formation^29^ (Fig. 1a,b). Similar to EGF repeats and TSRs, the EMI domain is also a highly conserved cysteine-rich domain with three disulfide bonds (Fig. 1b,c). However, unlike EGF repeats and TSRs, which are frequently found embedded within proteins as tandem repeats, the EMI domain is consistently singular and located at the N-terminus of proteins^30^. EMI domain-containing proteins constitute the EMI domain endowed (EDEN) superfamily^31^. This superfamily can be subdivided into three groups, with the EMILIN/Multimerin family being the most numerous one, including EMILIN-1, EMILIN-2, EMILIN-3, Multimerin-1 and Multimerin-2. All EMILIN/Multimerin family members can form homotrimers (Fig. 1b) and assemble into supramolecular multimers^32^. The EMI domain is proposed to be involved in protein–protein interactions and participates in multimerization^30,33^. The EMI *O*-fucose locates within a partially conserved motif (C^1^XXXX[S/T]X) among all human EMI domains, suggesting a potential role for *O*-fucose in EMI domain functionality (Fig. 1b,c). As the domain context and motif are distinct from classic *O*-fucosylation by POFUT1 and POFUT2, we hypothesized that an as-yet-unidentified POFUT is responsible for this modification. Here we show that neither POFUT1 nor POFUT2 is responsible for the *O*-fucosylation of MMRN1 EMI domain. Instead, we identify FUT10 and FUT11 as the POFUTs that modify protein EMI domains in vitro and in cells. Much like POFUT1 and POFUT2, both FUT10 and FUT11 function in the ER and rely on properly folded EMI structures for efficient modification. Eliminating EMI *O*-fucosylation by either mutating *O*-fucosylated sites or expression in *FUT10/11* double knockout (DKO) cells led to a substantial reduction in protein secretion. This suggests that, akin to POFUT1 and POFUT2, FUT10 and FUT11 participate in a non-canonical ER quality control pathway for EMI domains.

## Results

### Neither POFUT1 nor POFUT2 is responsible for addition of *O*-fucose to MMRN1 EMI domain

To confirm the *O*-fucose site, we performed transient transfections with a full-length human MMRN1 construct in HEK293T cells. Recombinant MMRN1 purified from conditioned culture medium was analyzed by glycoproteomics. Consistent with Houlahan et al.^29^, we observed high stoichiometry *O*-fucosylation at T216 on the N-terminal EMI domain (Fig. 1d) as well as the classical *O*-fucosylation of the C-terminal EGF domain at T1055 (Supplementary Data 1). Additionally, we identified a second *O*-fucose site on the EMI domain at T265, albeit with lower stoichiometry (Fig. 1d,e and Supplementary Data 1). Notably, T216 and T265 are in close spatial proximity within the predicted 3D structure of the MMRN1 EMI domain (Fig. 1c), suggesting that *O*-fucosylation at T265 might occur as a sequential reaction after the T216 modification.

**Fig. 2:**
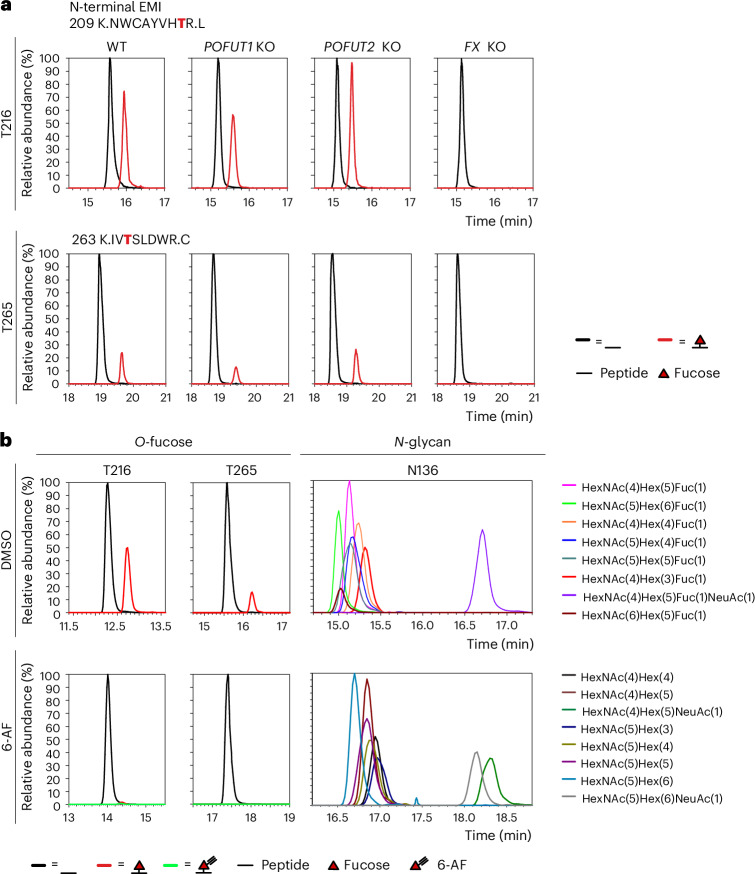
Neither POFUT1 nor POFUT2 is responsible for the *O*-fucosylation of MMRN1 EMI domains. **a**, EICs of different glycoforms of peptides containing the T216 or T265 *O*-fucose site from N-terminal EMI produced in HEK293T WT, *POFUT1* KO, *POFUT2* KO or *FX* KO cells. EICs of positive controls (mNOTCH1 EGF1–5 for POFUT1-mediated EGF *O*-fucosylaton and hTHBS1 TSR1–3 for POFUT2-mediated TSR *O*-fucosylation) are in Supplementary Fig. 2a,b. Spectra for the corresponding ions are in Supplementary Data 1. **b**, EICs of different glycoforms of peptides containing T216 or T265 *O*-fucose sites or N136 *N*-glycan site from N-terminal EMI produced in HEK293T WT cells incubated with DMSO or 6-AF. EICs of positive controls (mNOTCH1 EGF1–5 and hTHBS1 TSR1–3 for 6-AF incorporation) are in Supplementary Fig. 2c,d. Spectra for the corresponding ions are in Supplementary Data 1.

The full-length MMRN1 is poorly expressed in HEK293T cells, likely due to its strong tendency to form large-sized multimers via the interaction of EMI domain and C1q domain. We made a construct subcloned from MMRN1 with the first 290 amino acids covering the N-terminal EMI domain for better expression (Fig. 1a). To investigate if either POFUT1 or POFUT2 is the responsible enzyme for *O*-fucosylation of the EMI domain, we performed transient transfections with the N-terminal EMI construct in wild-type (WT), *POFUT1* KO^27^, *POFUT2* KO^34^ and *FX* KO HEK293T cells. *FX* encodes the enzyme required for GDP-fucose synthesis; knocking out *FX* eliminates all forms of fucosylation within cells and can be rescued by adding fucose to the medium (Supplementary Fig. 1b). The secreted N-terminal EMI was purified from conditioned culture medium and analyzed by glycoproteomics. Extracted ion chromatograms (EICs) were generated for the *O*-fucosylated and unmodified glycoforms of peptides containing either T216 or T265 site in each cell line. A lower *O*-fucosylation stoichiometry was observed in the isolated EMI domain compared to the full-length MMRN1, likely due to a shorter ER retention time for the smaller protein. Peptides containing T216 or T265 were modified with *O*-fucose at similar stoichiometries in WT, *POFUT1* KO and *POFUT2* KO cells but not in *FX* KO cells (Fig. 2a and Supplementary Data 1). Our positive controls showed loss of *O*-fucose on EGF2, EGF3 and EGF5 of NOTCH1 EGF1–5 in *POFUT1* KO cells and loss of *O*-fucose on TSR1, TSR2 and TSR3 of THBS1 TSR1–3 in *POFUT2* KO cells (Supplementary Fig. 2a,b and Supplementary Data 1), confirming that the KO cells cause loss of *O*-fucose on known substrates of POFUT1 and POFUT2, respectively. The loss of *O*-fucose in all recombinant proteins in *FX* KO cells confirms that the modification on EMI domain is a fucose (Fig. 2a, Supplementary Fig. 2a,b and Supplementary Data 1). Equivalent levels of EMI *O*-fucosylation were also observed in full-length MMRN1-transfected WT and *POFUT2* KO HEK293T cells (Supplementary Fig. 3a,b and Supplementary Data 1). These data show that neither POFUT1 nor POFUT2 is responsible for EMI *O*-fucosylation, strongly suggesting that an undiscovered POFUT exists that modifies the EMI domain.

Additional evidence supporting this hypothesis was derived from metabolic labeling of 6-alkynyl fucose (6-AF). Our previous work demonstrated that POFUT1 and POFUT2 can efficiently incorporate 6-AF onto their respective substrates^35^. Metabolic labeling experiments revealed robust incorporation of 6-AF by POFUT1 onto EGF2, EGF3 and EGF5 of NOTCH1 EGF1–5 and by POFUT2 onto TSR2 and TSR3 of THBS1 TSR1–3 (Supplementary Fig. 2c,d and Supplementary Data 1). However, no 6-AF incorporation was detected in EMI (Fig. 2b and Supplementary Data 1). In contrast, the complete absence of *O*-fucose and fucosylation of *N*-glycans on N-terminal EMI confirms that neither POFUT1 nor POFUT2 is responsible for their modification. Loss of fucose on *N*-glycans is consistent with previous results showing that 6-AF depletes cellular GDP-fucose levels^36,37^.

### AlphaFold2-multimer suggests FUT10/11–EMI interactions

**Fig. 3:**
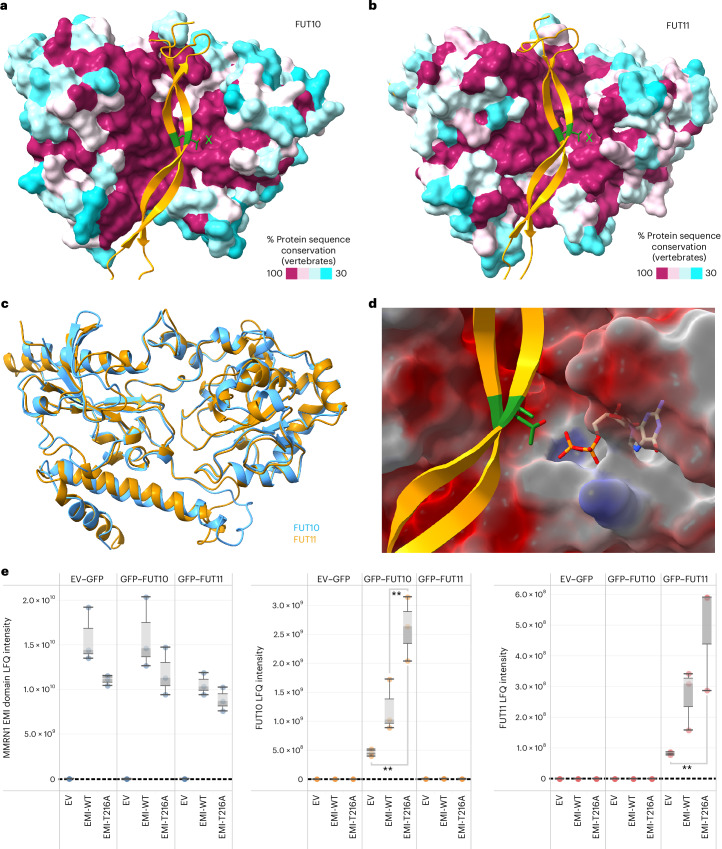
Interaction analysis between FUT10 or FUT11 and the EMI domain. **a**, AlphaFold2-multimer predicted structure of the human FUT10 protein (residues 81–479) and the human MMRN1 EMI domain (residues 184–282). The EMI domain is shown as a gold cartoon depiction with the *O*-fucosylated residues shown in dark green. The FUT10 protein is shown as a surface depiction colored by the sequence conservation across vertebrates. The light green X marks the putative GDP-fucose binding active site. **b**, AlphaFold2-multimer predicted structure of the human FUT11 protein (residues 73–492) and the human MMRN1 EMI domain (residues 184–282). The EMI domain is shown as a gold cartoon depiction with the *O*-fucosylated residues shown in dark green. The FUT11 protein is shown as a surface depiction colored by the sequence conservation across vertebrates. The light green X marks the putative GDP-fucose binding active site. **c**, Comparison of the predicted structures for FUT10 and FUT11. **d**, Docking of GDP into the putative active site of FUT11 using AlphaFold3 (ref. ^41^). The FUT11 protein is shown as a partially transparent surface depiction colored by electrostatic potential (blue, positive; red, negative). The EMI domain is shown as a gold cartoon depiction with the *O*-fucosylated residues shown in dark green. The GDP structure is shown as an atomic model. **e**, Boxplots showing label-free quantification (LFQ) intensity of proteins immunoprecipitated with MMRN1 N-terminal EMI-Myc that was either WT or a T216A mutant. These proteins were isolated from HEK293 cells that were transiently transfected with either empty vector (EV) or the N-terminal EMI-expressing vectors, with or without GFP–FUT10 or GFP–FUT11 vector as shown in the top and bottom axis labels (*n* = 3). For the boxplot, each circle is a biological replicate derived from an individual culture; ** indicates *P* < 0.01 from a one-way ANOVA; the boxes represent the median/quartiles; and whiskers represent 1.5 × the interquartile range. Co-immunoprecipitation of EMI-Myc with full-length FUT11 protein is presented in Supplementary Fig. 5. Source data

To examine potential interactions between all 13 human FUTs and the MMRN1 EMI domain, an AlphaFold2-multimer interaction screen was performed using ColabFold^38^. In this analysis, each FUT was folded with the EMI domain, and the structures, folding confidence (predicted IDDT) and predicted alignment error (PAE) plots were examined (Supplementary Data 2). Only FUT10 and FUT11 generated very high confidence structures (Fig. 3a,b), with both the core of the EMI domain and the majority of FUT10/11 having predicted IDDT scores over 90% (high confidence) (Supplementary Data 2). In addition, only the FUT10/11 models showed all PAE values < 5 Å (low error) for the interface between the EMI domain core and FUT10/11, suggesting a strong interaction. Comparison of the predicted structures for FUT10 and FUT11 showed very high similarity as expected given their approximately 40% protein sequence identity (Fig. 3c).

**Extended Data Fig. 1:**
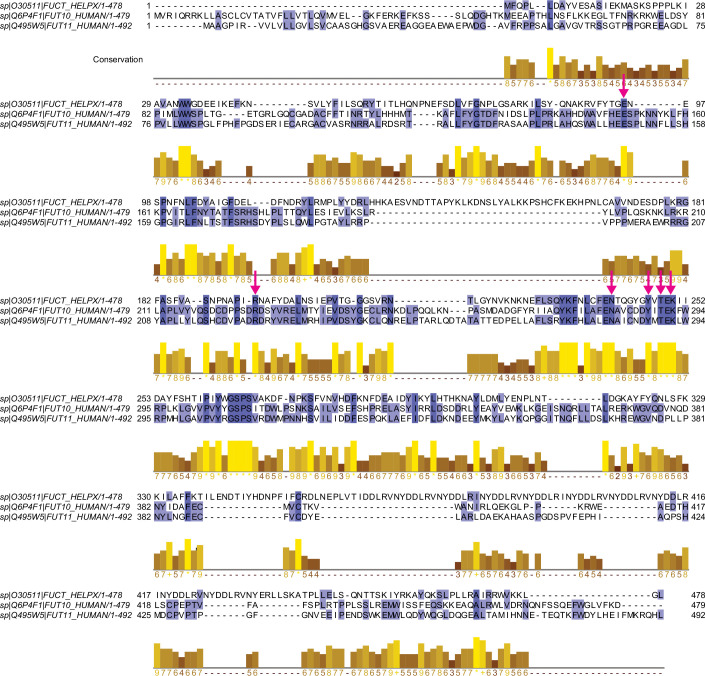
Protein sequence alignment of human FUT10 and FUT11 with *H. pylori* FucT. Clustal Omega alignment of human FUT10 and FUT11 sequences and *H. pylori* FucT sequence. Pink arrows highlight residues known to be essential for enzymatic activity and GDP-fucose binding in FucT, which are also conserved in both FUT10 and FUT11.

**Extended Data Fig. 2:**
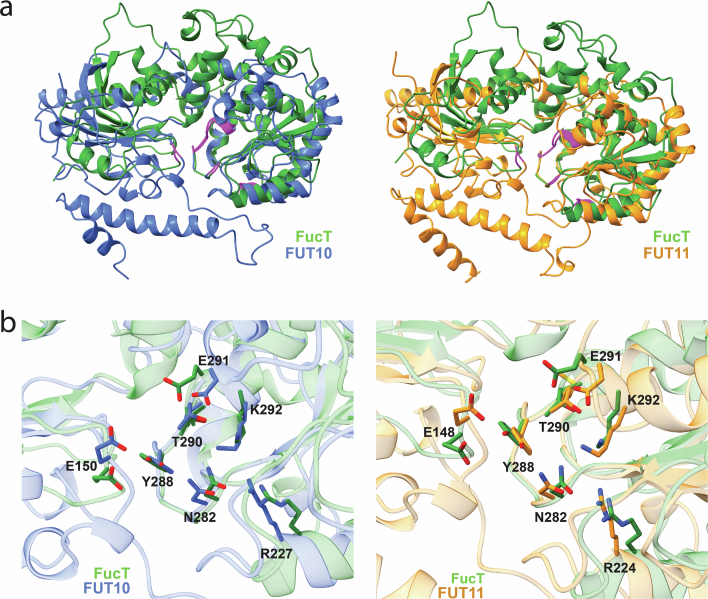
Alignment of human FUT10 and FUT11 AlphaFold2 structures with the crystal structure of *H. pylori* FucT. **a**, AlphaFold2-multimer predicted structures of the human FUT10 (left) and FUT11 (right) proteins, compared to the crystal structure of FucT (PDB: 2NZW) shown in green. Essential residues have been shown in purple. Structures show strong homology within the GDP-fucose binding region but vary more elsewhere in the proteins. **b**, Highlighting the side chains of residues known to be essential for enzymatic activity and GDP-fucose binding in *H. pylori* FucT, alongside the aligned residue within human FUT10/11. Residue number is relative to the human FUT10/11 sequences. Side chains are shown to be of similar charge and orientation between each protein.

**Extended Data Fig. 3:**
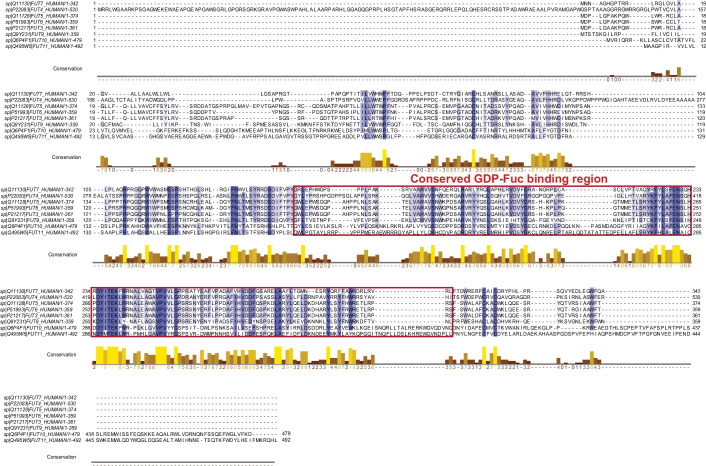
Protein sequence alignment of human GT10 family fucosyltransferases. Clustal Omega alignment of all human GT10 family members (FUT3-7, FUT9-11) coloured by conservation percentage. The GDP-fucose binding region showing high conservation across all GT10 proteins has been bound by a red box.

**Extended Data Fig. 4:**
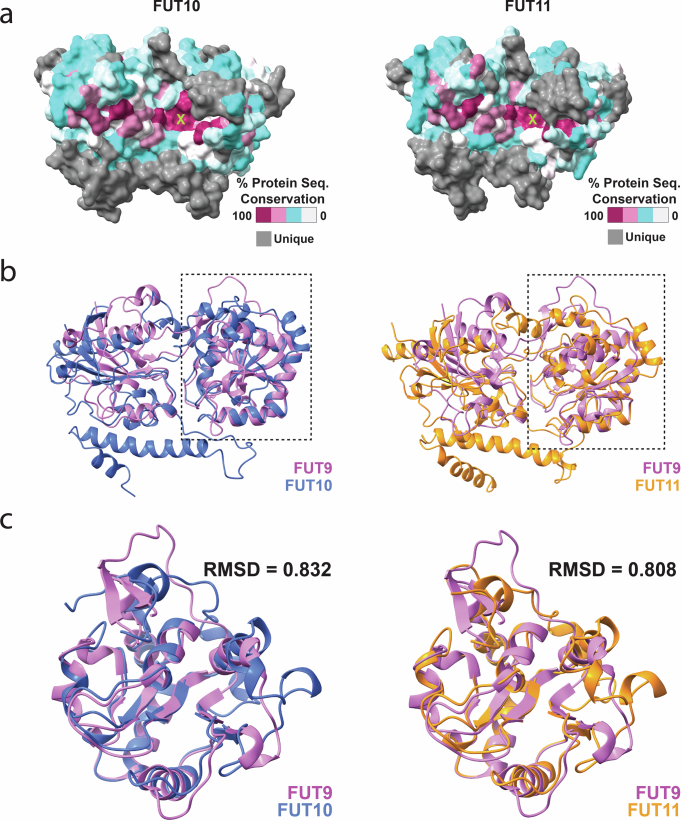
Comparison of FUT10 and FUT11 AlphaFold2 structures with known crystal structure of human FUT9. **a**, AlphaFold2-multimer predicted structures of human FUT10 (residues 81-479) and FUT11 (73-492) shown as surface depictions, colored by sequence conservation across human GT10 proteins. Residues colored in grey are areas unique to FUT10 or FUT11. The light green X marks the putative GDP-fucose binding site. **b**, Comparison of the crystal structure of human FUT9 (PDB: 8D0O) to predicted structures of FUT10 and FUT11 highlight their high structural similarity. The highly conserved GDP-fucose binding domain is bound by a black box. **c**, The GDP-fucose binding domain was isolated to analyze the high structural conservation in this region. The root mean squared deviation (RMSD) value comparing the structural similarity for either FUT10, or FUT11, against FUT9 is provided.

**Extended Data Fig. 5:**
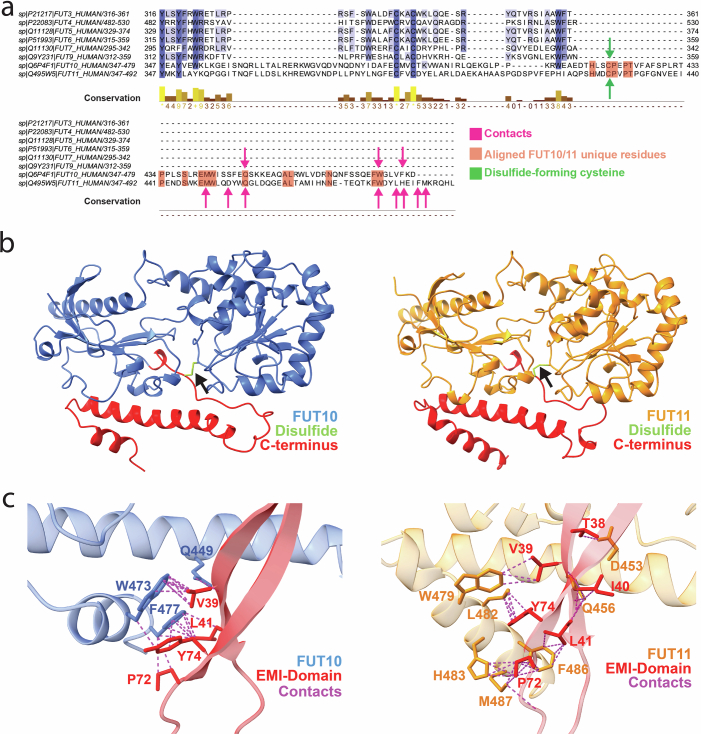
FUT10 and FUT11 have extended C-terminal regions absent in other human GT10 family proteins. **a**, Clustal Omega alignment of the C-terminal region of all human GT10 family proteins (FUT3-7, FUT9-11), colored by conservation percentage. Residues highlighted in red indicate conserved residues found in the extended C-terminal region of FUT10 and FUT11. Predicted contacts (hydrogen bonds, van der Waal interactions) between FUT10/11 and the human MMRN1 EMI domain, as predicted by ChimeraX, are indicated by the pink arrows. Additionally, a disulfide-forming cysteine residue found in the C-terminus unique to FUT10 and FUT11 is indicated by the green arrow. **b**, AlphaFold2-multimer predicted structures of FUT10 and FUT11 with the extended C-terminal region highlighted in red. A unique disulfide bond absent from all other human GT10 family proteins is indicated by the black arrow. **c**, Predicted hydrogen bonds and van der Waal interactions between the C-terminus of FUT10/FUT11 and the human MMRN1 EMI domain substrate, are shown in magenta dashed line. Residue numbers shown in blue and orange refer to FUT10 and FUT11 sequences, respectively; red residue numbers refer to the EMI domain sequence.

Protein sequence alignment between human FUT10 and FUT11 with *Helicobacter pylori* FucT showed strong conservation of residues essential for enzymatic activity and GDP-fucose binding, which is reflected in the aligned 3D protein structures^39^ (Extended Data Figs. 1 and 2). Conservation analysis among all human GT10 family fucosyltransferases, including FUT10 and FUT11, showed strong primary sequence similarity and 100% conservation at the putative GDP-fucose binding pocket (Extended Data Figs. 3 and 4a). 3D alignment of the human FUT9 structure to the human FUT10 and FUT11 predicted structures showed that these proteins adopt a GT-B type fold^40^. The C-terminal Rossmann-like domain responsible for GDP-fucose binding showed very high similarity with root mean squared deviation (r.m.s.d.) < 0.85 when comparing FUT9 and FUT10/11 (Extended Data Fig. 4b,c). We observed that both FUT10 and FUT11 have a unique C-terminal extension among the human GT10 family members, which is predicted to generate additional C-terminal α-helicies that are linked to the remainder of the protein via an additional disulfide bond (Extended Data Fig. 5a,b). This extended C-terminus appears to make multiple contacts with the EMI domain substrate and, thus, may be important for protein substrate selection (Extended Data Fig. 5c).

To determine the putative binding site for GDP-fucose within these enzymes, AlphaFold3 was used to examine FUT11 for GDP binding^41^. GDP bound to a pocket in FUT11 (Fig. 3d) that was highly conserved in FUT11 and FUT10 (Fig. 3a,b, marked with light green X) and was in close proximity to both T216 and T265 of the bound MMRN1 EMI domain. To validate the EMI–FUT11/10 interaction, co-immunoprecipitation using anti-Myc beads was performed from HEK293 cells transiently transfected with either GFP–FUT10 or GFP–FUT11 and N-terminal MMRN1 EMI-Myc (WT or T216A mutant) expressing plasmids. This analysis showed significant interaction between the EMI domain and both GFP–FUT10 and GFP–FUT11 that was strongly enhanced in the T216A EMI mutant (Fig. 3e). It is likely that the enzyme’s affinity for the EMI substrate drops dramatically upon fucose transfer; thus, mutation of the T216 site to alanine results in an overall higher affinity. Given that the GFP–FUT10/11 proteins lack the endogenous transmembrane domain (Supplementary Fig. 4), we also confirmed that full-length FUT11 protein can co-immunoprecipitate with MMRN1 EMI-Myc (Supplementary Fig. 5).

### FUT10 and FUT11 are POFUTs that add *O*-fucose to MMRN1 EMI domain in vitro

**Fig. 4:**
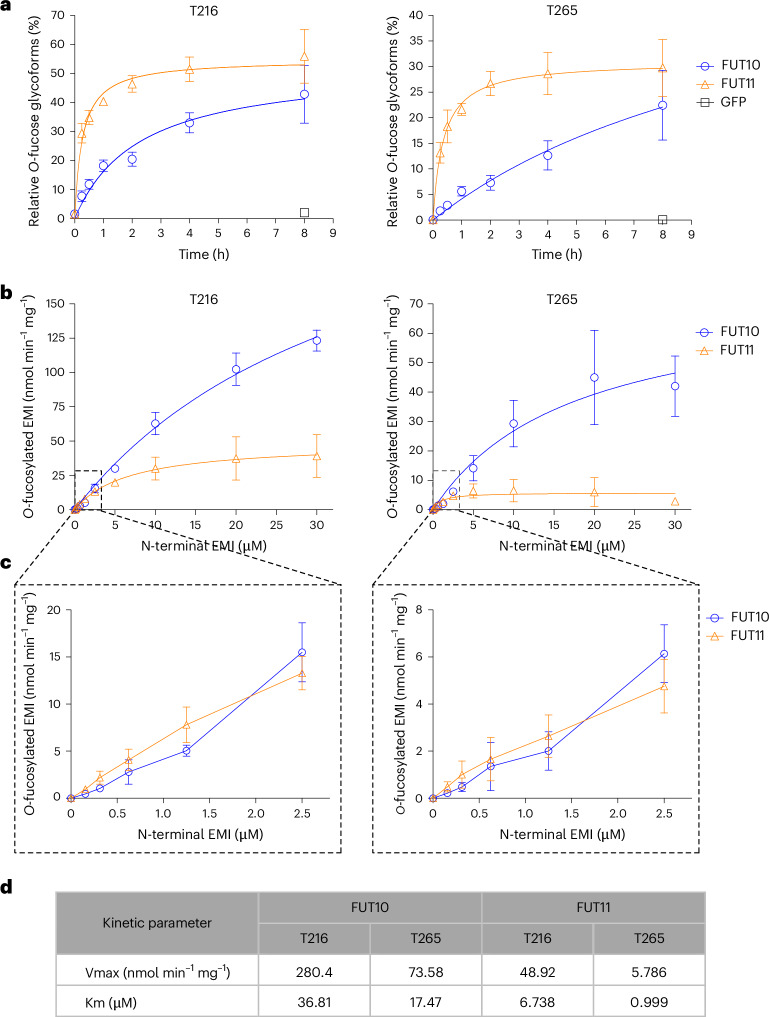
FUT10 and FUT11 are POFUTs that are responsible for the *O*-fucosylation of MMRN1 EMI domains. **a**, 0.1 μM purified GFP–FUT10, GFP–FUT11 or GFP (negative control) was incubated with 0.5 μM non-fucosylated N-terminal EMI and 100 μM GDP-fucose for indicated time periods. Reaction products were reduced, alkylated, digested with trypsin and analyzed by nano LC–MS/MS. Relative abundances of *O*-fucosylation for each reaction were calculated from the EICs of peptides containing T216 or T265 *O*-fucose site and plotted as time-dependent curves. Data are presented as mean ± s.d. from biological triplicates using three batches of purified enzymes (Supplementary Fig. 8). **b**, Substrate concentration-dependent kinetics of GFP–FUT10 and GFP–FUT11. 50 nM purified GFP–FUT10/11 was incubated with varied concentrations of non-fucosylated N-terminal EMI, 100 μM Ultra Pure GDP-fucose and 0.3 mM MnCl_2_ for 15 min. Reaction products were reduced, alkylated, digested with trypsin and analyzed by nano LC–MS/MS. *O*-fucosylation stoichiometry on T216 site and T265 site was quantified from EICs and converted into product concentration. Kinetic analysis was performed with nonlinear Michaelis–Menten fitting in Prism 7. GDP-fucose concentration-dependent kinetics of GFP–FUT11 are in Supplementary Fig. 14. **c**, Data points with substrate concentrations ranging from 0 μM to 2.5 μM in **b** were zoomed in to show the distinct kinetic profiles of GFP–FUT10 and GFP–FUT11 with low substrate concentration. Data are presented as mean ± s.d. from biological triplicates using three batches of purified enzymes (Supplementary Fig. 13). **d**, Kinetic parameters calculated using the data from **b** with nonlinear Michaelis–Menten fitting in Prism 7. Source data

To examine whether FUT10 and FUT11 can modify the MMRN1 EMI domain, we performed enzymatic assays using purified, recombinant GFP–FUT10/11 with non-fucosylated EMI substrates. GFP–FUT10 and GFP–FUT11 were expressed and purified from HEK293F cells using FUT10/11 expression constructs that are designed for glycoenzyme secretion^42^. Recombinant N-terminal EMI substrates were purified from HEK293F cells treated with 6-AF to remove all fucose glycans as described above. The purity of EMI substrates and GFP–FUT10/11 enzymes was confirmed by Coomassie blue staining (Supplementary Fig. 6). The similar quality of purified GFP–FUT10 and GFP–FUT11 was verified using circular dichroism (CD) spectroscopy and nano differential scanning fluorimetry thermostability assays (Supplementary Fig. 7). EMI substrates were incubated with GFP–FUT10 or GFP–FUT11 in the presence of GDP-fucose for different durations at 37 °C, and the products were analyzed by glycoproteomics. Both GFP–FUT10 and GFP–FUT11 can independently add fucose to EMI substrates, exhibiting time-dependent modification on both T216 and T265 sites (Fig. 4a and Supplementary Fig. 8). Curiously, GFP–FUT11 displayed higher efficiency in comparison to GFP–FUT10. GFP–FUT11-mediated reactions rapidly reached saturation within 2 h for both T216 and T265 sites. In contrast, GFP–FUT10 displayed a slower reaction, gradually approaching saturation around 4 h for the T216 site while maintaining a near-linear trend for the T265 site.

Because FUT10 and FUT11 are annotated in UniProt as α1,3-FUTs for *N*-glycans, we further investigated their *N*-glycan-modifying activity. N-terminal EMI contains multiple *N*-glycans (Fig. 1a and Supplementary Fig. 9). The EMI substrates used in enzymatic assays lack *N*-glycan fucosylation due to the 6-AF treatment, making them good substrates for *N*-glycan-modifying FUTs (Fig. 2b). GFP–FUT8 was used as a positive control, and it robustly added fucose to *N*-glycans on EMI after 30 min of incubation (Supplementary Fig. 10). Notably, no changes in *N*-glycan fucosylation on EMI were detected in the GFP–FUT10/11 enzymatic assays mentioned above (Supplementary Fig. 11).

These in vitro activity assays for both GFP–FUT10 and GFP–FUT11 showed that no divalent metal ions were needed for activity. However, the activity of both enzymes was substantially enhanced in the presence of divalent metal ions, including manganese, magnesium and calcium, where manganese showed the highest degree of activation (Supplementary Fig. 12). When supplemented with 0.313 mM MnCl_2_, GFP–FUT10 displayed greater activity enhancement compared to GFP–FUT11, reaching an equivalent or higher level of *O*-fucosylation, particularly at the T265 site. Interestingly, although EDTA does not affect GFP–FUT10 activity, it enhances GFP–FUT11 activity for reasons that remain unclear. Divalent cations have also been shown to enhance the activity of POFUT1 and FUT9 (refs. ^43,44^), although neither of these enzymes has a DxD motif to bind divalent cations nor metal ions observed in their active sites in previous structural analysis^18,40,45^. We propose that the manganese is likely stabilizing the GDP-fucose or the enzyme structure.

In summary, these findings clearly show that both FUT10 and FUT11 function as POFUTs, responsible for modifying EMI domains.

### Kinetic analysis of FUT10 and FUT11

We performed kinetic analyses of GFP–FUT10 and GFP–FUT11 with varied concentrations of acceptor substrate (non-fucosylated N-terminal EMI) supplemented with MnCl_2_ and analyzed with glycoproteomics. We decided to include 0.3 mM MnCl_2_ in the assays because it enhanced the activity of both GFP–FUT10 and GFP–FUT11 compared to controls (Supplementary Fig. 12). Similar kinetic profiles for both T216 and T265 sites were observed in both enzymes, suggesting a shared catalytic mechanism for modifying both sites (Fig. 4b and Supplementary Fig. 13). Notably, the T216 site exhibits higher Km (substrate concentration at half-maximum velocity) and Vmax (maximum reaction rate when the enzyme is fully saturated) values than the T265 site (Fig. 4d). Given the *O*-fucose stoichiometry at these two sites on full-length MMRN1, the EMI domain can accommodate two fucose residues simultaneously (Fig. 1d,e). The differences in kinetic parameters may result from a sequential addition of fucose residues. The presence of the initial fucose on the substrate could alter the catalytic environment, affecting addition of the second fucose. Surprisingly, the overall activity of GFP–FUT10 is significantly higher compared to GFP–FUT11, with Km and Vmax values over five times greater than GFP–FUT11 (T216 Km, 36.81 μM versus 6.738 μM; T216 Vmax, 280.4 nmol min^−1^ mg^−1^ versus 48.92 nmol min^−1^ mg^−1^) (Fig. 4b,d). At lower EMI substrate concentrations (0–1.5 μM), GFP–FUT11 displayed slightly higher activity at the T216 site and similar activity at the T265 site compared to GFP–FUT10 (Fig. 4c). This aligns with the trend that we observed in Fig. 4a, where the concentrations of enzyme and EMI substrate fell within this range, although the difference was notably smaller, likely due to the influence of MnCl_2_ that boosts GFP–FUT10 activity (Supplementary Fig. 12). The lower Km for GFP–FUT11 suggests that the EMI binds more tightly to FUT11. The GDP-fucose concentration-dependent kinetic analyses of GFP–FUT11 showed a curve pattern similar to its EMI concentration-dependent kinetic curve, with a Km of approximately 5 μM for both T216 and T265 (Supplementary Fig. 14). We could not perform GDP-fucose kinetic analysis for GFP–FUT10 owing to the challenge of generating sufficient EMI substrates to reach saturation (Methods). For that reason, the GFP–FUT10 kinetic data in Fig. 4d should be considered preliminary.

We additionally performed a GDP-Glo Glycosyltransferase assay for GFP–FUT10 to validate the kinetic properties obtained via mass spectrometry. As expected, we observed a similar kinetic curve pattern with a similar Km value but a higher Vmax value (Km, 47.22 μM; Vmax, 482.0 nmol min^−1^ mg^−1^) (Supplementary Fig. 15).

### Both FUT10 and FUT11 are independently capable of adding *O*-fucose to EMI domains in cells and are the sole enzymes responsible for this modification

**Fig. 5:**
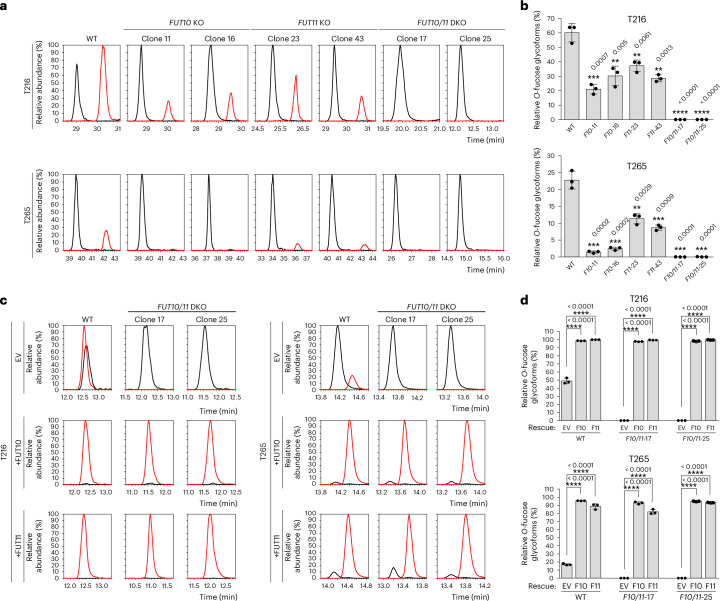
*FUT10* and *FUT11* are responsible for EMI *O*-fucosylation in HEK293T cells. **a**, EICs of different glycoforms of peptides containing the T216 or T265 *O*-fucose site from N-terminal EMI produced in HEK293T WT, *FUT10* KO, *FUT11* KO or *FUT10/11* DKO cells. Red lines, *O*-fucose modified; black lines, unmodified. **b**, Relative abundances of *O*-fucosylated glycoforms in **a** were quantified. *F10*-11, *FUT10* KO cells-clone 11; *F10*-16, *FUT10* KO cells-clone 16; *F11*-23, *FUT11* KO cells-clone 23; *F11*-43, *FUT11* KO cells-clone 43; *F10/11*-17, *FUT10/11* DKO cells-clone 17; *F10/11*-25, *FUT10/11* DKO cells-clone 25. Statistical analysis was performed with unpaired, two-tailed *t*-test in Prism 7. **P < 0.01; ***P < 0.001; ****P < 0.0001 compared to control (WT cells). **c**, EICs of different glycoforms of peptides containing the T216 or T265 *O*-fucose site from N-terminal EMI produced in HEK293T WT or *FUT10/11* DKO cells that co-transfected with plasmids encoding FUT10, FUT11 or EV. Red lines, *O*-fucose modified; black lines, unmodified. **d**, Quantified relative abundances of *O*-fucosylated glycoforms in **c**. F10, FUT10 plasmid; F11, FUT11 plasmid. Statistical analysis was performed with unpaired, two-tailed *t*-test in Prism 7. ****P < 0.0001 compared to control (EV). All data are shown as mean ± s.d. from biological triplicates of three individual transfections (Supplementary Figs. 17 and 18). Source data

Our in vitro data suggest that both FUT10 and FUT11 can add *O-*fucose to the EMI domain. To determine whether both enzymes participate in the *O*-fucosylation of the MMRN1 EMI domain within cells, we generated CRISPR–Cas9-mediated knockouts of *FUT10*, *FUT11* or both genes in HEK293T cells. Successful gene KOs were confirmed by genomic DNA sequencing for all *FUT10*, *FUT11* single KOs and *FUT10/11* DKOs (Supplementary Fig. 16). These cells were transiently transfected with the N-terminal EMI construct. Conditioned culture medium containing the secreted recombinant protein was analyzed by glycoproteomics. Knocking out *FUT10* led to an approximately 60% reduction in *O*-fucose stoichiometry at the T216 site and an approximately 90% reduction at the T265 site, whereas knocking out *FUT11* led to an approximately 50% reduction at the T216 site and an approximately 60% reduction at the T265 site. Knocking out both *FUT10* and *FUT11* completely eliminated *O*-fucosylation at both T216 and T265 sites (Fig. 5a,b and Supplementary Fig. 17). Overexpression of either FUT10 or FUT11 in the DKO cells fully restored *O*-fucosylation at both T216 and T265 sites and even drove the reaction to completion (Fig. 5c,d and Supplementary Fig. 18). These findings indicate that both FUT10 and FUT11 are independently capable of adding *O*-fucose to EMI domains within cells and are the sole enzymes in HEK293T cells responsible for modifying EMI domains.

In contrast to the significant change in *O*-fucosylation, no notable differences were observed in the fucosylation of *N*-glycans on N-terminal EMI between WT and *FUT10/11* DKO cells, even when co-transfected with FUT10 or FUT11, indicating that FUT10 and FUT11 are not modifying *N*-glycans (Supplementary Fig. 19).

**Extended Data Fig. 6:**
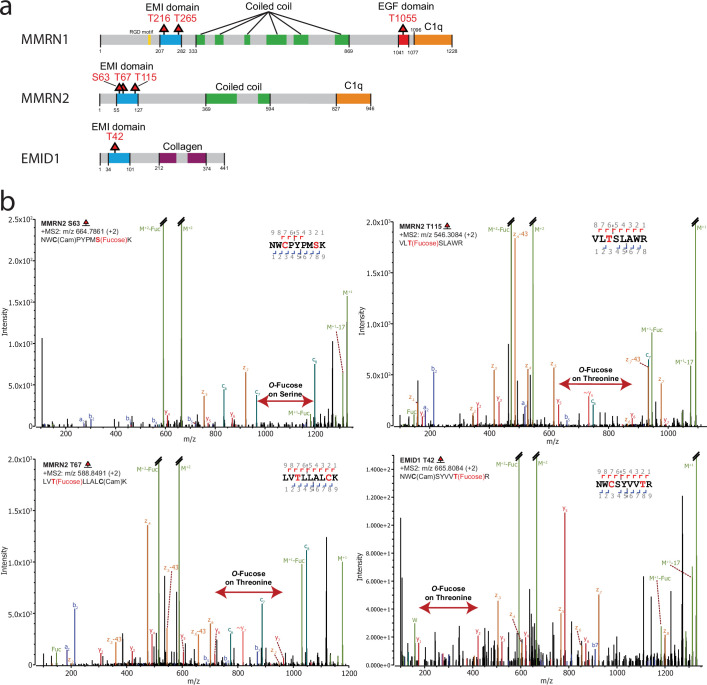
Annotated EAD MS/MS spectra for the *O*-fucosylated MMRN2 and EMID1. **a**, Domain organization of human MMRN1 protein compared to human MMRN2 and EMID1. The positions of *O*-linked fucosylation are shown as red triangles. Protein domains are illustrated: blue – EMI domain; green – coiled-coil region; red – EGF-like domain; orange – C1q domain; purple – collagen domain. **b**, Annotated EAD MS/MS spectra for the *O*-fucosylated human MMRN2 and EMID1 peptides expressed as FLAG-tagged fusion proteins in HEK293 cells. Eluates from FLAG-immunoprecipitation were trypsin digested into peptides and analyzed by targeted LC-MS/MS with electron activated dissociation (EAD) fragmentation. Ions in the MS/MS spectrum are annotated as either: c-ions, z.-ions, y-ions, bions, a-ions, ~y-ions with a neutral loss (for example whole glycan loss), M (intact precursor), M-Fuc (intact precursor with neutral loss of fucose), Fuc (fucose oxonium ion). Mass error for fragment matching is <20 ppm using the Byonic software package.

**Extended Data Fig. 7:**
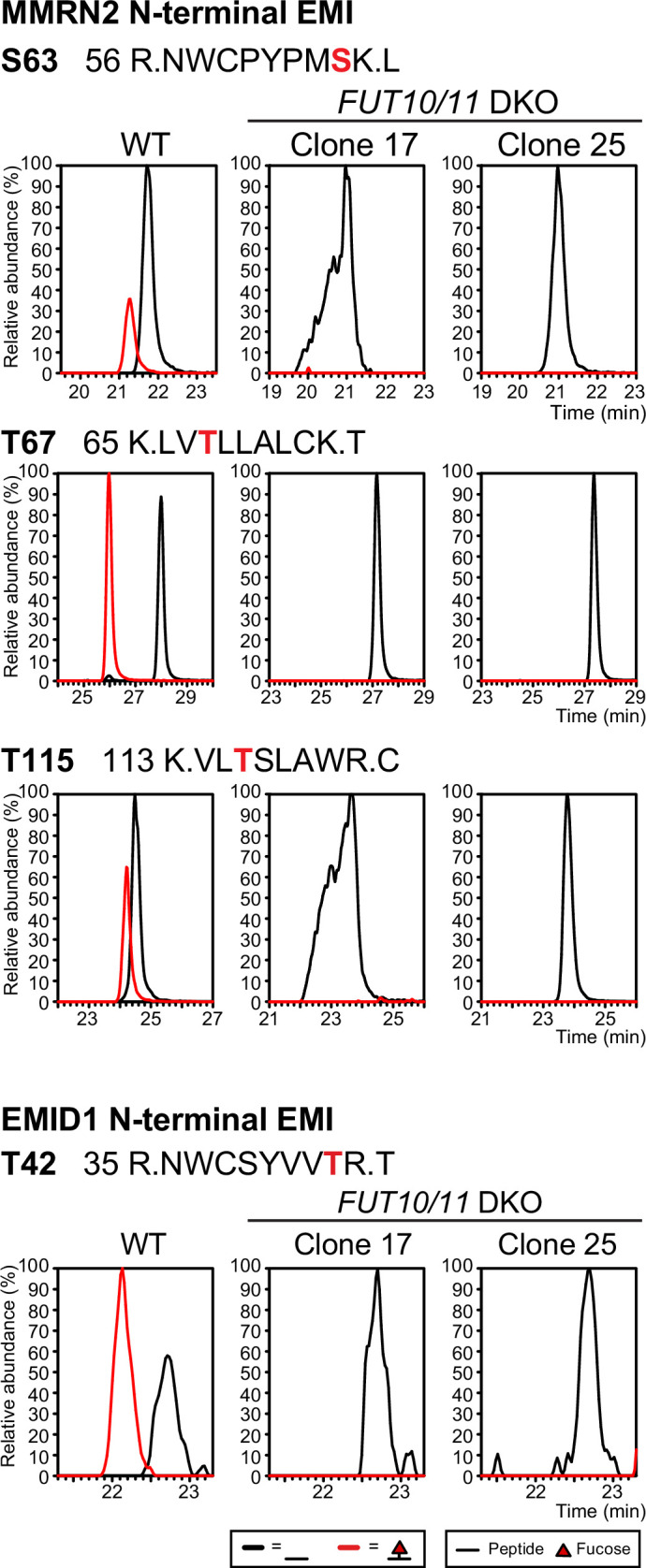
FUT10 and FUT11 are responsible for the *O*-fucosylation of EMI domains in MMRN2 and EMID1. HEK293T WT and *FUT10/11* DKO cells were transfected with constructs encoding the N-terminal EMI of human MMRN2 or EMID1 for 48 h. Proteins were purified from cultured medium and analyzed by nano-LC-MS/MS as described in Methods. EICs of different peptides containing *O*-fucose modifications were extracted. Red lines, *O*-fucose modified; black lines, unmodified. Spectra for the corresponding ions are in Supplementary Data 1.

*O*-fucosylation of the EMI domain was also identified in two other EMI-containing proteins: Multimerin-2 (MMRN2) and EMI domain-containing protein 1 (EMID1) (Extended Data Fig. 6). MMRN2 belongs to the EMILIN/Multimerin family, whereas EMID1 does not. Expression of the EMI domains from MMRN2 and EMID1 in *FUT10/11* DKO cells showed a complete loss of *O*-fucosylation, indicating that FUT10 and FUT11 modify a broader range of EMI-containing proteins beyond the EMILIN/Multimerin family (Extended Data Fig. 7 and Supplementary Data 1).

### FUT10 and FUT11 modify only folded EMI structures and participate in a non-canonical ER quality control pathway for EMI domains

**Fig. 6:**
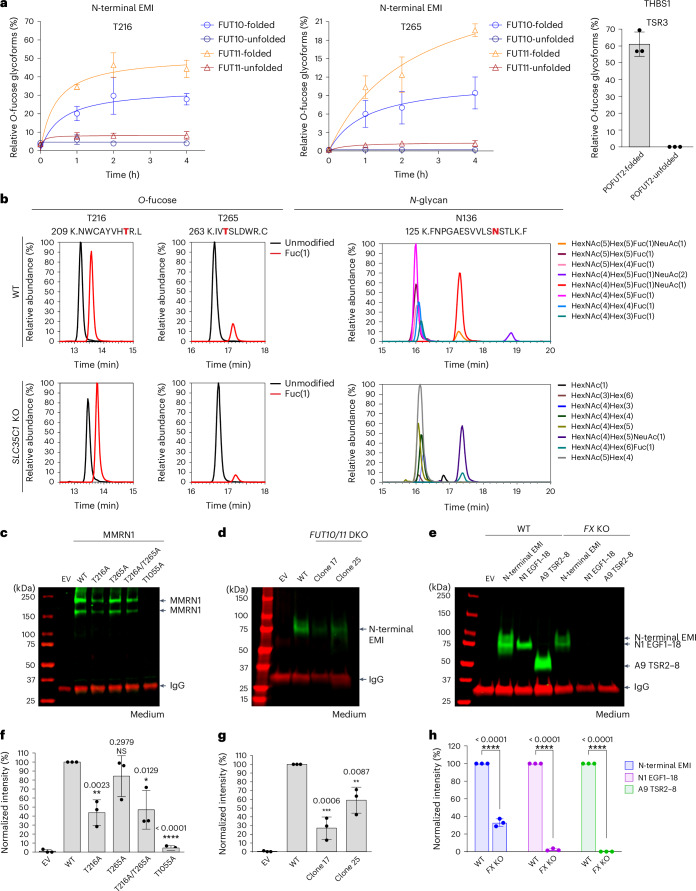
FUT10 and FUT11 require folded EMI structures for modification and function in the ER, participating in a non-canonical ER quality control pathway for EMI domains. **a**, POFUT enzymatic assays with folded and unfolded substrates. Left and middle, GFP–FUT10 and GFP–FUT11 with folded or unfolded N-terminal EMI. Right, GFP–POFUT2 with folded or unfolded hTHBS1 TSR3 as a positive control. Relative abundances of *O*-fucosylated glycoforms were calculated and plotted as time-dependent curves for GFP–FUT10 and GFP–FUT11 or as a bar graph for GFP–POFUT2. Data are shown as mean ± s.d. from biological triplicates using three batches of purified enzymes (Supplementary Fig. 21). **b**, FUT10 and FUT11 likely function in the ER rather than the Golgi. Spectra for the corresponding ions are in Supplementary Data 1. **c**, HEK293T cells were transfected with plasmids encoding Myc-tagged MMRN1 WT, MMRN1 T216A, MMRN1 T265A, MMRN1 T216A/T265A, MMRN1 T1055A or EV and IgG (secretion control). Two-day culture medium was collected and analyzed by western blot probed with anti-Myc and anti-human IgG antibodies. **d**, HEK293T WT or *FUT10/11* DKO cells were transfected with plasmids encoding Myc-tagged N-terminal EMI or EV and IgG (secretion control). Two-day culture medium was analyzed by western blot probed with anti-Myc and anti-human IgG antibodies. **e**, HEK293T WT or *FX* KO cells were transfected with plasmids encoding Myc-tagged N-terminal EMI, mNOTCH1 EGF1–18, hADAMTS9 TSR2–8 or EV and IgG (secretion control). Cells were cultured for 2 d. Culture medium was analyzed by western blot probed with anti-Myc and anti-human IgG antibodies. N1 EGF1–18, mNOTCH1 EGF1–18; A9 TSR2–8, hADAMTS9 TSR2–8. **f**–**h**, Bar graphs show quantified band intensity of protein normalized with IgG band intensity obtained in **c** (**f**), **d** (**g**) and **e** (**h**), respectively. All data are shown as mean ± s.d. from biological triplicates of three individual transfections (Supplementary Figs. 22–24). Data from cell lysates for **c**, **d** and **e** are in Supplementary Figs. 22–24. Statistical analysis was performed with unpaired, two-tailed *t*-test in Prism 7. NS, *P* > 0.05; *P < 0.1; **P < 0.01; ***P < 0.001; ****P < 0.0001 compared to control (WT cells). NS, not significant. Source data

All EGF-modifying *O*-glycosyltransferases (POFUT1, POGLUT1, POGLUT2, POGLUT3 and EOGT) and the TSR-specific *O*-fucosyltransferase (POFUT2) were shown to recognize and modify only properly folded structures in contrast to linear peptides^16,44,46–49^. Thus, we proposed that the EMI-modifying FUT10 and FUT11 may also share this requirement. To examine whether FUT10 and FUT11 modify only folded EMI structures, we performed enzymatic assays with folded versus unfolded EMI domains, with POFUT2 and THBS1 TSR3 as positive controls (Supplementary Fig. 20). N-terminal EMI or THBS1 TSR3 was denatured by reducing and alkylating the disulfide bonds and used as the unfolded substrates (Supplementary Fig. 20a). In line with our previous study, denaturation completely eliminated the ability of TSR3 to serve as a substrate for GFP–POFUT2 (Fig. 6a). Interestingly, similar behavior for both GFP–FUT10 and GFP–FUT11 was observed. Compared to the folded EMI, the unfolded EMI was a poor substrate (Fig. 6a and Supplementary Fig. 21). This suggests that, akin to other *O*-glycosyltransferases that modify EGF repeats or TSRs, FUT10 and FUT11 are capable of distinguishing between folded and unfolded EMI structures and accept only the folded forms for modification.

**Extended Data Fig. 8:**
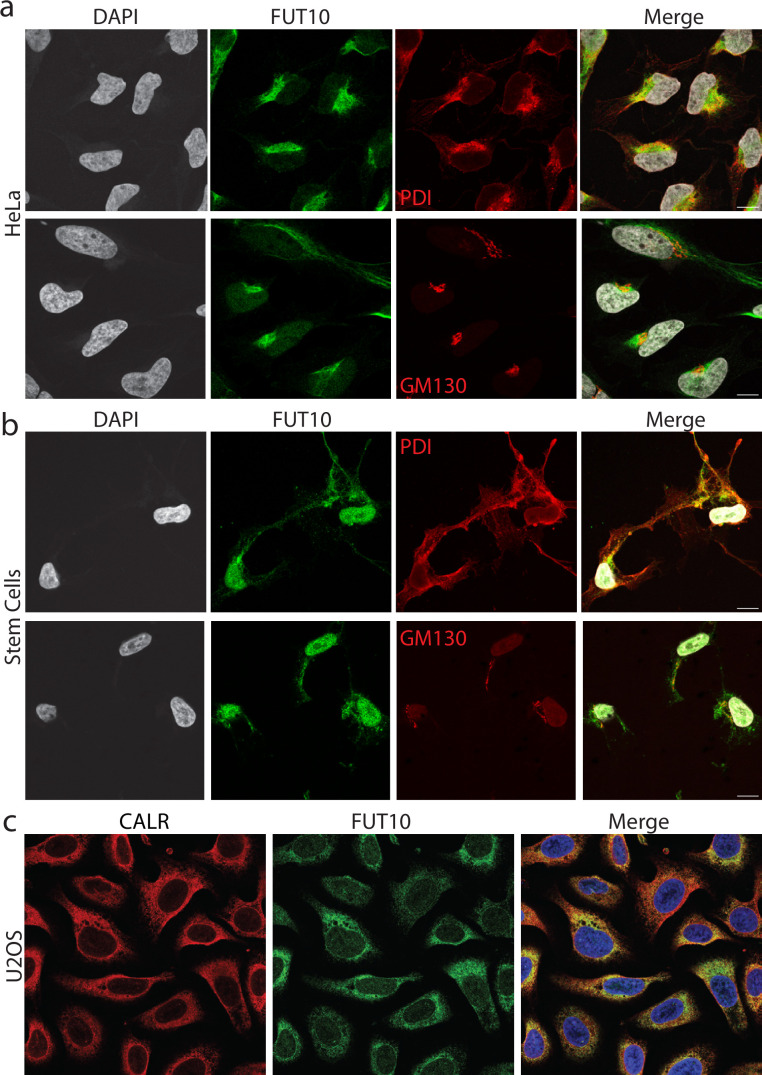
Confocal immunofluorescence microscopy of endogenous FUT10 protein. HeLa cells (**a**) or SCTI003-A human induced pluripotent stem cells (**b**) were stained for either the ER-marker protein disulfide isomerase (PDI) (red), or the cis-Golgi marker (GM130), alongside FUT10 (green) and DNA (DAPI, grey). Stained cells were imaged by confocal microscopy and a single slice is shown (n = 4). Scale bar is 10 μm. **c**, Confocal immunofluorescence microscopy images (n = 2) obtained from the Human Protein Atlas^67^ of human U2OS osteosarcoma cells stained for ether the ER-marker calreticulin (CALR) (red), endogenous FUT10 protein (green) and DNA (DAPI, blue).

**Extended Data Fig. 9:**
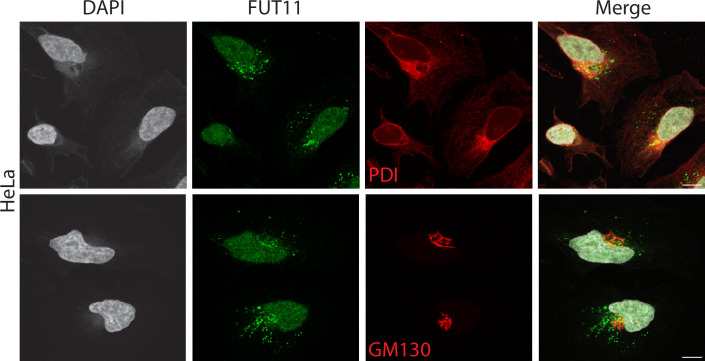
Confocal immunofluorescence microscopy of endogenous FUT11 protein. HeLa cells were stained for either the ER-marker protein disulfide isomerase (PDI) (red), or the cis-Golgi marker (GM130), alongside FUT11 (green) and DNA (DAPI, grey). Stained cells were imaged by confocal microscopy and a single slice is shown (n = 4). Scale bar is 10 μm.

POFUT1 and POFUT2 are localized to the ER and engage in a non-canonical ER quality control pathway for the folding and stabilization of EGF repeats and TSRs, respectively^26–28^. Previous proteomic studies of protein subcellular localization determined that FUT11 is localized in the ER of rat liver tissue and the human osteosarcoma (U2OS) cells^50,51^. In agreement with this, we confirmed using confocal immunofluorescence microscopy that both endogenous FUT10 and FUT11 proteins have substantial overlap with ER marker proteins but not *cis*-Golgi marker proteins in human HeLa, U2OS and stem cells (Extended Data Figs. 8 and 9). It is reasonable to propose that, akin to POFUT1 and POFUT2, FUT10 and FUT11 also function in the ER and participate in a quality control pathway for EMI folding. To examine if FUT10 and FUT11 function in the Golgi, we performed transient transfections with the N-terminal EMI construct in WT and *SLC35C1* KO HEK293T cells^52^ (Fig. 6b and Supplementary Data 1). *SLC35C1* encodes the Golgi GDP-fucose transporter. Loss of SLC35C1 leads to the depletion of the Golgi GDP-fucose pool, thereby inhibiting the activity of Golgi-localized FUTs. As expected, nearly all fucosylation of *N*-glycan on the EMI N136 site was lost in *SLC35C1* KO cells compared to WT cells, confirming the KO status of the cells (Fig. 6b). In contrast to *N*-glycan fucosylation, equivalent *O*-fucosylation levels on EMI T216 and T265 sites were observed in WT and *SLC35C1* KO cells (Fig. 6b). This suggests that FUT10 and FUT11 function in the ER rather than the Golgi.

A known function of *O*-fucose glycans is to aid in protein folding and promote secretion^26–28^. Mutating the T216 *O*-fucose site is known to reduce MMRN1 secretion^29^. To examine the effects of the T265 *O*-fucose site, we mutated either the T265 site alone or both T216 and T265 sites on full-length MMRN1 construct (Supplementary Fig. 22a). Consistent with Houlahan et al.^29^, the T216A mutation showed an approximately 50% reduction in MMRN1 secretion, and the T1055A mutation (EGF domain *O*-fucosylation site; Fig. 1a) showed a complete loss of secretion (Fig. 6c,f). Mutating both T216 and T265 on the EMI domain also led to an approximately 50% reduction in MMRN1 secretion, whereas mutating T265 alone resulted in a non-significant reduction (Fig. 6c,f and Supplementary Fig. 22b). This indicates that only T216 *O*-fucosylation plays a significant role in MMRN1 secretion. MMRN1 levels in cell lysates did not show reduction, indicating that the observed secretion defects are not due to reduced overall expression (Supplementary Fig. 22c–f). We also performed secretion assays in *FUT10/11* DKO cells. To eliminate the impact of *O*-fucosylation on the EGF repeat, we used the N-terminal EMI instead of full-length MMRN1. KO of both *FUT10* and *FUT11* led to a 40–70% reduction in N-terminal EMI secretion compared to WT cells (Fig. 6d,g and Supplementary Fig. 23). A similar reduction was observed in *FX* KO cells, which lack GDP-fucose synthesis^3^ (Fig. 6e,h). In line with our previous studies, eliminating *O*-fucosylation of EGF repeats or TSRs by expression in *FX* KO cells led to a near complete loss in the secretion of NOTCH1 EGF1–18 and ADAMTS9 TSR2–8 (refs. ^27,53^) (Fig. 6e,h and Supplementary Fig. 24). We observed a substantial approximately 70% decrease in the secretion of N-terminal EMI in *FX* KO cells compared to WT cells, highlighting the role of *O*-fucosylation in EMI domain secretion, similar to its contributions to proteins containing EGF repeats or TSRs.

## Discussion

Domain-specific *O*-fucosylation plays critical roles in modulating the biological functions of numerous proteins, with the most prominent substrates to date being the NOTCH receptors and ADAMTS family proteins. In addition to *O*-fucosylation of EGF repeats and TSRs, another domain-specific *O*-fucosylation was recently discovered within the EMI domain^29^. Here we report that FUT10 and FUT11 are the enzymes responsible for this modification. We designate them as POFUT3 and POFUT4, respectively. Both enzymes exhibited strong *O*-fucose transfer activity both in vitro and within cells. Similar to POFUT1 and POFUT2, POFUT3 and POFUT4 rely on properly folded EMI structures for efficient modification. Finally, our findings indicate that POFUT3 and POFUT4 likely function in the ER and participate in a non-canonical ER quality control pathway for EMI domains.

FUT10 and FUT11 have been annotated as α1,3-fucosyltransferases (see Q6P4F1 and Q495W5 in UniProt). They were originally identified in the human genome for sharing sequence homology with *Drosophila FucTB*^11^. The function and acceptor substrate for the fly FucTB has not yet been defined. FucTB is a homolog of FucTA, which has well-established core α1,3-FUT activity, but such activity was not detected for FucTB^54,55^. Both FUT10 and FUT11 belong to the CAZy GT10 family, along with the invertebrate and plant core α1,3-FUTs and all terminal Golgi α1,3/4-FUTs^4^. The Golgi α1,3/4-FUTs (FUT3–7 and FUT9) share five conserved peptide motifs^9^. Motifs VI and V are involved in the recognition and binding of the GDP-fucose donor, and they are well conserved in FUT10 and FUT11. Motifs I–III are involved in the recognition of acceptor substrate, whereas FUT10 and FUT11 show limited sequence homology with other α1,3/4-FUTs in these motifs. Motif II is particularly striking, as instead of [F/I/V]HH[R/W][E/D] that is present in all α1,3/4-FUTs for lactosamine acceptor recognition, FUT10 and FUT11 present a highly conserved FYGTD in the equivalent position, indicating that they have a distinct acceptor substrate^9^. Numerous studies attempted to explore the acceptor specificity of FUT10 and FUT11. Mollicone et al.^9^ reported core α1,3-FUT activity for FUT10 and FUT11 using crude lysates of FUT10/11-transfected COS7 cells as enzyme sources, and they tested with a library of synthetic glycan acceptors through a radioactive-based assay. Kumar et al.^8^ reported that FUT10 is involved in an α1,3-FUT activity for the synthesis of the Lewis X (Le^X^) epitope^8^. They used crude lysates of COS1 cells that overexpress FUT10 as an enzyme source. Although no activity was detected with synthetic glycan substrates, a small increase in Le^X^-containing *N*-glycans was detected in high-performance liquid chromatography (HPLC) analysis when crude lysates of Neuro2a cells were used as glycoprotein substrates. Although it is conceivable that FUT10/11 may add a small amount of fucose to *N*-glycans when tested with large quantities of glycan substrates, it is more likely that the observed activity in crude lysates indirectly results from protein substrates modified by FUT10/11. Supporting this, we did not detect any fucose addition to *N*-glycans on the N-terminal EMI in our in vitro enzymatic assay (Supplementary Fig. 11), whereas there was a robust addition of *O*-fucose (Fig. 4a). Moreover, no significant changes were observed in *N*-glycan fucosylation when *POFUT3* and *POFUT4* were knocked out in cells (Supplementary Fig. 19).

In contrast to the monoexonic α1,3/4-FUTs, the genomic structure of both FUT10 and FUT11 is polyexonic. Phylogenetic analysis indicates that FUT10 and FUT11 are ancient enzymes that originated at about 830 million years ago (MYA), clearly distinct from the α1,3/4-FUTs that originated about 450 MYA^5,9^. POFUT1 and POFUT2 are also ancient enzymes (originated >1,000 MYA) and have a polyexonic structure^56^. Moreover, in agreement with the predicted interaction between FUT11 and MMRN1 in our AlphaFold2 screens, the only FUTs expressed in platelets where MMRN1 is synthesized were FUT11, POFUT1, POFUT2 and FUT8 (ref. ^57^). These clues reinforce our conclusion that FUT10 and FUT11 (POFUT3 and POFUT4, respectively) function as POFUTs rather than α1,3/4-FUTs.

Similar to POFUT1 and POFUT2, POFUT3 and POFUT4 add fucose to the MMRN1 EMI domain within a motif C^1^XXXX[S/T]X that is highly conserved across EMI domains (Fig. 1b). Interestingly, a second fucose was found in close proximity to the primary fucose site in 3D space, and many other EMI domains also possess a threonine at this equivalent site (Fig. 1b,c). *O*-fucosylation at sites equivalent to those in the MMRN1 EMI domain was identified in two other EMI-containing proteins, MMRN2 and EMID1. Examining more EMI domain-containing proteins would provide more information and refine the precision of the consensus sequence. Based on the fucosylation stoichiometry of the two sites, it appears that POFUT3/4 can incorporate at least two fucose modified residues into the same EMI substrate. It would be intriguing to understand the spatial orientation of these fucose groups and the catalytic mechanisms involved in their addition to EMI. AlphaFold3 predictions indicate that POFUT3/4 can accommodate *O*-fucosylated EMI domain substrates (Supplementary Fig. 25). For MMRN2, which can be *O*-fucosylated at three different sites, modification of T67 would require a different orientation of the EMI domain in the POFUT3/4 active site that is not indicated in the AlphaFold predictions. Regarding reaction mechanism, because POFUT3/4 do not require divalent cations for substrate modification, they likely share an S_N_2-like reaction mechanism, similar to other fucosyltransferases^45^.

Finally, we demonstrated that POFUT3 and POFUT4 function and localize in the ER, require folded EMI structures for efficient modification and assist in protein secretion. This evidence indicates that these enzymes play an important role in the quality control of EMI domain-containing proteins, ensuring that they are properly folded for secretion. *O*-fucose glycans are known to form intramolecular interactions that stabilize EGF repeats and TSRs, facilitating protein folding and secretion by preventing the re-entry of these domains into the folding cycle^13^. In the context of EMI domains, as the two fucose sites are situated in the middle of two separate but anti-parallel β-strands, intramolecular interactions may occur between the fucose and spatially nearby amino acid residues on the other β-strand. This interaction could bring the two β-strands closer together, thereby restricting their flexibility and compacting the EMI structure. Supporting this hypothesis is the observation that an MMRN1 T216A mutant displayed a reduced fucosylation level at the T265 site, indicating that the presence of the T216 fucose facilitates the addition of the T265 fucose (Supplementary Fig. 22a). Further work will be needed to explore the detailed mechanisms of how the two fucose residues stabilize EMI structures. Furthermore, *O*-fucose glycans can form direct intermolecular interactions to facilitate protein–protein interactions, regulating processes such as Notch–ligand binding. EMI domains were observed to self-interact, presumably forming a trimer^30^. In addition, the N-terminal EMI domain is proposed to interact with the C-terminal C1q domain for multimerization, indicating a potential role for *O*-fucosylation in these processes^30^.

We are just beginning to understand the biological function of POFUT3/4 and the proteins that they modify. POFUT3 and POFUT4 were reported to have important functions in vertebrate development^8,10^. They are expressed ubiquitously in mouse embryos throughout development, but in adults, they have very distinct expression profiles, and knocking down POFUT4 in zebrafish embryos resulted in malformations of the posterior trunk and tail^10^. Elevated expression of POFUT4 in gastric cancer is linked to poor survival and may serve as a promising biomarker for cancer^58^. POFUT3 was reported to be required for the maintenance of mouse embryonic stem cells and neural stem cells^8^. Identifying the protein substrates of POFUT3/4 that are involved in regulating these biological processes would provide a better understanding of the biological role of POFUT3/4 in embryonic development, tumorigenesis and stem cell maintenance.

## Methods

### Cell culture

HEK293T WT, *POFUT1* KO, *POFUT2* KO, *FUT10* KO, *FUT11* KO, *FUT10/11* DKO, *FX* KO and *SLC35C1* KO cells were cultured at 37 °C with 5% CO_2_ in DMEM (GE Healthcare Life Sciences) supplemented with 10% (v/v) BCS (VWR, 10158-358), 100 U ml^−1^ penicillin and 100 μg ml^−1^ streptomycin (Lonza, 17-602F). HEK293F cells were cultured in FreeStyle 293 medium (Gibco) with 100 U ml^−1^ penicillin and 100 μg ml^−1^ streptomycin. HEK293T cells were purchased from the American Type Culture Collection (ATCC). HEK293F cells were generously provided by Kelley Moremen at the University of Georgia.

### Plasmids and mutagenesis

The mammalian expression plasmids for MMRN1 analysis, including pcDNA3.1-hMMRN1 WT-Myc-His_6_, pcDNA3.1-hMMRN1 T216A-Myc-His_6_ and pcDNA3.1-hMMRN1 T1055A-Myc-His_6_, were described previously^29^. For the generation of pcDNA3.1-hMMRN1 T265A-Myc-His_6_ and pcDNA3.1-hMMRN1 T216A/T265A-Myc-His_6_, the T265A mutation was introduced by standard polymerase chain reaction (PCR)-based mutagenesis using CloneAmp HiFi PCR Premix (Takara Bio) with mutagenic primers AI452 and AI464 using the parental plasmid cDNA3.1-hMMRN1 WT-Myc-His_6_ (for T265A mutant) or pcDNA3.1-hMMRN1 T216A-Myc-His_6_ (for T216A/T265A double mutant) as template. PCR products were treated with DpnI to remove the parental plasmid before being transformed into DH5α-competent cells (Invitrogen).

pcDNA4-hMMRN1 N-terminal EMI-Myc-His_6_ encoding the first 290 amino acids covering the N-terminal EMI domain of hMMRN1 was subcloned from pcDNA3.1-hMMRN1 WT-Myc-His_6_ using CloneAmp HiFi PCR Premix with primers AI364 and AI365. PCR products were digested with HindIII and XhoI and then ligated to pcDNA4/TO/Myc-His_6_ expression vector using T4 Polynucleotide Ligase (New England Biolabs (NEB)).

pcDNA4-hMMRN2 N-terminal EMI-Myc-His_6_, encoding the first 132 amino acids corresponding to the N-terminal portion and EMI domain, was subcloned from full-length pcDNA3.1-hMMRN2-FLAG (made by gene synthesis, GenScript) as template, using cloned Pfu polymerase (VWR). Primers RU004 and RU005, synthesized by Invitrogen, were used. The purified PCR product was digested with restriction enzymes *BamH*I (NEB) and *Xho*I (NEB), ligated with T4 Ligase (NEB) and cloned into pcDNA4/TO/Myc-His_6_.

pcDNA4-hEMID1 N-terminal EMI-Myc-His_6_, encoding the first 102 amino acids comprising the N-terminal portion and EMI, was subcloned from full-length pcDNA3.1-hEMID1-FLAG (made by gene synthesis, GenScript) as template. For PCR, primers RU006 and RU007 and cloned Pfu polymerase were used. The purified PCR product was digested with *Hind*III and *Xho*I, ligated using T4 Ligase and cloned into pcDNA4/TO/Myc-His_6_.

pSecTag2-mNOTCH1 EGF1–5-Myc-His_6_, pSecTag2-mNOTCH1 EGF1–18-Myc-His_6_, pSecTag2-hTHBS1 TSR1–3-Myc-His_6_ and pSecTag2-hADAMTS9 TSR2–8-Myc-His_6_ plasmids were described previously^35,53,59^. pGEn2-GFP, pGEn2-GFP-hPOFUT2, pGEn2-GFP-hFUT10 and pGEn2-GFP-hFUT11 plasmids were described previously and were generously provided by Kelly Moremen at the University of Georgia^42^.

For generating pcDNA4-full-length hFUT10-Myc-His_6_ for the rescue assays: the 5′ signal peptide portion (99 bp) of hFUT10 was amplified by PCR using Platinum SuperFi II DNA Polymerase (Thermo Fisher Scientific) with primers AI456 and AI457 using pUC57-hFUT10 5′ side (made by gene synthesis, GenScript) as template. The hFUT10 coding region was amplified by PCR using CloneAmp HiFi PCR Premix with primers AI458 and AI459 using pGEn2-GFP-hFUT10 as template. The backbone vector portion was amplified by PCR using Platinum SuperFi II DNA Polymerase with primers AI454 and AI455 using pcDNA4/TO/Myc-His_6_ expression vector as template. The infusion reaction was performed using In-Fusion HD Cloning Kits (Takara Bio).

For generating pcDNA4-full-length hFUT11-Myc-His_6_ for the rescue assays: the 5′ signal peptide portion (93 bp) of hFUT11 was amplified by PCR using Platinum SuperFi II DNA Polymerase (Thermo Fisher Scientific) with primers AI460 and AI461 using pUC57-hFUT11 5′ side (made by gene synthesis, GenScript) as template. The hFUT11 coding region was amplified by PCR using CloneAmp HiFi PCR Premix with primers AI462 and AI463 using pGEn2-GFP-hFUT11 as template. The backbone vector portion was amplified by PCR using Platinum SuperFi II DNA Polymerase with primers AI454 and AI455 using pcDNA4/TO/Myc-His_6_ expression vector as template.

All plasmids were confirmed by sequencing (Eurofins). Sequences of all primers are listed in Supplementary Table 1.

### Production of proteins used for mass spectral analysis of *O*-fucosylation

HEK293T WT, *POFUT1* KO, *POFUT2* KO, *FUT10/11* DKO, *FX* KO or *SLC35C1* KO cells (6 × 10^6^ cells per plate) were seeded in 10-cm dishes in 8 ml of DMEM with 10% BCS and 1% penicillin–streptomycin. Cells were cultured overnight to reach a confluency of 70%. Each plate of cells was transiently transfected with 10 μg of pcDNA3.1-hMMRN1 WT-Myc-His_6_, 5 μg of pcDNA4-hMMRN1 N-terminal EMI-Myc-His_6_, 10 μg of pSec-mNOTCH1 EGF1–5-Myc-His_6_, 10 μg of pSec-hTHBS1 TSR1–3-Myc-His_6_, 5 μg of pcDNA4-hMMRN2 N-terminal EMI-Myc-His_6_ or 5 μg of pcDNA4-hEMID1 N-terminal EMI-Myc-His_6_ in 8 ml of Opti-MEM (Thermo Fisher Scientific, 31985088) using 60 μl of polyethylenimine (PEI) (1 μg μl^−1^ stock). For metabolic labeling of cells with 6-AF (generously provided by Peng Wu at the Scripps Research Institute), cells were transfected in 8 ml of Opti-MEM containing 100 μM 6-AF or equal volume of DMSO. Two days later, media from 3–8 plates of cells were combined. Recombinant proteins were purified using 600 μl of Ni-NTA agarose (Qiagen) and eluted with 600 μl of 250 mM imidazole in Tris-buffered saline (TBS), pH 7.5. Eluted purified proteins were stored at −20 °C until use.

For testing the *FUT10/11* KO cells, HEK293T WT, *FUT10* KO, *FUT11* KO or *FUT10/11* DKO cells (1 × 10^6^ cells per well) were seeded in six-well plates and cultured overnight for attachment. Cells were transiently transfected with 1 μg of pcDNA4-hMMRN1 N-terminal EMI-Myc-His_6_ in 1.2 ml of Opti-MEM using 6 μl of PEI (1 μg μl^−1^ stock). Two days later, media were collected and stored at −20 °C until use. For FUT10/11 rescue assay coupled with mass spectrometry analysis, HEK293T WT and *FUT10/11* DKO cells (1 × 10^6^ cells per well) were seeded in six-well plates followed by being transiently transfected with 1 μg of pcDNA4-hMMRN1 N-terminal EMI-Myc-His_6_ with 0.2 μg of pcDNA4-full-length hFUT10-Myc-His_6_, pcDNA4-full-length hFUT11-Myc-His_6_ or empty vector in 1.2 ml of Opti-MEM using 7.2 μl of PEI (1 μg μl^−1^ stock). Cells were cultured for 2 d. Culture media were collected and stored at −20 °C until use.

### Glycoproteomic site mapping of *O*-fucosylated peptides

Purified proteins (300–600 μl of elution from Ni-NTA purification) or 300 μl of conditioned culture medium were precipitated with 3× volumes of cold acetone overnight at −20 °C. After centrifuging at 18,213*g* for 15 min at 4 °C, the pellets were denatured and reduced using 50 μl of reduction buffer containing 8 M urea, 0.4 M ammonium bicarbonate and 10 mM TCEP (Thermo Fisher Scientific, 77720), incubated at 60 °C for 10 min. Alkylation was performed by adding 25 μl of 100 mM iodoacetamide in 50 mM Tris, pH 8, and incubating in the dark for 40 min at room temperature. Samples were then diluted with 225 μl of water and digested with 1 μg of trypsin (Thermo Fisher Scientific, 90057) in a 37 °C water bath overnight. For hTHBS1 TSR1–3 samples, 1 μg of chymotrypsin (Thermo Fisher Scientific, 90056) was added after trypsin digestion, and samples were incubated for 3 h in a 37 °C water bath. Samples were then acidified with formic acid (FA) to 0.5%, sonicated for 20 min and desalted with a C18 ZipTip (Millipore, ZTC18S960). Samples (1–10 μl, ~5 ng of peptides per run in 10% acetonitrile (ACN) and 0.1% aqueous FA) were analyzed by nano liquid chromatography with tandem mass spectrometry (LC–MS/MS) using an EASY-nLC 1200 System (Thermo Fisher Scientific) coupled to a Q Exactive Plus mass spectrometer (Thermo Fisher Scientific) with Tune 2.12 (Build 3134). Peptides were loaded via autosampler and pre-concentrated onto an Acclaim PepMap-100 75 μm × 2 cm nanoViper C18 pre-column with loading buffer, 5% ACN and 0.1% aqueous FA (Solvent A). Peptides were gradient eluted onto a C18 EasySpray analytical column (PepMap RSLC C18, 50 μm × 15 cm, Thermo Fisher Scientific) at a constant flow rate of 300 nl min^−1^ using a 30-min gradient and a 61-min instrument method (for purified protein samples) or a 90-min gradient and a 120-min instrument method (for whole-medium digested samples). The gradient profile was as follows: (min: % Solvent B (80% ACN and 0.1% aqueous FA)): 30-min gradient 0:0, 25:50, 28:98, 31:98, 34:2, 37:2, 40:98, 43:98, 46:2, 49:2, 52:98, 55:98, 58:2, 61:2; 90-min gradient 0:0, 90:50, 93:98, 96:98, 99:2, 102:2, 105:98, 108:98, 111:2, 114:2, 117:98, 120:98. The instrument method used an MS1 resolution of 70,000, an AGC target of 1 × 10^6^ and a mass range from 400 *m*/*z* to 2,000 *m*/*z*. Dynamic exclusion was enabled for 6 s. Only charge states 2–6 were permitted for fragmentation. MS2 scans were acquired in the Orbitrap at a resolution of 17,500, an isolation window of 1.2 *m*/*z* and an AGC target of 1 × 10^5^. The higher-energy collisional dissociation (HCD) fragmentation was set with fixed collision energy of 27%. Data analysis was performed with Byonic software (Protein Metrics, version 4.1.10). Search parameters included fully specific cleavage specificity at the C-terminal site of R and K for trypsin with two missed cleavages allowed and the C-terminal site of R, K, F, W, Y and L for trypsin/chymotrypsin double digestion with five missed cleavages allowed. Mass tolerance was set at 10 ppm for precursors and 0.1 Da for fragments. Cysteine carbamidomethylation was set as fixed modification. Methionine oxidation (common 1), asparagine oxidation (common 1), asparagine deamidation (common 1), N-terminal acetylation (rare 1) and tryptophan hexosylation (common 2) were set as variable modifications with a total common max of 3 and a rare max of 2. Glycans were set as variable modifications (common 1) using a customed *O*-glycan search space including Fuc(1), HexNAc(1)Fuc(1), HexNAc(1)Hex(1)Fuc(1), HexNAc(1)Hex(1)Fuc(1)NeuAc(1), Hex(1)Fuc(1), Hex(1), Hex(1)Pent(1), Hex(1)Pent(2) and HexNAc(1). For 6-AF metabolic labeled samples, 6-AF incorporated glycopeptides were searched with an additional 9.9800 *m*/*z* to account for the chemical modification. Peptides with *N*-glycans were searched with the internal *N*-glycan search list of Byonic (52 common biantennary). To make the EICs for a given peptide, the ions of each glycoform were extracted from the MS1 spectrum using their respective *m*/*z* (observed *m*/*z* from the peptide with the highest Byonic score, mass tolerance: ±0.005 for purified protein samples, ±0.003 for whole-medium digested samples) and then overlaid to compare the relative ion intensity. The EICs were smoothed using a Gauss algorithm. All EICs were manually generated and analyzed using Xcalibur (Thermo Fisher Scientific, version 4.0.27.19).

### AlphaFold2-multimer interaction analysis between FUTs and EMI domains

ColabFold (version 1.5.5) was used to analyze the folding of the MMRN1 EMI domain (MMRN1_HUMAN: 184–282) with each of the following fucosyltransferase protein sequence residue ranges^38^: FUT1_HUMAN: 78–365; FUT2_HUMAN: 61–343; FUT3_HUMAN: 60–361; FUT4_HUMAN: 187–530; FUT5_HUMAN: 75–374; FUT6_HUMAN: 60–359; FUT7_HUMAN: 46–342; FUT8_HUMAN: 105–575; FUT9_HUMAN: 62–359; FUT10_HUMAN: 81–479; FUT11_HUMAN: 73–492; OFUT1_HUMAN: 28–388; and OFUT2_HUMAN: 41–429. Templating from the Protein Data Bank (PDB) was allowed, and relaxation was not used. Structural alignment between predicted structures for human FUT10 and FUT11 and crystal structures of other fucosyltransferases were performed in ChimeraX (version 1.7.1) using the Matchmaker algorithm^60^.

### Co-immunoprecipitation of FUT11 with the MMRN1 EMI domain

HEK293 cells were transiently transfected using Lipofectamine 3000 (Thermo Fisher Scientific) with the plasmids pGEn2-GFP (empty vector GFP), pGEn2-GFP-hFUT10, pGEn2-GFP-hFUT11 or full-length FUT11-FLAG and pcDNA4 (empty vector) or pcDNA4-hMMRN1 N-terminal EMI-Myc-His_6_ (either WT or a T216A mutant). After expression for 24 h in DMEM with 10% FCS, cells were washed with PBS and lysed on ice in lysis buffer (1% Igepal CA-630 (Sigma-Aldrich), 50 mM Tris-HCl pH 7.4, 150 mM NaCl, 5% glycerol, 1 mM MgCl_2_, cOmplete protease inhibitors EDTA-free (Roche)). Lysates were sonicated using a QSonica Q800R2 at 4 °C for 10 min total sonication time, with 30 s ON, 30 s OFF, at 20% amplitude. Lysates were clarified by centrifugation at 18,000*g* for 10 min at 4 °C. Equal amounts of protein from the clarified lysates were incubated with 25 μl of anti-c-Myc tag (9E10) magnetic bead slurry (Thermo Fisher Scientific) at 4 °C for 2 h with rotation. The beads were pelleted on a magnetic rack for 2 min at 4 °C, and the lysate was removed. Bead washing was performed three times at 4 °C using 1 ml per wash of wash buffer (0.05% Igepal CA-63, 150 mM NaCl, 50 mM Tris pH 7.4, 5% glycerol). Excess wash buffer was removed by one wash with 0.5 ml of PBS, and then all liquid was removed from the beads. Co-immunoprecipitated proteins were eluted from the beads in 50 μl of elution buffer (4% sodium deoxycholate (SDC), 0.1 M Tris-HCl pH 8.0) and heated to 95 °C for 5 min. The bead-free eluate was moved to a new tube where TCEP and chloroacetamide were added to 10 mM and 40 mM final concentrations, respectively, with heating to 95 °C for 10 min to reduce and alkylate disulfide bonds. Eluates were cooled to room temperature and diluted with water to achieve a final concentration of 1% SDC. Proteins (10 μg) were digested by adding 200 ng of lysC (Wako) and 200 ng of trypsin (Sigma-Aldrich) to each sample and incubated for 16 h at 37 °C with mixing at 1,000 r.p.m. in a Thermomixer-C (Eppendorf). Peptide cleanup was performed using SDB-RPS StageTips^61^. After digestion, an equal volume of 99% ethyl acetate/1% trifluoroacetic acid (TFA) was added to the digested peptides. Each StageTip was wetted with 100 μl of 100% ACN and centrifuged at 1,000*g* for 1 min. After wetting, each StageTip was equilibrated with 30% methanol/1% TFA and 100 μl of 0.2% TFA in water with centrifugation for each at 1,000*g* for 3 min. Each StageTip was then loaded with the equivalent of approximately 10 μg of peptide from the lower aqueous phase. The peptides were washed twice with 100 μl of 99% ethyl acetate/1% TFA, which was followed by one wash with 100 μl of 0.2% TFA in water. To elute the bound peptides, 100 μl of 5% ammonium hydroxide/80% ACN was added to each tip and centrifuged as above for 5 min. Elutes peptides were dried using a GeneVac EZ-2 using the ammonia setting at 40 °C for 1 h. Dried peptides were resuspended in 5% FA in water, and 500 ng was injected for LC–MS/MS analysis^62^. Peptides were injected onto a 50 cm × 75 μm C18 (Dr. Maisch, 1.9 μm) fused silica analytical column with a 10-μm pulled tip, coupled online to a nanospray electrospray ionization (ESI) source. Peptides were resolved over a gradient from 5% to 35% ACN over 70 min with a flow rate of 300 nl min^−1^. Peptides were ionized by ESI at 2.4 kV. MS/MS analysis was performed using an Exploris 480, a Fusion Lumos or an Eclipse mass spectrometer (Thermo Fisher Scientific) with HCD. MS/MS spectra were attained in a data-dependent acquisition of the top 20 most abundant ions in each MS1 full scan. Proteins were quantified using MaxQuant^63^. A false discovery rate (FDR) of 1% using a target-decoy-based strategy was used for protein and peptide identification. The database used for identification contained the UniProt human database alongside the MaxQuant contaminants database. Mass tolerance was set to 4.5 ppm for precursor ions and 20 ppm for fragments. Trypsin was set as the digestion enzyme with a maximum of two missed cleavages. Oxidation of Met, deamidation of Asn/Gln, pyro-Glu/Gln and protein N-terminal acetylation were set as variable modifications. Carbamidomethylation of Cys was set as a fixed modification. A one-way ANOVA was calculated for each co-immunoprecipitation using R version 4.2.1.

### Electron-activated dissociation fragmentation analysis of *O*-fucosylated EMI domains

For MMRN1 T265 analysis, human platelet thrombin-stimulated releasate was generated^29^, with human ethics approval from the University of Sydney (approval number 2014/244), and our study abides by Declaration of Helsinki principles. Platelets were isolated from whole blood within 4 h of collection. Whole blood was fractioned by centrifugation (200*g* for 20 min, brake = 0) to separate platelet-rich plasma. All centrifugation steps were performed at room temperature. Platelet-rich plasma was rested for 30 min in a water bath at 37 °C before the addition of 20% (v/v) pre-warmed (37 °C) citrate-dextrose solution (Sigma-Aldrich, C3821). Platelets were separated from plasma by centrifugation (800*g* for 10 min, brake = 4). The platelets were resuspended in pre-warmed (37 °C) modified HEPES/Tyrodeʼs (HTGlc) buffer (129 mM NaCl, 0.34 mM Na_2_HPO_4_, 2.9 mM KCl, 12 mM NaHCO_3_, 20 mM HEPES, 5 mM glucose, 1 mM MgCl_2_; pH 7.4). Resuspended platelets were rested for 20 min in a 37 °C water bath, after the addition of 10% (v/v) pre-warmed (37 °C) citrate-dextrose solution and 0.02 U ml^−1^ apyrase. Platelets were pelleted by centrifugation (800*g* for 5 min, brake = 4) before resuspension in pre-warmed HTGlc buffer. Addition of prostaglandin E1 (2 μM) took place immediately before all centrifugation steps to minimize platelet activation. Washed platelets were divided into 250-μl aliquots. To activate the platelets, thrombin (Sigma-Aldrich, T6884) at a final activity of 0.2 U ml^−1^ was added and incubated in a 37 °C water bath for 5 min. After incubation, D-phenylalanyl-N-[(1S)-4-[(aminoiminomethyl)amino]-1-(2-chloroacetyl)butyl]-L-prolinamide dihydrochloride (Abcam, ab141451) was added at 25 nM immediately before centrifugation as above. The supernatant at this point was regarded as the ‘platelet releasate’ and was aspirated and stored under argon at −80 °C.

For analysis of MMRN2 and EMID1 *O*-fucosylation, either pcDNA3.1-hMMRN2-FLAG or pcDNA3.1-hEMID1-FLAG was transiently transfected into HEK293T cells using Lipofectamine 3000 (Thermo Fisher Scientific) according to the manufacturer’s instructions. The FLAG-tagged proteins were isolated using Protein G magnetic beads (Thermo Fisher Scientific) and anti-FLAG M2 antibody (Sigma-Aldrich) for 2 h as described above for co-immunoprecipitation experiments. Releasate proteins and FLAG-IP eluates were digested with trypsin/lysC and peptides purified as previously described for the co-immunoprecipitation analysis. From each digest, 1 μl of peptides (from 10 μl total) was loaded onto Evotip Pure tips (EvoSep) after dilution with 0.1% FA in water according to the manufacturer’s instructions. Loaded Evotips were mounted on an EvoSep1 HPLC coupled to a 75 μm × 8 cm C18 column via an Optiflow source to a SCIEX 7600 ZenoTOF instrument with SCIEX OS (version 3.4.0.19154). Samples were separated using the 60 samples per day (SPD) LC method and analyzed on the 7600 using multiple reaction monitoring (MRM) targeting the MMRN1 peptide containing T265 (*m*/*z* = 568.3033, +2), the MMRN2 peptides containing S63 (*m*/*z* = 664.7861, +2), T67 (*m*/*z* = 588.8491, +2) and T115 (*m*/*z* = 546.3084, +2) or the EMID1 peptide containing T42 (*m*/*z* = 665.8084, +2). The source settings were as follows: 1–50 μl Micro; Curtain gas, 25; CAD gas, 7; Ion source gas 1, 12 psi; Ion source gas 2, 60 psi; Temperature, 150 °C; Column temperature, 40 °C; Spray voltage, +4,500 V. MRM was performed using electron-activated dissociation (EAD)-based fragmentation with the following settings: Q1 resolution was ‘Unit’; Zeno pulsing, True; Start Mass, 100 Th; Stop Mass, 2,000 Th; Accumulation time, 0.2–1 s; Collision energy, 10–20 V; Collision energy spread, 0 V; Declustering potential, 80 V; Time bins to sum, 16; Channels, 1/2/3/4 True; Zeno threshold, 20,000 counts per second; QJet RF amplitude, 175.2497584 V; EAD RF, 100; Electron kinetic energy, 4–8 eV; Electron beam current, 9,000 nA; and Reaction time, 40 ms. EAD and collision-induced dissociation (CID) parameters were optimized for each peptide ion. EAD fragmentation spectra were averaged across the chromatographic peak and submitted to Byonic (Protein Metrics, version 3.11.3) for identification. Either the full human proteome (UniProt) or the platelet releasate focused database containing 1,529 proteins was used^29^. An FDR of 2% using a target-decoy-based strategy was used for protein and peptide identification. MS1 and MS2 mass tolerance was set to 4 ppm and 20 ppm, respectively. Trypsin was set as the digestion enzyme with a maximum of two missed cleavages. Oxidation of Met (common 2), deamidation of Asn/Gln (common 1), pyro-Glu/Gln (rare 1) and protein N-terminal acetylation (rare 1) were set as variable modifications. Additional variable modifications all set to rare 1 for *O*-glycosylation were included: Fuc(1), HexNAc(1)Fuc(1), Hex(1)Fuc(1), HexNAc(1)Hex(1)Fuc(1), HexNAc(1)Hex(1)Fuc(1)NeuAc(1), HexNAc(1)Hex(1)NeuAc(2), Hex(1), HexNAc(1), Hex(1)Pent(2), HexNAc(1)Hex(1)NeuAc(1), HexNAc(2)Hex(2)NeuAc(2), Hex(1)Pent(3), HexNAc(2)Hex(2)NeuAc(1), HexNAc(1)Hex(1)NeuAc(3), Hex(1)NeuAc(1) and HexNAc(2)Hex(2)Fuc(1)NeuAc(1). Carbamidomethylation of Cys was set as a fixed modification. Total common max was set to 1, and total rare max was set to 2.

### GFP–FUT10, GFP–FUT11, GFP–POFUT2 and N-terminal EMI, expression and purification

Recombinant GFP–POFUT2, GFP–FUT10 and GFP–FUT11 (lacking transmembrane domain) were expressed and purified from HEK293F cells. HEK293F cells were maintained in FreeStyle 293 medium to a density of 3 × 10^6^ cells per milliliter. For transfection, 200 × 10^6^ cells were calculated and centrifuged at 157*g* for 3 min. Cell pellets were resuspended in 50 ml of 9:1 medium (FreeStyle 293 medium: EX-CELL medium (Sigma Aldrich), v/v) and transiently transfected with 200 μg of pGEn2-GFP-hPOFUT2, pGEn2-GFP-hFUT10, pGEn2-GFP-hFUT11 or pGEn2-GFP plasmids (4 μg of plasmid per milliliter) with 450 μl of PEI (1 μg μl^−1^ stock). After 24 h, valproic acid (Sigma-Aldrich) was supplemented to cells in 50 ml of 9:1 medium to a final concentration of 2.2 mM. Three days later, culture medium was collected and filtered through 0.45-μm filters (Millipore).

For generating non-fucosylated EMI substrates for enzymatic assays, 200 × 10^6^ HEK293F cells were resuspended in 50 ml of 9:1 medium containing 200 μM 6-AF and transiently transfected with 200 μg of pcDNA4-hMMRN1 N-terminal EMI-Myc-His_6_ plasmid (4 μg of plasmid per milliliter) with 450 μl of PEI (1 μg μl^−1^ stock). After 24 h, valproic acid was diluted to 2.2 mM in 50 ml of 9:1 medium containing 200 μM 6-AF and added to transfected cells. After 3 d, culture medium was collected and filtered through 0.45-μm filters.

Recombinant proteins were purified using Ni-NTA affinity chromatography with 1 ml of Ni-NTA agarose and eluted with 3 ml of 250 mM imidazole in TBS, pH 7.5. Eluted proteins were buffer exchanged to 10% glycerol in 50 mM HEPES, pH 7, with 10-kDa molecular weight cutoff centrifugal filters (Amicon) using the manufacturer’s recommended procedures and stored at −80 °C until use. Protein concentration was measured with NanoDrop (Thermo Fisher Scientific). The extinction coefficient values for individual proteins were calculated based on their respective sequences on the ProtParam tool in ExPASy^64^. The purity of proteins was verified by SDS-PAGE and Coomassie blue staining.

### Enzymatic assays for POFUT activity and kinetic analysis

For acquiring the time-dependent EMI-modifying profiles for GFP–FUT10 and GFP–FUT11, 0.1 μM recombinant GFP–FUT10, GFP–FUT11, GFP–FUT8 (positive control for *N*-glycan fucosylation, generously provided by Kelley Moremen, University of Georgia) or GFP (negative control) was incubated in 50-μl reaction mixtures containing 100 μM GDP-fucose, 0.5 μM purified non-fucosylated N-terminal EMI and 50 mM HEPES, pH 7. Reactions were incubated at 37 °C for indicated times and stopped by adding 500 μl of cold acetone. Reaction products were analyzed with nano LC–MS/MS as described earlier.

The GDP-Glo Glycosyltransferase assay is a common method for determining kinetic parameters of glycosyltransferases. However, we encountered a challenge in quenching the activity of GFP–FUT11 without impacting the luciferases required for luminescence signal generation, possibly due to the strong affinity between FUT11 with EMI, which enables the enzyme to remain active even in the presence of detergent. As a solution, we used mass spectrometric analysis to quantify the fucosylation stoichiometry and subsequently convert it into kinetic parameters. Considering the inherent ionization suppression effects of glycopeptides, the calculated parameters could potentially underestimate the actual values.

For acquiring the EMI substrate concentration-dependent kinetics of GFP–FUT10 and GFP–FUT11, 50 nM recombinant GFP–FUT10 or GFP–FUT11 was incubated in 30-μl reaction mixtures containing 100 μM Ultra Pure GDP-fucose (Promega), 0.3 mM MnCl_2_ with 0.156 μM, 0.313 μM, 0.625 μM, 1.25 μM, 2.5 μM, 5 μM, 10 μM, 20 μM or 30 μM purified non-fucosylated N-terminal EMI in 50 mM HEPES, pH 7. Reactions were incubated at 37 °C for 15 min and stopped by adding 500 μl of cold acetone. For negative control samples, reaction mixtures containing 1.25 μM EMI substrate but without enzyme were precipitated with acetone first, followed by adding GPF–FUT10 or GFP–FUT11 to a final concentration of 50 nM. To obtain GDP-fucose concentration-dependent kinetic data, a saturating concentration of EMI is required. For GFP–FUT10, this concentration exceeds 30 μM, which would require an impractical amount of 6-AF to produce enough EMI substrate. In contrast, 20 μM EMI is sufficient to saturate GFP–FUT11, so we conducted the GDP-fucose concentration-dependent kinetics for GFP–FUT11. Next, 50 nM recombinant GFP–FUT11 was incubated in 30-μl reaction mixtures containing 20 μM purified non-fucosylated N-terminal EMI, 0.3 mM MnCl_2_ with 0 μM (negative control), 1.563 μM, 3.125 μM, 6.25 μM, 12.5 μM, 25 μM, 50 μM, 100 μM or 200 μM Ultra Pure GDP-fucose in 50 mM HEPES, pH 7. Reactions were incubated at 37 °C for 15 min and stopped by adding 300 μl of cold acetone. The acetone-precipitated proteins were reduced, alkylated, digested with trypsin and analyzed with nano LC–MS/MS using the procedures described earlier. Data analysis with Byonic, EIC generation and quantification were performed as described earlier. The conversion of the relative abundance of fucosylation to product concentrations was performed using the following equation:

Product concentration = (Fuc% − Fuc%_background_) × EMI substrate concentration (Fuc%: relative abundance of fucosylation; Fuc%_background_: relative abundance of fucosylation in negative control samples).

The kinetic curve was plotted using product formation rate per enzyme (nmol min^−1^ mg^−1^) with EMI or GDP-fucose substrate concentration (μM). The kinetic parameters were determined with nonlinear Michaelis–Menten least squares (ordinary) fitting in Prism 7. All reactions were performed in biological triplicates with three batches of purified enzymes.

### EMI unfolding assays

Purified non-fucosylated N-terminal EMI (20 μg; prepared as described above) or hTHBS1 TSR3 (bacterially expressed) was reduced in 100 μl of buffer containing 0.4 M ammonium bicarbonate and 8 M urea with or without 5 mM TCEP and incubated at 50 °C for 15 min. After cooling down to room temperature, 20 μl of 200 mM iodoacetamide or water was added. Samples were incubated at room temperature for 40 min in the dark. Samples were then buffer exchanged to 100 μl of 10% glycerol in 50 mM HEPES, pH 7, using 10-kDa molecular weight cutoff Zeba spin column (Thermo Fisher Scientific) according to the manufacturer’s recommended procedures. Protein concentrations were measured by NanoDrop with extinction coefficient number of 10.6% (calculated with the ProtParam tool in ExPASy using the protein sequence of N-terminal EMI). Proteins were stored at −80 °C until use. Unmodified human THBS-1 TSR3 was expressed and purified from *Escherichia coli* BL21 strain (Invitrogen) as previously described^49^. Unfolded and folded proteins were verified by SDS-PAGE and Coomassie blue staining.

Enzymatic assays with folded or unfolded substrates were performed in 50-μl reaction containing 0.1 μM purified enzyme (recombinant GFP–FUT10/11 or GFP–POFUT2), 0.5 μM folded or unfolded substrate (N-terminal EMI or hTHBS1 TSR3), 100 μM GDP-fucose and 50 mM HEPES, pH 7. Reactions were incubated at 37 °C for 0 h, 1 h, 2 h or 4 h before being stopped with 500 μl of cold acetone. Reaction products were reduced, alkylated, digested with trypsin (for N-terminal EMI) or double-digested with trypsin/chymotrypsin (for hTHBS1 TSR3) and analyzed with nano LC–MS/MS using the procedures described earlier. All reactions were performed in biological triplicates with three batches of purified enzymes.

### Generation of CRISPR–Cas9-mediated genome editing

CRISPR–Cas9 HEK293T KOs of *POFUT1*, *POFUT2* and *SLC35C1* were generated in Takeuchi et al., Benz et al. and Lu et al.^27,34,52^.

*FUT10* KO, *FUT11* KO, *FX* KO and *FUT10/11* DKO cells were generated using modified CRISPR–eSpCas9 plasmids^65^. Each plasmid encoded the eSpCas9 nuclease, mCherry, a puromycin resistance gene and a gene-specific guide RNA. HEK293T cells seeded in a six-well dish were transfected with 3 μg of the two indicated gene-specific plasmids encoding guide RNAs (Supplementary Table 2) using 10 μl of Lipofectamine 2000 transfection reagent (Invitrogen, 11668-019) according to the manufacturer’s protocol. After 96 h, mCherry-positive cells were sorted using a Bio-Rad S3 Sorter (CTEGD Cytometry Shared Resource Laboratory, University of Georgia), and single cells were acquired by limiting dilution in 96-well plates. Cells were cultured for 3 weeks in DMEM medium with 10% BCS and 1% penicillin–streptomycin and supplemented with 25% of 0.2-μm-filtered 3 d cultured HEK293T cells medium. Genomic DNA was isolated from individual clones and WT cells. Sequences flanking the guide RNA were amplified using Hot Start Taq DNA Polymerase (NEB, M0481L). PCR products were denatured, reannealed with denatured PCR products from WT and digested with T7 endonuclease I (NEB, M0302L) according to the manufacturer’s protocol. Candidate clones were transfected with plasmid expressing N-terminal EMI and screened by mass spectrometric analysis for their fucosylation level. PCR products from selected clones with low EMI fucosylation level were ligated into pGEM-T Easy Vector System (Promega, A1360) using the manufacturer’s manual and transformed into *E. coli* DH5α strain (Invitrogen), and colonies were isolated. Plasmids were isolated from 8–10 colonies, and the inserts were sequenced. PCR primer pairs and guide RNAs are listed in Supplementary Table 2.

### Western blot–based secretion assay

To compare the secretion of MMRN1 WT and mutants, HEK293T WT cells were seeded in six-well dishes (0.8–1 × 10^6^ cells per well) or 10-cm dishes (8 × 10^6^ cells per dish) and incubated overnight to reach a confluency of 80%. Medium was changed to Opti-MEM before transfection. Transient transfection was performed using PEI (6 μl of PEI per 1 μg of plasmid). For six-well dishes, cells were transfected with 2 μg per well of pcDNA3.1-hMMRN1 WT-Myc-His_6_, pcDNA3.1-hMMRN1 T216A-Myc-His_6_, pcDNA3.1-hMMRN1 T265A-Myc-His_6_, pcDNA3.1-hMMRN1 T216A/T265A-Myc-His_6_, pcDNA3.1-hMMRN1 T1055A-Myc-His_6_ or empty vector, together with 0.1 μg per well of IgG plasmid as secretion control. For 10-cm dishes, cells were transfected with 10 μg per dish of the respective plasmids and 0.5 μg per dish of IgG plasmid. Medium samples were collected after 48 h of incubation. Lysate samples were collected after 24 h of incubation to ensure clear visualization of MMRN1 bands in western blot images.

To compare N-terminal EMI secretion in HEK293T WT and *FUT10/11* DKO cells, HEK293T WT or *FUT10/11* DKO cells were seeded in six-well dishes (1 × 10^6^ cells per well) or 10-cm dishes (8 × 10^6^ cells per dish) and incubated overnight to reach a confluency of 80%. Transient transfection was performed using PEI (6 μl of PEI per 1 μg of plasmid). For six-well dishes, cells were transfected with 0.5 μg per well of pcDNA4-hMMRN1 N-terminal EMI-Myc-His_6_ or empty vector, together with 0.1 μg per well of IgG plasmid. For 10-cm dishes, cells were transfected with 4 μg per dish of pcDNA4-hMMRN1 N-terminal EMI-Myc-His_6_ or empty vector, together with 0.8 μg per dish of IgG plasmid. Cells were incubated for 48 h before collecting medium and lysate samples.

To compare protein secretion in HEK293T WT and *FX* KO cells, HEK293T WT or *FX* KO cells (1 × 10^6^ cells per well) were seeded in six-well dishes and incubated overnight to reach a confluency of 80%. Transient transfection was performed using PEI (6 μl of PEI per 1 μg of plasmid) with 0.5 μg per well of pcDNA4-hMMRN1 N-terminal EMI-Myc-His_6_, 1 μg per well of pSec-mNOTCH1 EGF1–18-Myc-His_6_, 1 μg per well of pSec-hADAMTS9 TSR2–8-Myc-His_6_ or empty vector, together with 0.1 μg per well of IgG plasmid as secretion control. Cells were incubated for 48 h before collecting medium and lysate samples.

Media (100 μl) were precipitated with 500 μl of cold acetone, incubating overnight at −20 °C. After centrifuging at 18,213*g* for 15 min at 4 °C, pellets were resuspended in 12 μl of 2× reducing sample buffer (containing 4% SDS, 200 mM 2-mercaptoethanol, 20% glycerol in 100 mM Tris/HCl, pH 6.8) by sonication for 10 min and boiled at 105 °C for 5 min. For collecting lysate samples, transfected cells were freeze–thawed with one cycle at −20 °C to break down long DNA chains. Cell pellets were lysed with 200 μl of 1% NP-40/TBS or RIPA buffer (Thermo Fisher Scientific, 89900) supplemented with cOmplete protease inhibitors EDTA-free (Roche), incubated on ice for 15 min and centrifuged at 18,213*g* for 5 min at 4 °C. Cleared supernatants were collected. Protein concentrations were measured using BCA assay according to the manufacturer’s protocol (Thermo Fisher Scientific, 23225). Then, 40–80 μg of cell lysate was used for analysis. Samples were loaded onto 4–20% SDS-PAGE gels (Bio-Rad) and transferred to a nitrocellulose membrane. Membranes were blocked with 5% non-fat milk (Bio-Rad) for 30 min at room temperature, followed by incubation with anti-Myc antibody (Invitrogen, clone 9E10, 1:2,500) or co-incubation with anti-Myc antibody (Invitrogen, clone 9E10, 1:2,500) and anti-His antibody (Bio-Rad, MCA1396, 1:2,500) at 4 °C overnight. Membranes were then incubated with IDRye 800-conjugated goat anti-mouse IgG antibody (LI-COR, 1:2,500) and IDRye 680-conjugated goat anti-human IgG antibody (LI-COR, 1:2,500) for 1 h at room temperature. The western blot bands were visualized and quantified using an Odyssey system (LI-COR) with LI-COR Image Studio (version 5.2.5).

### Confocal immunofluorescence microscopy

HeLa cells (ATCC, CCL-2) were plated onto coverslips until they reached approximately 50% confluency before fixation. Human induced pluripotent cells, SCTI003-A, were obtained from STEMCELL Technologies. Cells were maintained in feeder-free conditions using Matrigel-coated plates and MTSER Plus (STEMCELL Technologies). Cells were passaged in bulk using ReleSR (STEMCELL Technologies) during expansion and/or maintenance. For imaging, single-cell suspensions were prepared using Accutase (STEMCELL Technologies), and media were supplemented with 10 μM ROCK inhibitor (Y27632) less than 24 h after plating onto Matrigel-coated coverslips. HeLa/stem cells were washed once with PBS and fixed with 4% PFA for 15 min at room temperature. Coverslips were washed with PBS three times and permeabilized with 0.1% SDS (in PBS) for 4 min at room temperature. Coverslips were washed five times with PBS, and non-specific binding was blocked with Dako Protein serum-free ready-to-use buffer (Agilent, X0909). Coverslips were then incubated with primary antibodies overnight at 4 °C. Primary antibodies used were mouse anti-PDI (Thermo Fisher Scientific, MA3-019, 1:100), mouse anti-GM130 (BD Biosciences, 610823, 1:500), rabbit anti-FUT10 (Proteintech, 18660-1-AP, 1:200) and rabbit anti-FUT11 (Proteintech, 17175-1-AP, 1:200). Coverslips were washed with PBS five times and incubated with highly cross-absorbed secondary antibodies at 1:500 for 2 h at room temperature. Secondary antibodies used included goat anti-rabbit IgG (H+L) with Alexa Fluor 488 and goat anti-mouse IgG (H+L) with Alexa Fluor 647 (Thermo Fisher Scientific). The coverslips were then washed five times with PBS and mounted onto glass slides with Prolong Diamond Antifade (with DAPI) (Thermo Fisher Scientific) and allowed to cure overnight at room temperature before imaging. Slides were imaged using a Leica LCS SP8 confocal microscope with a ×93 magnification glycerol lens using a white laser. Leica LAS X software (version 5.2) was used for image acquisition. Co-localization analysis was done using the Coloc 2 plugin from Fiji ImageJ (version 1.54f), with regions of interest drawn around cells, using Costes’ automatic thresholds.

### CD

CD spectra of purified GFP–FUT10 and GFP–FUT11 were acquired with a Jasco J-715 spectra polarimeter with a Peltier temperature controller and a cuvette with 1-mm path length. Data were collected with Spectra Manager (version 1.08.01, Build 1). The protein samples consisted of 5.8 μM GFP–FUT10 or 6.1 μM GFP–FUT11 in 3.3% glycerol and 16.7 mM HEPES, pH 7. Data were collected at 20 °C from 260 nm to 190 nm in 1-nm increments with a scan rate of 100 nm min^−1^. Data fitting from the CD data was performed via the DichroWeb site using the CDSSTR algorithm^66^.

### Thermostability assay

FUT10 and FUT11 were thermally unfolded, and melting points were determined using nano differential scanning fluorimetry (nanoDSF). In brief, recombinant GFP–FUT10 and GFP–FUT11 proteins were diluted to 100 ng μl^−1^ in PBS, and 10-μl aliquots were taken up into NT.48 Series nanoDSF Grade High Sensitivity Capillaries (NanoTemper Technologies). Thermal unfolding was measured using the nanoDSF function of a Prometheus Panta (NanoTemper Technologies) with Panta Analysis software (version 1.1). Temperature was increased from 15 °C to 90 °C at a rate of 0.5 °C min^−1^. Samples were excited at a wavelength of λ = 280 nm, and tryptophan fluorescence emissions were monitored at λ = 330 nm and λ = 350 nm. Protein melting point (T_m_) was determined using Panta Analysis software (NanoTemper Technologies) and defined as the inflection point of the ratio of fluorescence emission (F350/F330) as a product of temperature.

### Analysis of *N*-glycans on N-terminal EMI by PNGase F digestion

HEK293T cells (6 × 10^6^) were seeded and grown to 70% confluence in 10-cm dishes. Cells were transiently transfected with 5 μg of pcDNA4-hMMRN1 N-terminal EMI-Myc-His_6_ or 2 μg of pSecTag2-mLfng-Myc-His_6_ using PEI (6 μl per 1 μg of DNA, 1 μg μl^−1^ stock) in 8 ml of Opti-MEM (Thermo Fisher Scientific, 31985088). After 2 d, cell media from 3–5 plates of cells were combined. Recombinant proteins were purified using 600 μl of Ni-NTA agarose (Qiagen) and eluted with 600 μl of 250 mM imidazole in TBS, pH 7.5. Then, 50 μl of Ni-NTA elution was precipitated with 250 μl of cold acetone and incubated at −20 °C for 2 h. Samples were centrifuged at 18,213*g* for 15 min at 4 °C. Protein pellets were resolubilized with 20 μl of reduction buffer containing 1% SDS, 10 mM TCEP and 0.4 M NH_4_HCO_3_ with vigorous shaking. Proteins were boiled at 105 °C for 5 min and cooled to room temperature. Next, 10 μl of 100 mM iodoacetamide (Sigma-Aldrich) was added, and samples were incubated in the dark for 40 min. Then, 170 μl of 1% NP-40/TBS was added to dilute SDS to a final concentration of 0.1%. Next, 1.5 μl of PNGase F (20 U μl^−1^, Lectez Bio) was added to samples and incubated at 37 °C for more than 6 h. Samples were precipitated again with 1 ml of cold acetone and resolubilized with 12 μl of 2× reducing sample buffer (containing 4% SDS, 200 mM 2-mercaptoethanol, 20% glycerol in 100 mM Tris/HCl, pH 6.8) by sonication for 10 min and boiled at 105 °C for 5 min. Proteins were analyzed with western blot probed with anti-Myc antibody (Invitrogen, clone 9E10, 1:2,500).

### Metal ion screening assay

Recombinant GFP–FUT10 or GFP–FUT11 (0.1 μM) was incubated in 50 μl of reaction mixtures containing 100 μM GDP-fucose, 0.5 μM purified non-fucosylated N-terminal EMI and varied concentrations of divalent metal ions (MnCl_2_, MgCl_2_ or CaCl_2_, dissolved in water) or 5 mM EDTA in 50 mM HEPES, pH 7. Reactions were incubated at 37 °C for 4 h and stopped by adding 250 μl of cold acetone. Proteins were incubated with acetone at −20 °C for 2 h and centrifuged at 18,213*g* at 4 °C for 15 min. Protein pellets were reduced, alkylated, digested with trypsin and analyzed with nano LC–MS/MS using the procedures described earlier. Data analysis with Byonic, EIC generation and quantification were performed as described earlier.

### GDP-Glo Glycosyltransferase assay

Recombinant GFP–FUT10 (50 nM) was incubated in 20-μl reaction mixtures containing 100 μM Ultra Pure GDP-fucose (Promega), 0.3 mM MnCl_2_ with 0 μM (negative control), 0.156 μM, 0.313 μM, 0.625 μM, 1.25 μM, 2.5 μM, 5 μM, 10 μM, 20 μM or 30 μM purified non-fucosylated MMNR1 N-terminal EMI in 50 mM HEPES, pH 7. Reactions were incubated at 37 °C for 15 min, and samples were immediately put on ice. According to the manufacturer’s guidelines, nucleotide detection reaction buffer (NDR; Promega) could stop enzyme activity, followed by a 1-h incubation period before luminescence detection. However, we observed that FUT10 still exhibited weak activity by following the above method. We modified the protocol and measured luminescence right after adding 20 μl of the NDR buffer (with GDP Glo enzyme). Luminescence was collected on a BioTek Cytation 5 with BioTek Gen5 (version 3.03). The GDP standard (Promega) used in parallel showed a strong linear relationship with an *r*^2^ value over 0.999 (data not shown), indicating that the luminescence is proportional to the GDP concentration in the reactions. Produced GDP was quantified based on GDP standards and converted to desired units (nmol min ^−1^ mg^−1^). The kinetic parameters were determined with nonlinear Michaelis–Menten least squares (ordinary) fitting in Prism 7. All reactions were performed in biological triplicates with three batches of purified enzymes.

### Reporting summary

Further information on research design is available in the Nature Portfolio Reporting Summary linked to this article.

## Online content

Any methods, additional references, Nature Portfolio reporting summaries, source data, extended data, supplementary information, acknowledgements, peer review information; details of author contributions and competing interests; and statements of data and code availability are available at 10.1038/s41589-024-01815-x.

## Supplementary information

Supplementary Figs. 1–25, Tables 1 and 2 and the raw gel images used in Supplementary Figs. 3, 6, 9 and 22–24.

Reporting Summary

Supplementary Data 1 Annotated HCD–MS/MS spectra for peptides used in the generation of EICs in Figs. 1d, 2 and 6b, Extended Data Fig. 7 and Supplementary Figs. 2 and 3.

Supplementary Data 2 AlphaFold2-multimer PAE plots for the predicted EMI domain and fucosyltransferases structures.

Supplementary Data 3 Statistical source data for supplementary figures.

## Source data

Source Data Fig. 3 Statistical source data.

Source Data Fig. 4 Statistical source data.

Source Data Fig. 5 Statistical source data.

Source Data Fig. 6 Statistical source data.

Source Data Fig. 6 Unprocessed western blots.

## Data Availability

Confocal images in Extended Data Fig. 8c were obtained from the Human Protein Atlas (https://www.proteinatlas.org/ENSG00000172728-FUT10/subcellular). All data generated and analyzed during this study are provided within the paper and the Supplementary Data. Note for Supplementary Data 1: Annotated HCD–MS/MS spectra for peptides used in the generation of EICs in Figs. 1d, 2 and 6b, Extended Data Fig. 7 and Supplementary Figs. 2 and 3. Due to the lability of the fucose–peptide bond in HCD, Byonic software is frequently unable to correctly assign the *O*-fucosylation site when multiple serine or threonine residues are present in a peptide. All assignments are based on the well-documented sequences for *O*-fucosylation of EGF repeats (C^2^XXXX[S/T]C^3^) and group 1 TSRs (C^1^XX[S/T]C^2^). Note for Supplementary Data 2: AlphaFold2-multimer PAE plots for the predicted EMI domain and fucosyltransferases structures. Source data are provided with this paper.
