# Supplementary material for: FUT10 and FUT11 are protein O-fucosyltransferases that modify protein EMI domains
Source: Nat Chem Biol. 2025 Jan 7;21(4):598–610. doi: 10.1038/s41589-024-01815-x (PMC11949838; doi:10.1038/s41589-024-01815-x)
Supplement: Supplementary file 1 — Supplementary Figs. 1–25, Tables 1 and 2 and the raw gel images used in Supplementary Figs. 3, 6, 9 and 22–24. [file 41589_2024_1815_MOESM1_ESM.pdf]

nature chemical biology

Supplementary information https://doi.org/10.1038/s41589-024-01815-x

FUT10 and FUT11 are protein *O*-fucosyltransferases that modify protein EMI domains

In the format provided by the authors and unedited

Supplementary Figures and Tables

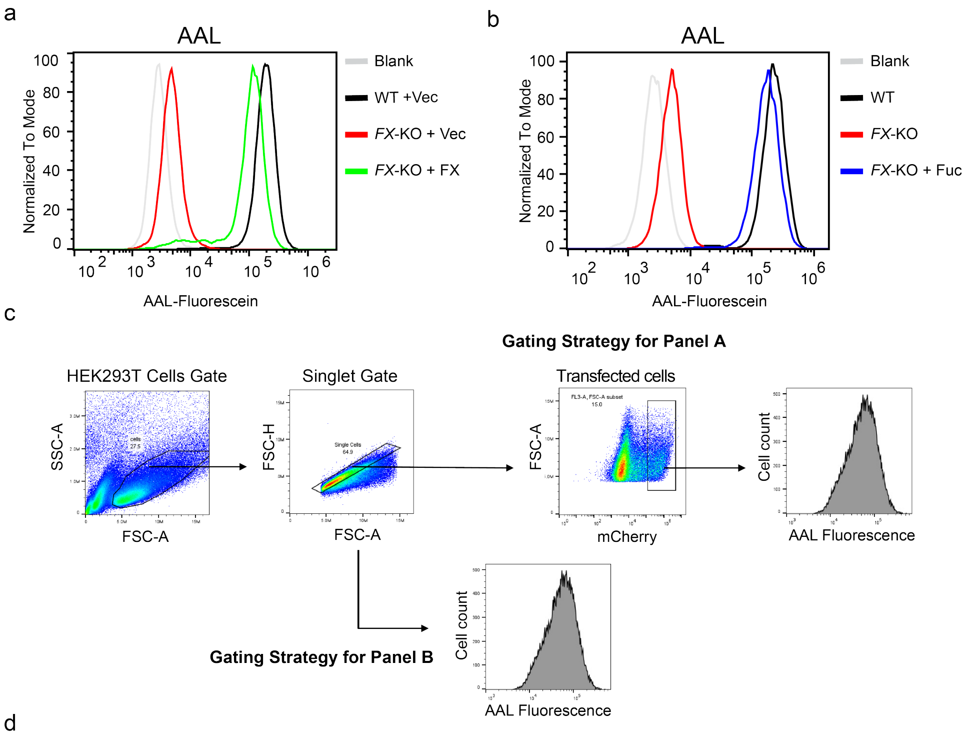

d

FX KO (clone 3):
Allele 1: 61-bp deletion
Allele 2: 17-bp deletion and 28-bp insertion

```
WT(gRNA1 and 3): TTTGTCTCCTCTAAAGACGCCGATCTCACGTGAGTGAGCCCATCCCCAGATCAGCCCTTCCTCTCCATCCTACCC
Allele 1 :       TTTGTCTC--------------------CGTGAGTGAGCCCATCCCCAGATCAGCCCTTCCTCTCCATCCTACCC
Allele 2 :       TTTGTCTCCTCTAAAGACGCCGATCTCACGTGAGTGAGCCCATCCCCAGATCAGCCCTTCCTCTCCATCCTACCC

WT(gRNA1 and 3): CCCACCCTAG...268bp...CTGACAGCCTGGCTTCCTACAGGGATACAGCACAGACCCGCGCCCTGTTTGA
Allele 1:        CCCACCCTAG...268bp...CTGACAGCCT-----------------------------------------A
Allele 2:        CCCACCCTAG...268bp...CTGACAGCCTGGCTTCCTACAGGGATACAGCACAG-----------------

WT(gRNA1 and 3):                                GAAGGTCCAACC
Allele 1:                                       GAAGGTCCAACC
Allele 2:       (GGCGCGGGTCTGTGCTGTATCCCTGTAG)GAAGGTCCAACC

        Underlined: guide RNA         -: Deletion         (NNN): Insertion
```

Supplementary Fig. 1. Generation of *FX* KO HEK293T cells using CRISPR/Cas9. a, *FX* KO was rescued by transfection with a plasmid encoding FX using the fucose specific lectin, Aleuria aurantia lectin (AAL). Flow cytometric analysis of Fluorescein-conjugated AAL binding to WT and *FX* KO HEK293T cells transfected with a plasmid encoding FX protein or transfected with empty vector (Vec) as described in Lu et al. 2023[1]. For negative control (Blank), WT cells were premixed with 5 mM L-Fucose to block AAL binding before adding fluorescent AAL. **b,** *FX* KO was rescued by incubating cells with 100 μM L-Fucose (Fuc). AAL binding to WT and *FX* KO cells grown for 3 days in medium supplemented with or without 100 μM Fuc prior to flow cytometric analysis. Data was collected with BD Accuri C6 and analyzed with Flowjo (10.8.1). In panels **a** and **b**, the loss of AAL binding to *FX* KO cells (red peak vs. black peak) indicates depletion of the cellular GDP-fucose pool. This loss can be rescued by either transfecting a plasmid encoding FX (panel **a**, green peak), thus restoring the *de novo* synthesis pathway, or by feeding cells with fucose (panel **b**, blue peak), which restores GDP-fucose pool through the salvage pathway. This data indicates the loss of FX function in the KO cells, resulting in the inhibition of the GDP-fucose *de novo* synthesis pathway and depletion of the cellular GDP-fucose pool. **c,** Gating Strategy used in panel **a** and **b**. **d,** Genomic sequences of *FX* amplified from WT and *FX* KO HEK293T cells. Primers to amplify regions surrounding gRNA sequences (underlined) are in Table S2. No WT sequences were detected in *FX* KO clone 3. Two altered *FX* alleles were detected in *FX* KO clone 3.

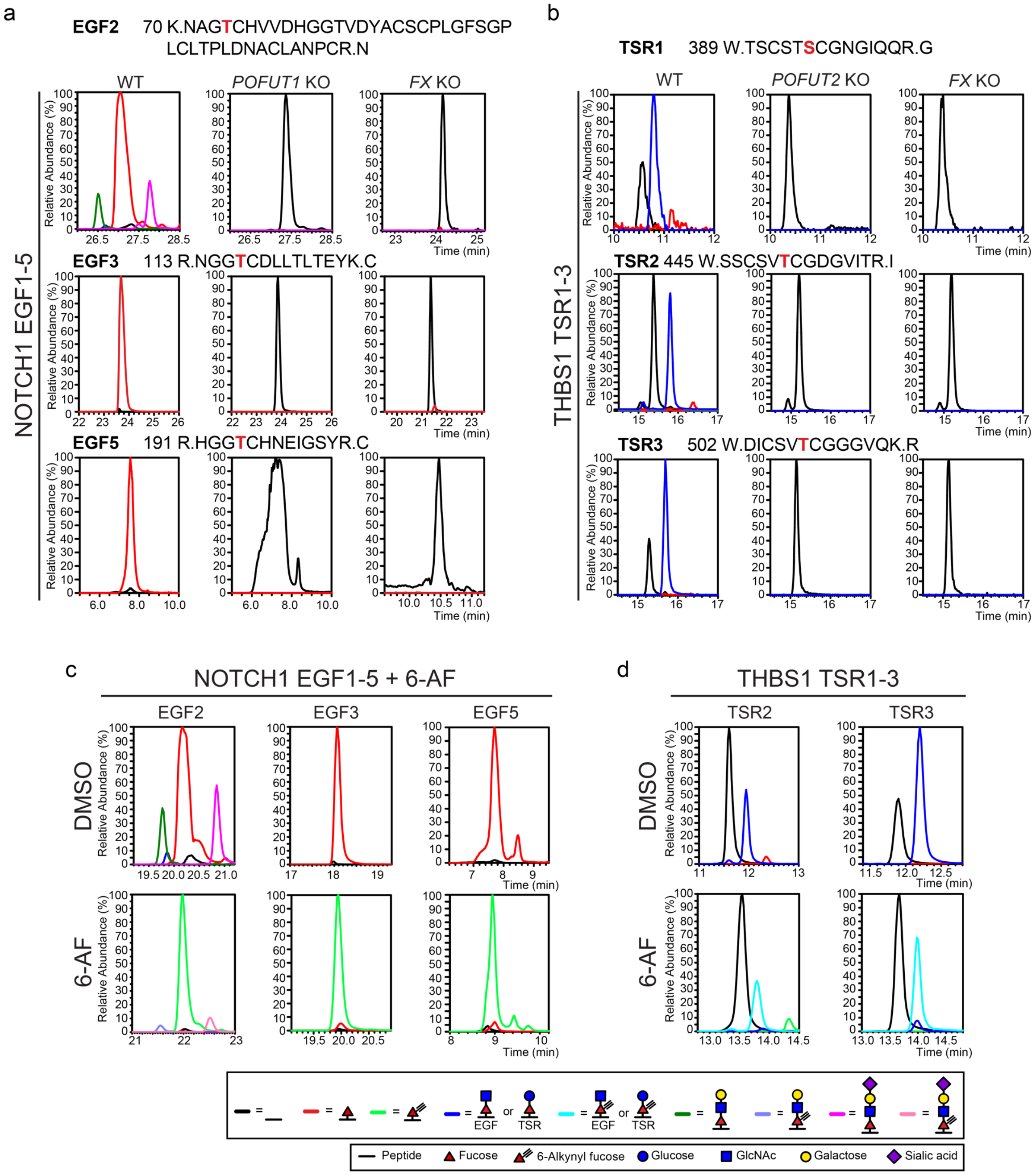

Supplementary Fig. 2. Mass spectrometric analysis of peptides from transfected mNOTCH1 EGF1-5 and hTHBS1 TSR1-3 as positive controls for the data shown in Fig. 2. a, EICs of different glycoforms of peptides from EGF2, 3, and 5 of mouse NOTCH1 EGF1-5 expressed and purified in WT, *POFUT1* KO, or *FX* KO HEK293T cells, demonstrating the loss of *O*-fucose on known POFUT1 substrate when knocking out *POFUT1* or *FX*. **b,** EICs of different glycoforms of peptides from TSR1, 2, and 3 of human THBS1 TSR1-3 expressed and purified in WT, *POFUT2* KO, or *FX* KO HEK293T cells, demonstrating the loss of *O*-fucose on known POFUT2 substrate when knocking out *POFUT2* or *FX*. **c,** EICs of peptides from EGF2, 3, and 5 of mouse NOTCH1 EGF1-5 expressed and purified in WT HEK293Tcells incubated with 6-alkynyl fucose (6-AF) or equal volume of DMSO, showing the incorporation of 6-AF to known POFUT1 substrate. **d,** EICs of peptides from TSR1, 2, and 3 of human THBS1 TSR1-3 expressed and purified in WT HEK293T cells incubated with 6-AF or equal volume of DMSO, demonstrating the incorporation of 6-AF to known POFUT2 substrate. Spectra for the corresponding ions are in Data S1.

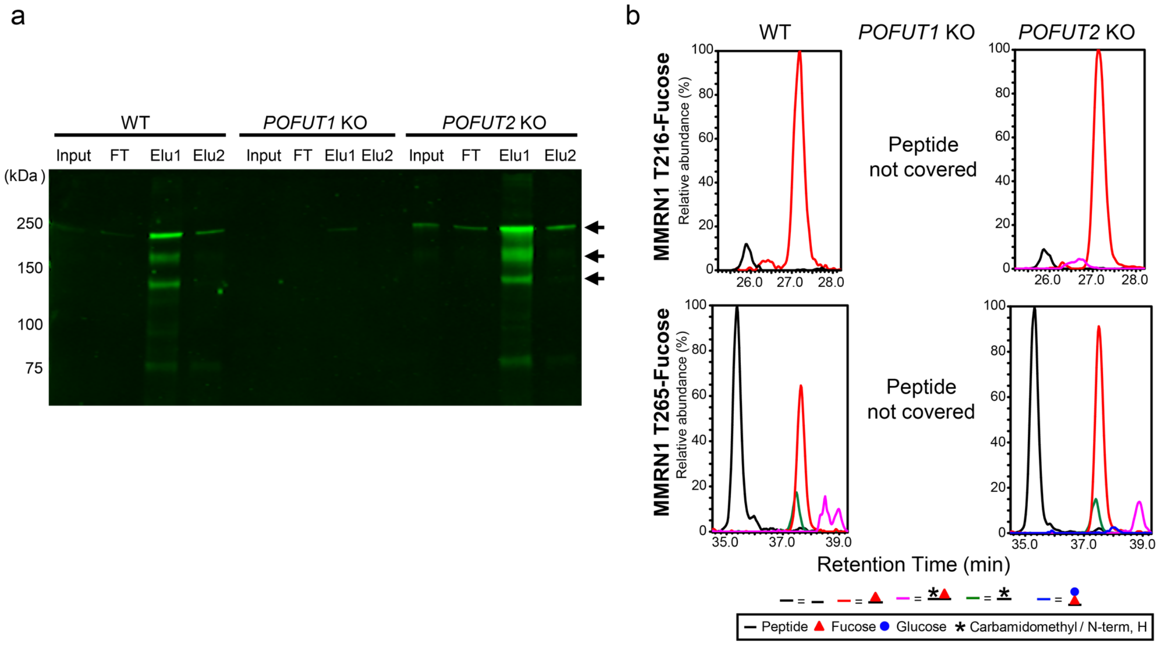

Supplementary Fig. 3. Mass spectrometric analysis of peptides from full-length hMMRN1 expressed and purified from WT, *POFUT1* KO, and *POFUT2* KO HEK293T cells. a, WT, *POFUT1* KO or *POFUT2* KO HEK293T cells were transfected with a plasmid encoding human MMRN1. After 2 days, MMRN1 was purified from conditioned media using Ni-NTA agarose as described in Materials and Methods. MMRN1 was analyzed by Western blot probed with anti-Myc and anti-His antibodies (n=2). Arrows indicate different forms of MMRN1. FT: flowthrough from Ni-NTA purification; Elu1: first elution; Elu2: second elution. **b,** MMRN1 from panel **a** was reduced, alkylated, digested with trypsin, and analyzed by mass spectrometry. EICs of different glycoforms of the peptides containing T216 or T265 *O*-fucose sites were generated. Spectra for the corresponding ions are in Data S1.

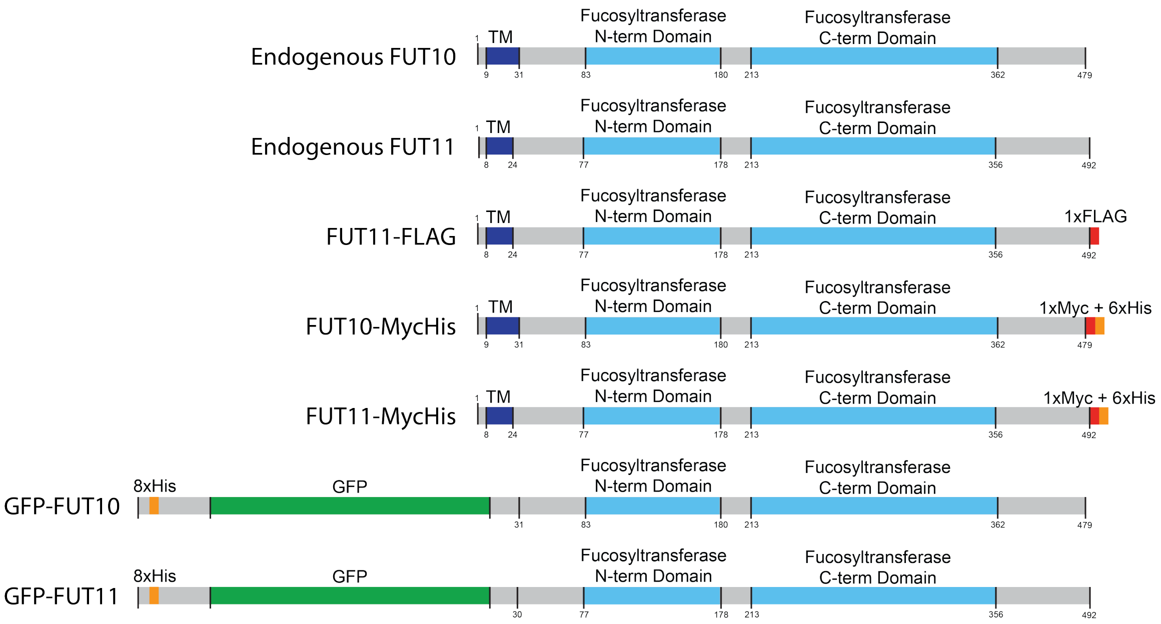

Supplementary Fig. 4. Domain comparison of endogenous FUT10/11 with full-length FUT11-FLAG, full-length FUT10/11-MycHis and GFP-FUT10/11 used in this paper. The residue numbers shown are for the endogenous human FUT10 and FUT11 proteins. Protein domains are illustrated: light blue – fucosyltransferase; green – GFP-tag; red and orange – peptide purification tags; dark blue – transmembrane (TM) domain.

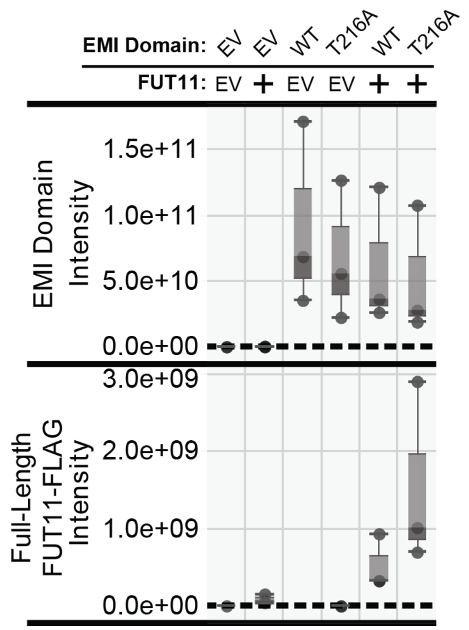

Supplementary Fig. 5. Co-immunoprecipitation of N-terminal EMI-Myc with full length FUT11 protein. Boxplot showing the immunoprecipitation of N-terminal EMI-Myc using anti-Myc agarose that was either wildtype (WT), or a T216A mutant. These proteins were isolated from HEK293 cells that were transiently transfected with either empty vector (EV), or the EMI-expressing vectors, with or without FUT11-FLAG vector as shown in the top legend (n=3). For the boxplot each circle is a biological replicate derived from an individual culture and the boxes represent the median/quartiles and whiskers represent 1.5X the interquartile range.

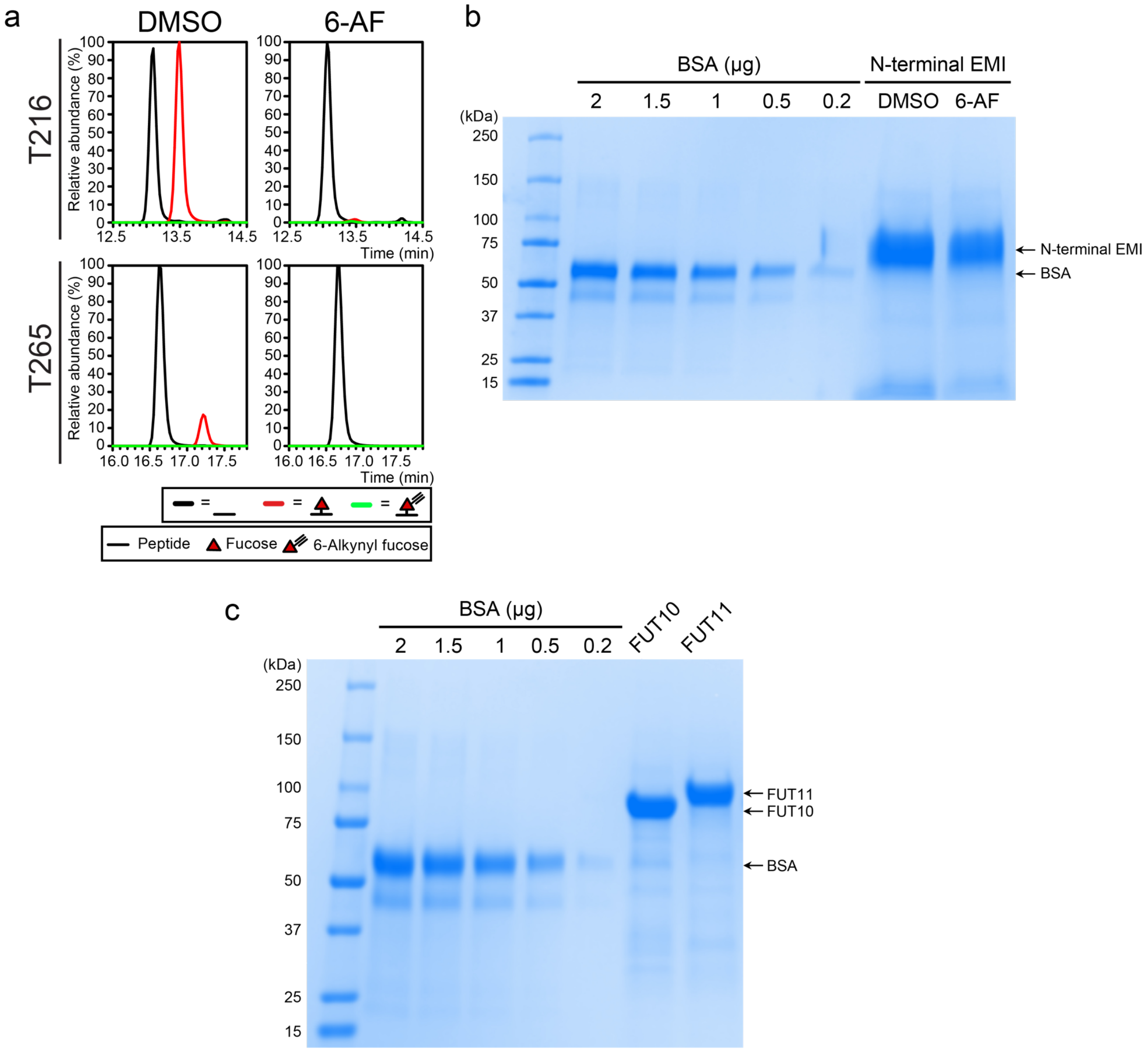

Supplementary Fig. 6. Purified, recombinant FUT10, FUT11, and non-fucosylated N-terminal EMI used for enzymatic assays. a, EICs of different glycoforms of peptides containing the T216 or T265 *O*-fucose site from N-terminal EMI produced in HEK293F cells incubated with 6-AF or equal volume of DMSO, demonstrating the non-fucosylated status of N-terminal EMI purified from 6-AF treated HEK293F cells. 300 μL of conditioned culture medium was used for mass spectrometric analysis. **b,** The N-terminal EMI produced in panel **a** was purified by Ni-NTA agarose as described in Materials and Methods. The purity of protein was verified by Coomassie blue staining. 35 μL Ni-NTA elution (6% of total purified proteins from a 100 mL cell culture) was used for testing (n=3). **c,** Coomassie blue staining of GFP-FUT10 and GFP-FUT11 expressed and purified from HEK293F cells as described in Materials and Methods. 10 μL Ni-NTA elution (2% of total purified proteins produced from a 100 mL cell culture) was used for testing (n=3).

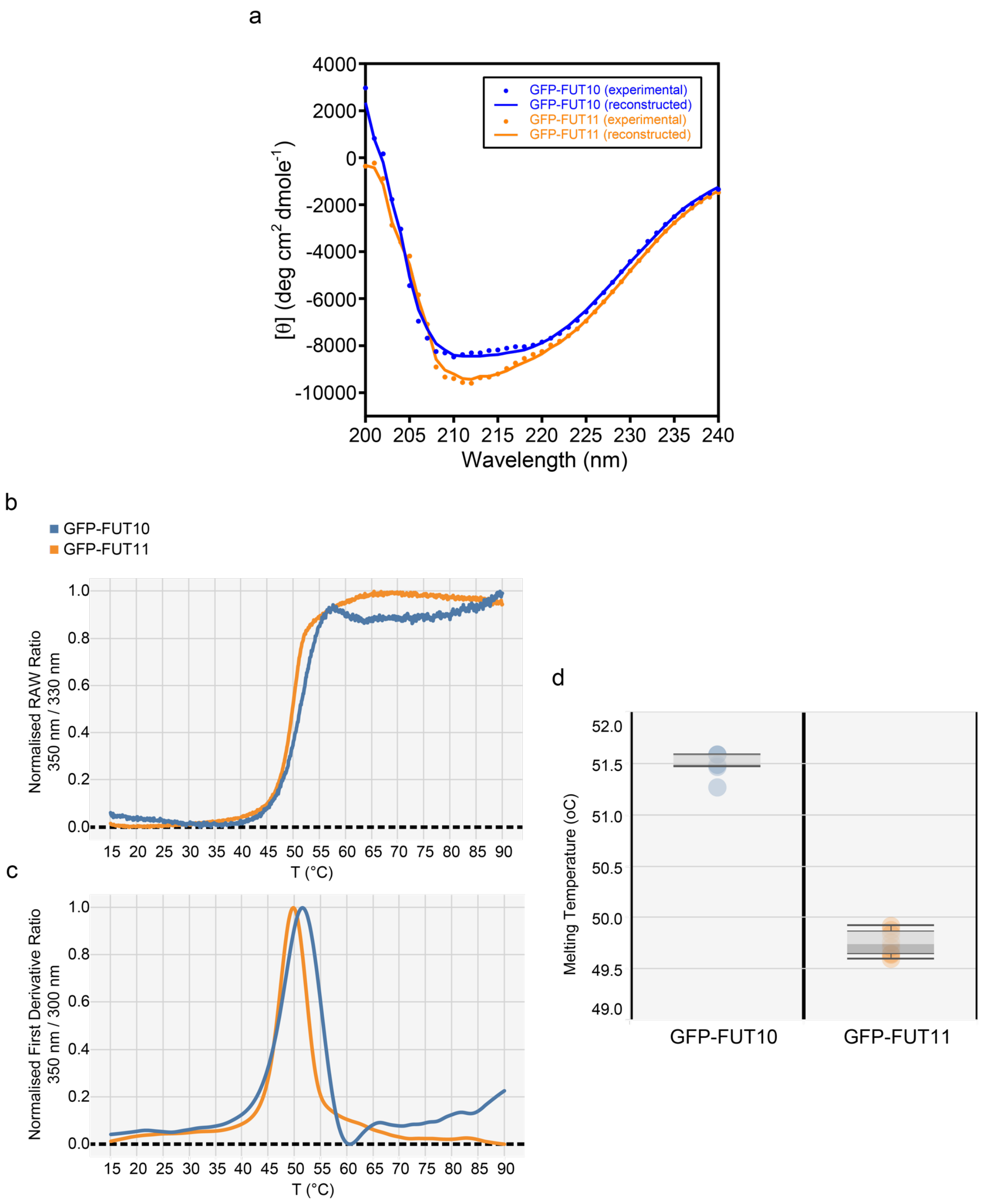

Supplementary Fig. 7. Comparable quality of purified FUT10 and FUT11 measured using circular dichroism (CD) spectroscopy and tryptophan fluorescent thermostability assays. a, CD spectra of purified GFP-FUT10 and GFP-FUT11. Symbols represent experimental data and lines represent the fits of data as described in Supplementary Materials and Methods. **b,** Protein thermal unfolding analysis of FUT10 and FUT11. Purified GFP-FUT10 and GFP-FUT11 were thermally unfolded and melting points were determined using nano-differential scanning fluorimetry (nanoDSF). Samples were excited at a wavelength of 280 nm and fluorescent emissions monitored at 330 nm and 350 nm. Data is expressed as the normalized emission ratio as a mean of 8 technical replicates. **c,** The first derivative of the emission ratio is shown and used to determine the melting point of each protein. Data is expressed as the mean of 8 technical replicates. **d,** Boxplots of the melting point observed across 8 replicates for the GFP-FUT10 and GFP-FUT11 proteins. The boxes represent the median/quartiles and whiskers represent 1.5X the interquartile range.

Replicate 1

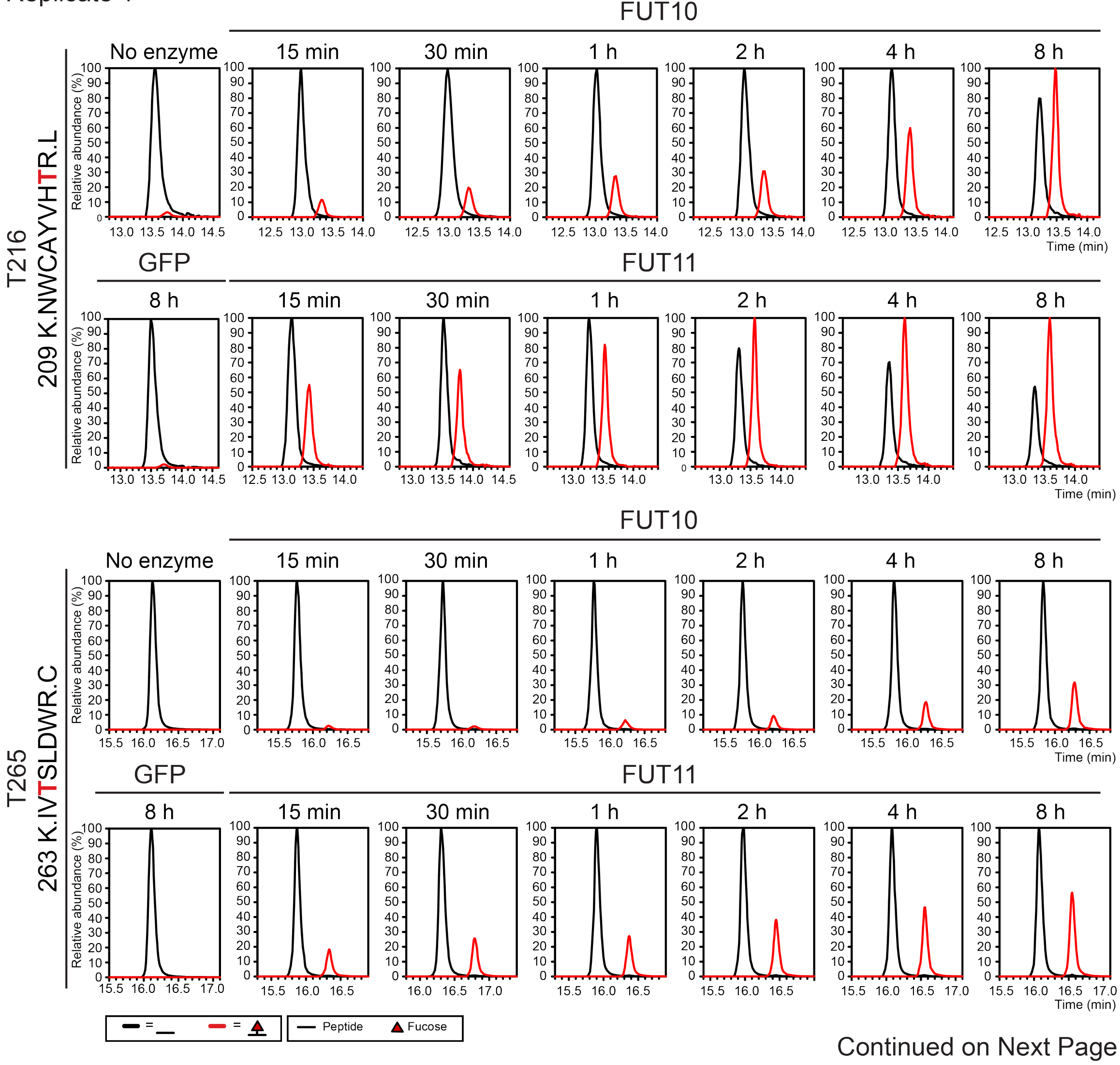

Continued on Next Page

Replicate 2

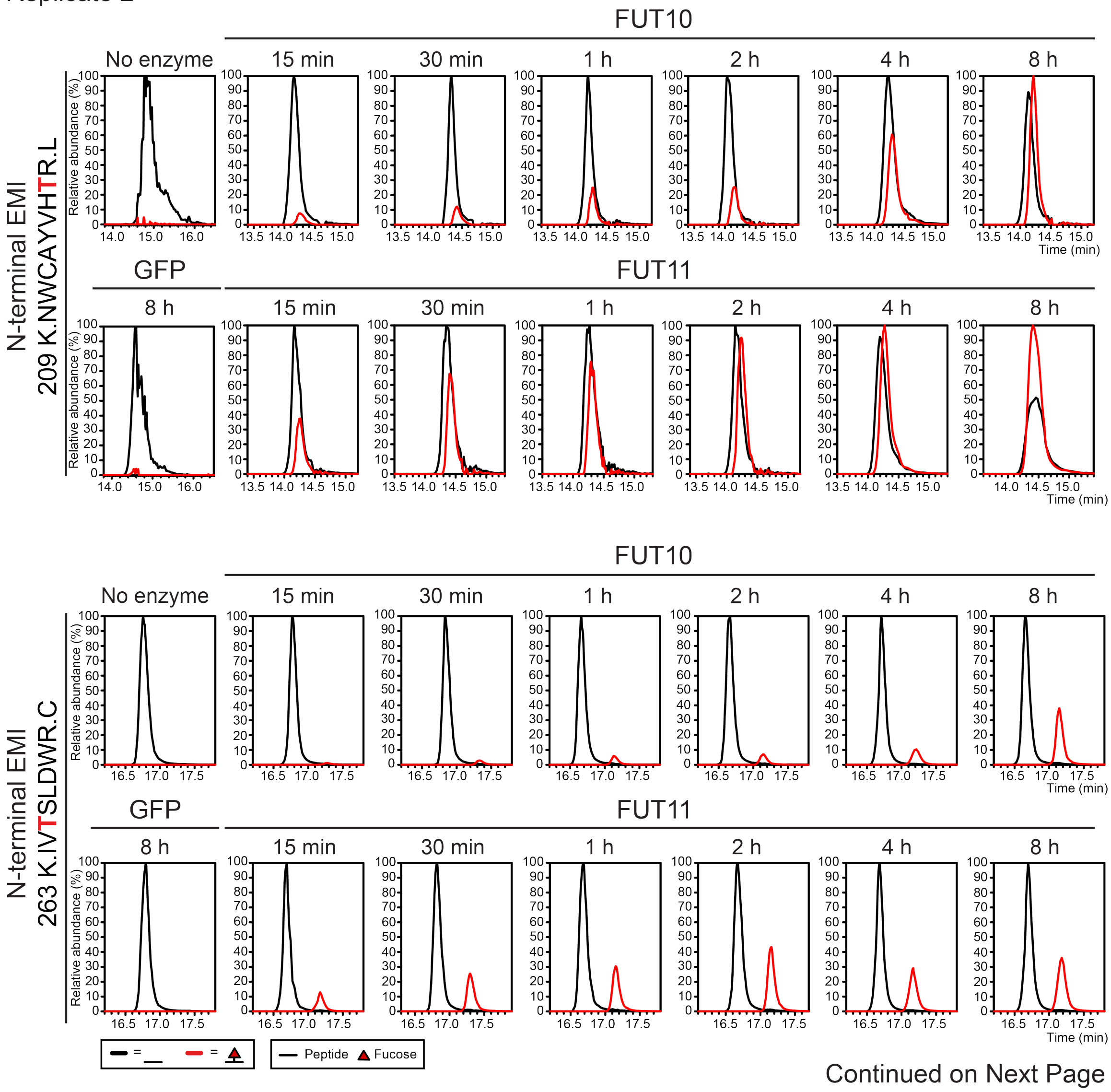

Continued on Next Page

Replicate 3

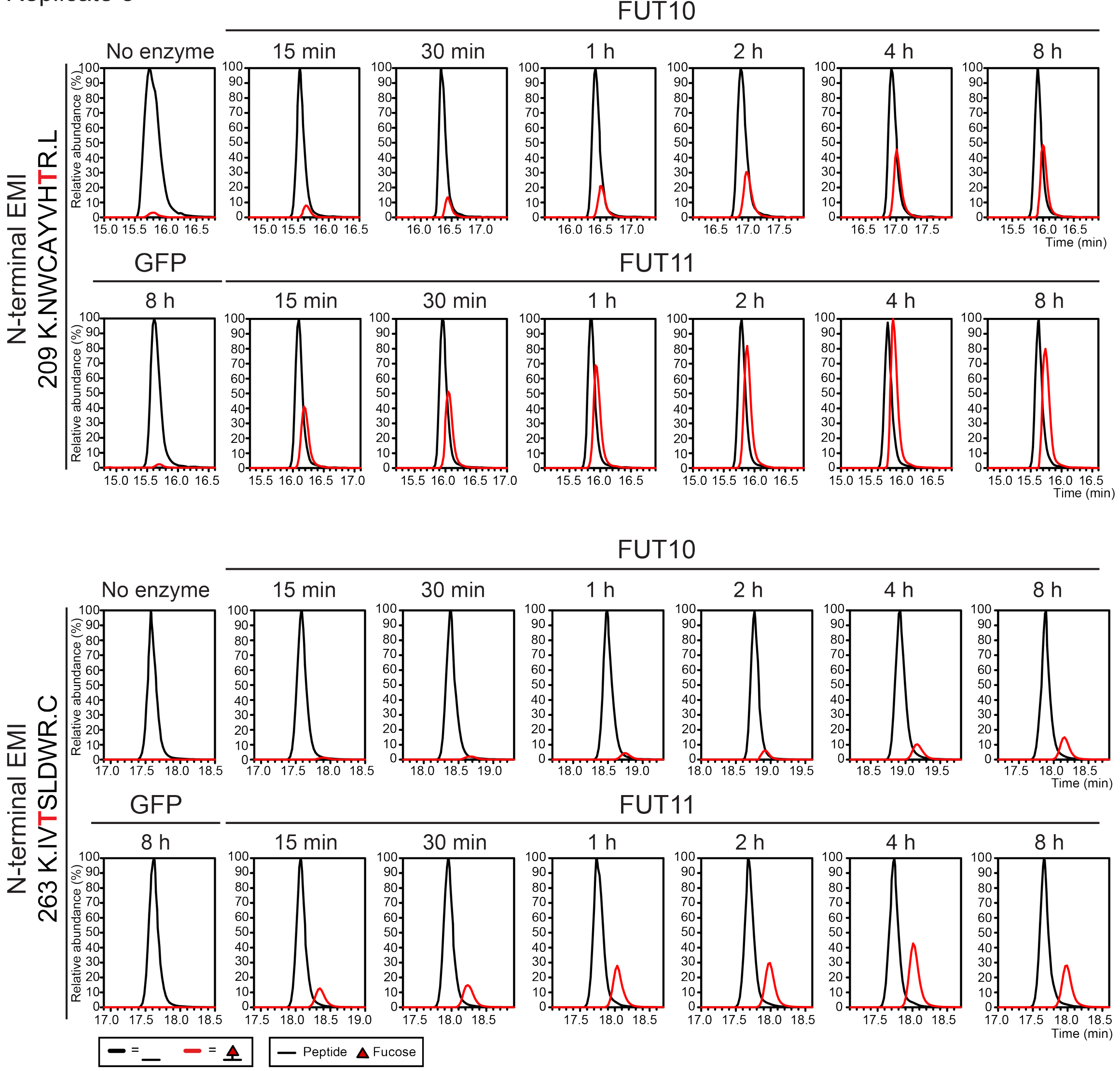

Supplementary Fig. 8. Biological triplicates for the data presented in Fig. 4a. Enzymatic assays using 0.5 μM non-fucosylated N-terminal EMI, 100 μM of GDP-fucose, and 0.1 μM of purified GFP-FUT10, GFP-FUT11, or GFP (negative control). Reaction products were analyzed with nano-LC-MS/MS and EICs of peptides containing the T216 or T265 *O*-fucose site from N-terminal EMI were generated as described in Materials and Methods. Biological triplicates of data using three batches of purified enzymes included for the statistical analysis in Fig. 4a are presented.

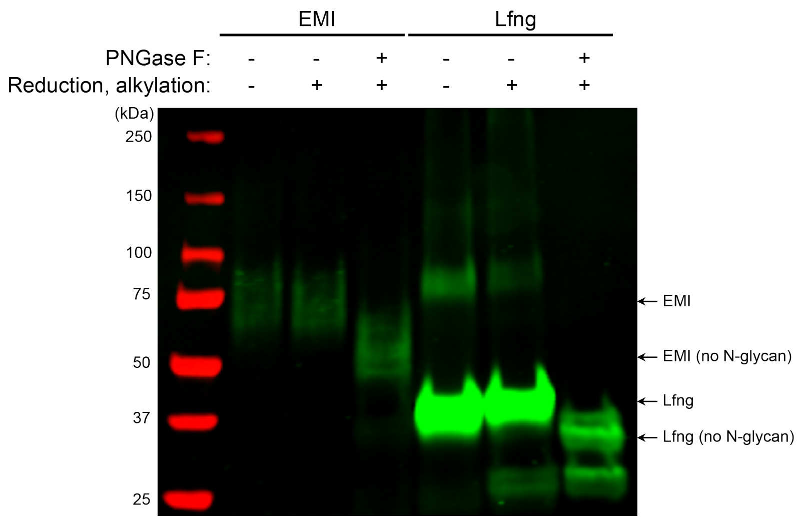

Supplementary Fig. 9. N-terminal EMI contains multiple *N*-glycans. N-terminal EMI and mouse Lfng (contains one *N*-glycan, used as a positive control) were expressed and purified from HEK293T cells. Proteins were reduced, alkylated, treated with PNGase F, and analyzed by Western blot probed with anti-Myc antibodies as described in Supplementary Materials and Methods (n=2).

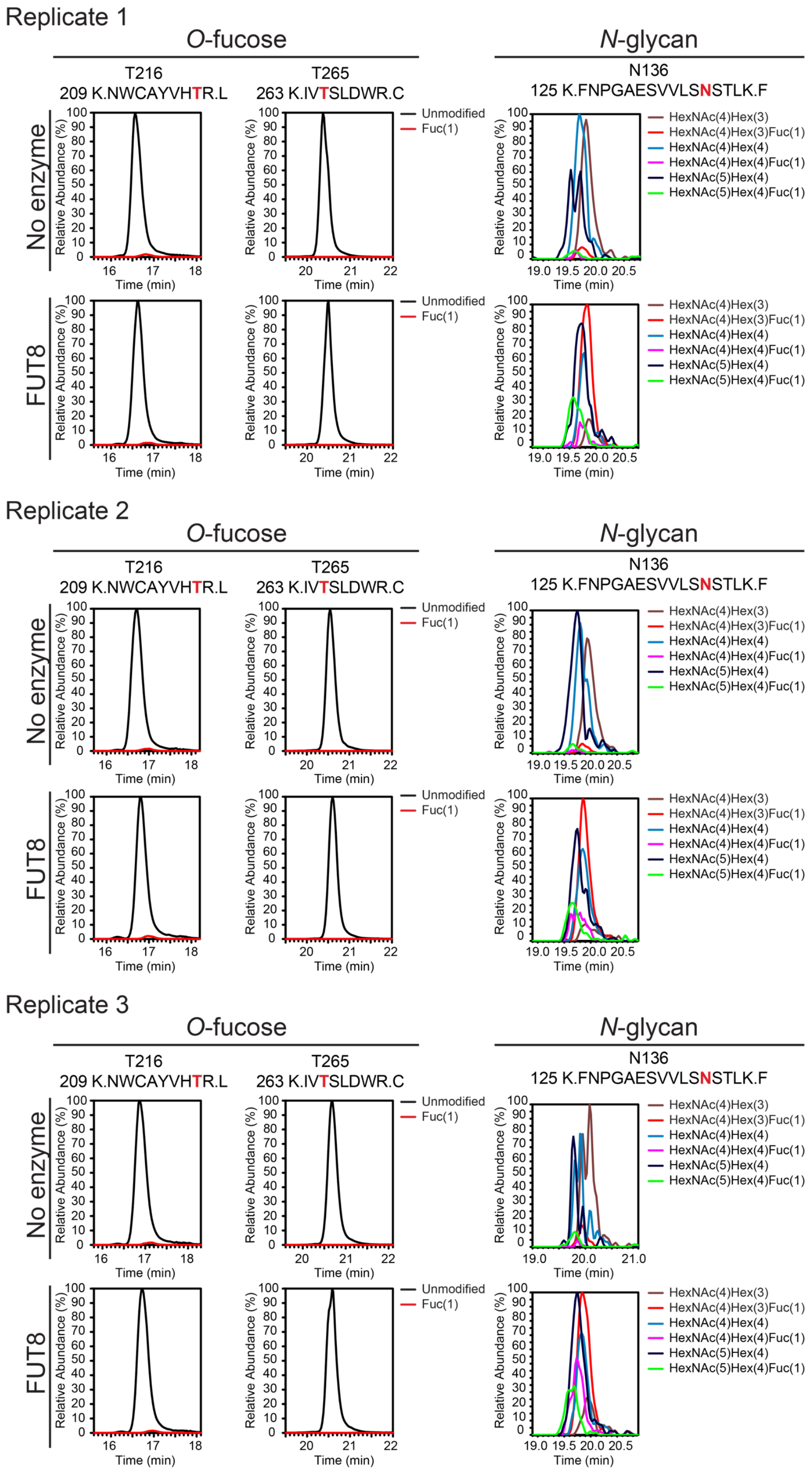

Supplementary Fig. 10. FUT8 adds fucose to *N*-glycans on N-terminal EMI. Enzymatic assays contained 0.5 μM non-fucosylated N-terminal EMI, 100 μM of GDP-fucose, and 0.1 μM of purified GFP-FUT8. Reactions were incubated at 37 °C for 30 minutes. Products were analyzed with nano-LC-MS/MS and EICs of peptides containing T216 or T265 *O*-fucose sites, or N136 *N*-glycan site from N-terminal EMI were generated as described in Materials and Methods. Biological triplicates of data using three batches of purified enzymes are presented.

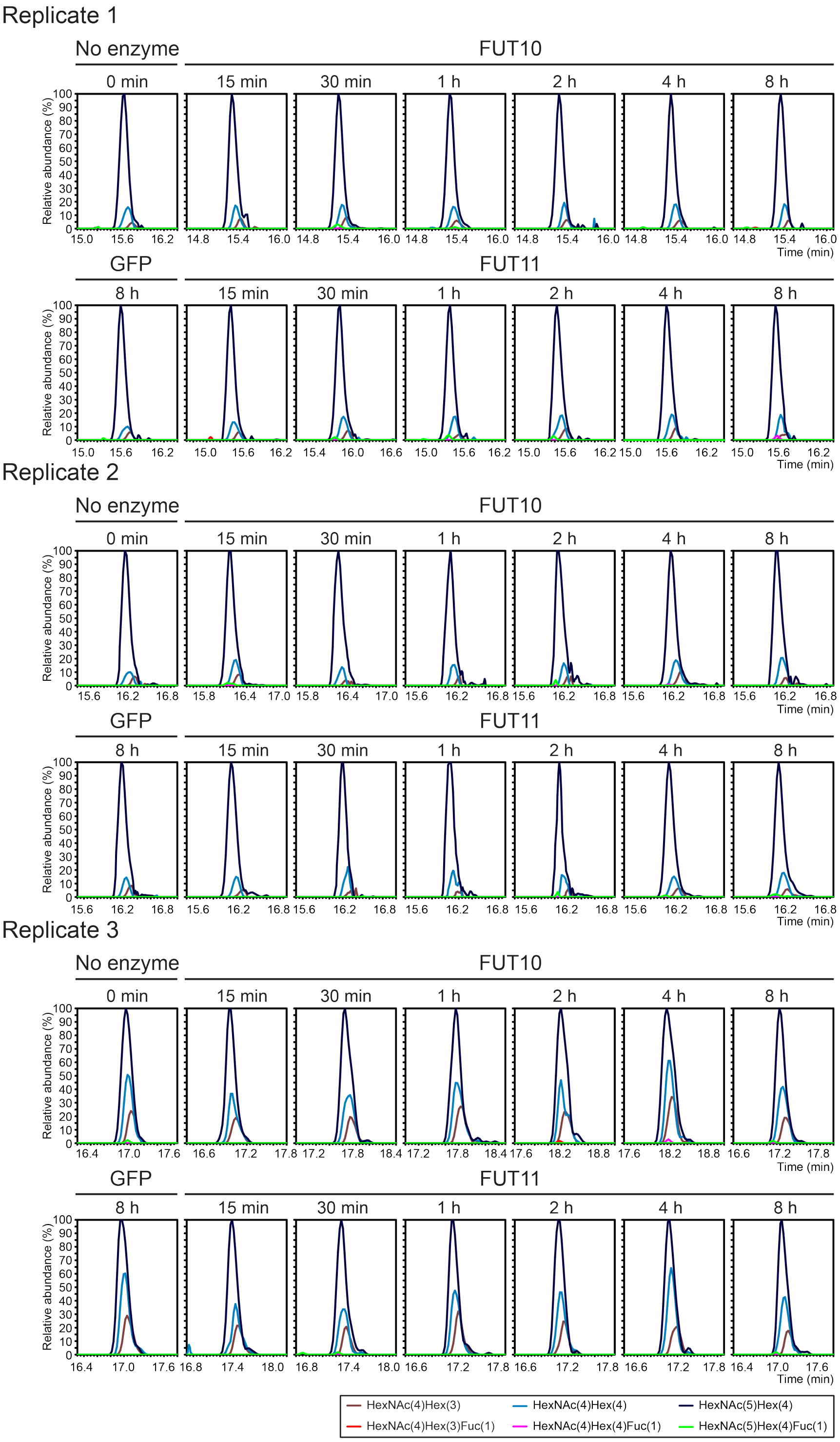

Supplementary Fig. 11. FUT10 and FUT11 do not modify *N*-glycans in enzymatic assays. Mass spectrometric analysis of peptide ^{125}FNPGAESVVLS<u>N</u>STLK^{140} containing a complex *N*-glycan site in N-terminal EMI that was produced in the enzymatic assays shown in Fig. 4a. EICs of *N*-glycosylated peptides with or without fucosylation were generated. Biological triplicates of data using three batches of purified enzymes are presented.

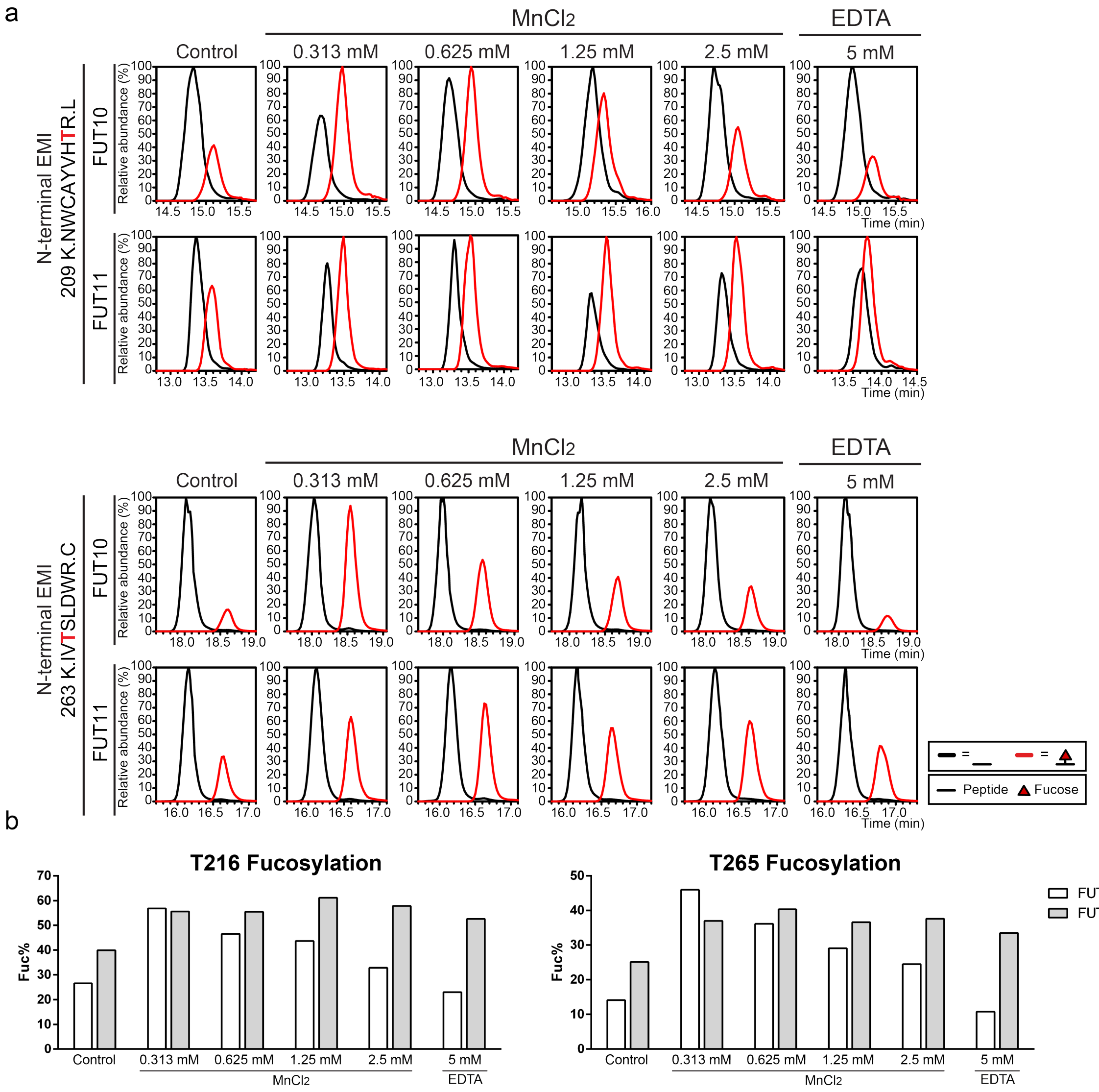

Continued on Next Page

c

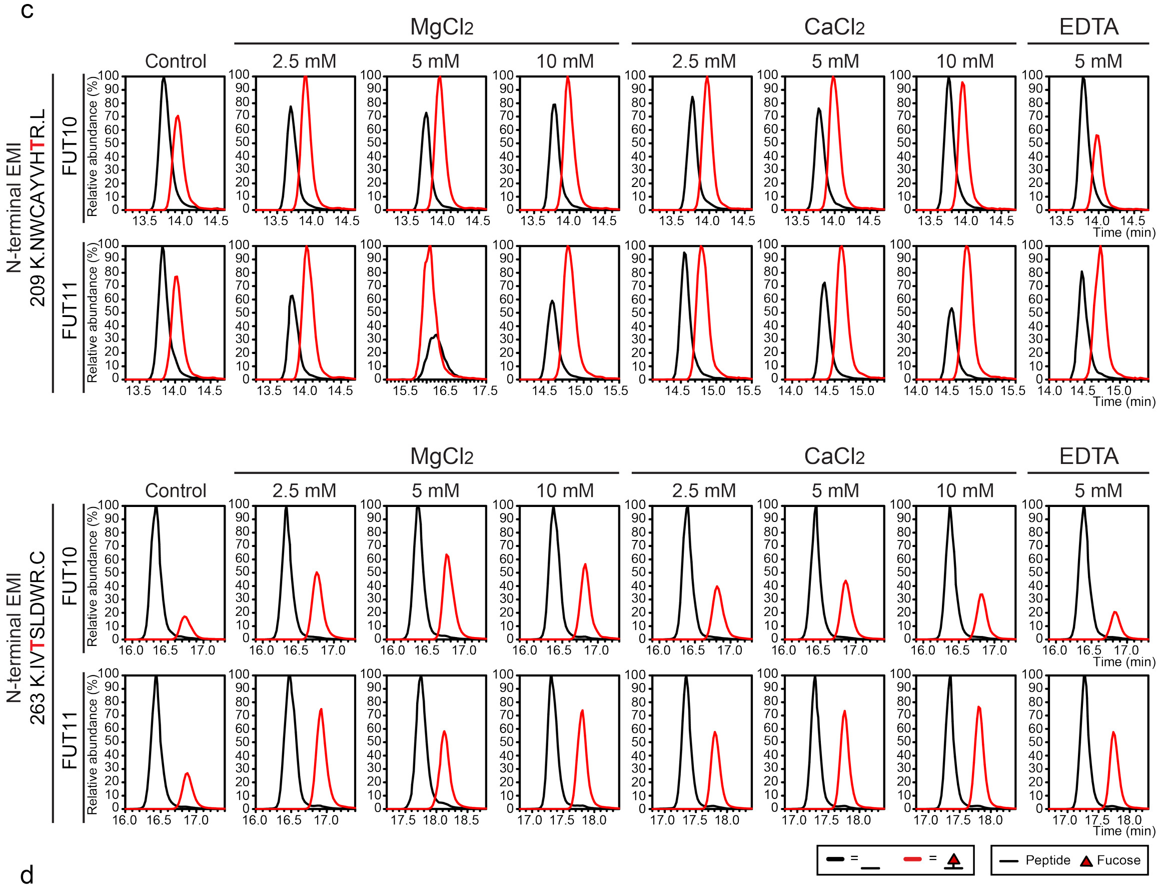

d

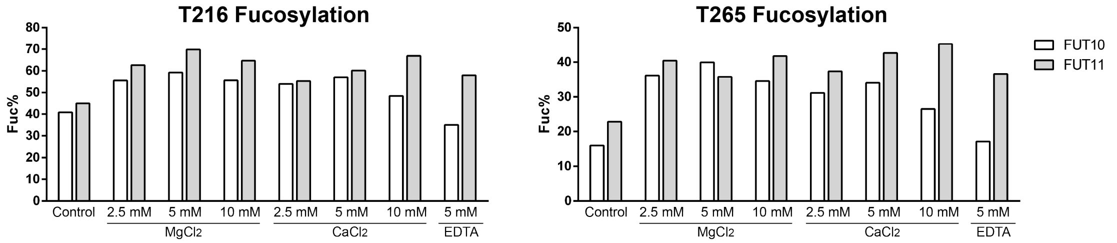

Supplementary Fig. 12. The activities of FUT10 and FUT11 were enhanced by divalent metal ions. Enzymatic assays of GFP-FUT10 and GFP-FUT11 with N-terminal EMI were supplied with varied concentrations of $MnCl_2$ (**a**), $MgCl_2$ (**c**), or $CaCl_2$ (**c**). Reaction products were analyzed with nano-LC-MS/MS. EICs of peptides containing the T216 or T265 *O*-fucose site from N-terminal EMI were generated and quantified in **b** and **d**. Experimental details can be found in Supplementary Materials and Methods.

a

Replicate 1

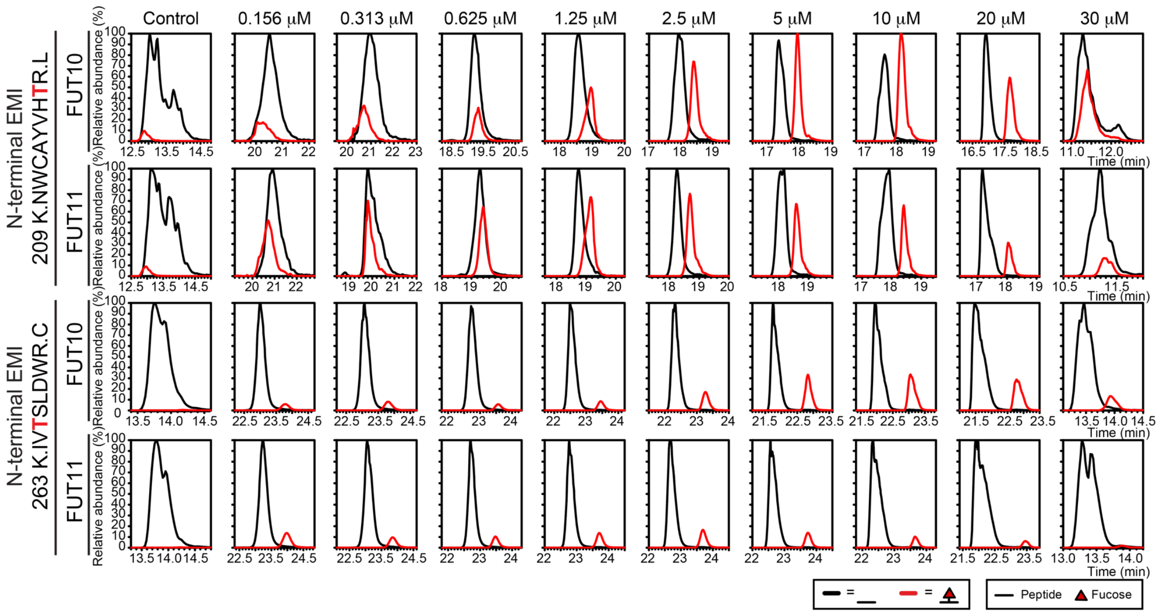

b

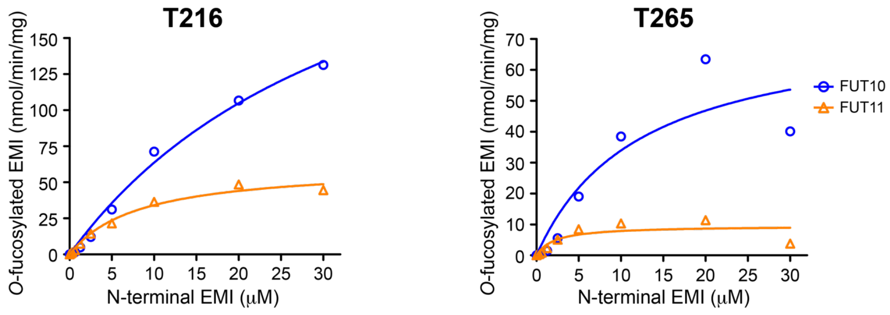

Continued on Next Page

c

Replicate 2

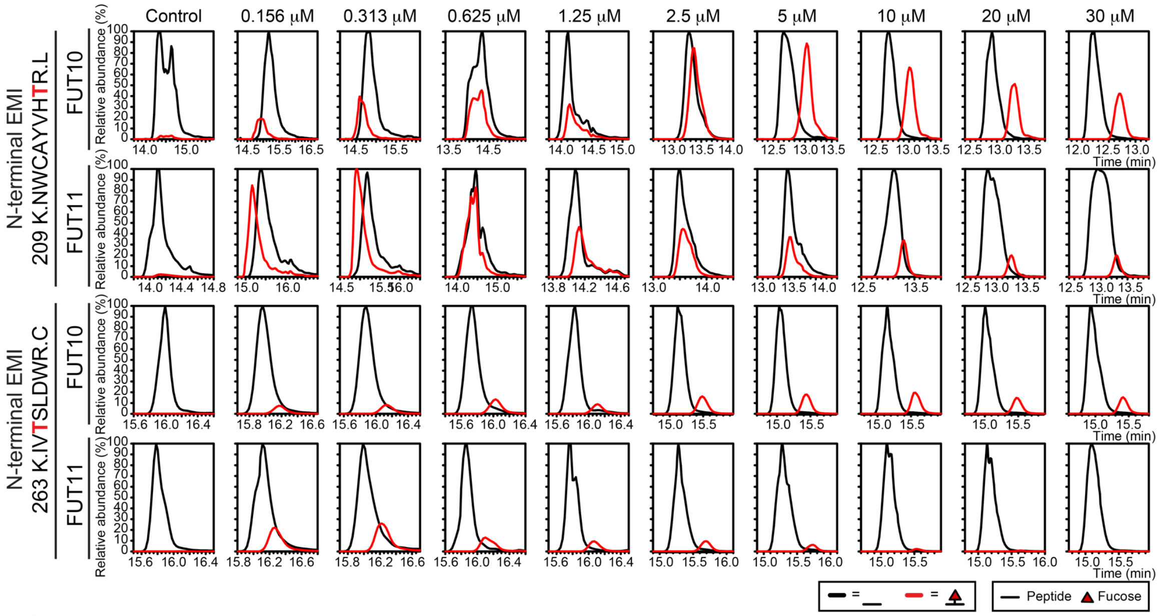

d

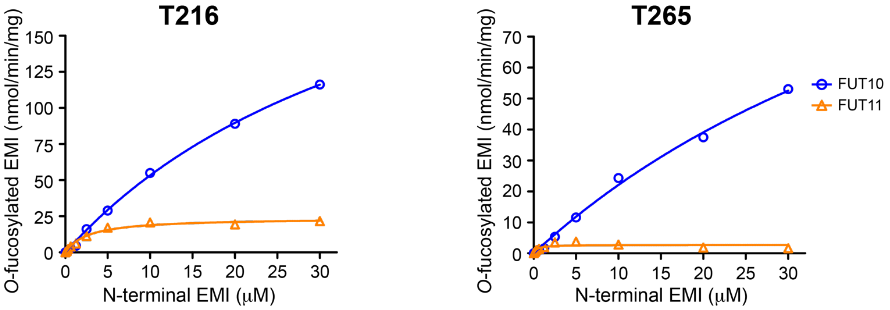

Continued on Next Page

e

Replicate 3

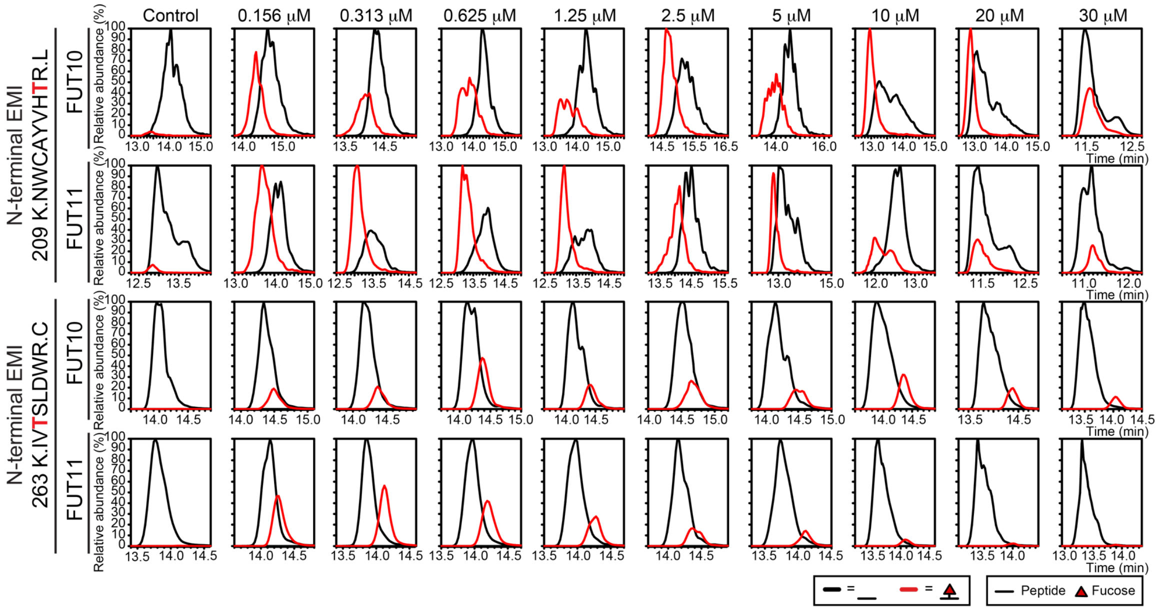

f

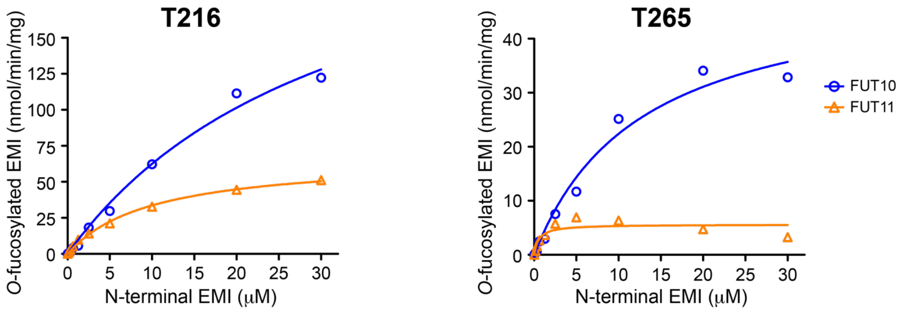

Supplementary Fig. 13. Biological triplicates for the data presented in Fig. 4b-d. Kinetic analysis of GFP-FUT10 and GFP-FUT11 with varied concentrations of N-terminal EMI substrates. Reaction products were analyzed using a mass spectrometric method as described in Materials and Methods. Biological triplicates of data included for the kinetics analysis in Fig. 4b-d are presented as EICs in **a**, **c**, and **e**, and plotted kinetic curves in **b**, **d**, and **f**.

a

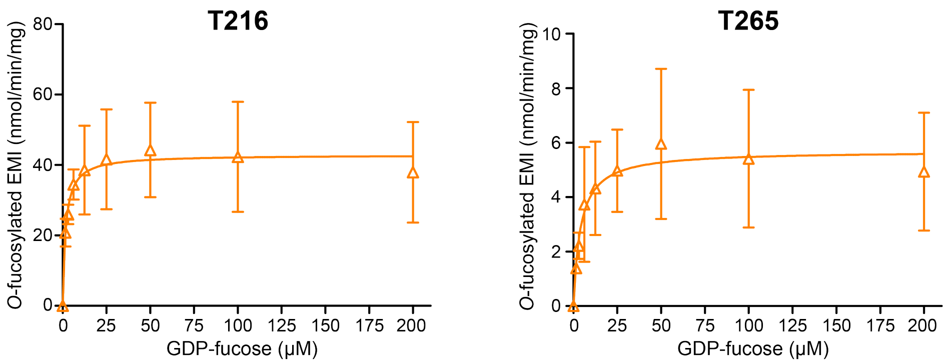

b

Kinetic parameter	FUT11	
	T216	T265
Vmax (nmol/min/mg)	42.86	5.695
Km (μM)	1.695	4.033

c

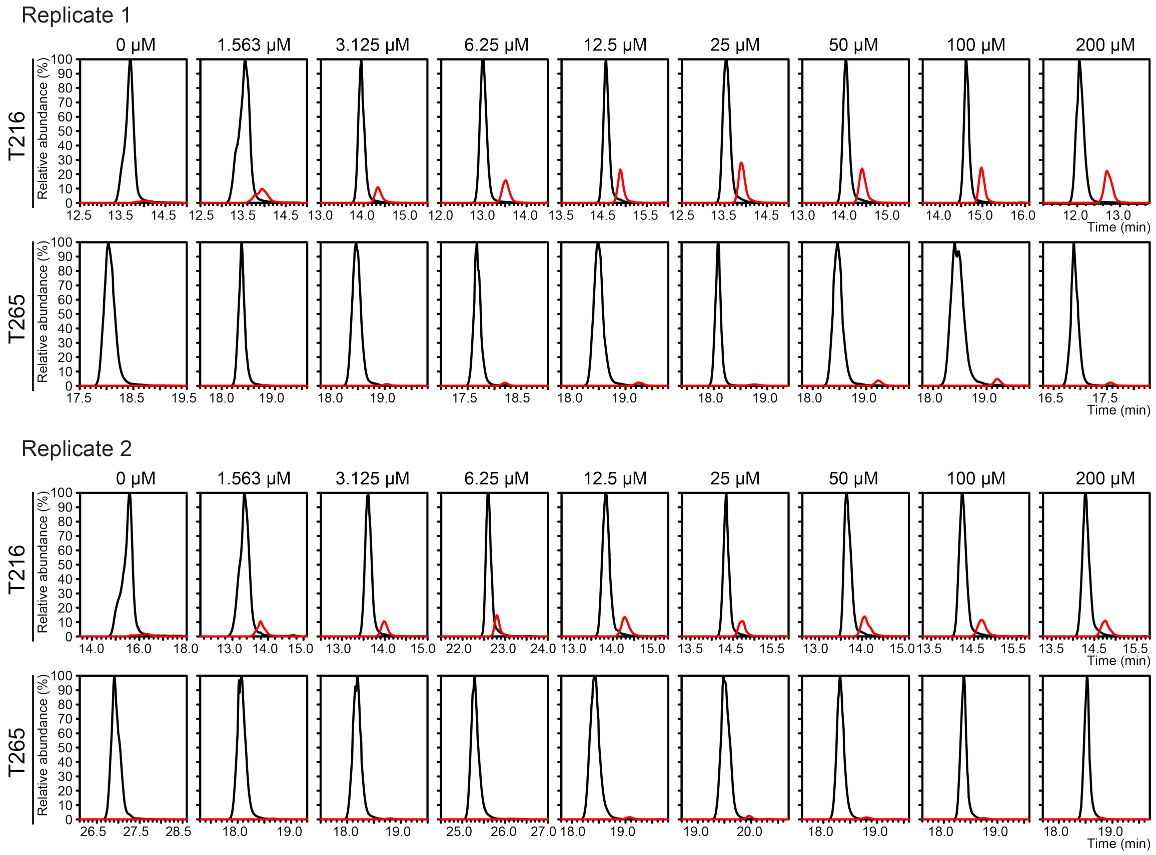

Continued on Next Page

Replicate 3

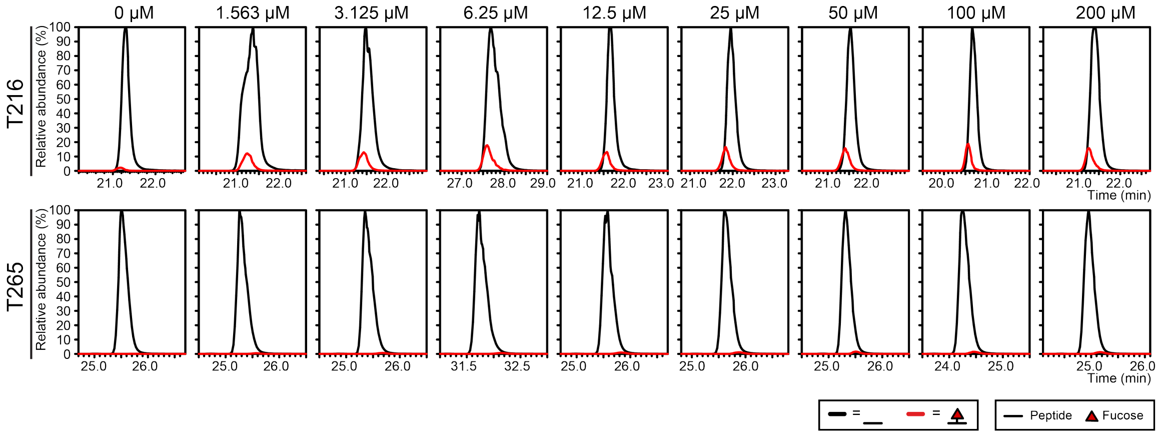

Supplementary Fig. 14. GDP-fucose concentration-dependent kinetics of FUT11. a, 50 nM of purified GFP-FUT11 was incubated with varied concentration of ultra-pure GDP-fucose, 20 μM of non-fucosylated N-terminal EMI, and 0.3 mM $MnCl_2$ for 15 min. Reaction products were reduced, alkylated, digested with trypsin, and analyzed by nano-LC-MS/MS. *O*-fucosylation stoichiometry on T216 site and T265 site was quantified from EICs and converted into product concentration as described in Materials and Methods. Kinetic curve was generated using nonlinear Michaelis-Menten fitting in Prism 7. Data presented as mean with +/-SD from biological triplicates using three batches of purified enzymes. **b,** Kinetic parameters calculated with nonlinear Michaelis-Menten fitting in Prism 7. **c,** Biological triplicates of data included for the kinetics analysis are presented.

a

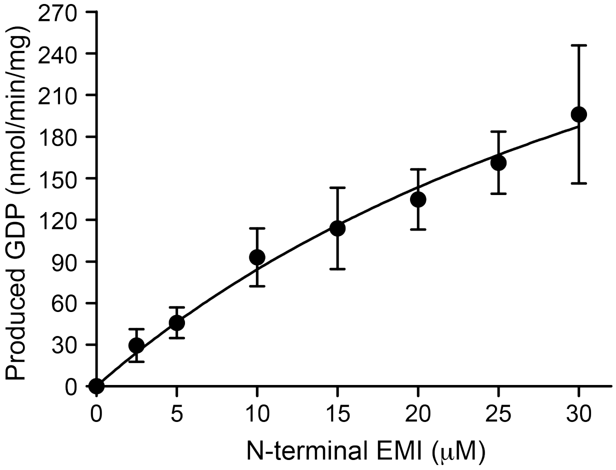

b

Substrate	FUT10	
	Km (μM)	Vmax (nmol/min/mg)
N-terminal EMI	47.22	482.00

Supplementary Fig. 15. EMI substrate concentration-dependent kinetics of FUT10 measured by GDP-Glo Glycosyltransferase assay. a, 50 nM of purified GFP-FUT10 was incubated with varied concentration of non-fucosylated N-terminal EMI, 100 μM of ultra-pure GDP-fucose and 0.3 mM $MnCl_2$ for 15 min. GDP-Glo Glycosyltransferase assays were performed to determine the kinetic parameters. The curve was generated with nonlinear Michaelis-Menten fitting in Prism 7. Data presented as mean with +/-SD from biological triplicates using three batches of purified enzymes. **b,** Kinetic parameters calculated with nonlinear Michaelis-Menten fitting in Prism 7. Experimental details can be found in Supplementary Materials and Methods.

a *FUT10* KO (Clone 11): 31-bp, 20-bp, 21-bp deletion in *FUT10*
(Clone 16): 1-bp insertion, 1-bp insertion & 4-bp deletion & 6-bp substitution,
1-bp insertion & 5-bp deletion, 6-bp deletion in *FUT10*

```
WT(gRNA2 and 3):   CATTATGCTCTGGTGGTCCCCGCTG   ACGGGGGAGAC...28bp...GCTTGTTTCTTCACCATC   AACCGGACCTACCTCCATCATCAC
FUT10-11 Allele 1: CATTATGCTCTGGT-----------   ----GGGAGAC...28bp...GCTTGTTTCTTCACCATC   A----------------TCATCAC
FUT10-11 Allele 2: CATT--------------------G   ACGGGGGAGAC...28bp...GCTTGTTTCTTCACCATC   AACCGGACCTACCTCCATCATCAC
FUT10-11 Allele 3: CATT--------------------G   ACGGGGGAGAC...28bp...GCTTGTTTCTTCACCATC   A-CCGGACCTACCTCCATCATCAC

..........................................................................................................

WT(gRNA2 and 3):   CATTATGCTCTGGTGGTCCCCGCTG   ACGGGGGAGAC...28bp...GCTTGTTTCTTCACCATC   AACCGGACCTACCTCCATCATCAC
FUT10-16 Allele 1: CATTATGCTCTGGTGGTCCCCGCTG(C)ACGGGGGAGAC...28bp...GCTTGTTTCTTCACCATC   AACCGGACCTACCTCCATCATCAC
FUT10-16 Allele 2: CATTATGCTCTGGTGGTCCCCGCTG(C)ACGGGGGAGAC...28bp...GCTTGTTTCACCATC---   -ACCGGACCTACCTCCATCATCAC
FUT10-16 Allele 3: CATTATGCTCTGGTGGTCCCCGCTG   -----GGAGAC...28bp...GCTTGTTTCTTCACCATC(A)AACCGGACCTACCTCCATCATCAC
FUT10-16 Allele 4: CATTATGCTCTGGTGGTCCCCGCTG   -----GGAGAC...28bp...GCTTGTTTCTTCACCATC   -ACCGGACCTACCTCCATCATCAC
```

b *FUT11* KO (Clone 23): 363-bp deletion in *FUT11*,
(Clone 43): 122-bp deletion in *FUT11*

```
WT(gRNA2 and 3):   TGGTGTTGGTCCTTCTAGGGGTGCTCAGTGTCTGTGCAGCCAGCGGCCATGGGTCCGTAGCGGAGAGGG...265bp...GCTGCTCTT
FUT11-23 Allele 1: TGGTGTTGGTCCTTCTAGGGGTGCTCAGTGTCTGTGCAGCCAGCGGCC---------------------...265bp...---------

WT(gRNA2 and 3):   CTACGGCACAGACTTCCGCGCGTCGGCCGCCCCGCTGCCGCGCCTGGCGCACCAGAGCTGGGCGCTCCTCCACGAGGAGTCGC
FUT11-23 Allele 1: --------------------------------------------------------------------TCCACGAGGAGTCGC

..........................................................................................................

WT(gRNA2 and 3):   TGGTGTTGGTCCTTCTAGGGGTGCTCAGTGTCTGTGCAGCCAGCGGCCATGGGTCCGTAGCGGAGAGGG...265bp...GCTGCTCTT
FUT11-43 Allele 1: TGGTGTTGG------------------------------------------------------AGAGGG...265bp...GCTGCTCTT

WT(gRNA2 and 3):   CTACGGCACAGACTTCCGCGCGTCGGCCGCCCCGCTGCCGCGCCTGGCGCACCAGAGCTGGGCGCTCCTCCACGAGGAGTCGC
FUT11-43 Allele 1: CTACGGCAC--------------------------------------------------------------------AGTCGC
```

c *FUT10/11* DKO (Clone 17): 65-bp deletion in *FUT11*
(Clone 25): 212-bp deletion and 113-bp substitution in *FUT11*

```
WT(gRNA2 and 3):            TGGTCCTTCTAGGGGTGCTCAGTGTCTGTGCAGCCAGCGGCCATGGGTCCGTAGCGGAGA...201bp...AGTGTGCGCGCGGCGCGTGCG
FUT10/11 DKO-17 Allele 1:   TGGTCCTTCTAGGGGTGCTCAGTGTCTGTGCAGCCAGCGGC-----------AGCGGAGA...201bp...AGTGTGCGCGCGGCGCGTGCG

WT(gRNA2 and 3):            TGGCGTCCCGGAACCGCCGAGCGCTGAGGGACTCGCGGACGCGCGCGCTGCTCTTCTACGGCACAGACTTCCGCGCGTCGGCCGCCCC
FUT10/11 DKO-17 Allele 1:   TGGCGTCCCGGAACCGCCGAGCGCTGAGGGACTCGCGGACGCGCGCGCTGCTCTTCTACGGCACAGACTTCC----------------

WT(gRNA2 and 3):            CGCTGCCGCGCCTGGCGCACCAGAGCTGGGCGCTCCTCCACGAGGAGTCGCCCC
FUT10/11 DKO-17 Allele 1:   ---------------------------------------ACGAGGAGTCGCCCC

..........................................................................................................

WT(gRNA2 and 3):            TGGTCCTTCTAGGGGTGCTCAGTGTCTGTGCAGCCAGCGGCCATGGGTCCGTAGCGGAGA...201bp...AGTGTGCGCGCGGCGCGTGCG
FUT10/11 DKO-25 Allele 1:   TGGTCCTTCTAGGGGTGCTCAGTGTCTGTGCAGCCAGCGGCCATGGGTCCG---------...201bp...--CACACTATGAGCAACATGC

WT(gRNA2 and 3):            TGGCGTCCCGGAACCGCCGAGCGCTGAGGGACTCGCGGACGCGCGCGCTGCTCTTCTACGGCACAGACTTCCGCGCGTCGGCCGCCCC
FUT10/11 DKO-25 Allele 1:   AGATCTACAGCGCCAGAGTGCTGACGCCTTACTGGCAATGCTTCCACAGCGTAAATACACCCACGTACTGGACGCGGGTTGTGGACCT

WT(gRNA2 and 3):            CGCTGCCGCGCCTGGCGCACCAGAGCTGGGCGCTCCTCCACGAGGAGTCGCCCC
FUT10/11 DKO-25 Allele 1:   TGCTGGTGCGCCTGGCGCACCAGAGCTGGGCGCTCCTCCACGAGGAGTCGCCCC
```

Underlined: guide RNA   Red: Edited genomic sequence   -: Deletion   (NNN): Insertion

Supplementary Fig. 16. Generation of *FUT10* KO, *FUT11* KO, *FUT10/11* DKO HEK293T cells using CRISPR/Cas9. Genomic sequences of *FUT10*, and *FUT11* amplified from WT and *KO* HEK293T cells. Primers to amplify regions surrounding gRNA sequences (underlined) are in Table S2. No WT sequences were detected in any of the KO cell lines. **a,** Three altered *FUT10* alleles were detected in *FUT10* KO clone 11 and four altered *FUT10* alleles were detected in *FUT10* KO clone 16. **b,** One altered *FUT11* allele was detected in *FUT11* KO clone 23 and clone 43. **c,** *FUT10/11* DKO clone 17 and clone 25 were generated from *FUT10* KO clone 11 and clone 16, respectively. Genomic sequence of *FUT11* were amplified from *FUT10/11* DKO cells. One altered *FUT11* allele was detected in both *FUT10/11* DKO clones.

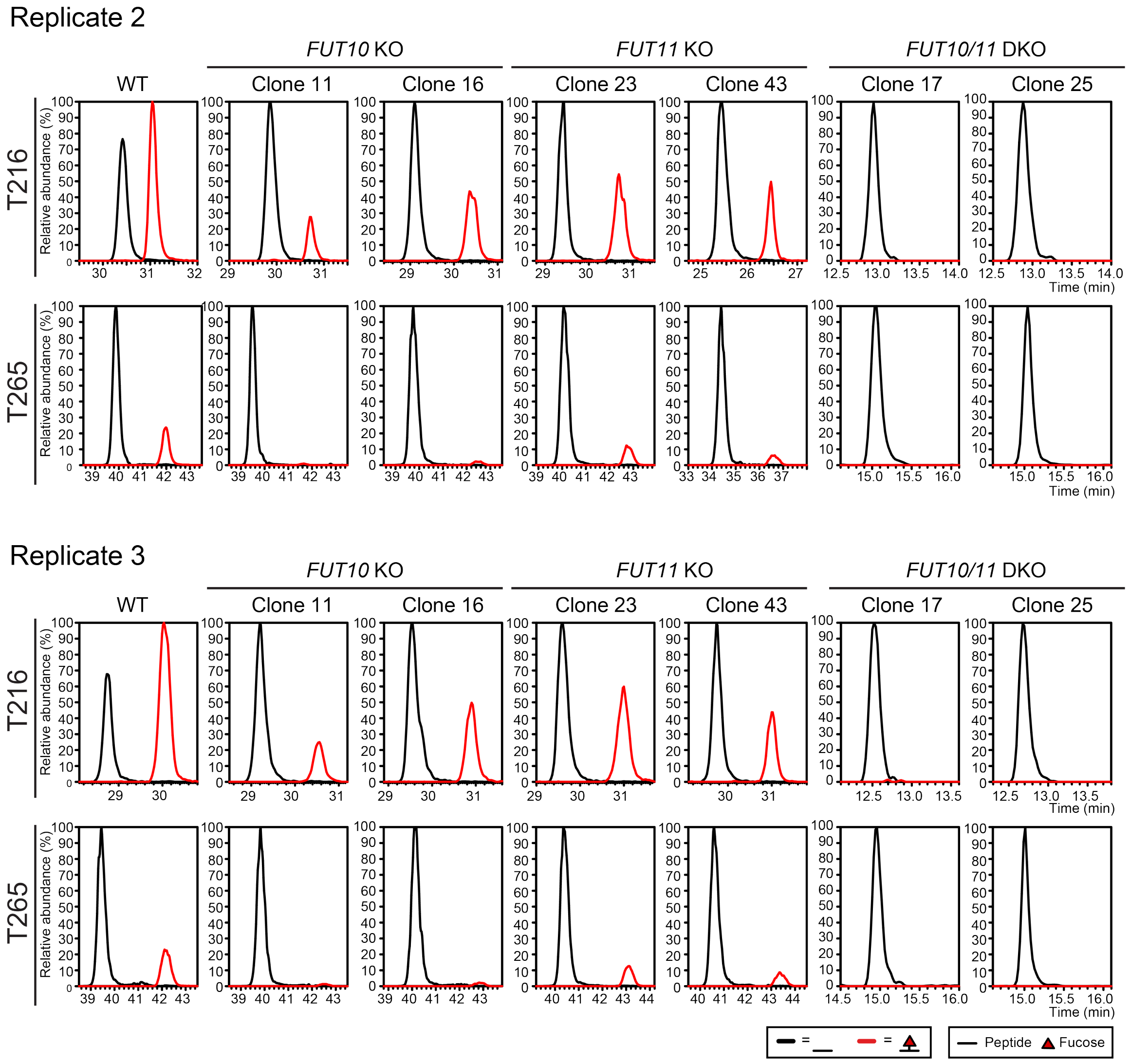

Supplementary Fig. 17. Biological triplicates for the data presented in Fig. 5a,b. N-terminal EMI was expressed in WT, *FUT10* KO, *FUT11* KO, or *FUT10/11* DKO HEK293T cells. 300 μL of conditioned culture medium were analyzed with nano-LC-MS/MS as described in Materials and Methods. EICs of peptides containing the T216 or T265 *O*-fucose site from N-terminal EMI were generated. In addition to the one replicate used in Fig. 5a, two additional biological replicates of data included for the statistical analysis in Fig. 5b are presented.

Replicate 2

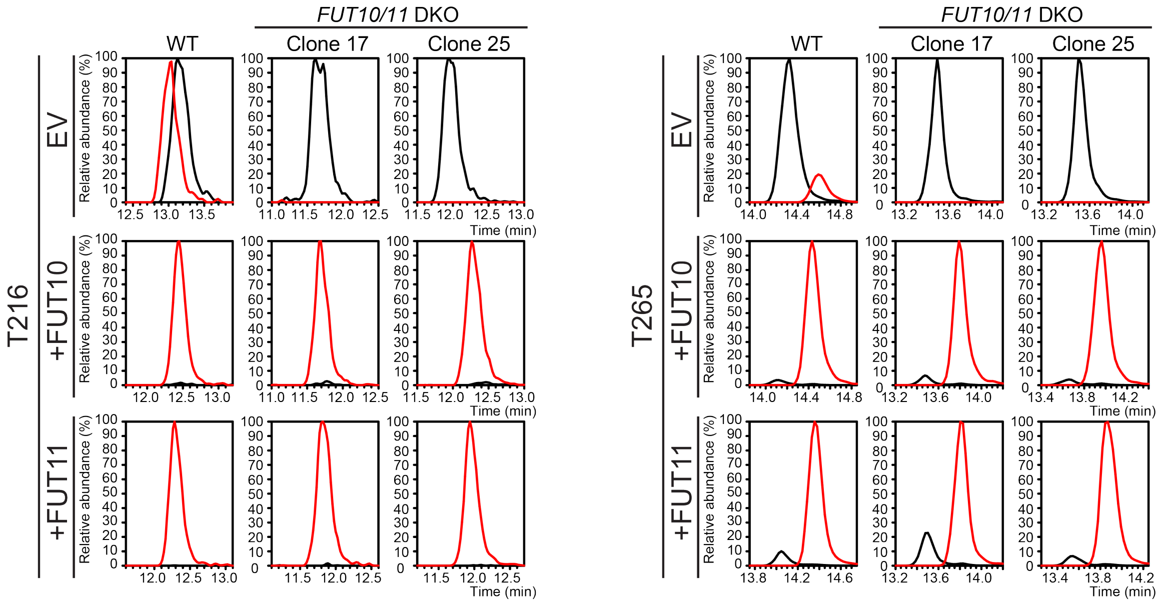

Replicate 3

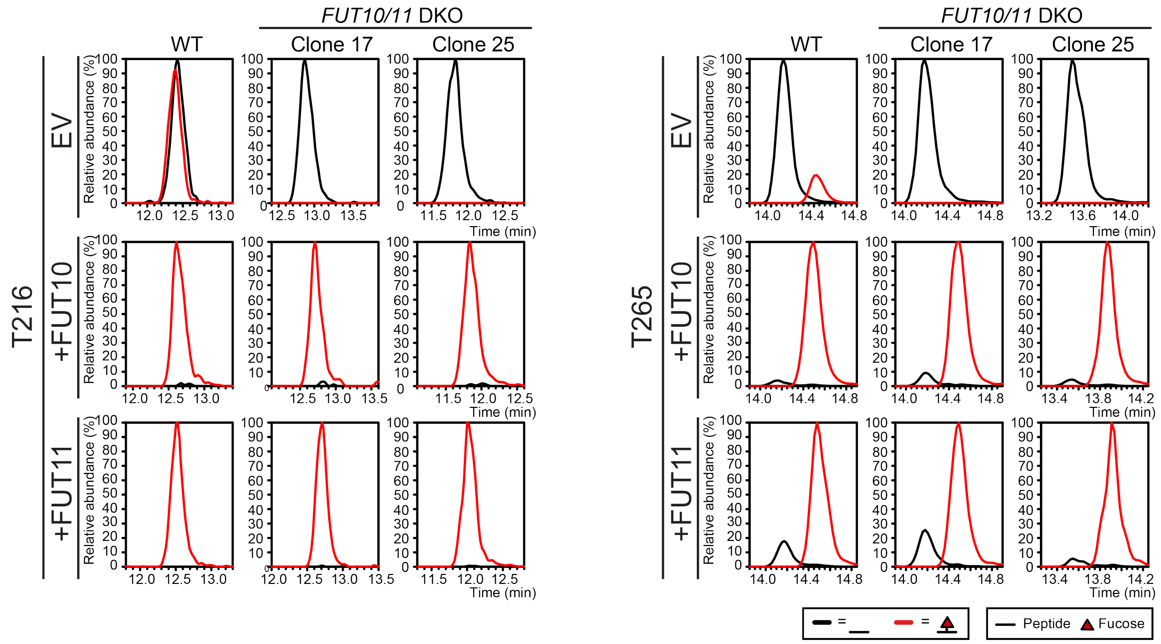

Supplementary Fig. 18. Biological triplicates for the data presented in Fig. 5c,d. N-terminal EMI was transfected in WT or *FUT10/11* DKO HEK293T cells co-transfected with plasmids encoding full-length FUT10, FUT11 or empty vector (EV). 300 μL of conditioned culture medium were analyzed with nano-LC-MS/MS as described in Materials and Methods. EICs of peptides containing the T216 or T265 *O*-fucose site from N-terminal EMI were generated. In addition to the one replicate used in Fig. 5c, two additional biological replicates of data included for the statistical analysis in Fig. 5d are presented.

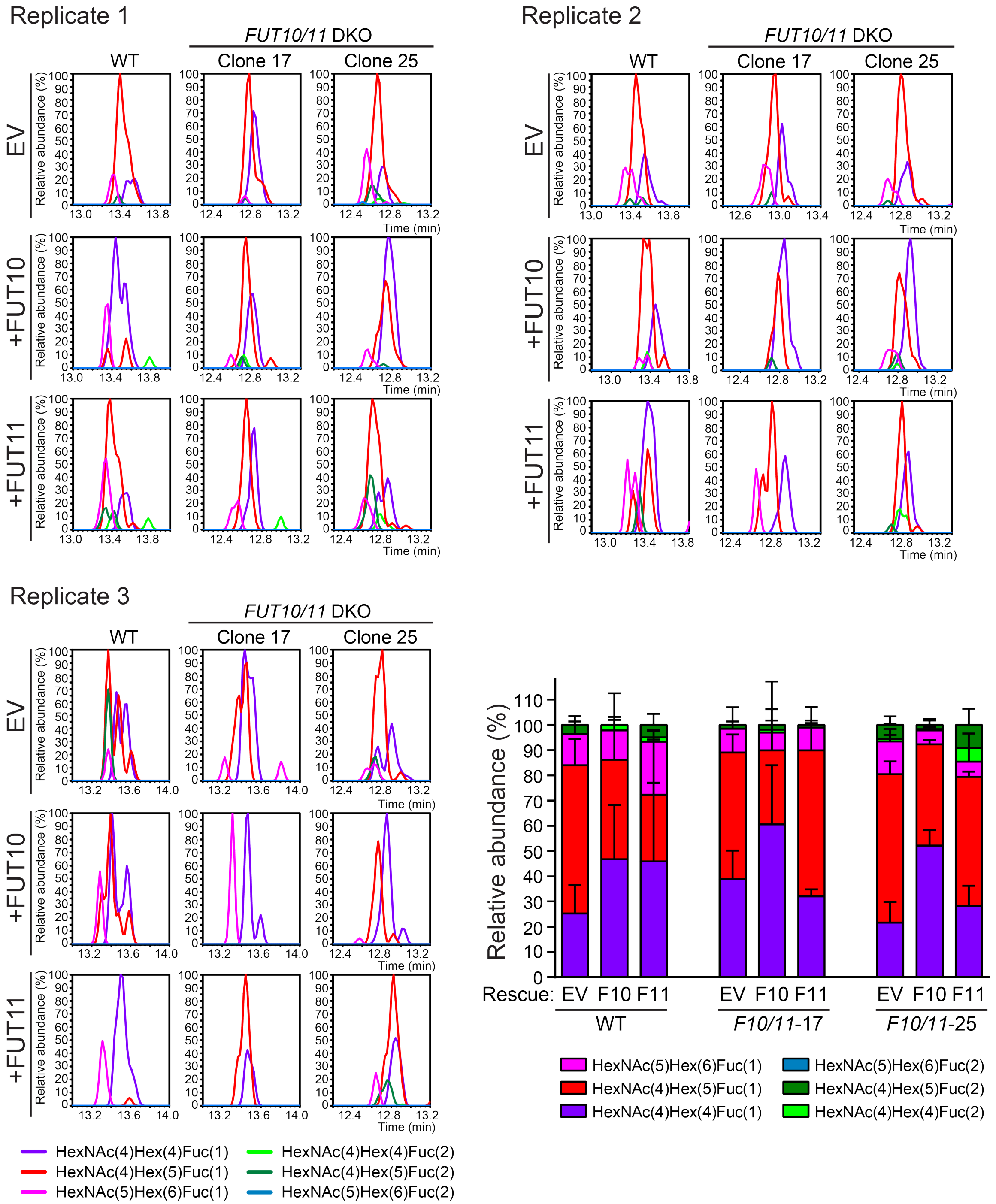

Supplementary Fig. 19. FUT10 and FUT11 do not modify *N*-glycans in cells. Mass spectrometric analysis for *N*-glycan fucosylation using the data obtained in Fig. 5c,d, and Fig. S18. EICs of different glycoforms of peptides ^{125}FNPGAESVVLSNSTLK^{140} modified with fucosylated *N*-glycans were generated. Quantified relative abundances of different *N*-glycan glycoforms are presented as bar graph in the bottom right panel. Data is shown as mean with +/-SD from biological triplicates. *F10/11*-17, *FUT10/11* DKO cells-clone 17; *F10/11*-25, *FUT10/11* DKO cells-clone 25; EV, empty vector; F10, FUT10 plasmid; F11, FUT11 plasmid.

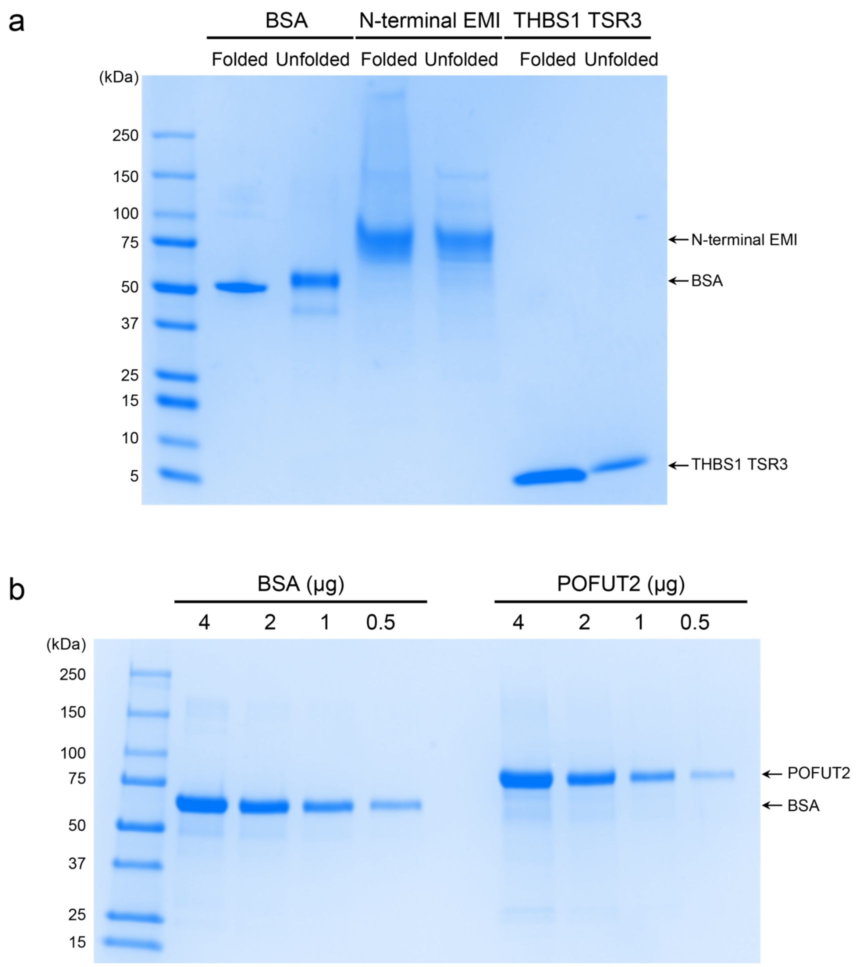

Supplementary Fig. 20. Folded and unfolded substrates (N-terminal EMI and THBS1 TSR3) and purified POFUT2 used for EMI unfolding assays presented in Fig. 6a. a, Non-fucosylated N-terminal EMI and bacterial produced human THBS1 TSR3 were unfolded as described in in Materials and Methods. 5 µg of protein was mixed with non-reducing sample buffer (4% SDS, 20% glycerol in 100 mM Tris/HCl, pH 6.8) and loaded for Coomassie blue staining (n=3). Folded BSA standard: 2 µg BSA mixed with non-reducing sample buffer; unfolded BSA standard: 2 µg BSA mixed with reducing sample buffer (4% SDS, 200 mM 2-mercaptoethanol, 20% glycerol in 100 mM Tris/HCl, pH 6.8) and boiled at 105°C for 5 min. **b,** Purity of the recombinant and purified POFUT2 was verified by Coomassie blue staining (n=2).

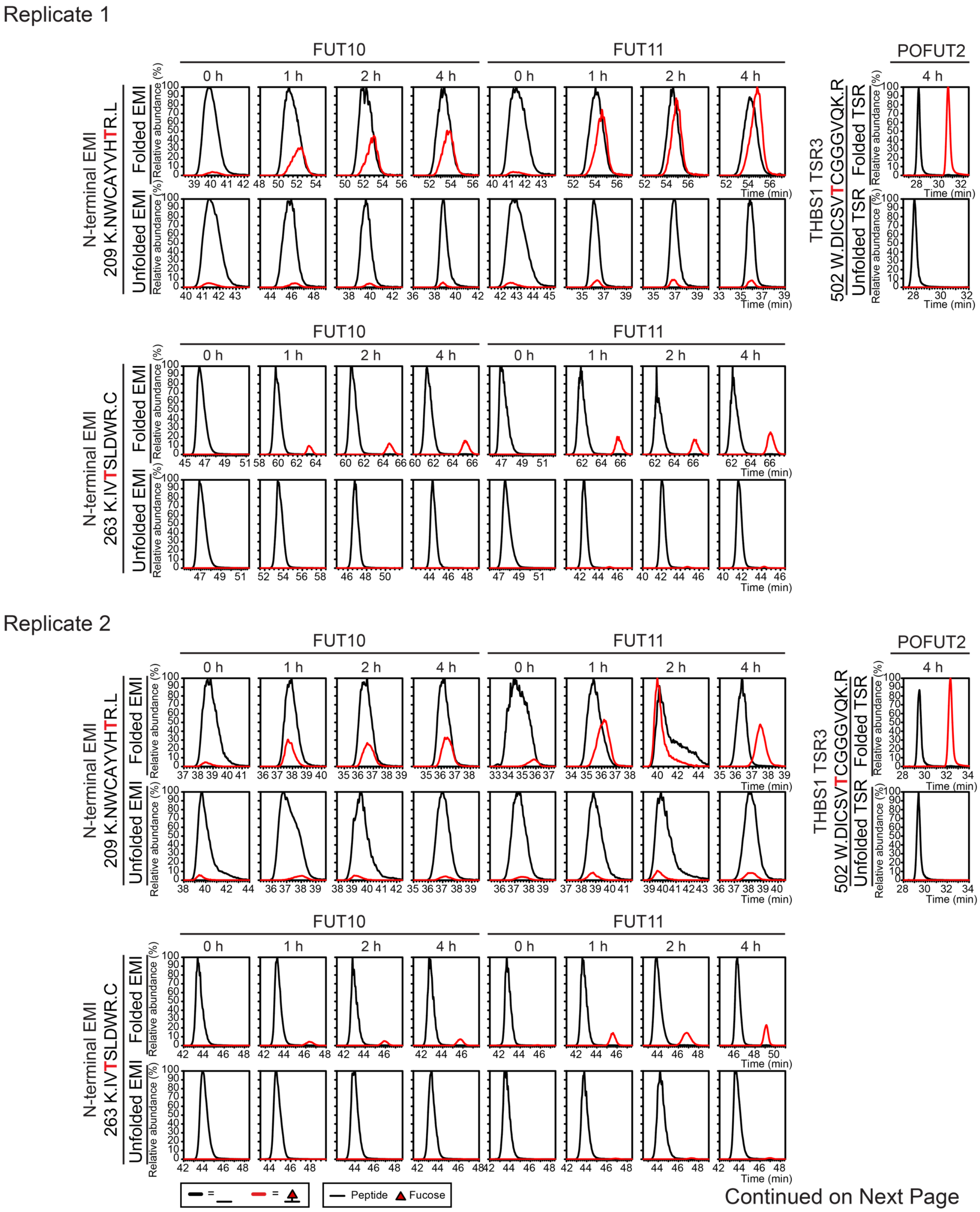

Continued on Next Page

Replicate 3

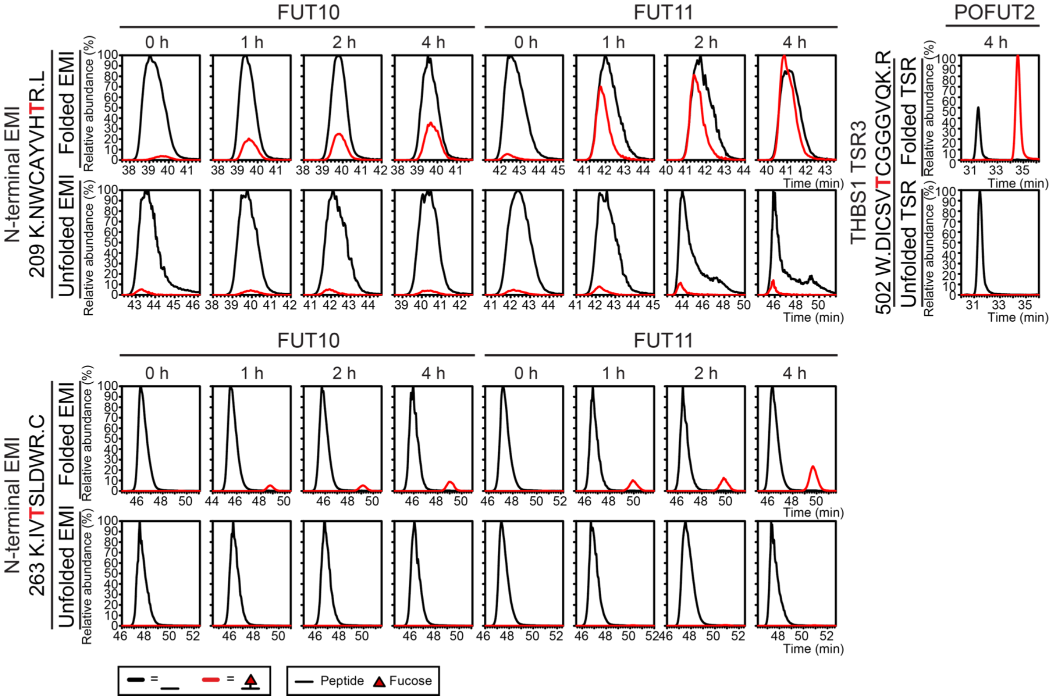

Supplementary Fig. 21. Biological triplicates for the data presented in Fig. 6a. Enzymatic assays of GFP-FUT10 and GFP-FUT11 with folded or unfolded N-terminal EMI substrates. POFUT2 with folded or unfolded THBS1 TSR3 was used as a positive control. Reaction products were analyzed with nano-LC-MS/MS and EICs of peptides containing the T216 or T265 *O*-fucose site from N-terminal EMI were generated as described in Materials and Methods. Biological triplicates of data included for the curve plotted in Fig. 6a are presented.

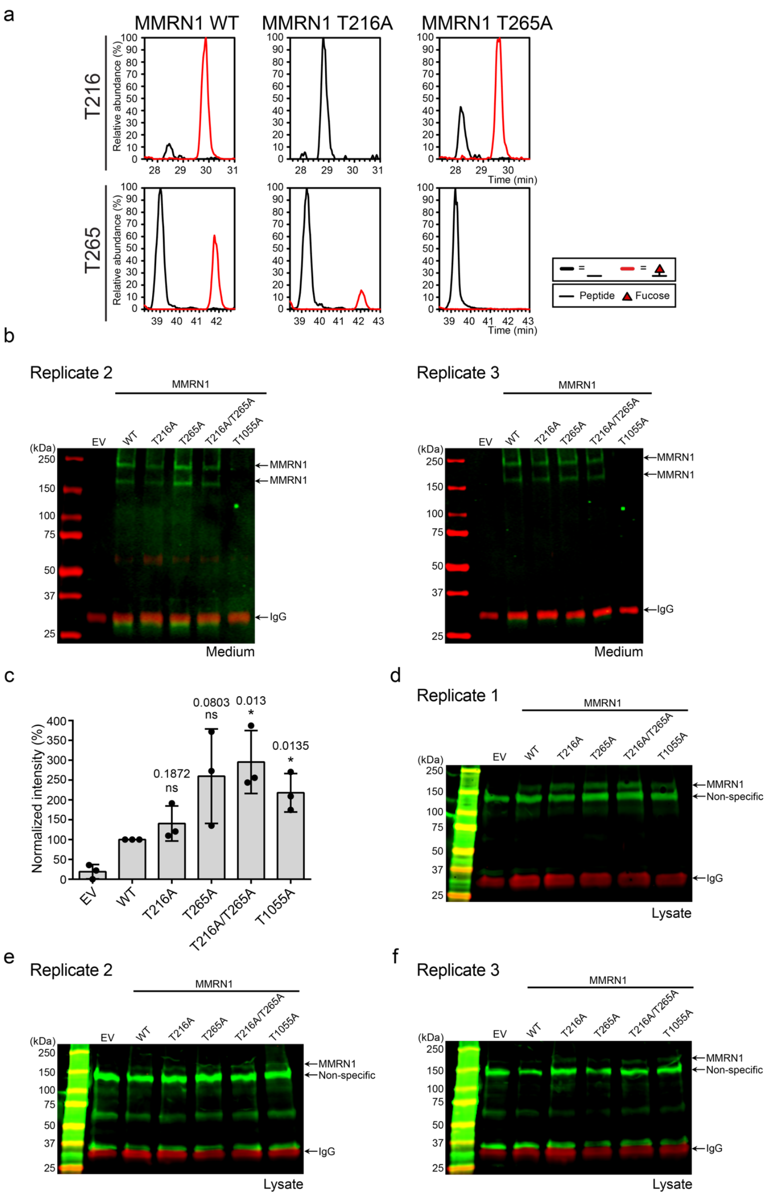

Supplementary Fig. 22. Comparing MMRN1 WT with MMRN1 T216A, MMRN1 T265A, MMRN1 T216A/T265A, and MMRN1 T1055A mutants. a, Full-length MMRN1 WT, T216A mutant, and T265A mutant were expressed and purified from HEK293T cells. The *O*-fucosylation status on the T216 and T265 sites on EMI domains was analyzed by nano-LC-MS/MS as described in Materials and Methods. **b,** HEK293T cells were transfected with plasmids encoding MMRN1 WT, MMRN1 T216A, MMRN1 T265A, MMRN1 T216A/T265A, MMRN1 T1055A, or empty vector (EV) and IgG (secretion control) for 48 h. Medium were collected and analyzed by Western blot probed with anti-Myc and anti-human IgG antibodies. In addition to the one replicate used in Fig. 6c, two additional biological replicates of data included for the statistical analysis in Fig. 6f are presented. **c-f,** HEK293T cells were transfected with plasmids encoding MMRN1 WT, different MMRN1 mutants, or empty vector (EV) and IgG for 24 h. Cell lysates were collected and analyzed by Western blot probed with anti-Myc, anti-His and anti-human IgG antibodies. Biological triplicates of blots are presented in panel **d**, **e** and **f**. Bar graph of quantified band intensity of MMRN1 normalized with IgG is presented in panel **c**. Data is shown as mean with +/-SD from biological triplicates of three individual transfections. Statistical analysis is performed with unpaired, two-tailed t test in Prism 7. ns, $p > 0.05$; *, $p < 0.1$ compared with control (WT cells).

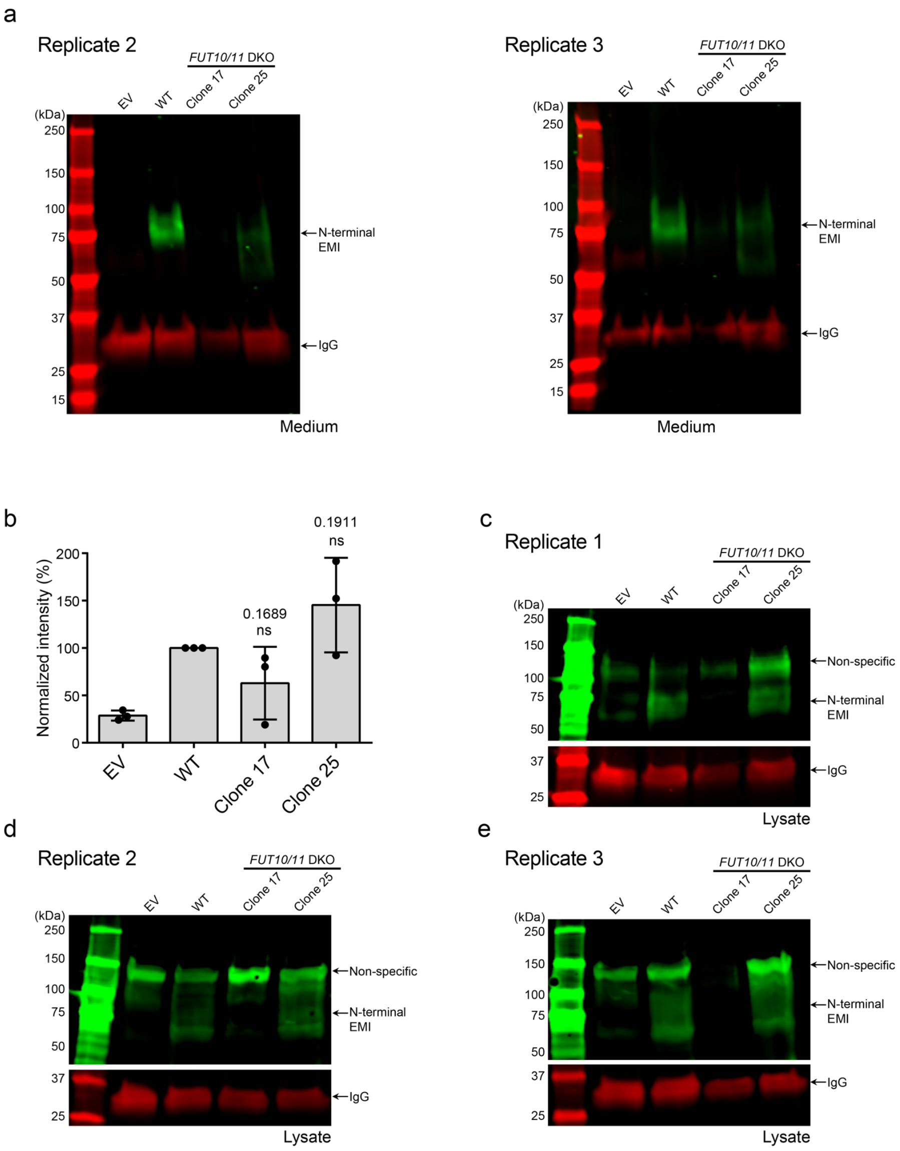

Supplementary Fig. 23. Secretion of N-terminal EMI in HEK293T WT and *FUT10/11* DKO cells. HEK293T WT or *FUT10/11* DKO cells were transfected with plasmids encoding Myc-tagged N-terminal EMI or empty vector (EV) and IgG (secretion control) for 48 h. Culture medium and cell lysates were collected and analyzed by Western blot. **a,** In addition to the one replicate used in Fig. 6d, two additional biological replicates of data included for the statistical analysis in Fig. 6g are presented. **b-e,** Cell lysates analyzed by Western blot probed with anti-Myc, anti-His and anti-human IgG antibodies. Biological triplicates of blots are presented in panel **c**, **d** and **e**. Bar graph of quantified band intensity of N-terminal EMI normalized with IgG is presented in panel **b**. Data is shown as mean with +/-SD from biological triplicates of three individual transfections. Statistical analysis is performed with unpaired, two-tailed t test in Prism 7. ns, $p > 0.05$ compared with control (WT cells).

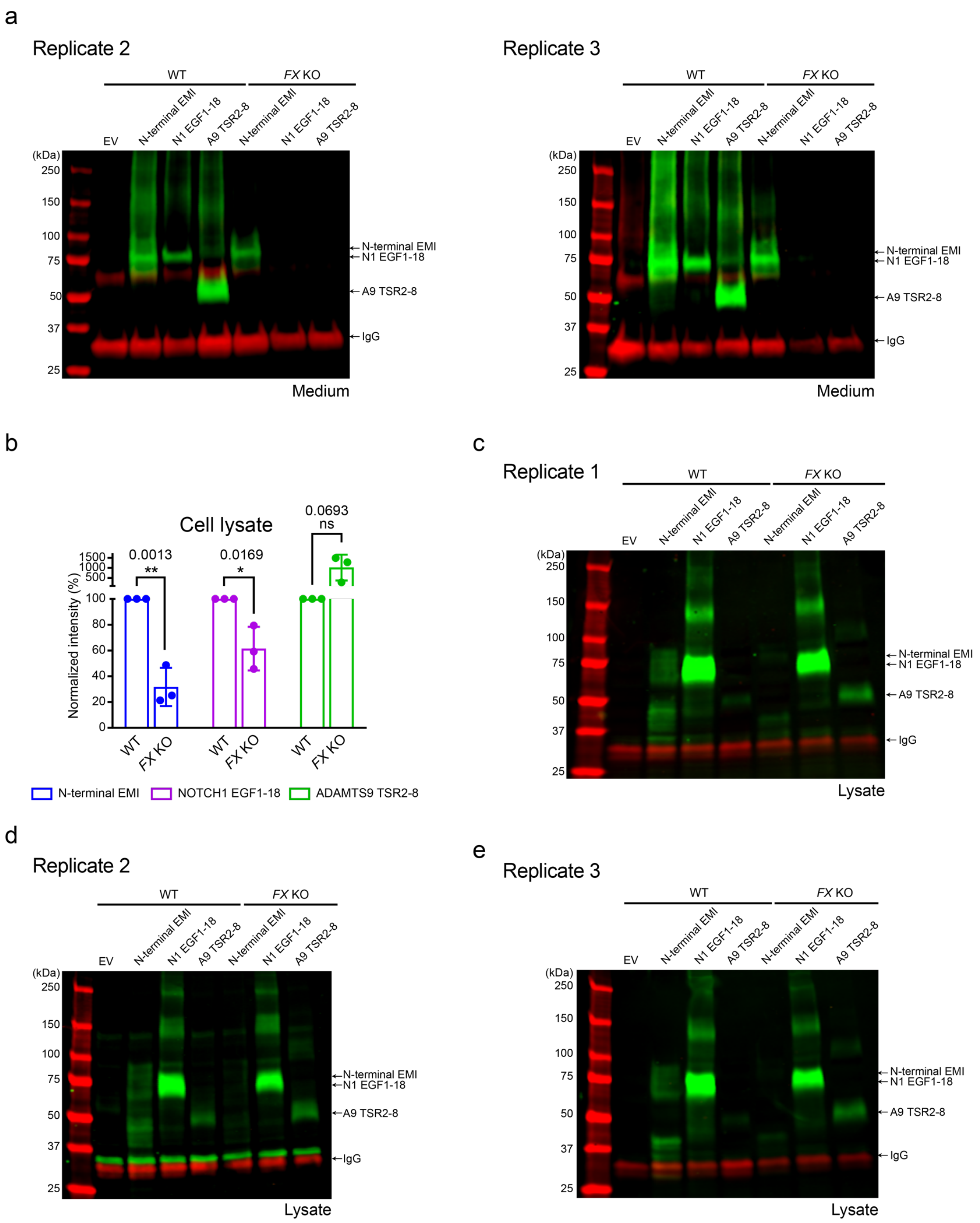

Supplementary Fig. 24. Secretion of N-terminal EMI in HEK293T WT and *FX* KO cells. HEK293T WT or *FX* KO cells were transfected with N-terminal EMI, mouse NOTCH1 EGF1-18, human ADAMTS9 TSR2-8, or empty vector (EV) and IgG (secretion control). Cells were incubated for 48 h. Culture medium and cell lysates were analyzed by Western blot probed with anti-Myc and anti-human IgG antibodies. **a,** In addition to the one replicate used in Fig. 6e, two additional biological replicates of data included for the statistical analysis in Fig. 6h are presented. **b-e,** Cell lysates analyzed by Western blot. Biological triplicates of blots are presented in panel **c**, **d** and **e**. N1 EGF1-18, mNOTCH1 EGF1-18; A9 TSR2-8, hADAMTS9 TSR2-8. Bar graph of quantified band intensity of protein normalized with IgG is presented in panel **b**. Data is shown as mean with +/-SD from biological triplicates of three individual transfections. Statistical analysis is performed with unpaired, two-tailed t test in Prism 7. ns, $p > 0.05$; *, $p < 0.1$; **, $p < 0.01$ compared with control (WT cells).

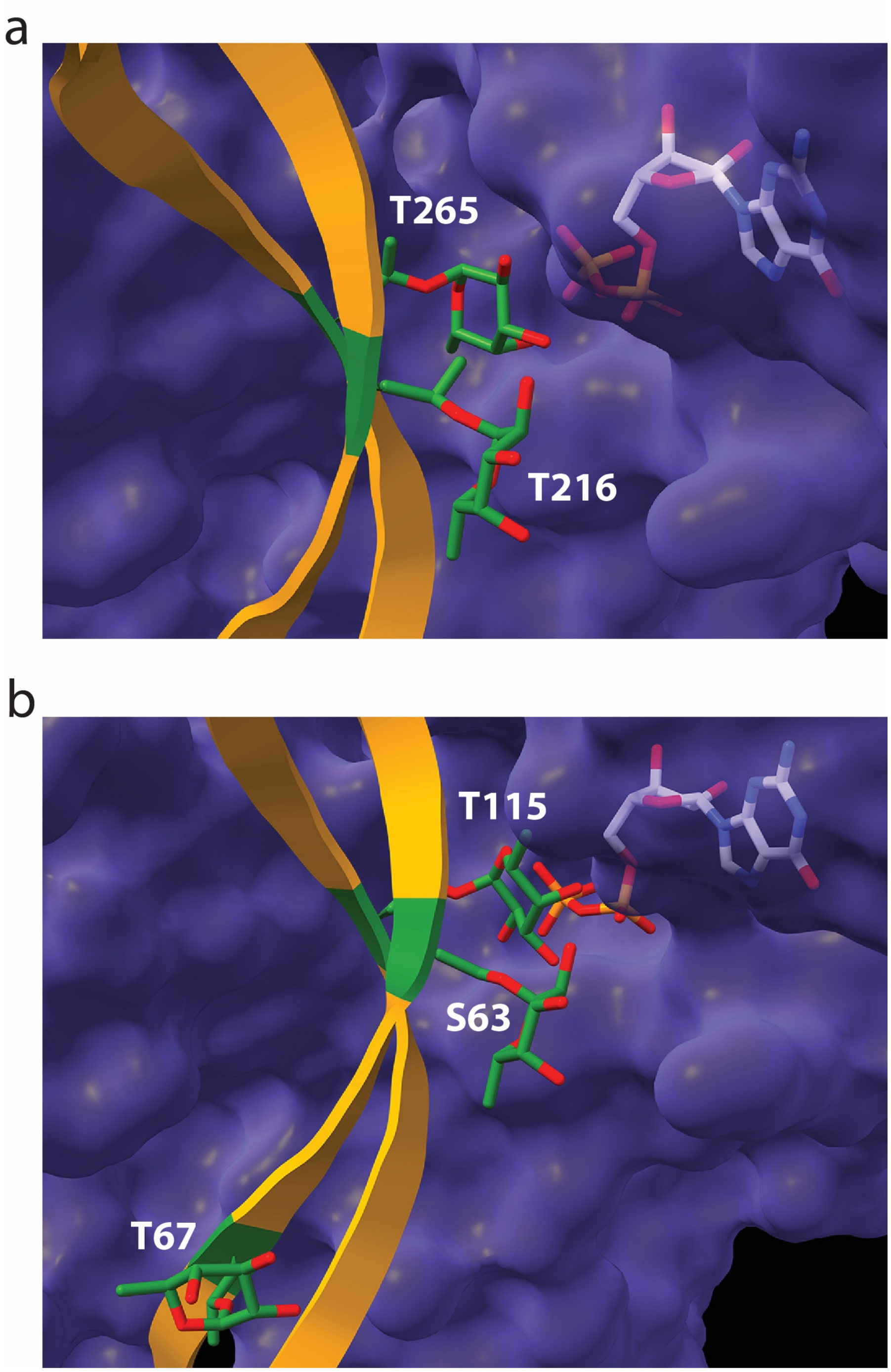

Supplementary Fig. 25. Predicted spatial arrangement for multiply *O*-fucosylated EMI substrates. a, Predicted structure of MMRN1 EMI domain with *O*-fucosylated T216 and T265 folded with FUT11 and GDP using Alphafold3[2]. **b,** Predicted structure of MMRN2 EMI domain with *O*-fucosylated S63, T67 and T115 folded with FUT11 and GDP using Alphafold3[2]. The FUT11 protein is shown as a partially transparent surface depiction colored blue. The EMI domain is shown as a gold cartoon depiction with the *O*-fucosylated residues shown in dark green. The GDP structure is shown as an atomic model.

Supplementary Table 1. Primers used to make expression plasmids

Name		Primer sequence
**pcDNA3.1-hMMRN1 T265A-Myc-His$_6$ and pcDNA3.1-hMMRN1 T216A/T265A-Myc-His$_6$**		
5’	AI452	5’-ATAAAATTGTCGCCTCATTGGATTGGAGGTGCTGTCC-3’
3’	AI464	5’-ATCCAATGAGGCGACAATTTTATGTTGCATCCTATAGACAGG-3’
**pcDNA4-MMRN1-N-terminal EMI-Myc-His$_6$**		
5’	AI364	5’-ATATATAAGCTTGCCACCATGAAGGGGGCAAGATTATTTG-3’
3’	AI365	5’-ATCTAGCTCGAGCAAACTTTGCTGTTCCTGGG-3’
**pcDNA4-MMRN2 EMI-Myc-His$_6$**		
5’	RU004	5’-CCGCTAGGATCCGCCACCATGATTCTGAGCTTGCTG-3’
3’	RU005	5’-CCAGGACTCGAGGTGTTCACAGTTGGGGCCCGTG-3’
**pcDNA4-EMID1 EMI-Myc-His$_6$**		
5’	RU006	5’-CCCCCTAAGCTTGCCACCATGGGAGGACCTCGGGCTTGGG-3’
3’	RU007	5’-GGCAGACTCGAGTTCCTCGCAGCTCACTCCTGAGTGC-3’
**pcDNA4-full length hFUT10-Myc-His$_6$ and pcDNA4-full length hFUT11-Myc-His$_6$**		
5’	AI456	5’-TGGCTAGTTAAGCTTGCCACCATGGTGAGAATC-3’
3’	AI457	5’-CTCGAACTTGCCCAGCTCCACCATCACCTGCAG-3’
5’	AI458	5’-CTGGGCAAGTTCGAGCG-3’
3’	AI459	5’-CCCTCTAGACTCGAGGTCCTTAAAGACCAGGCC-3’
5’	AI454	5’-CTCGAGTCTAGAGGGCCCTTC-3’
3’	AI455	5’-AAGCTTAACTAGCCAGCTTGGG-3’
5’	AI460	5’-TGGCTAGTTAAGCTTGCCACCATGGCCGCCGGC-3’
3’	AI461	5’-CCCGCCGGCCTCCCTCTCGGCCACGCTGCCGTG-3’
5’	AI462	5’-AGGGAGGCCGGCGGGGAG-3’
3’	AI463	5’-CCCTCTAGACTCGAGGAGATGTTGCCTCTTCATGAAGATTTCATGTAG GTAATC-3’

Supplementary Table 2. Oligonucleotides used to make guide RNAs for CRISPR/Cas9 KO plasmids, to amplify genomic sequences covering guide RNA regions or to generate cDNA of *FX* from wild type HEK293T cells

Gene	name	application	sequence	reference
FUT10	FUT10_gRNA2_F	Guide RNA	GATCGATGGAGGTAGGTCCGGTTGA	
	FUT10_gRNA2_R	Guide RNA	AAACTCAACCGGACCTACCTCCATC	
FUT10	FUT10_gRNA3_F	Guide RNA	GATCGTCTGGTGGTCCCCGCTGACG	
	FUT10_gRNA3_R	Guide RNA	AAACCGTCAGCGGGGACCACCAGAC	
FUT11	FUT11_gRNA2_F	Guide RNA	GATCGCGGCACAGACTTCCGCGCGT	
	FUT11_gRNA2_R	Guide RNA	AAACACGCGCGGAAGTCTGTGCCGC	
FUT11	FUT11_gRNA3_F	Guide RNA	GATCGCAGCGGCCATGGGTCCGTAG	
	FUT11_gRNA3_R	Guide RNA	AAACCTACGGACCCATGGCCGCTGC	
FX	FX_gRNA1_F	Guide RNA	GATCGCGTGAGATCGGCGTCTTTAG	
	FX_gRNA1_R	Guide RNA	AAACCTAAAGACGCCGATCTCACGC	
FX	FX_gRNA3_F	Guide RNA	GATCGGACCCGCGCCCTGTTTGAGA	
	FX_gRNA3_R	Guide RNA	AAACTCTCAAACAGGGCGCGGGTCC	
FUT10	FUT10_gRNA2-3_F	Genotyping	TCATGGTTGAGCTGGGGAAGT	NC_000008.11 region (33308061...33473146)
	FUT10_gRNA2-3_R	Genotyping	GAGAATTCAGAAGCCACCACGC	
FUT11	FUT11_gRNA2-3_F	Genotyping	GGCTGCCGGAGTGGACATGG	NC_000010.11 Region (73772276..73780254)
	FUT11_gRNA2-3_R	Genotyping	CCGTGGCTCAGCAAGAAGTTGT	
FX	FX_gRNA1-3_P1F	Genotyping	AGCCTCCCACTGTTTGACAT	NC_000008.11 Region (143612618..143618048)
	FX_gRNA1-3_P1R	Genotyping	GCCACCCTCACTTACCCAGAA	
FX	FX_EcoRI_F	cloning	GAATTCGCCACCATGGGTGAACCCCA GGGATCCA	NM_001413408.1
	FX_EcoRV_R	cloning	GATATCCTTCCGGGCCTGCTCGTAGTT	

Supplementary References

1. Lu, L. et al. In vivo evidence for GDP-fucose transport in the absence of transporter SLC35C1 and putative transporter SLC35C2. *J Biol Chem* **299**, 105406 (2023).
2. Abramson, J. et al. Accurate structure prediction of biomolecular interactions with AlphaFold 3. *Nature* **630**, 493-500 (2024).

Uncropped gels for Supplementary Figures

Image Display Values

Channel	Color	Minimum	Maximum	K
800	Green	0.590	67.9	1

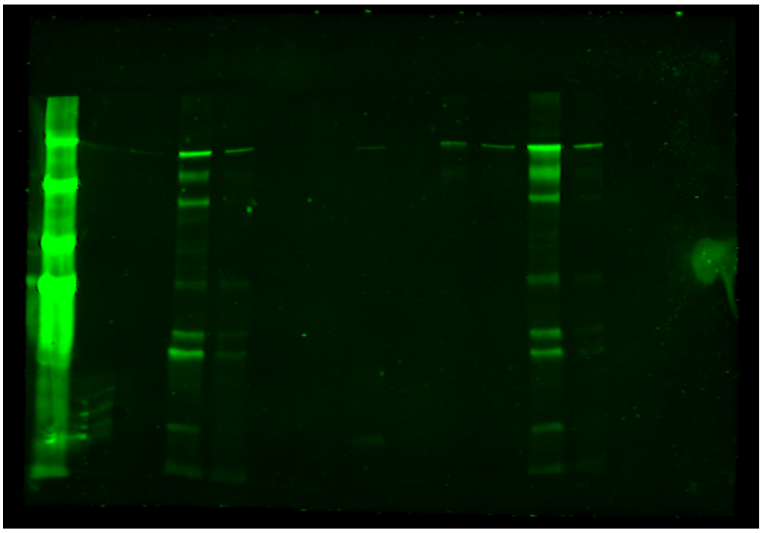

Fig. S3a

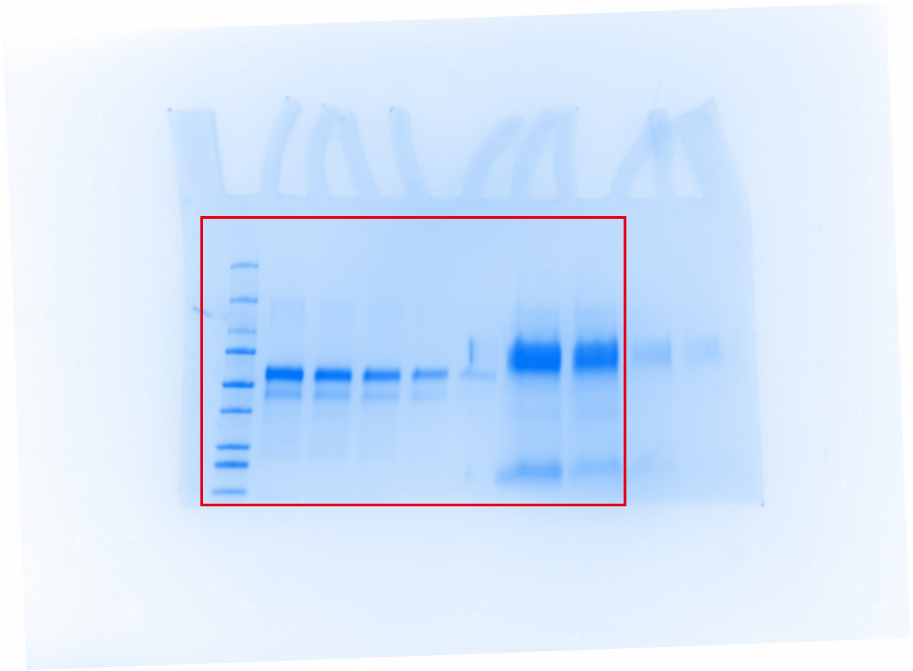

Fig. S6b

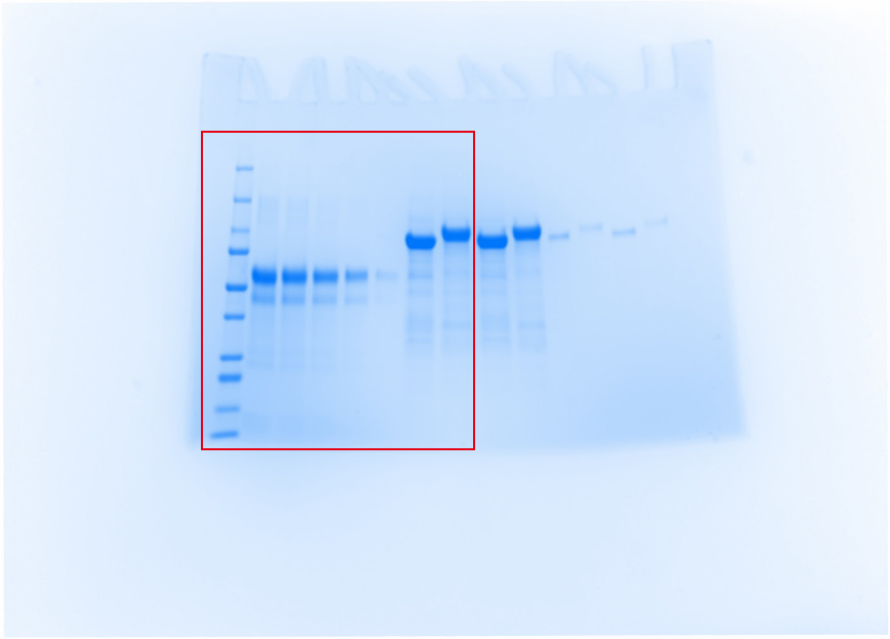

Fig. S6c

Image Display Values

Channel	Color	Minimum	Maximum	K
700	Red	15.7	105	1
800	Green	1.29	17.2	1

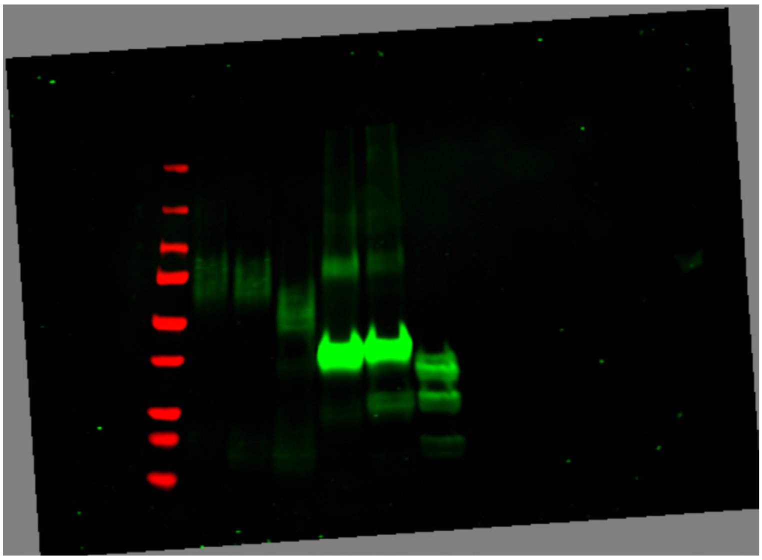

Fig. S9

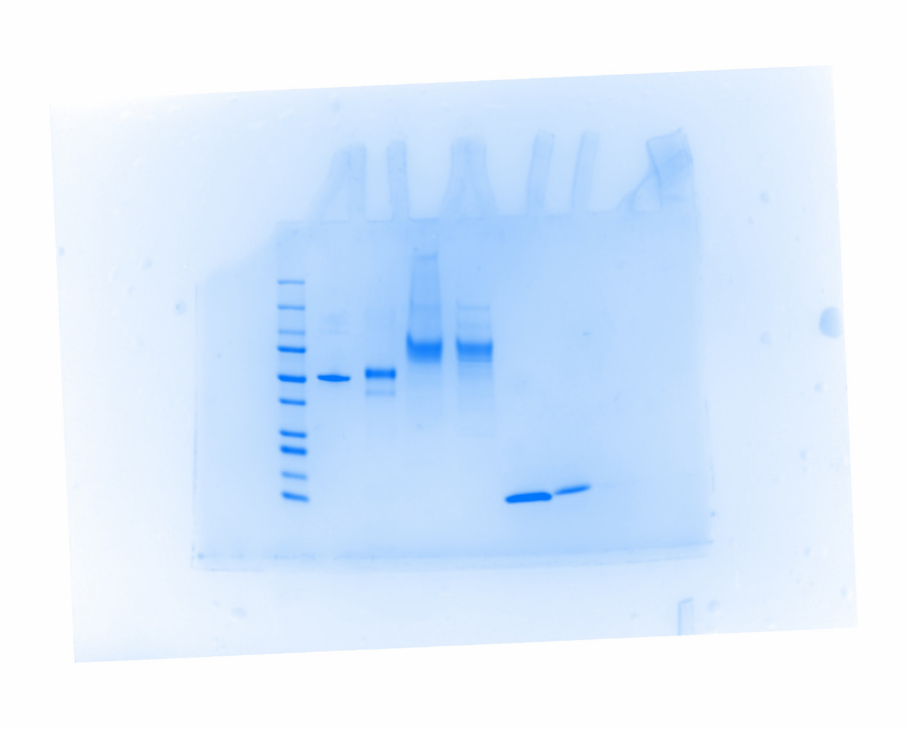

Fig. S20a

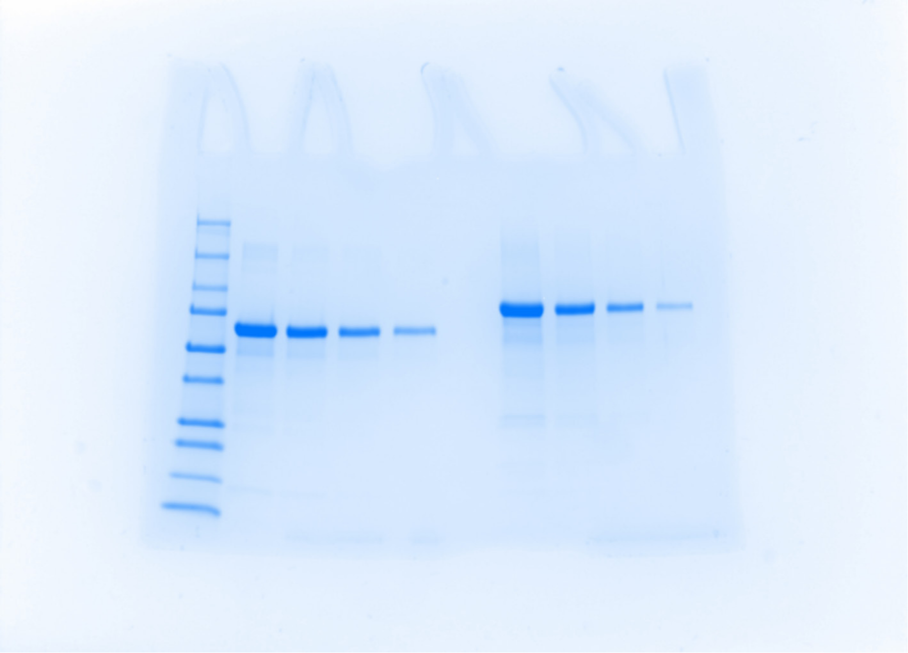

Fig. S20b

Image Display Values

Channel	Color	Minimum	Maximum	K
700	Red	22.7	298	1
800	Green	1.75	2.47	1

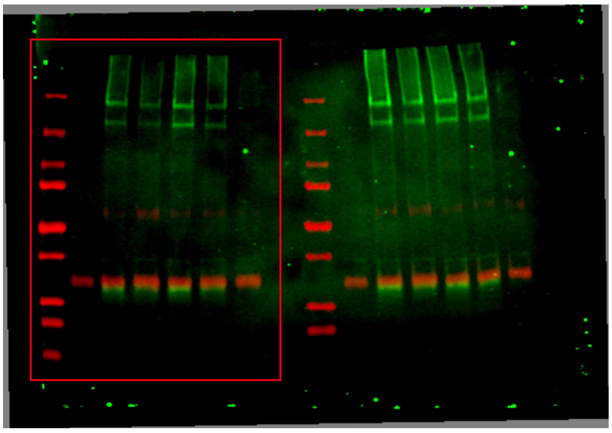

Image Display Values

Channel	Color	Minimum	Maximum	K
700	Red	45.0	129	1
800	Green	1.88	3.18	1

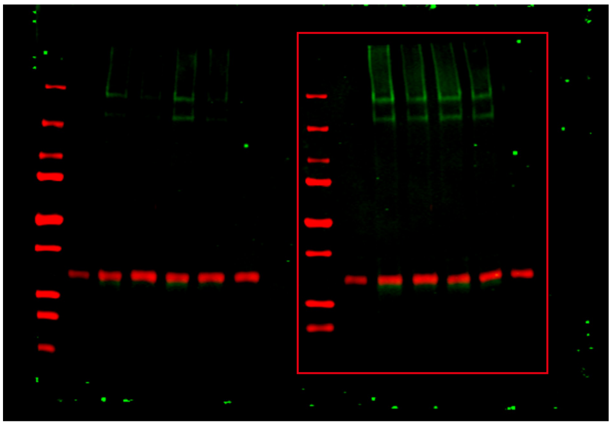

Fig. S22b

Image Display Values

Channel	Color	Minimum	Maximum	K
700	Red	3.99	49.5	1
800	Green	3.71	85.0	1

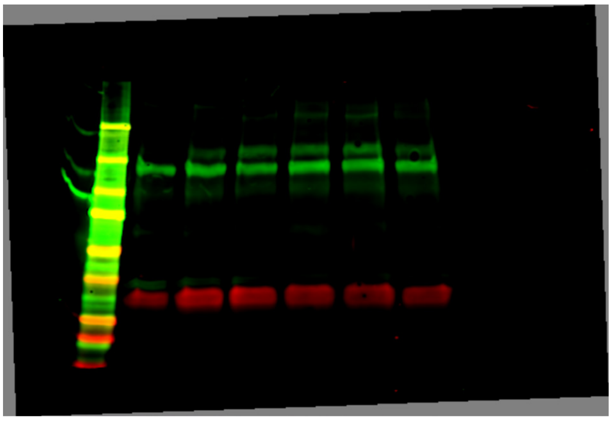

Fig. S22d

Image Display Values

Channel	Color	Minimum	Maximum	K
700	Red	4.65	43.9	1
800	Green	1.60	12.6	1

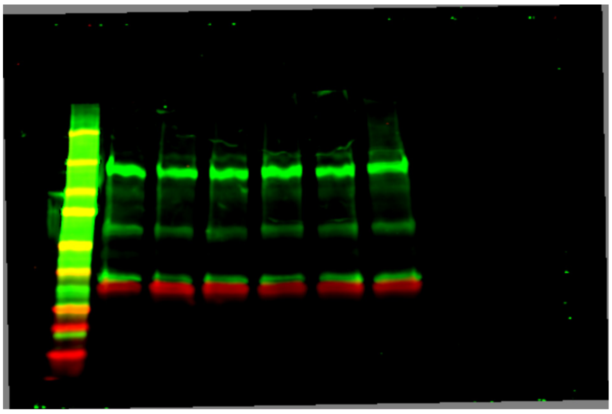

Fig. S22e

Image Display Values

Channel	Color	Minimum	Maximum	K
700	Red	4.59	110	1
800	Green	1.63	12.1	1

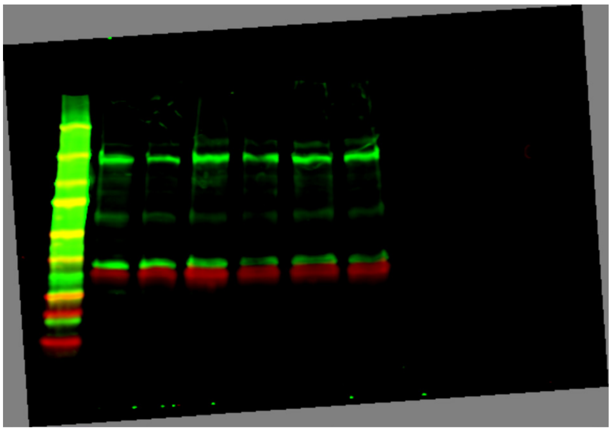

Fig. S22f

Image Display Values

Channel	Color	Minimum	Maximum	K
700	Red	3.74	80.5	1
800	Green	3.23	17.1	1

Image Display Values

Channel	Color	Minimum	Maximum	K
700	Red	3.64	42.7	1
800	Green	2.78	31.8	1

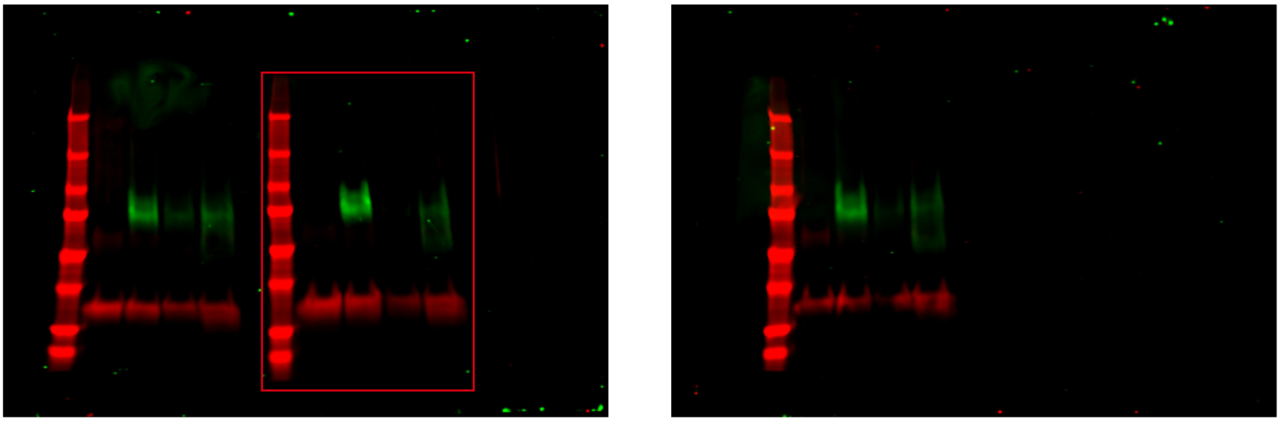

Fig. S23a

Image Display Values

Channel	Color	Minimum	Maximum	K
800	Green	4.09	31.0	1

Image Display Values

Channel	Color	Minimum	Maximum	K
700	Red	3.64	20.2	1

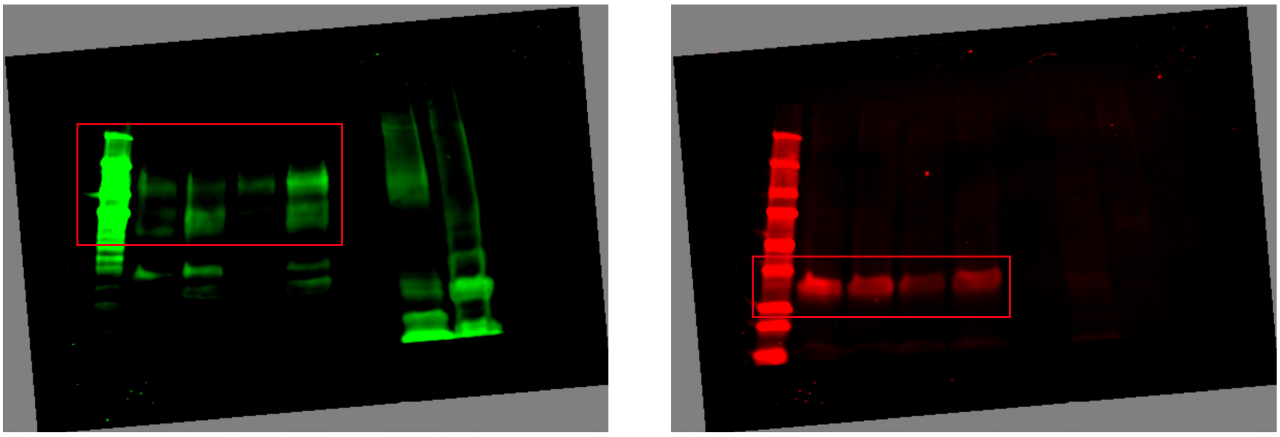

Fig. S23c

Image Display Values

Channel	Color	Minimum	Maximum	K
800	Green	4.58	116	1

Image Display Values

Channel	Color	Minimum	Maximum	K
700	Red	6.48	272	1

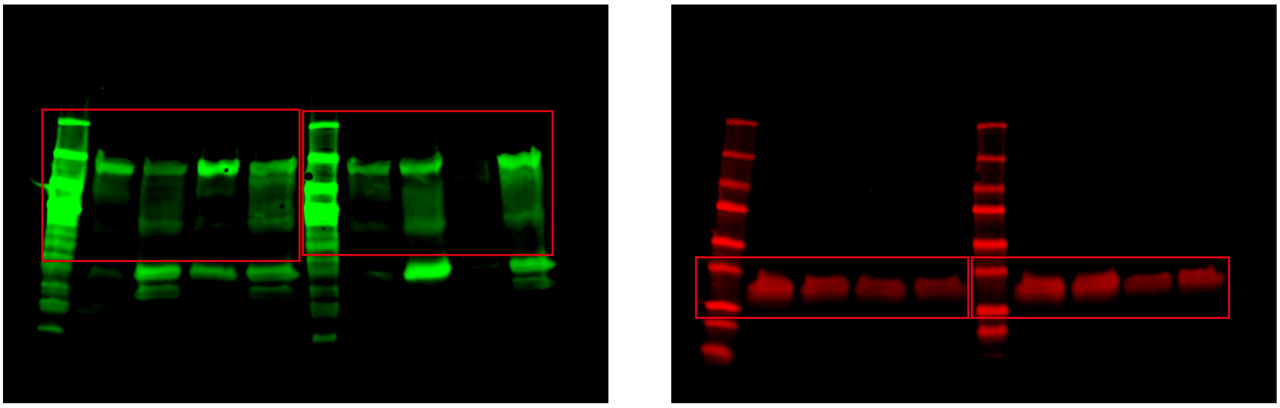

Fig. S23d,e

Image Display Values

Channel	Color	Minimum	Maximum	K
700	Red	13.2	347	1
800	Green	2.72	270	1

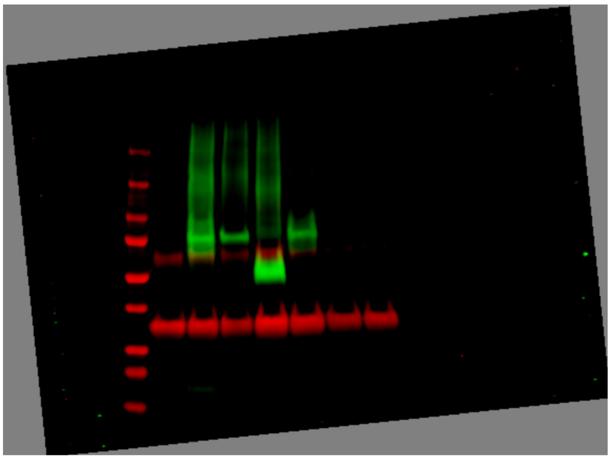

Image Display Values

Channel	Color	Minimum	Maximum	K
700	Red	6.12	124	1
800	Green	1.44	65.5	1

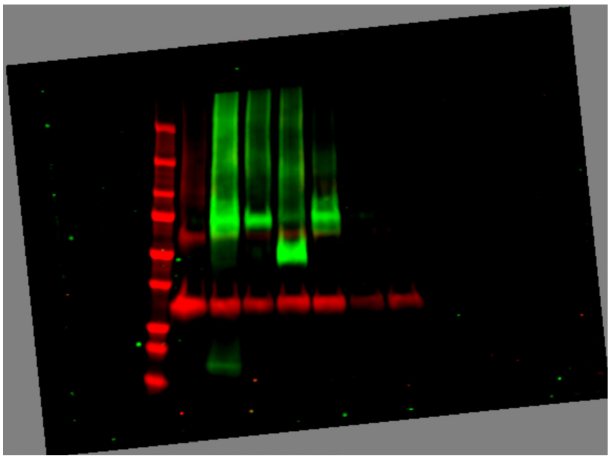

Fig. S24a

Image Display Values

Channel	Color	Minimum	Maximum	K
700	Red	4.29	113	1
800	Green	3.07	23.3	1

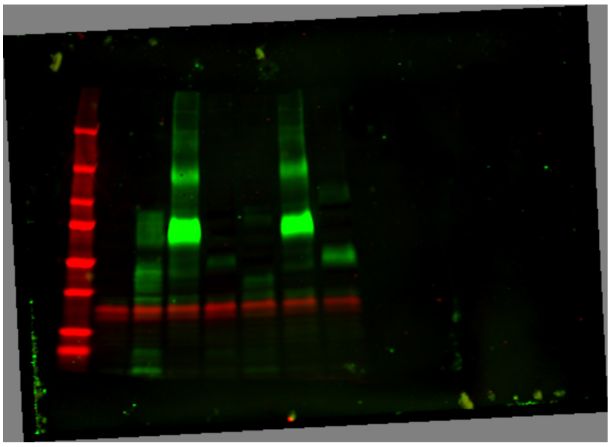

Fig. S24c

Image Display Values

Channel	Color	Minimum	Maximum	K
700	Red	5.71	82.9	1
800	Green	1.14	29.0	1

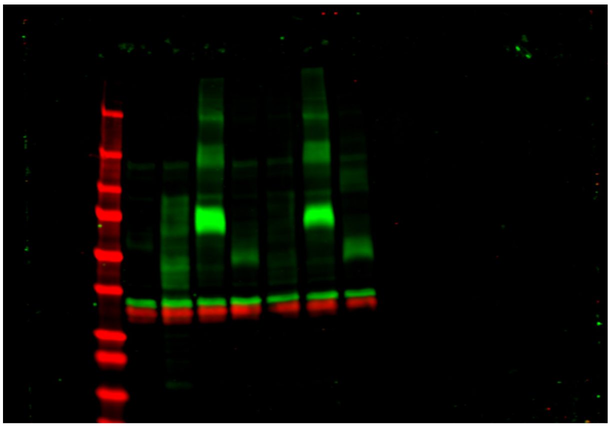

Fig. S24d

Image Display Values

Channel	Color	Minimum	Maximum	K
700	Red	5.30	84.4	1
800	Green	3.15	54.2	1

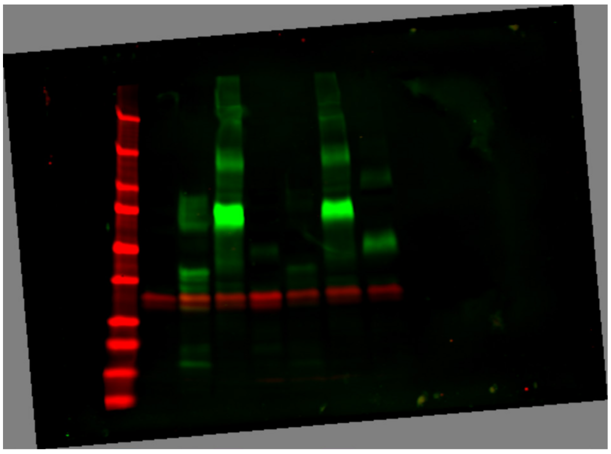

Fig. S24e
