# Supplementary material for: FUT10 and FUT11 are protein O-fucosyltransferases that modify protein EMI domains
Source: Nat Chem Biol. 2025 Jan 7;21(4):598–610. doi: 10.1038/s41589-024-01815-x (PMC11949838; doi:10.1038/s41589-024-01815-x)
Supplement: Supplementary file 2 — Reporting Summary [file 41589_2024_1815_MOESM2_ESM.pdf]

nature portfolio

Corresponding author(s): Robert S. Haltiwanger, Mark Larance

Last updated by author(s): Nov 12, 2024

Reporting Summary

Nature Portfolio wishes to improve the reproducibility of the work that we publish. This form provides structure for consistency and transparency in reporting. For further information on Nature Portfolio policies, see our Editorial Policies and the Editorial Policy Checklist.

Statistics

For all statistical analyses, confirm that the following items are present in the figure legend, table legend, main text, or Methods section.

n/a	Confirmed	
☐	☒	The exact sample size (*n*) for each experimental group/condition, given as a discrete number and unit of measurement
☐	☒	A statement on whether measurements were taken from distinct samples or whether the same sample was measured repeatedly
☐	☒	The statistical test(s) used AND whether they are one- or two-sided<br>*Only common tests should be described solely by name; describe more complex techniques in the Methods section.*
☐	☒	A description of all covariates tested
☒	☐	A description of any assumptions or corrections, such as tests of normality and adjustment for multiple comparisons
☐	☒	A full description of the statistical parameters including central tendency (e.g. means) or other basic estimates (e.g. regression coefficient) AND variation (e.g. standard deviation) or associated estimates of uncertainty (e.g. confidence intervals)
☐	☒	For null hypothesis testing, the test statistic (e.g. *F*, *t*, *r*) with confidence intervals, effect sizes, degrees of freedom and *P* value noted<br>*Give P values as exact values whenever suitable.*
☒	☐	For Bayesian analysis, information on the choice of priors and Markov chain Monte Carlo settings
☒	☐	For hierarchical and complex designs, identification of the appropriate level for tests and full reporting of outcomes
☒	☐	Estimates of effect sizes (e.g. Cohen's *d*, Pearson's *r*), indicating how they were calculated

Our web collection on statistics for biologists contains articles on many of the points above.

Software and code

Policy information about availability of computer code

Data collection	MS: Thermo Fisher Easy nLC-1200 system coupled with Q-Exactive Plus Mass Spectrometer, Tune 2.12 (Build 3134), Xcalibur (version 4.4.16.14) and SciexOS (version 3.4.0.19154)<br>Western Blot: LI-COR Image Studio (version 5.2.5)<br>Flow Cytometry: BD Accuri C6 (version 1.0.264.21)<br>Plate reader: BioTek Gen5 (version 3.03)<br>Circular Dichroism: Spectra Manager Version 1.08.01 (Build 1)<br>Thermostability assay: Panta Analysis software (version 1.1)<br>Confocal microscopy: Leica LAS X software (version 5.2)<br>Protein structural prediction: ColabFold v1.5.5 with AlphaFold2 using MMseqs2
Data analysis	General Data Analysis: GraphPad Prism (version 7.0), Microsoft Excel (version 2401)<br>MS: Xcalibur Qual Browser (version 4.0.27.19) with Gauss algorithm, Byonic (version 4.1.10)<br>Western blot analysis: LI-COR Image Studio (version 5.2.5)<br>Flow cytometry data analysis: Flowjo (version 10.8.1)<br>Protein structural analysis: UCSF ChimeraX (version 1.7.1)<br>Circular Dichroism data analysis: DichroWeb server<br>Thermostability assay: Panta Analysis software (version 1.1)<br>Confocal microscopy: Fiji ImageJ (version 1.54f)

For manuscripts utilizing custom algorithms or software that are central to the research but not yet described in published literature, software must be made available to editors and reviewers. We strongly encourage code deposition in a community repository (e.g. GitHub). See the Nature Portfolio guidelines for submitting code & software for further information.

Data

Policy information about availability of data

All manuscripts must include a data availability statement. This statement should provide the following information, where applicable:

- Accession codes, unique identifiers, or web links for publicly available datasets
- A description of any restrictions on data availability
- For clinical datasets or third party data, please ensure that the statement adheres to our policy

Confocal images in Extended Data Fig. 8c were obtained from the Human Protein Atlas (https://www.proteinatlas.org/ENSG00000172728-FUT10/subcellular). All data generated and analyzed during this study are provided within the paper and the Supplementary data. Source data files are supplied with this paper. Note for Supplementary Data S1: Annotated HCD-MS/MS spectra for peptides used in the generation of EICs in Fig. 1d, Fig. 2, Fig. 6b, Extended Data Fig. 7, Fig. S2 and Fig. S3. Due to the lability of the fucose-peptide bond in high energy collision dissociation, Byonic is frequently unable to correctly assign the O-fucosylation site when multiple serine or threonine residues are present in a peptide. All assignments are based on the well-documented sequences for O-fucosylation of EGF repeats (C2XXXX[S/T]C3) and Group 1 TSRs (C1XX[S/T]C2). Note for Supplementary Data S2: Alphafold2-multimer alignment error (PAE) plots for the predicted EMI domain and fucosyltransferases structures.

Human research participants

Policy information about studies involving human research participants and Sex and Gender in Research.

Reporting on sex and gender	N/A
Population characteristics	N/A
Recruitment	N/A
Ethics oversight	N/A

Note that full information on the approval of the study protocol must also be provided in the manuscript.

Field-specific reporting

Please select the one below that is the best fit for your research. If you are not sure, read the appropriate sections before making your selection.

☒ Life sciences ☐ Behavioural & social sciences ☐ Ecological, evolutionary & environmental sciences

For a reference copy of the document with all sections, see nature.com/documents/nr-reporting-summary-flat.pdf

Life sciences study design

All studies must disclose on these points even when the disclosure is negative.

Sample size	Sample sizes were determined based on our previous publication for equivalent experiments (examples include: DOI: 10.1016/j.devcel.2016.12.013; DOI: 10.1074/jbc.M116.732537 and DOI: 10.1038/nchembio.2520). MS label-free quantification, image-based quantification and kinetics data were collected from n=3 biological replicates as specified in the Methods.<br>Glycopeptide IDs from HEK293T WT, POFUT1 KO, POFUT2 KO, FX KO, SLC35C1 KO, and FUT10/11 DKO cells in Fig. 2, Fig. 6b, Extended Data Fig. 7, and Fig. S2 and their corresponding extracted ion chromatograms (EICs) and MS2 spectra used a single replicate each, because one replicate was sufficient to demonstrate the presence of glycopeptides as the high-quality MS data obtained from precursors and MS2 fragmentations provide enough accuracy for glycopeptide assignment.
Data exclusions	No data were excluded.
Replication	All biological replicates were obtained from biologically independent experiments at least in triplicate. Reported results were consistently reproducible across multiple experiments with all replicates generating similar results.
Randomization	Randomization was not applicable in our study as we were comparing purified samples (purified enzymes and substrates) under well-controlled conditions. To account for the lack of randomization, all experiments were performed with appropriate controls.
Blinding	Measurements and analysis were not blinded as a negative control was run in parallel with every experiment, and the readouts of MS were objective, no observer bias expected in this study.

Reporting for specific materials, systems and methods

We require information from authors about some types of materials, experimental systems and methods used in many studies. Here, indicate whether each material, system or method listed is relevant to your study. If you are not sure if a list item applies to your research, read the appropriate section before selecting a response.

Materials & experimental systems

n/a	Involved in the study
☐	☒ Antibodies
☐	☒ Eukaryotic cell lines
☒	☐ Palaeontology and archaeology
☒	☐ Animals and other organisms
☒	☐ Clinical data
☒	☐ Dual use research of concern

Methods

n/a	Involved in the study
☒	☐ ChIP-seq
☐	☒ Flow cytometry
☒	☐ MRI-based neuroimaging

Antibodies

Antibodies used	Anti-c-Myc Magnetic Beads (Thermo Fisher, Cat #88842, Clone 9E10)<br>Mouse anti-c-Myc Antibody (Invitrogen, Cat #MA1-980, Clone 9E10)<br>Mouse anti-Histidine Tag Antibody (Bio-Rad, Cat #MCA1396, Clone AD1.1.10)<br>IDRye 800-conjugated goat anti-mouse IgG antibody (LI-COR, Cat #926-32210)<br>IDRye 680-conjugated goat anti-human IgG antibody (LI-COR, Cat #926-68078)<br>Mouse anti-FLAG M2 antibody (Sigma, Cat #B3111-1MG)<br>Mouse anti-PDI (ThermoFisher, Cat #MA3-019, Clone RL90)<br>Mouse anti-GM130 (BD Biosciences, Cat #610823)<br>Rabbit anti-FUT10 (Proteintech Group, Cat #18660-1-AP)<br>Rabbit anti-FUT11 (Proteintech Group, Cat #17175-1-AP)<br>Goat anti-Rabbit IgG (H+L) with AlexaFluor 488 (ThermoFisher, Cat #A-11034)<br>Goat anti-Mouse IgG (H+L) with AlexaFluor 647 (ThermoFisher, Cat #A-21236)
Validation	All antibodies were used following previous publications and/or by manufacturer protocols:<br>Anti-c-Myc Magnetic Beads: https://www.thermofisher.com/order/catalog/product/88842?SID=srch-srp-88842<br>Mouse anti-c-Myc Antibody: https://www.thermofisher.com/antibody/product/c-Myc-Antibody-clone-9E10-Monoclonal/MA1-980<br>Mouse anti-Histidine Tag Antibody: https://www.bio-rad-antibodies.com/monoclonal/synthetic-peptide-histidine-tag-antibody-ad1-1-10-mca1396.html?f=purified&JSESSIONID_STERLING=119BA86E4F4435262B93CDD8A4B202C1.ecommerce2&evCntryLang=US-en&cntry=US&thirdPartyCookieEnabled=true<br>IDRye 800-conjugated goat anti-mouse IgG antibody: https://www.licor.com/bio/reagents/irdye-800cw-goat-anti-mouse-igg-secondary-antibody<br>IDRye 680-conjugated goat anti-human IgG antibody:https://www.licor.com/bio/reagents/irdye-680rd-goat-anti-human-igg-secondary-antibody<br>Mouse anti-FLAG M2 antibody: https://www.sigmaaldrich.com/US/en/product/sigma/b3111<br>Mouse anti-PDI antibody: https://www.thermofisher.com/antibody/product/PDI-Antibody-clone-RL90-Monoclonal/MA3-019<br>Mouse anti-GM130 antibody: https://www.bdbiosciences.com/en-au/products/reagents/microscopy-imaging-reagents/immunofluorescence-reagents/purified-mouse-anti-gm130.610823<br>Rabbit anti-FUT10 antibody: https://www.ptglab.com/products/FUT10-Antibody-18660-1-AP.htm<br>Rabbit anti-FUT11 antibody: https://www.ptglab.com/products/FUT11-Antibody-17175-1-AP.htm<br>Goat anti-Rabbit IgG (H+L) with AlexaFluor 488 antibody: https://www.thermofisher.com/antibody/product/Goat-anti-Rabbit-IgG-H-L-Highly-Cross-Adsorbed-Secondary-Antibody-Polyclonal/A-11034<br>Goat anti-Mouse IgG (H+L) with AlexaFluor 647 antibody: https://www.thermofisher.com/antibody/product/Goat-anti-Mouse-IgG-H-L-Highly-Cross-Adsorbed-Secondary-Antibody-Polyclonal/A-21236

Eukaryotic cell lines

Policy information about cell lines and Sex and Gender in Research

Cell line source(s)	HEK293T cells (ATCC CRL-3216)<br>HEK293 cells (ATCC CRL-1573)<br>HEK293F cells were a gift from Dr. Kelley Moremen (UGA) (original commercial source: Thermo R79007)<br>SCTI003-A human induced pluripotent cells (STEMCELL Technologies)<br>HeLa cells (ATCC CCL-2)
Authentication	Cells were obtained from and authenticated by ATCC, STEMCELL Technologies, and Thermo. No further authentication was performed beyond inspection based on morphological criteria.
Mycoplasma contamination	Cell lines were tested negative for mycoplasma contamination.
Commonly misidentified lines (See ICLAC register)	No commonly misidentified cell lines were used in this study.

Flow Cytometry

Plots

Confirm that:

☒ The axis labels state the marker and fluorochrome used (e.g. CD4-FITC).

☒ The axis scales are clearly visible. Include numbers along axes only for bottom left plot of group (a 'group' is an analysis of identical markers).

☐ All plots are contour plots with outliers or pseudocolor plots.

☐ A numerical value for number of cells or percentage (with statistics) is provided.

Methodology

Sample preparation	Cells were lifted with cold PBS.
Instrument	Accuri C6 Puls flow cytometer (BD Biosciences)
Software	BD Accuri C6 (version 1.0.264.21) was used to collect the data. Data analysis was done in FlowJo (version 10.8.1).
Cell population abundance	Each condition was represented by biological triplicate. 20,000 events were collected for each sample.
Gating strategy	Cells were incubated with fluorescein-conjugated AAL (Vector Laboratories, Cat: F-1391-1). Single cells were determined by FSC and SSC parameters. Single cells were gated with positive fluorescein signal for analysis. For cells transfected with mCherry-FX or mCherry-EV (empty vector), cells were first gated with positive mCherry expression, then gated with positive fluorescein signal for analysis.

☒ Tick this box to confirm that a figure exemplifying the gating strategy is provided in the Supplementary Information.
