# Supplementary material for: FUT10 and FUT11 are protein O-fucosyltransferases that modify protein EMI domains
Source: Nat Chem Biol. 2025 Jan 7;21(4):598–610. doi: 10.1038/s41589-024-01815-x (PMC11949838; doi:10.1038/s41589-024-01815-x)

Supplementary Data S1

EIC and MS2 spectrum for peptides used in Fig. 1d

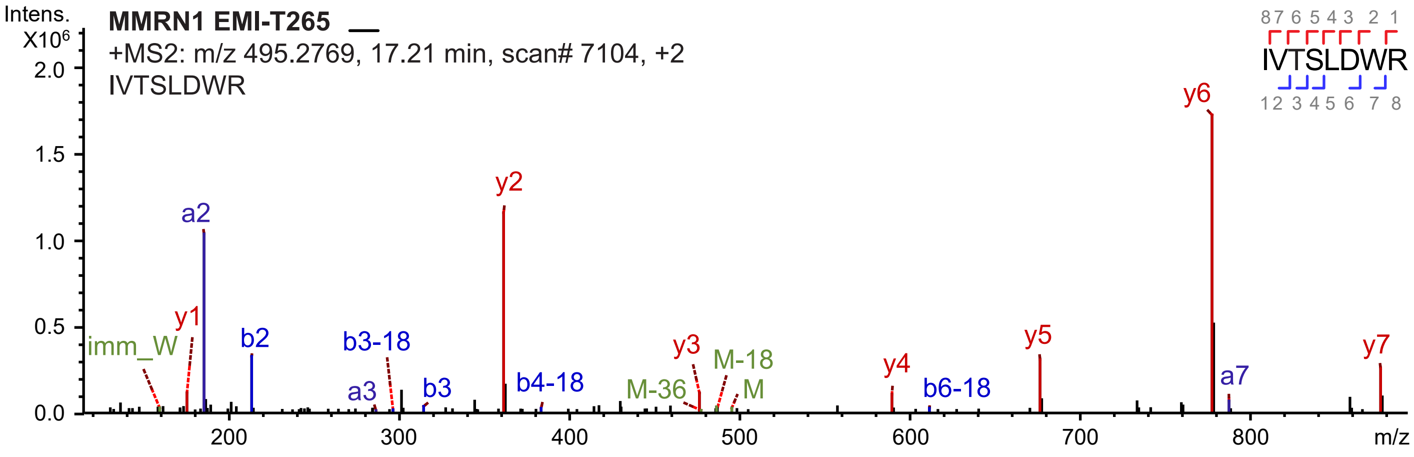

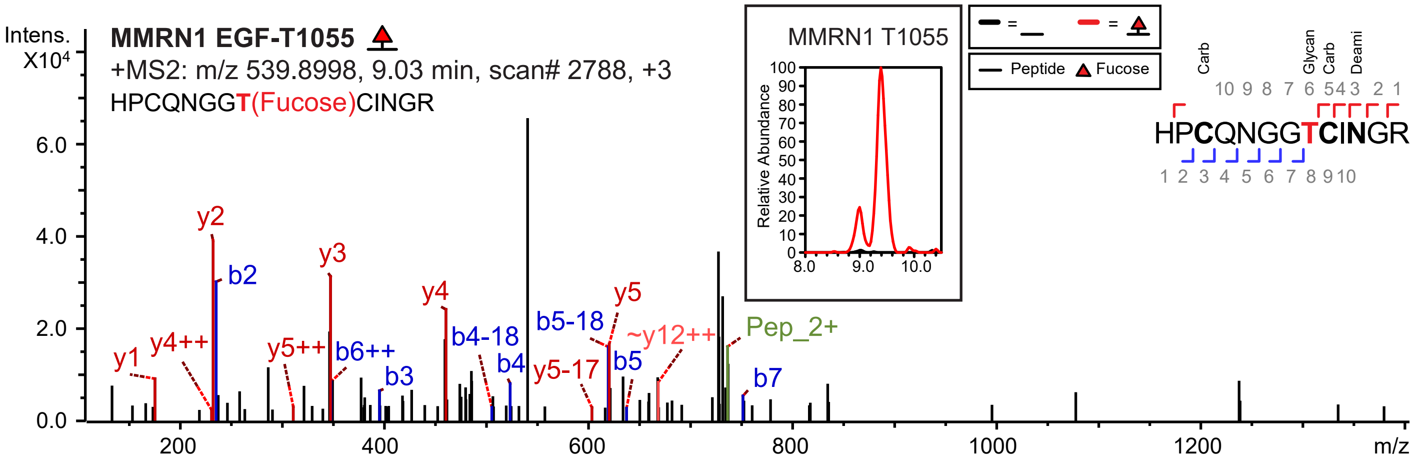

MS2 spectra for peptides used in Fig. 2a

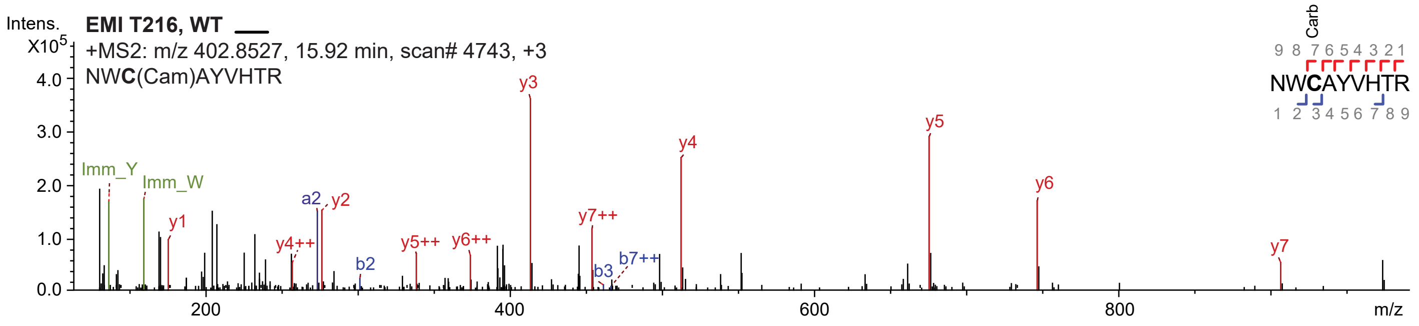

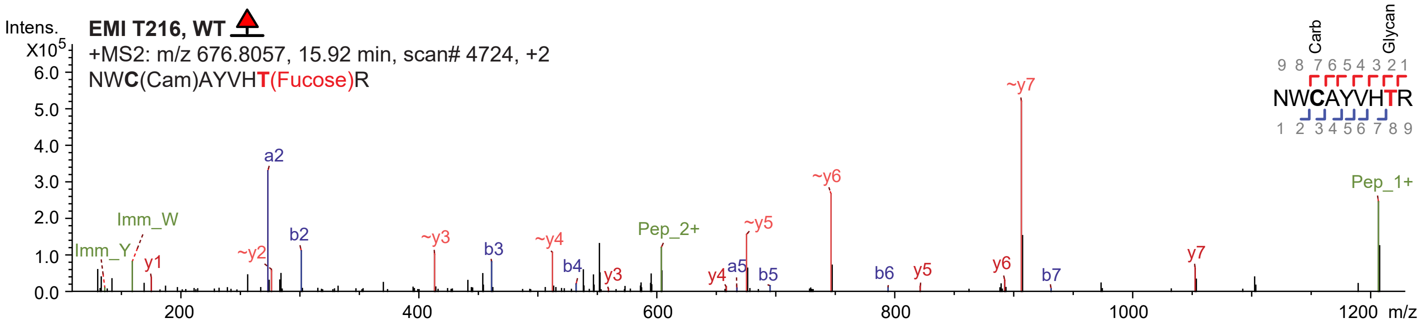

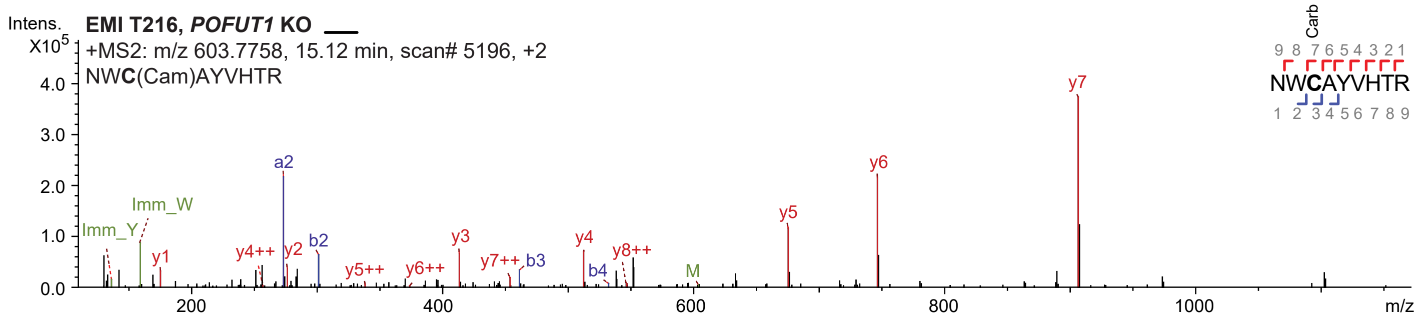

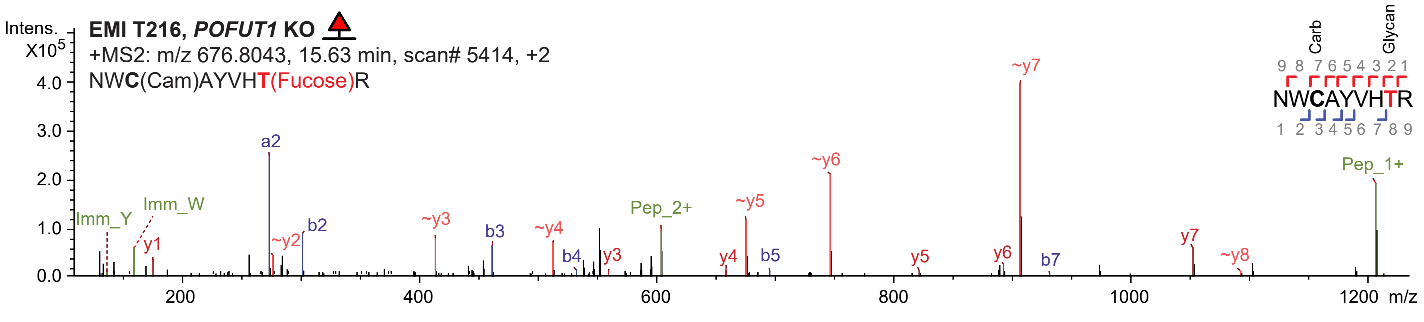

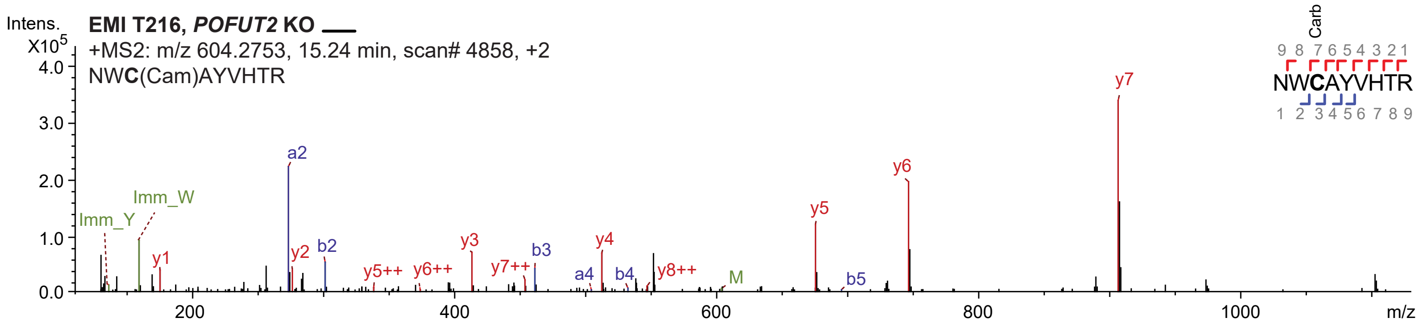

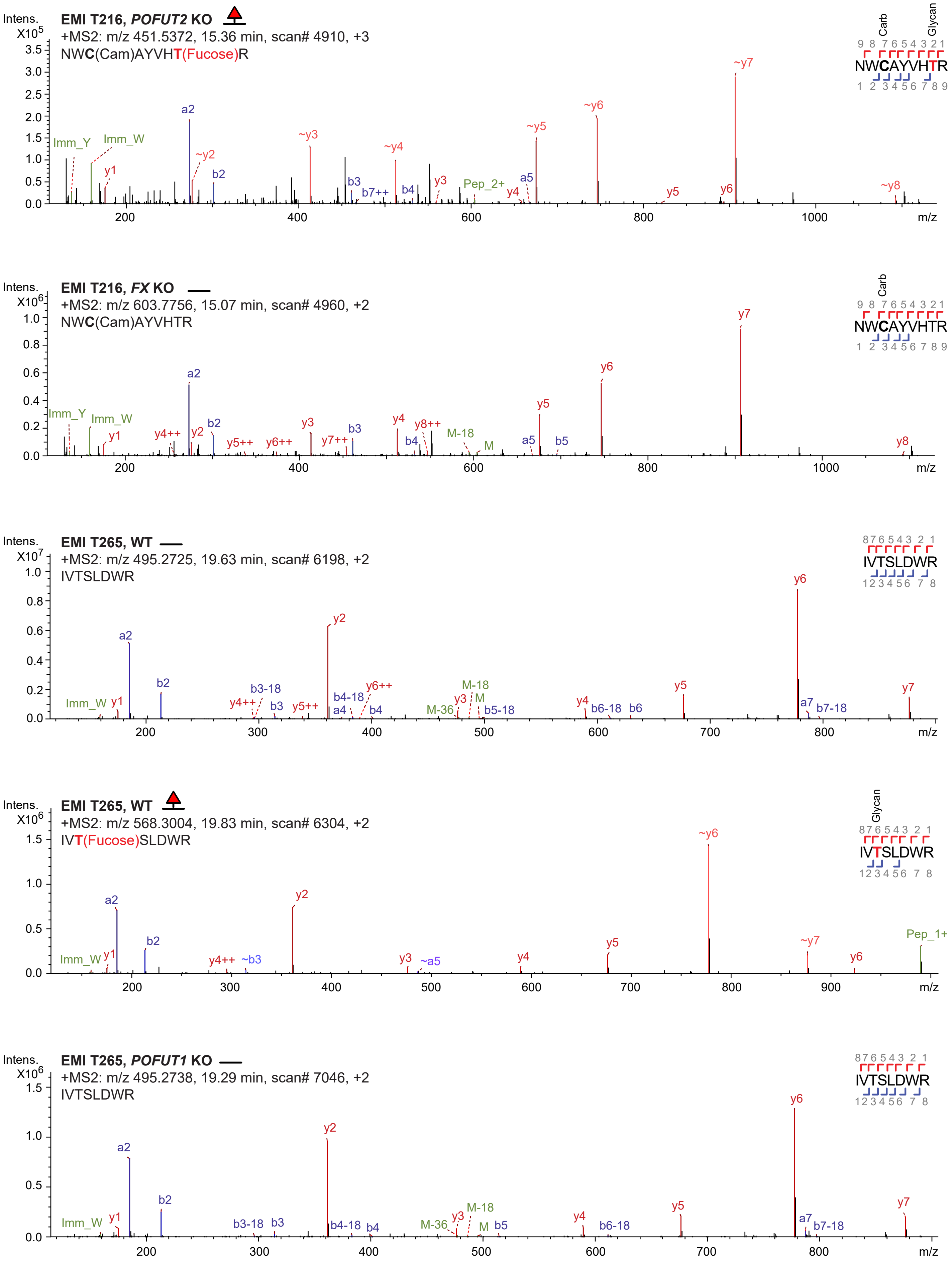
EMI T216, POFUT2 KO
+MS2: m/z 451.5372, 15.36 min, scan# 4910, +3
NWC(Cam)AYVHT(Fucose)R
Intens. X10^5
m/z
EMI T216, FX KO
+MS2: m/z 603.7756, 15.07 min, scan# 4960, +2
NWC(Cam)AYVHTR
Intens. X10^6
m/z
EMI T265, WT
+MS2: m/z 495.2725, 19.63 min, scan# 6198, +2
IVTSLDWR
Intens. X10^7
m/z
EMI T265, WT
+MS2: m/z 568.3004, 19.83 min, scan# 6304, +2
IVT(Fucose)SLDWR
Intens. X10^6
m/z
EMI T265, POFUT1 KO
+MS2: m/z 495.2738, 19.29 min, scan# 7046, +2
IVTSLDWR
Intens. X10^6
m/z

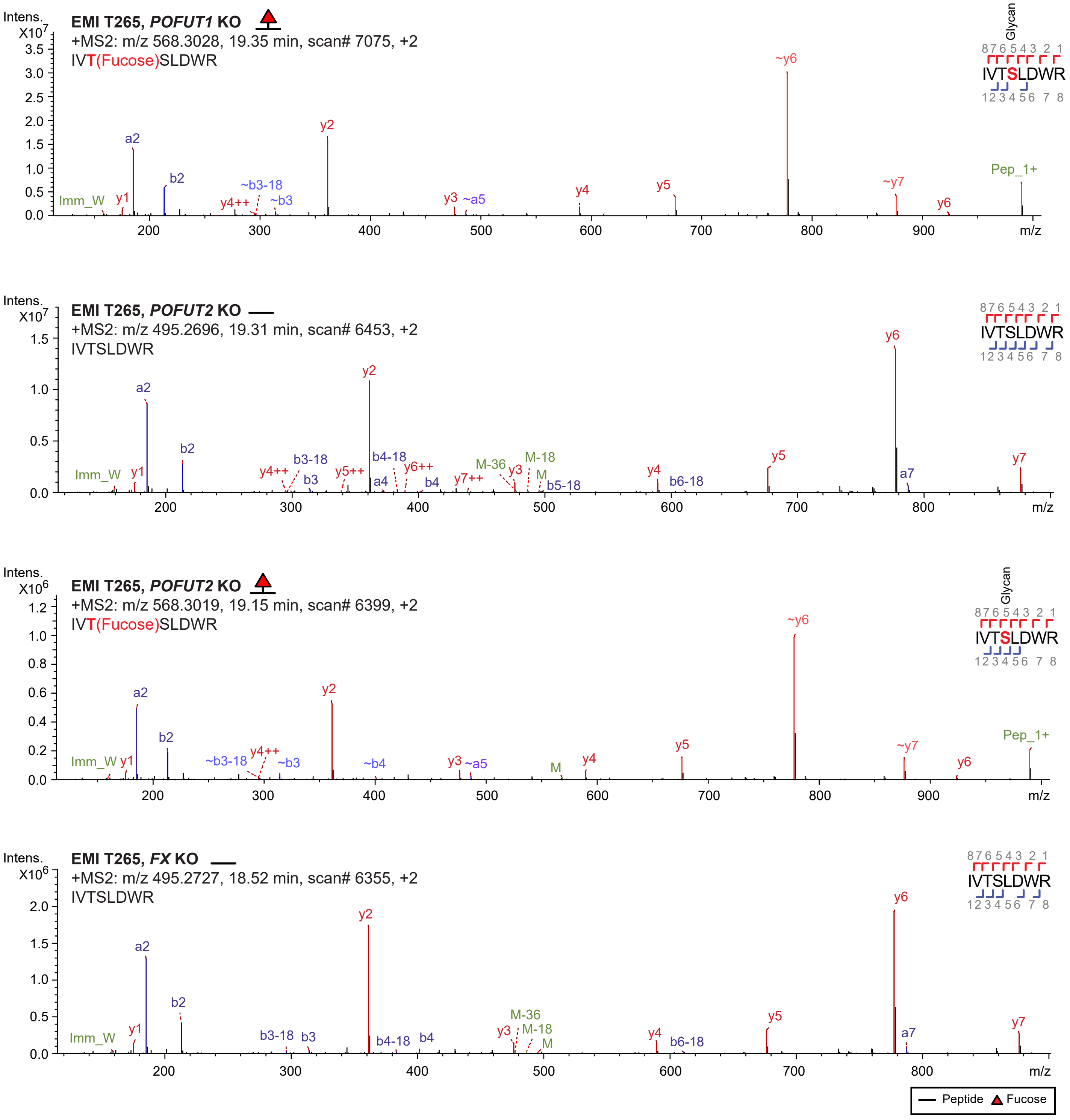

Intens. X10^7
EMI T265, POFUT1 KO
+MS2: m/z 568.3028, 19.35 min, scan# 7075, +2
IVT(Fucose)SLDWR
Glycan
IVTSLDWR
Imm_W y1 a2 b2 ~b3-18 y4++ ~b3 y2 y3 ~a5 y4 y5 ~y6 ~y7 y6 Pep_1+
m/z
Intens. X10^7
EMI T265, POFUT2 KO
+MS2: m/z 495.2696, 19.31 min, scan# 6453, +2
IVTSLDWR
Imm_W y1 a2 b2 y4++ b3-18 b3 y5++ y2 b4-18 a4 y6++ b4 y7++ M-36 y3 M-18 M b5-18 y4 b6-18 y5 y6 a7 y7
m/z
Intens. X10^6
EMI T265, POFUT2 KO
+MS2: m/z 568.3019, 19.15 min, scan# 6399, +2
IVT(Fucose)SLDWR
Glycan
IVTSLDWR
Imm_W y1 a2 b2 ~b3-18 y4++ ~b3 y2 ~b4 y3 ~a5 M y4 y5 ~y6 ~y7 y6 Pep_1+
m/z
Intens. X10^6
EMI T265, FX KO
+MS2: m/z 495.2727, 18.52 min, scan# 6355, +2
IVTSLDWR
Imm_W y1 a2 b2 b3-18 b3 y2 b4-18 b4 y3 M-36 M-18 M y4 b6-18 y5 y6 a7 y7
m/z
Peptide
Fucose

MS2 spectra for peptides used in Fig. 2b

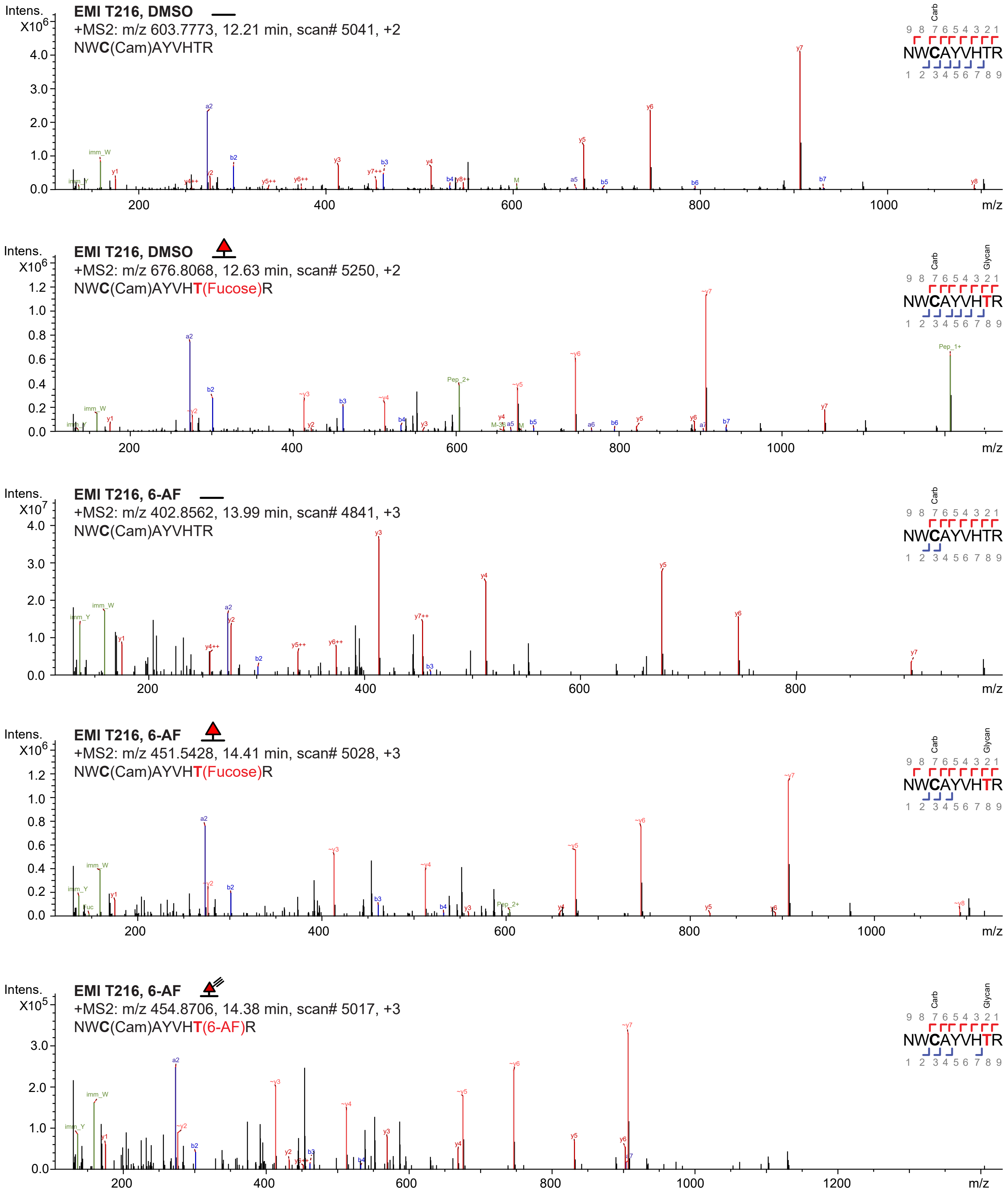

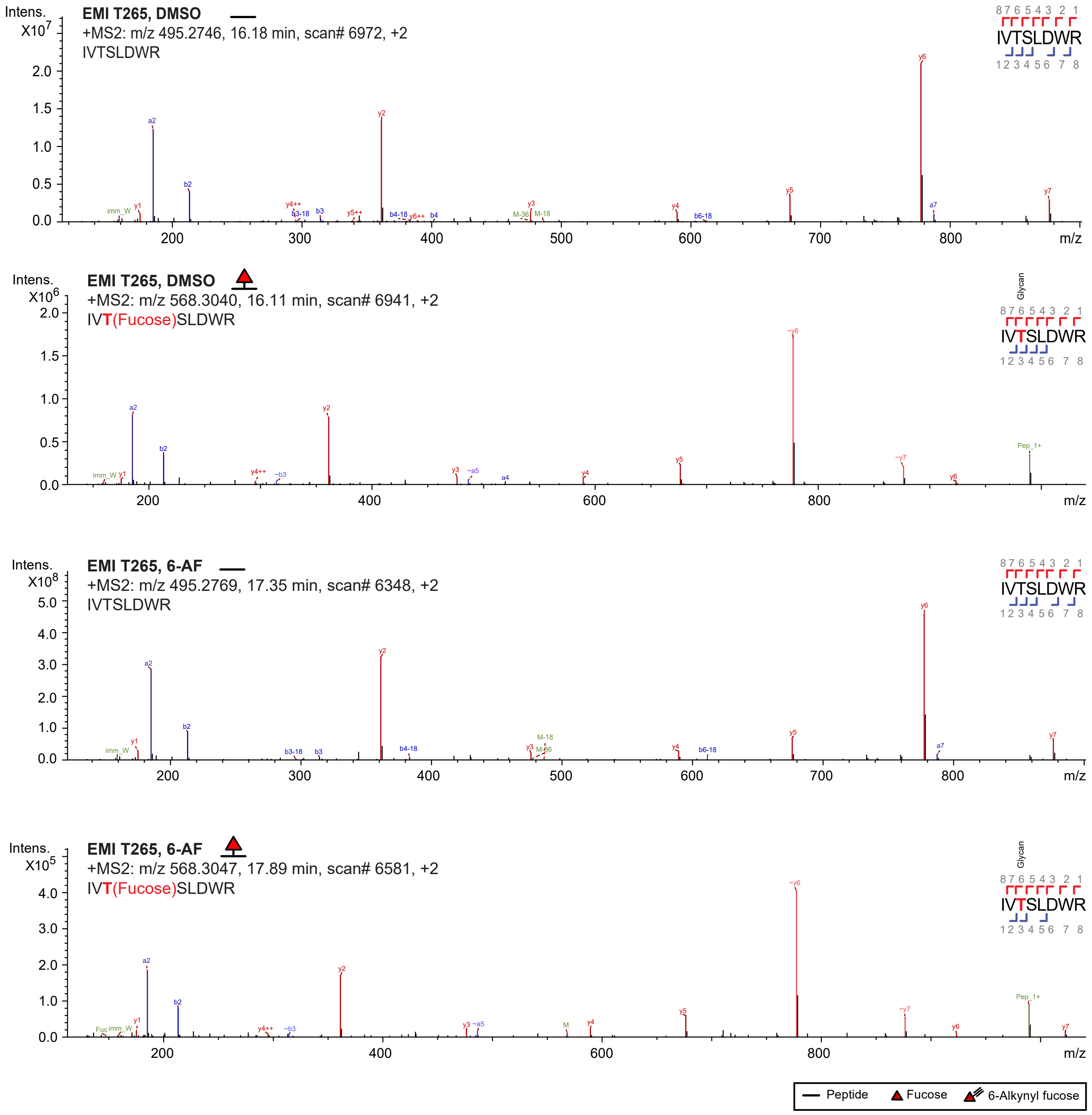

EMI T265, DMSO
+MS2: m/z 495.2746, 16.18 min, scan# 6972, +2
IVTSLDWR
EMI T265, DMSO
+MS2: m/z 568.3040, 16.11 min, scan# 6941, +2
IVT(Fucose)SLDWR
EMI T265, 6-AF
+MS2: m/z 495.2769, 17.35 min, scan# 6348, +2
IVTSLDWR
EMI T265, 6-AF
+MS2: m/z 568.3047, 17.89 min, scan# 6581, +2
IVT(Fucose)SLDWR
Intens.
m/z
Peptide
Fucose
6-Alkynyl fucose

EMI *N*-glycan, DMSO

+MS2: m/z 1036.8088, 15.27 min, scan# 6541, +3

FNPGAESVVLS**N**[HexNAc(4)Hex(3)Fuc(1)]STLK

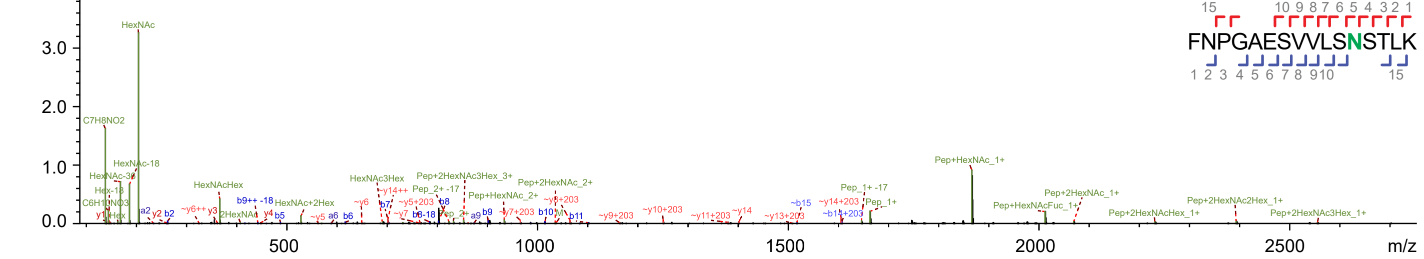

EMI *N*-glycan, DMSO

+MS2: m/z 818.3708, 15.27 min, scan# 6534, +4

FNPGAESVVLS**N**[HexNAc(4)Hex(4)Fuc(1)]STLK

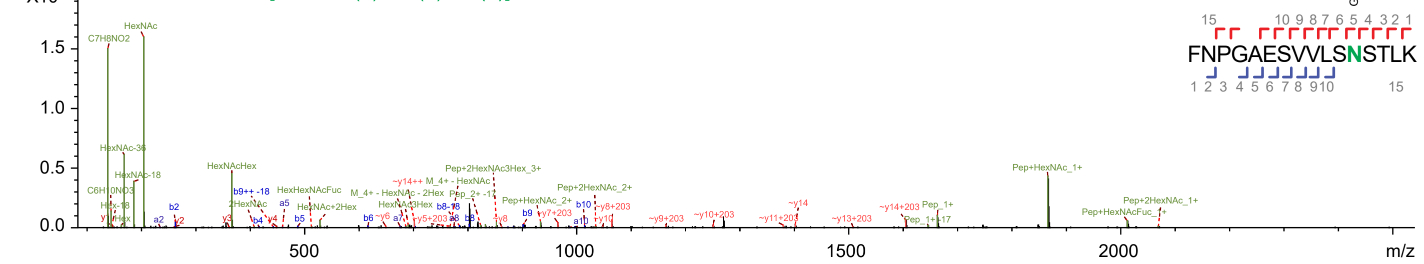

EMI *N*-glycan, DMSO

+MS2: m/z 1145.1787, 15.41 min, scan# 6609, +3

FNPGAESVVLS**N**[HexNAc(4)Hex(5)Fuc(1)]STLK

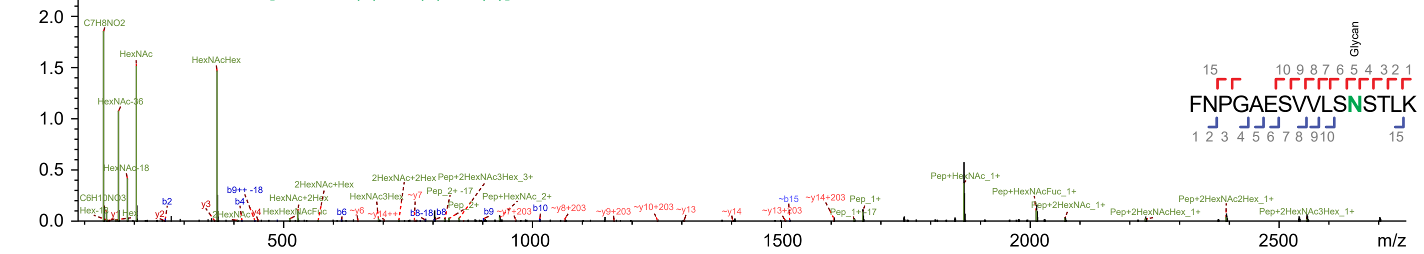

EMI *N*-glycan, DMSO

+MS2: m/z 1242.2112, 16.75 min, scan# 7233, +3

FNPGAESVVLS**N**[HexNAc(4)Hex(5)Fuc(1)NeuAc(1)]STLK

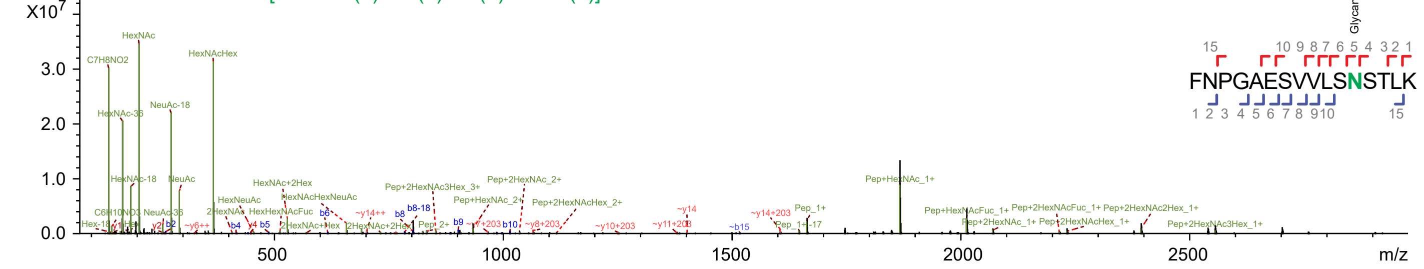

EMI *N*-glycan, DMSO

+MS2: m/z 1158.8535, 15.20 min, scan# 6506, +3

FNPGAESVVLS**N**[HexNAc(5)Hex(4)Fuc(1)]STLK

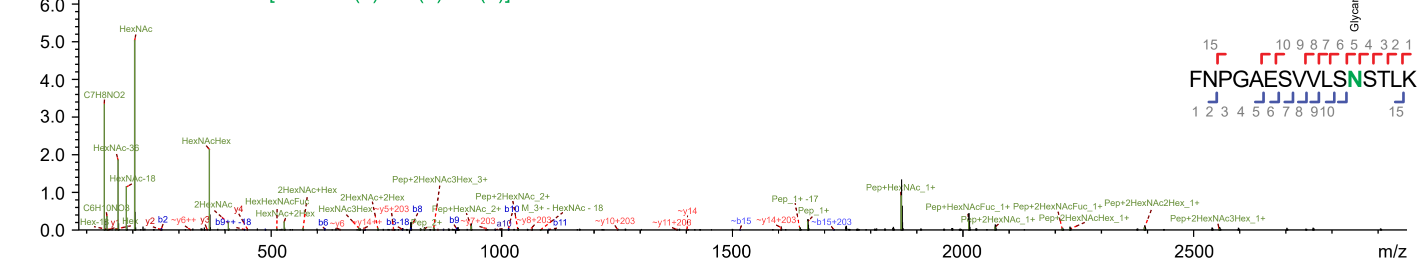

EMI *N*-glycan, DMSO

+MS2: m/z 909.9052, 15.13 min, scan# 6470, +4

FNPGAESVVLS**N**[HexNAc(5)Hex(5)Fuc(1)]STLK

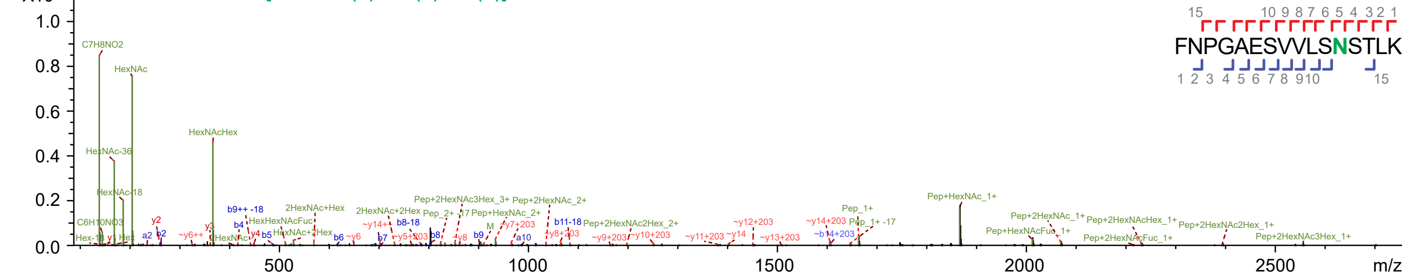

EMI *N*-glycan, DMSO

+MS2: m/z 1266.8883, 14.94 min, scan# 6374, +3

FNPGAESVVLS**N**[HexNAc(5)Hex(6)Fuc(1)]STLK

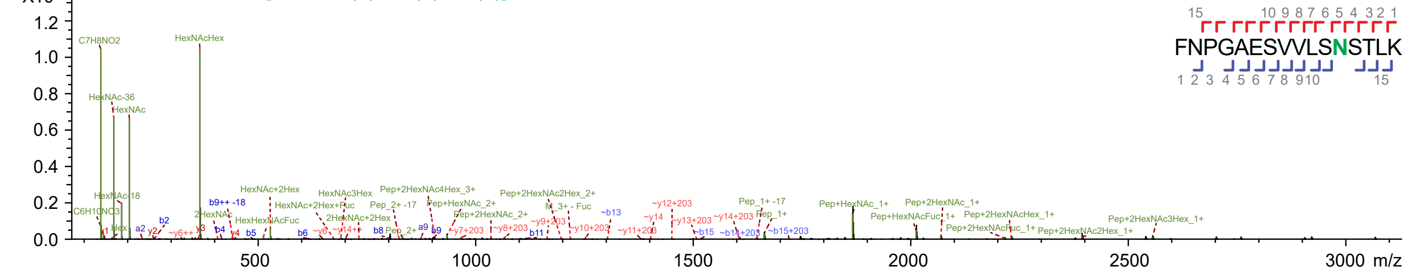

EMI *N*-glycan, DMSO

+MS2: m/z 1280.5651, 14.98 min, scan# 6394, +3

FNPGAESVVLS**N**[HexNAc(6)Hex(5)Fuc(1)]STLK

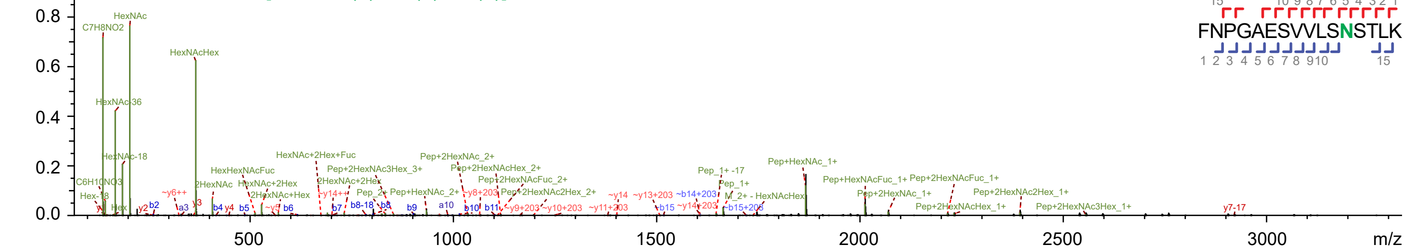

EMI *N*-glycan, 6-AF
+MS2: m/z 1042.1433, 16.89 min, scan# 6147, +3
FNPGAESVVLS**N**[HexNAc(4)Hex(4)]STLK

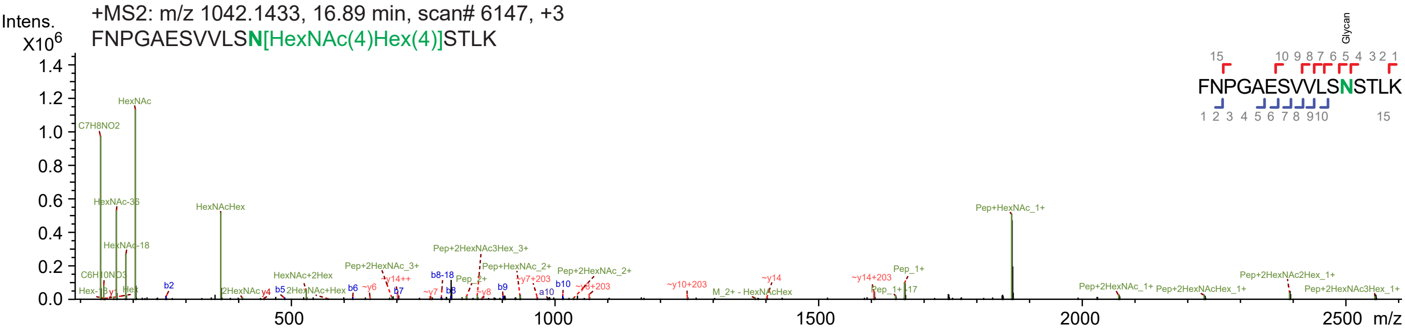

EMI *N*-glycan, 6-AF
+MS2: m/z 1096.1614, 16.90 min, scan# 6150, +3
FNPGAESVVLS**N**[HexNAc(4)Hex(5)]STLK

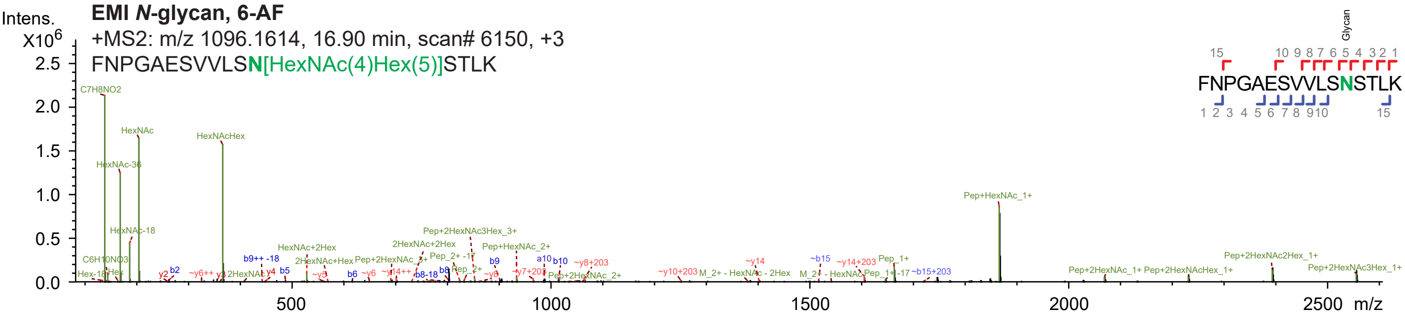

EMI *N*-glycan, 6-AF
+MS2: m/z 1193.5297, 18.36 min, scan# 6788, +3
FNPGAESVVLS**N**[HexNAc(4)Hex(5)NeuAc(1)]STLK

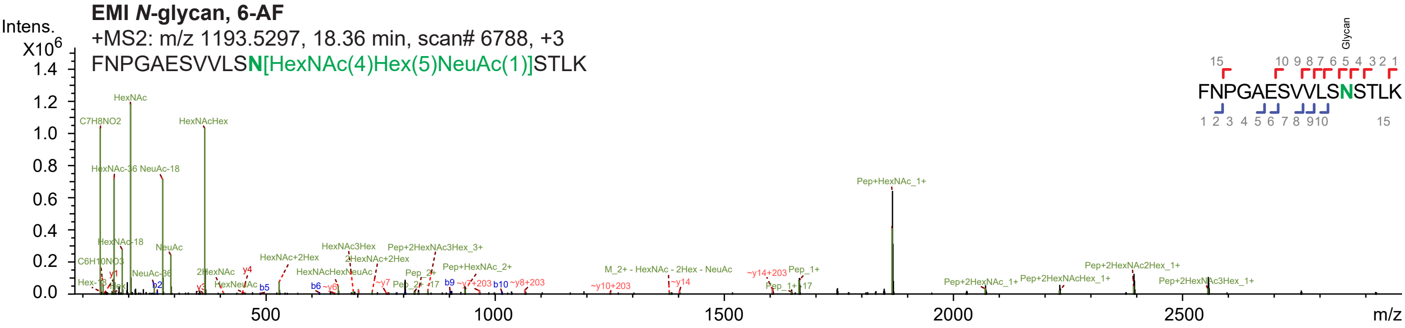

EMI *N*-glycan, 6-AF
+MS2: m/z 1055.8201, 16.95 min, scan# 6174, +3
FNPGAESVVLS**N**[HexNAc(5)Hex(3)]STLK

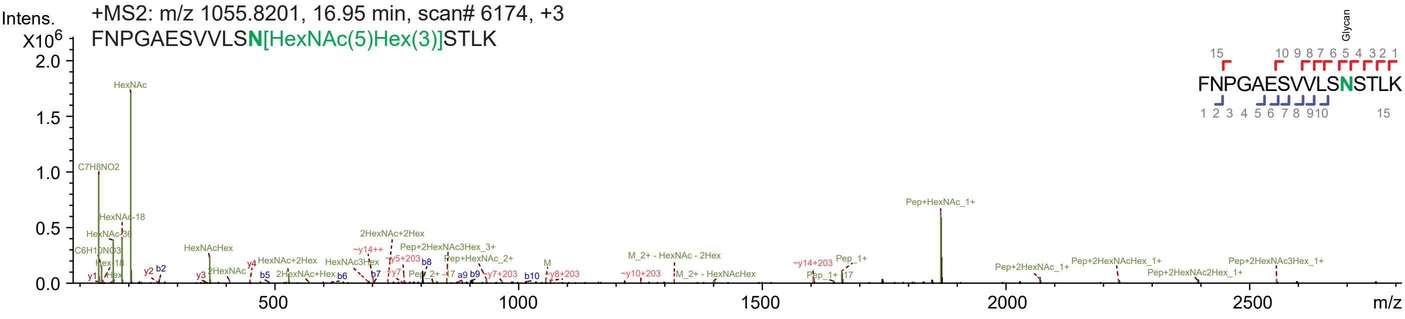

EMI *N*-glycan, 6-AF
+MS2: m/z 1110.1716, 16.86 min, scan# 6130, +3
FNPGAESVVLS**N**[HexNAc(5)Hex(4)]STLK

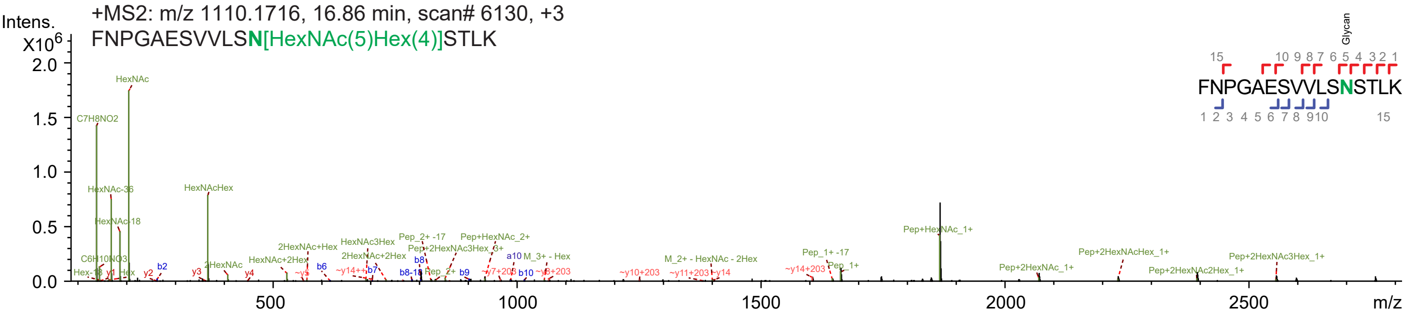

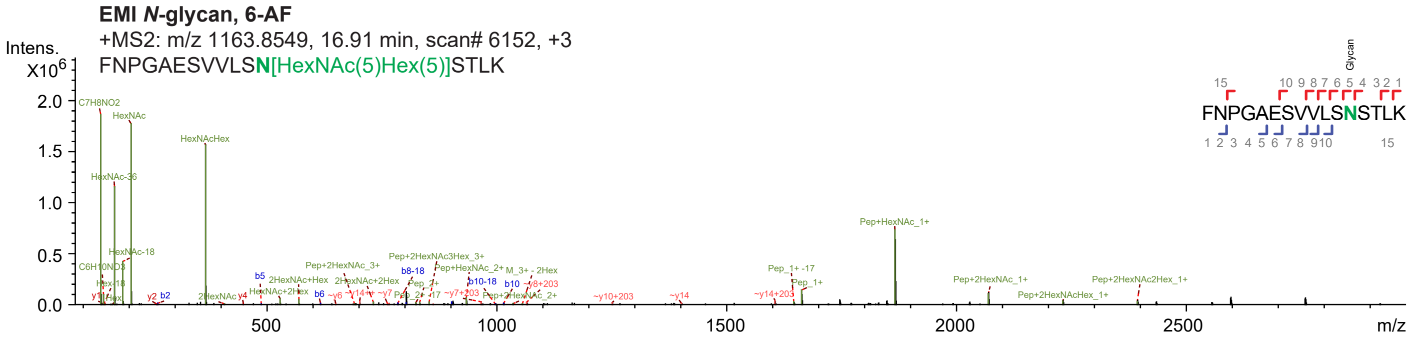
EMI N-glycan, 6-AF
+MS2: m/z 1163.8549, 16.91 min, scan# 6152, +3
FNPGAESVVLSN[HexNAc(5)Hex(5)]STLK
Intens.
X10^6
m/z
Glycan
FNPGAESVVLSNSTLK

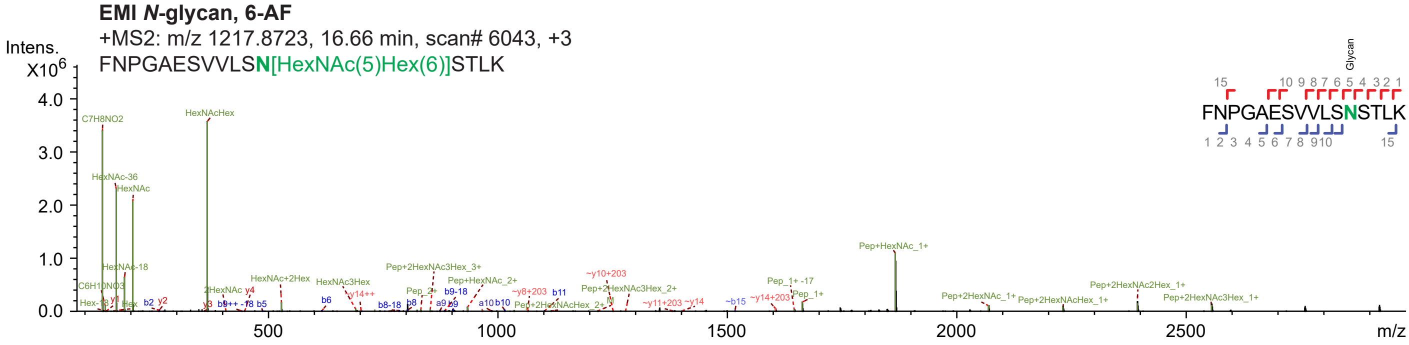
EMI N-glycan, 6-AF
+MS2: m/z 1217.8723, 16.66 min, scan# 6043, +3
FNPGAESVVLSN[HexNAc(5)Hex(6)]STLK
Intens.
X10^6
m/z
Glycan
FNPGAESVVLSNSTLK

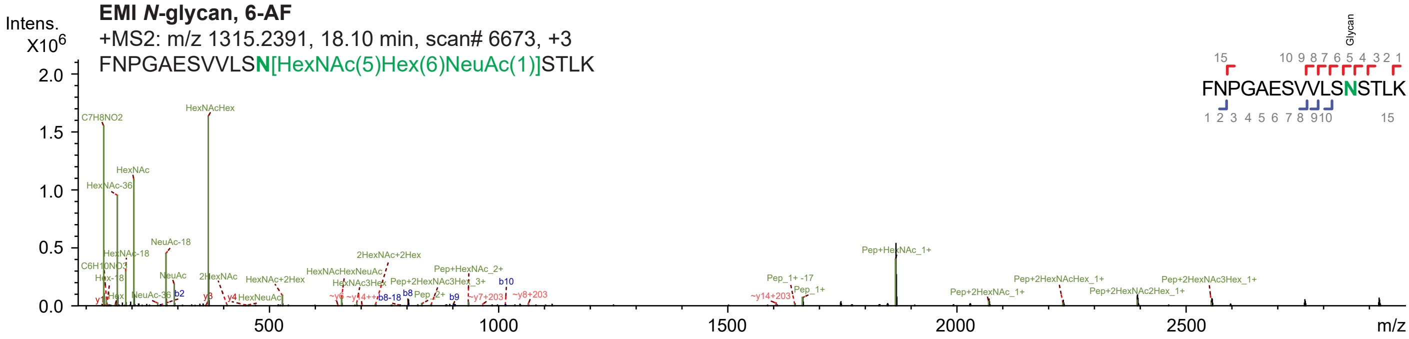
EMI N-glycan, 6-AF
+MS2: m/z 1315.2391, 18.10 min, scan# 6673, +3
FNPGAESVVLSN[HexNAc(5)Hex(6)NeuAc(1)]STLK
Intens.
X10^6
m/z
Glycan
FNPGAESVVLSNSTLK

MS2 spectra for peptides used in Fig. 6b

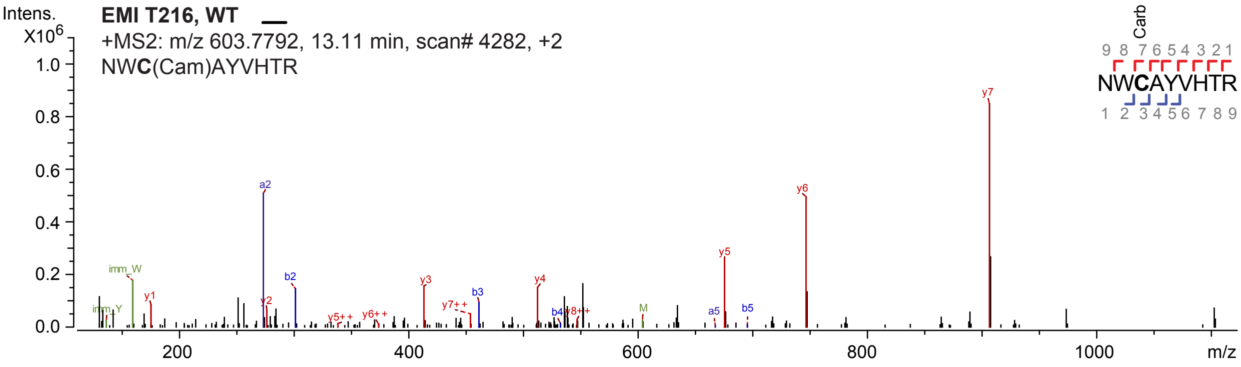

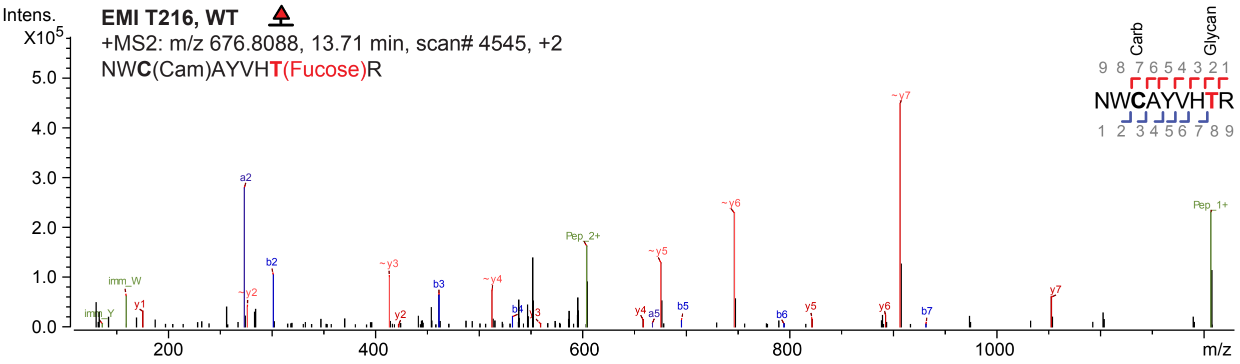

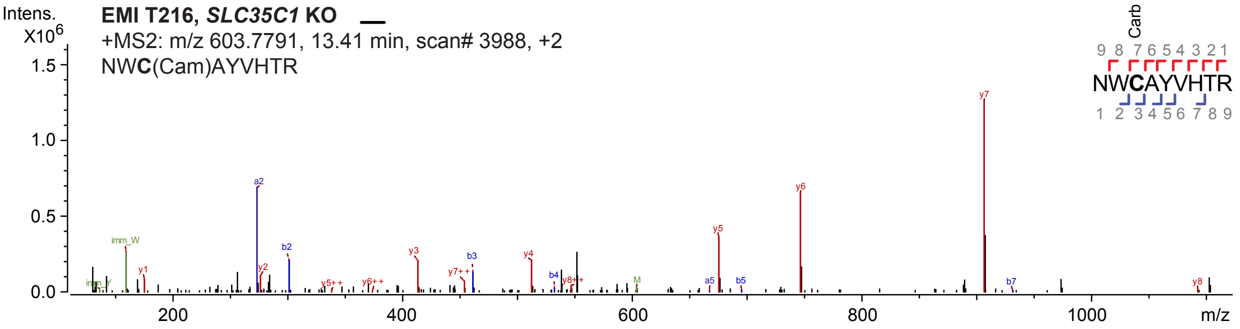

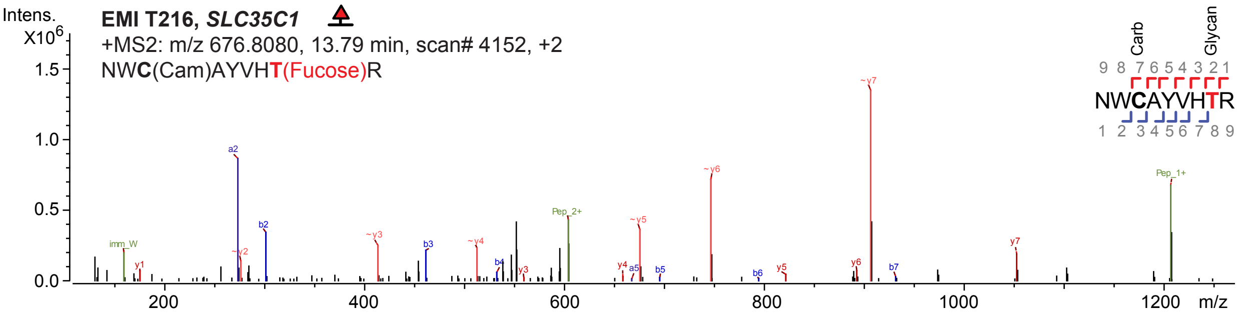

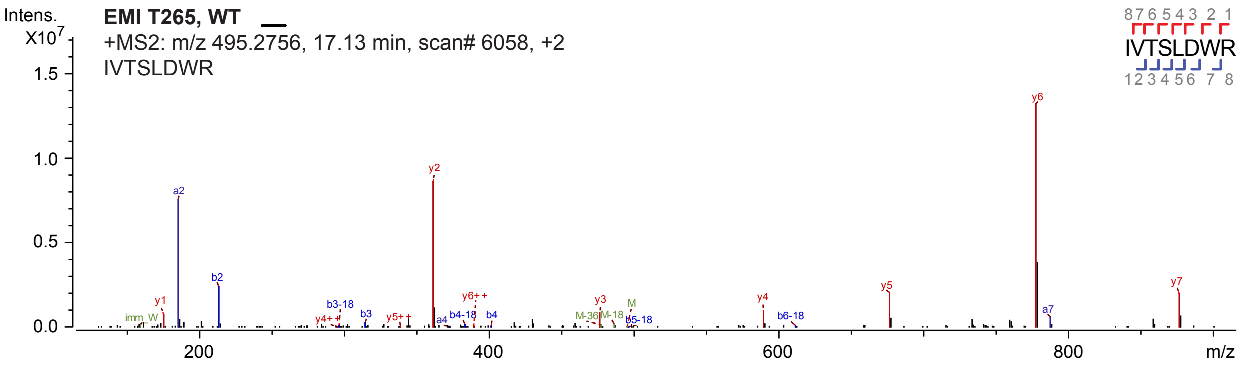
Intens.
X10^7
EMI T265, WT
+MS2: m/z 495.2756, 17.13 min, scan# 6058, +2
IVTSLDWR
m/z

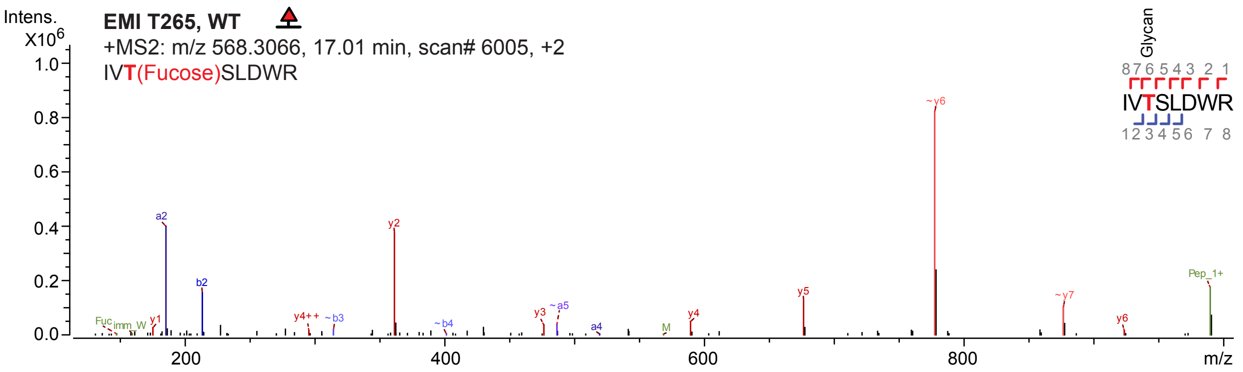
Intens.
X10^6
EMI T265, WT
+MS2: m/z 568.3066, 17.01 min, scan# 6005, +2
IVT(Fucose)SLDWR
Glycan
m/z

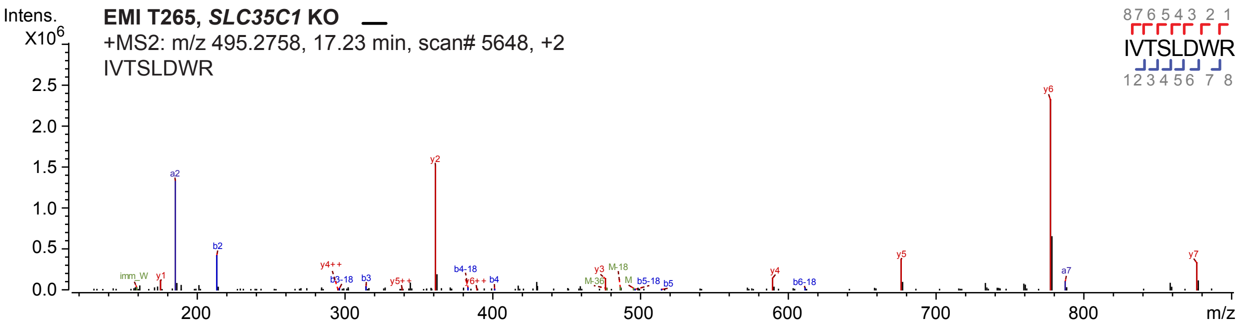
Intens.
X10^6
EMI T265, SLC35C1 KO
+MS2: m/z 495.2758, 17.23 min, scan# 5648, +2
IVTSLDWR
m/z

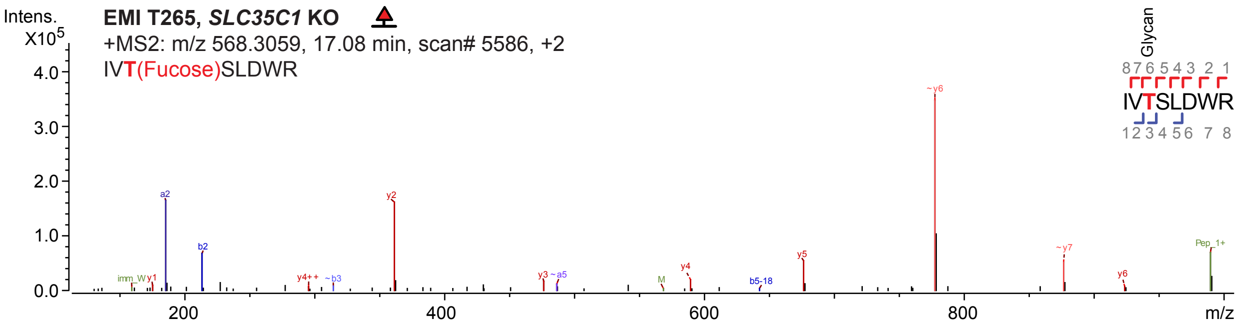
Intens.
X10^5
EMI T265, SLC35C1 KO
+MS2: m/z 568.3059, 17.08 min, scan# 5586, +2
IVT(Fucose)SLDWR
Glycan
m/z

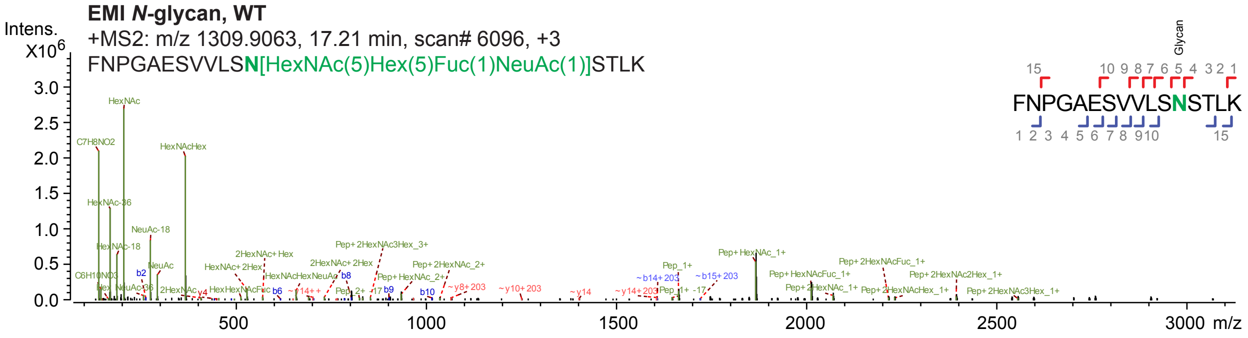
EMI N-glycan, WT
+MS2: m/z 1309.9063, 17.21 min, scan# 6096, +3
FNPGAESVVLSN[HexNAc(5)Hex(5)Fuc(1)NeuAc(1)]STLK
Intens.
X10^6
m/z

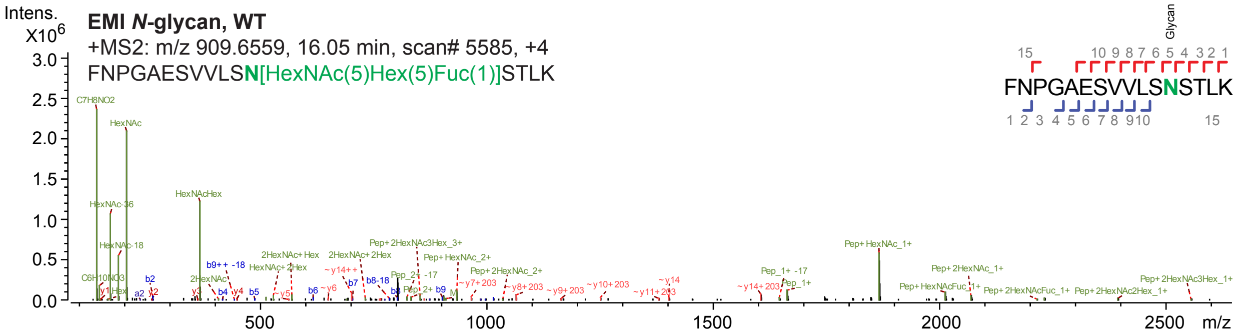
EMI N-glycan, WT
+MS2: m/z 909.6559, 16.05 min, scan# 5585, +4
FNPGAESVVLSN[HexNAc(5)Hex(5)Fuc(1)]STLK
Intens.
X10^6
m/z

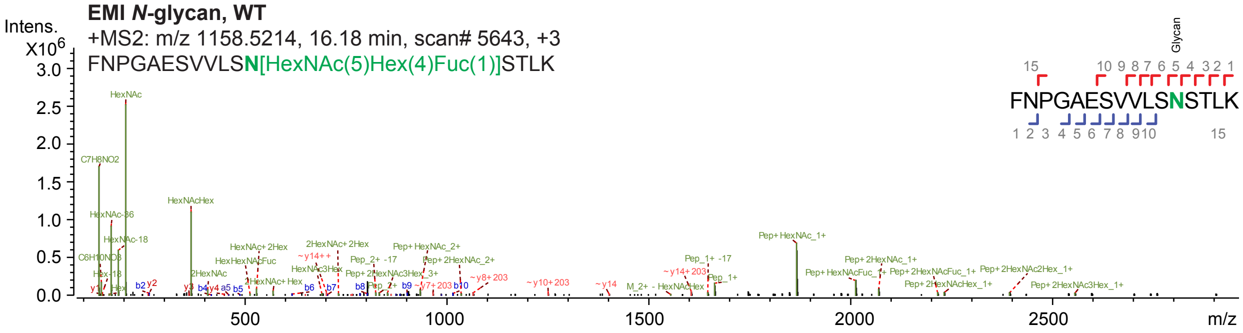
EMI N-glycan, WT
+MS2: m/z 1158.5214, 16.18 min, scan# 5643, +3
FNPGAESVVLSN[HexNAc(5)Hex(4)Fuc(1)]STLK
Intens.
X10^6
m/z

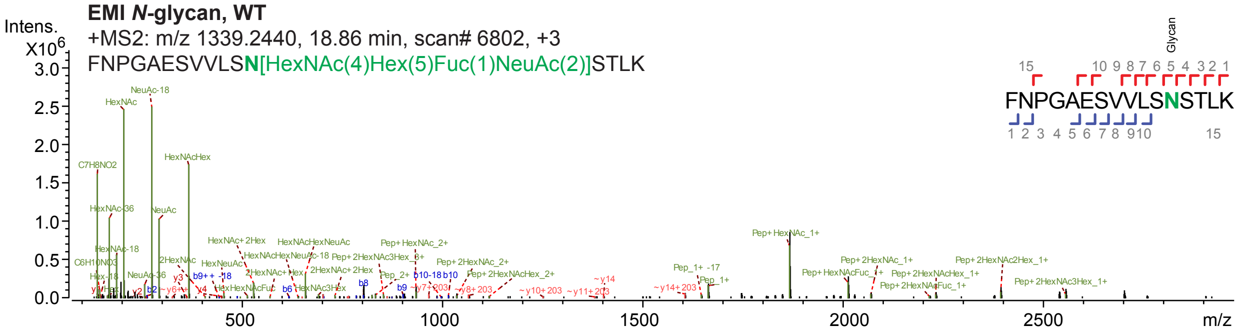
EMI N-glycan, WT
+MS2: m/z 1339.2440, 18.86 min, scan# 6802, +3
FNPGAESVVLSN[HexNAc(4)Hex(5)Fuc(1)NeuAc(2)]STLK
Intens.
X10^6
m/z

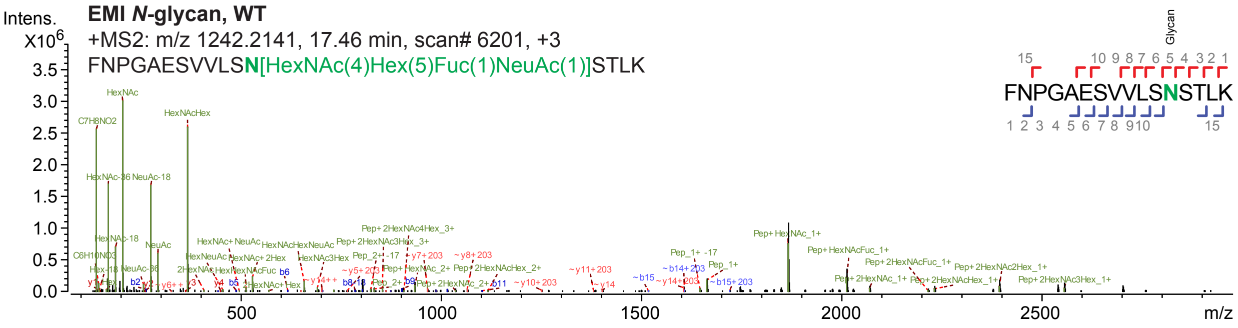
Intens.
x10^6
EMI N-glycan, WT
+MS2: m/z 1242.2141, 17.46 min, scan# 6201, +3
FNPGAESVVLSN[HexNAc(4)Hex(5)Fuc(1)NeuAc(1)]STLK
FNPGAESVVLSNSTLK
Glycan
HexNAc
HexNAcHex
C7H8NO2
NeuAc-18
NeuAc
Pep+HexNAc_1+
Pep+HexNAcFuc_1+
Pep+2HexNAcFuc_1+
Pep+2HexNAc2Hex_1+
Pep+2HexNAc3Hex_1+
m/z

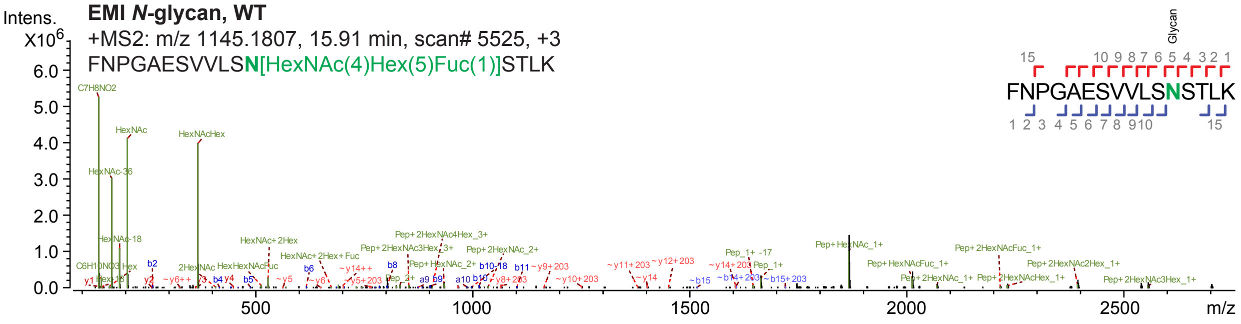
Intens.
x10^6
EMI N-glycan, WT
+MS2: m/z 1145.1807, 15.91 min, scan# 5525, +3
FNPGAESVVLSN[HexNAc(4)Hex(5)Fuc(1)]STLK
FNPGAESVVLSNSTLK
Glycan
C7H8NO2
HexNAc
HexNAcHex
HexNAc-36
HexNAc-18
Pep+HexNAc_1+
Pep+2HexNAcFuc_1+
Pep+2HexNAc2Hex_1+
Pep+2HexNAc3Hex_1+
m/z

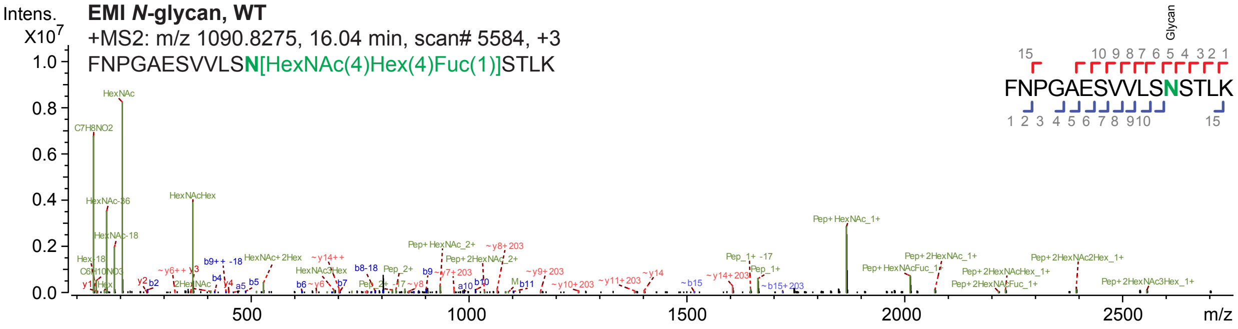
Intens.
x10^7
EMI N-glycan, WT
+MS2: m/z 1090.8275, 16.04 min, scan# 5584, +3
FNPGAESVVLSN[HexNAc(4)Hex(4)Fuc(1)]STLK
FNPGAESVVLSNSTLK
Glycan
HexNAc
C7H8NO2
HexNAc-36
HexNAc-18
HexNAcHex
Pep+HexNAc_1+
Pep+2HexNAc_1+
Pep+2HexNAc2Hex_1+
Pep+2HexNAc3Hex_1+
m/z

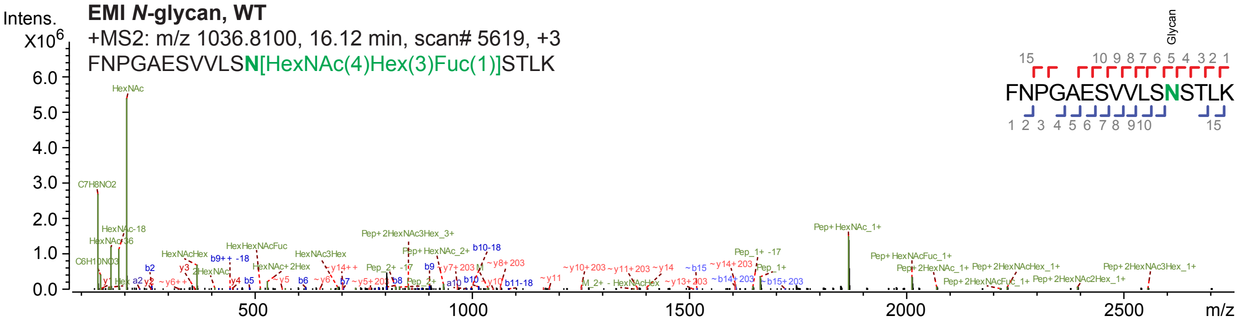
Intens.
x10^6
EMI N-glycan, WT
+MS2: m/z 1036.8100, 16.12 min, scan# 5619, +3
FNPGAESVVLSN[HexNAc(4)Hex(3)Fuc(1)]STLK
FNPGAESVVLSNSTLK
Glycan
HexNAc
C7H8NO2
HexNAc-18
HexNAc-36
Pep+HexNAc_1+
Pep+HexNAcFuc_1+
Pep+2HexNAcHex_1+
Pep+2HexNAc3Hex_1+
m/z

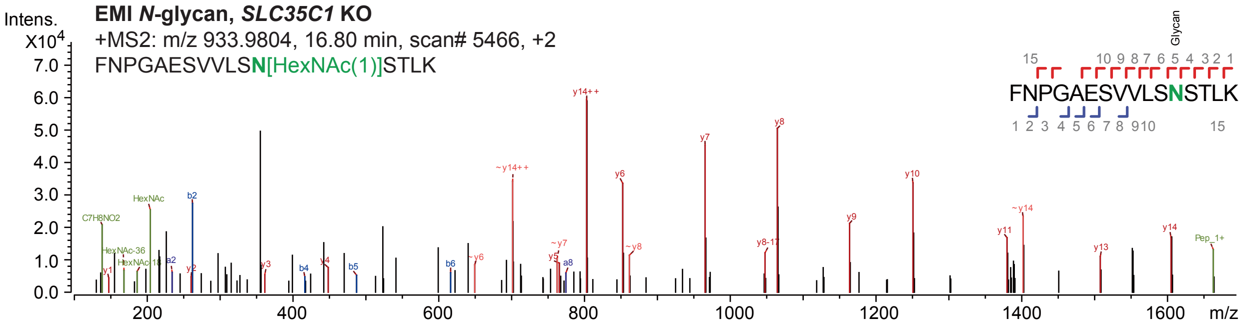
EMI N-glycan, SLC35C1 KO
+MS2: m/z 933.9804, 16.80 min, scan# 5466, +2
FNPGAESVVLSN[HexNAc(1)]STLK
Intens.
X10^4
m/z

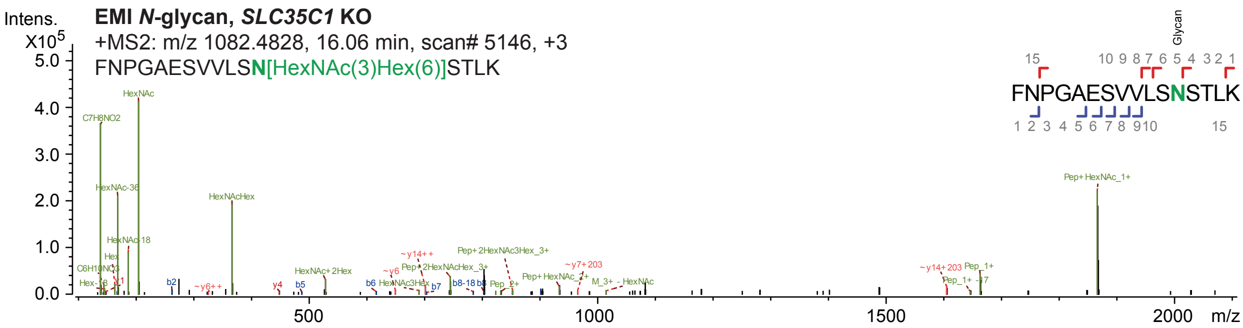
EMI N-glycan, SLC35C1 KO
+MS2: m/z 1082.4828, 16.06 min, scan# 5146, +3
FNPGAESVVLSN[HexNAc(3)Hex(6)]STLK
Intens.
X10^5
m/z

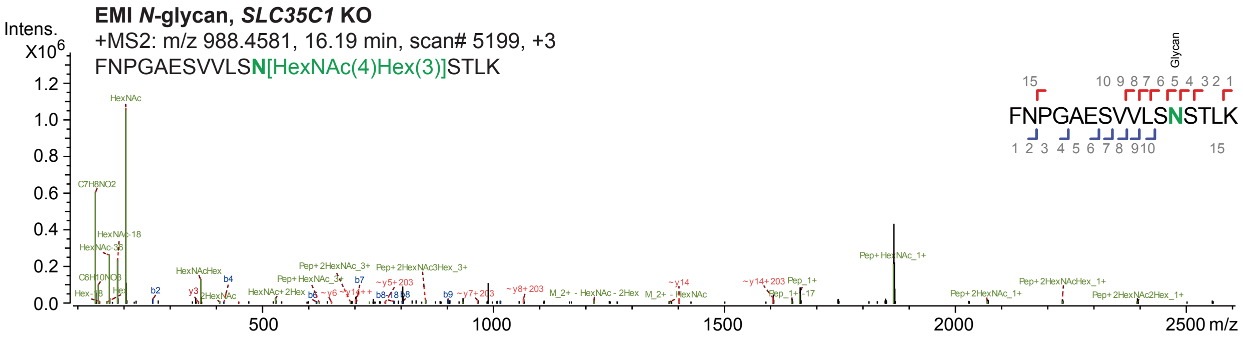
EMI N-glycan, SLC35C1 KO
+MS2: m/z 988.4581, 16.19 min, scan# 5199, +3
FNPGAESVVLSN[HexNAc(4)Hex(3)]STLK
Intens.
X10^6
m/z

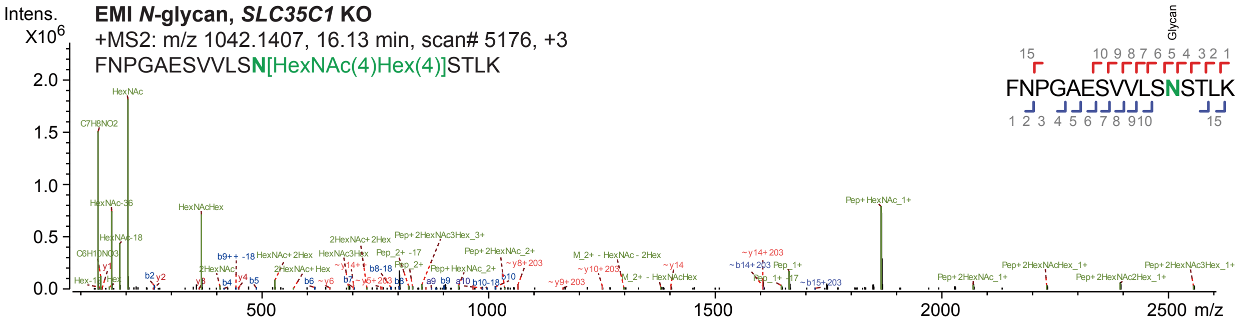
EMI N-glycan, SLC35C1 KO
+MS2: m/z 1042.1407, 16.13 min, scan# 5176, +3
FNPGAESVVLSN[HexNAc(4)Hex(4)]STLK
Intens.
X10^6
m/z

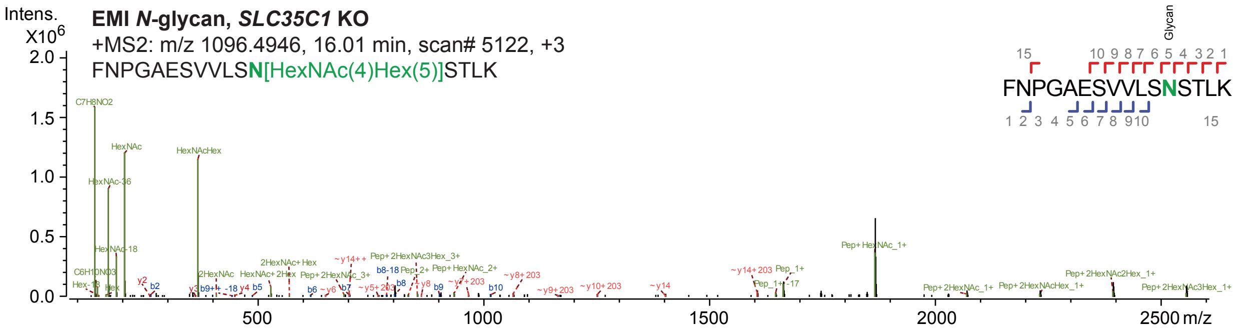
EMI N-glycan, SLC35C1 KO
+MS2: m/z 1096.4946, 16.01 min, scan# 5122, +3
FNPGAESVVLSN[HexNAc(4)Hex(5)]STLK
Intens.
X10^6
m/z

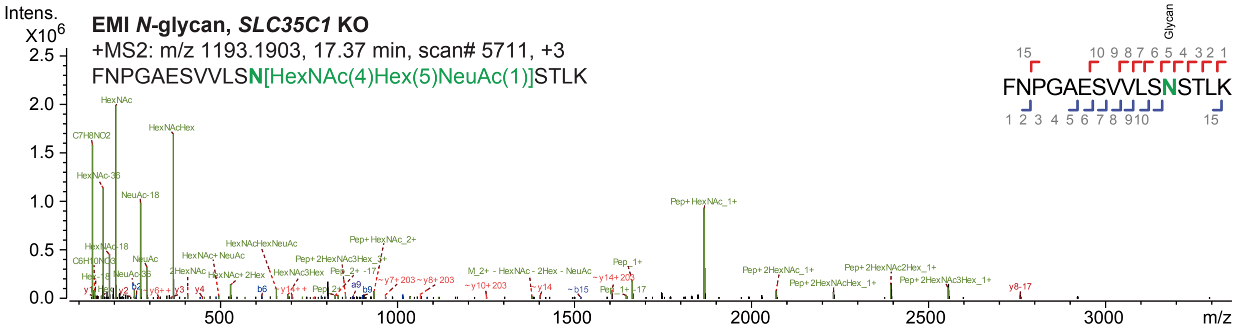
EMI N-glycan, SLC35C1 KO
+MS2: m/z 1193.1903, 17.37 min, scan# 5711, +3
FNPGAESVVLSN[HexNAc(4)Hex(5)NeuAc(1)]STLK
Intens.
X10^6
m/z

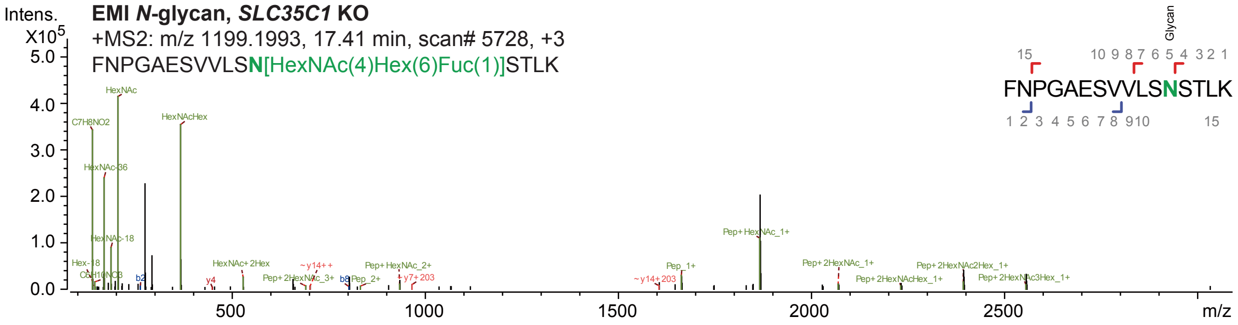
EMI N-glycan, SLC35C1 KO
+MS2: m/z 1199.1993, 17.41 min, scan# 5728, +3
FNPGAESVVLSN[HexNAc(4)Hex(6)Fuc(1)]STLK
Intens.
X10^5
m/z

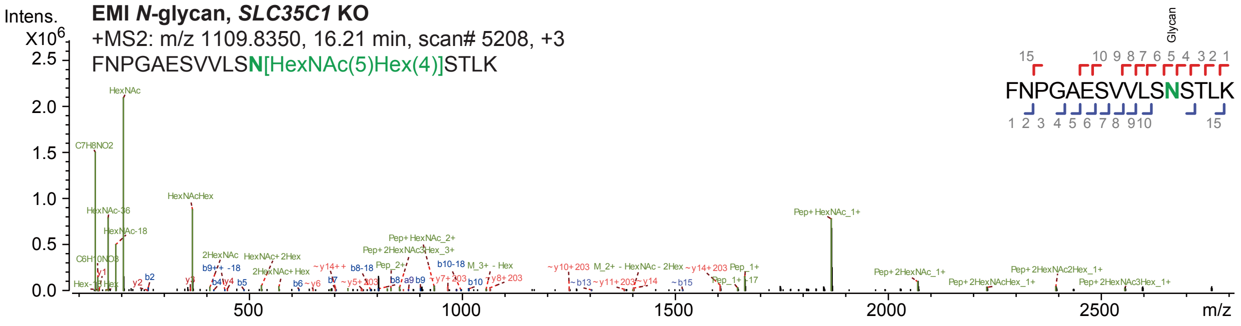
EMI N-glycan, SLC35C1 KO
+MS2: m/z 1109.8350, 16.21 min, scan# 5208, +3
FNPGAESVVLSN[HexNAc(5)Hex(4)]STLK
Intens.
X10^6
m/z

MS2 spectra for peptides used in Extended Data Fig. 7

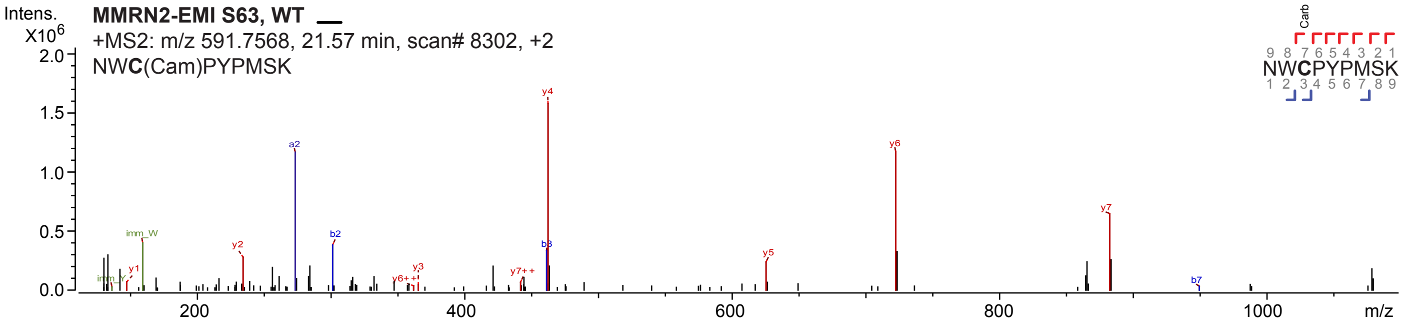

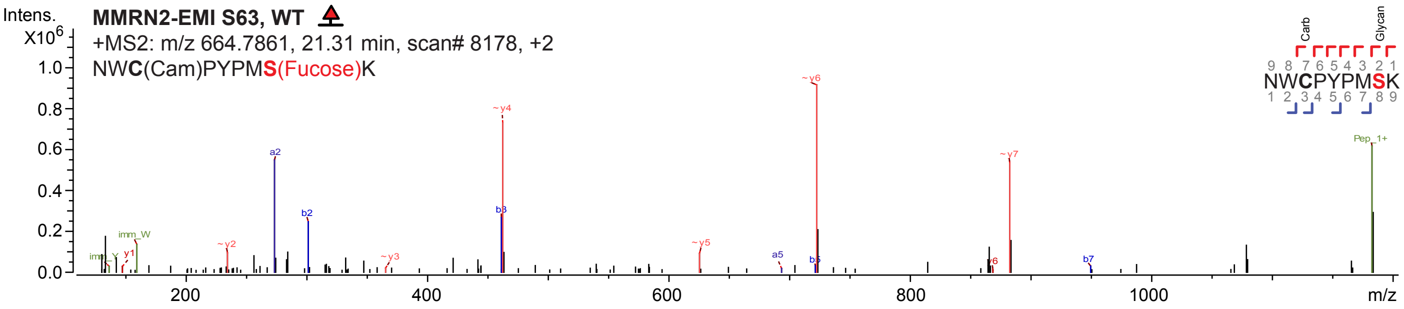

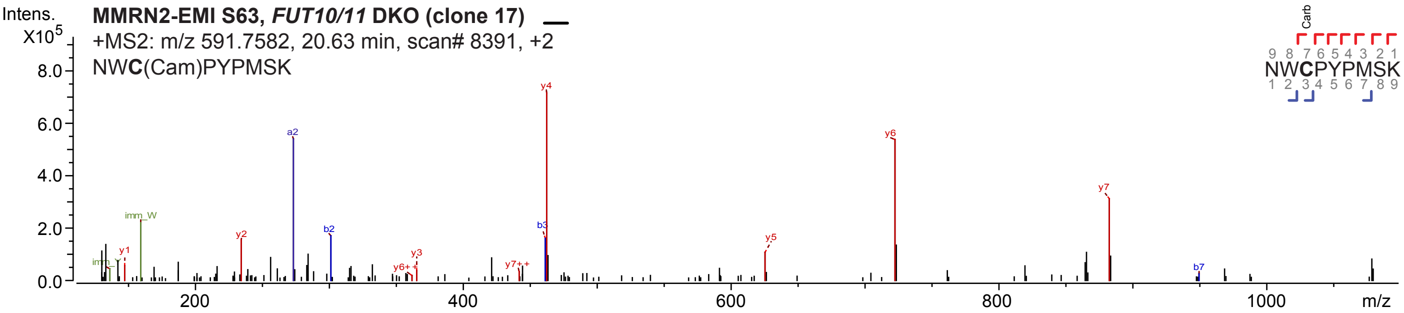

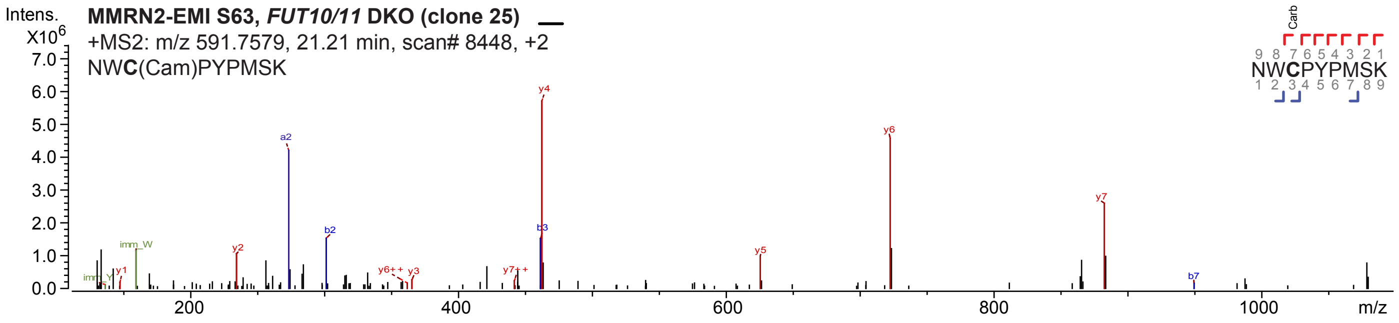

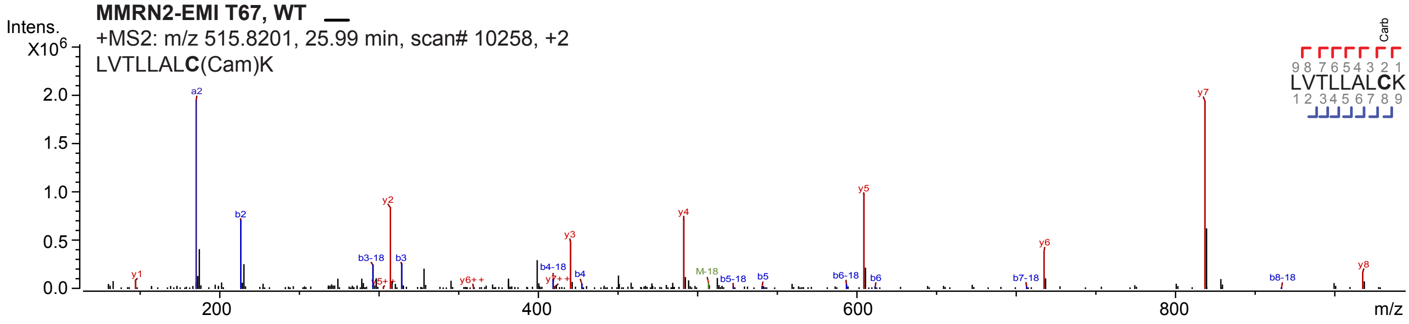
MMRN2-EMI T67, WT
+MS2: m/z 515.8201, 25.99 min, scan# 10258, +2
LVTLLALC(Cam)K
Intens.
X10^6
m/z

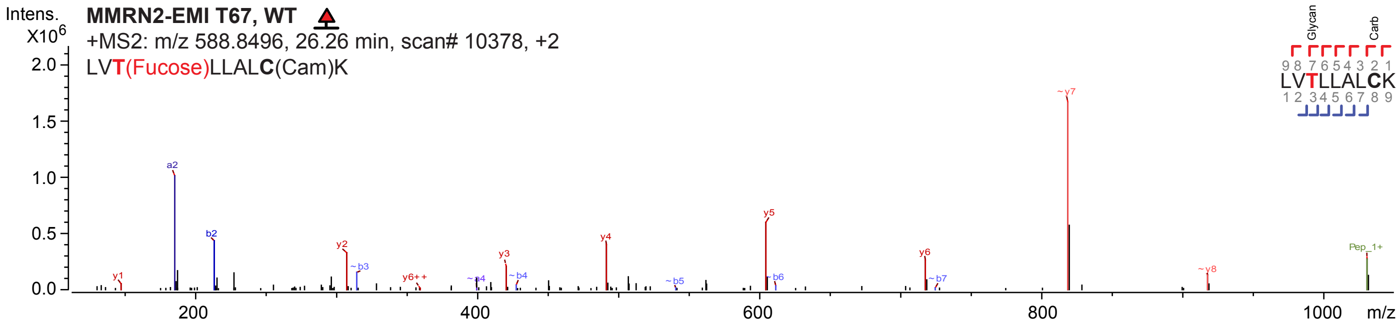
MMRN2-EMI T67, WT
+MS2: m/z 588.8496, 26.26 min, scan# 10378, +2
LVT(Fucose)LLALC(Cam)K
Intens.
X10^6
m/z

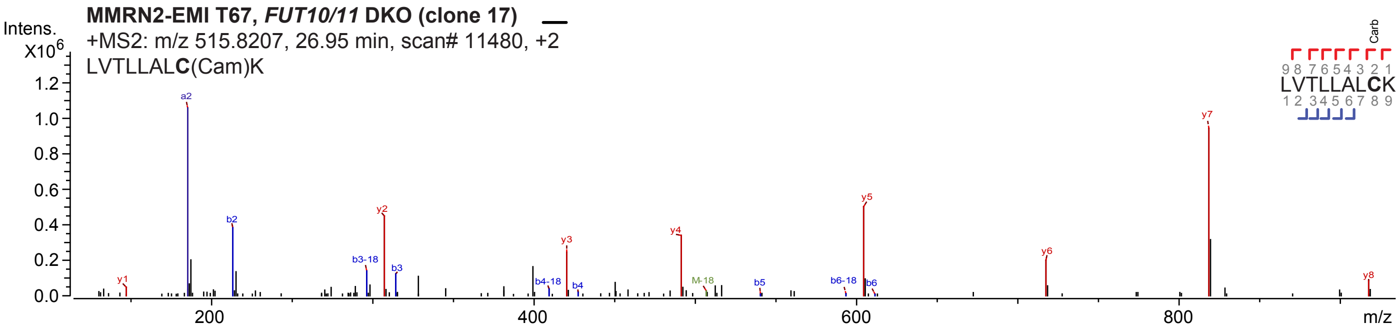
MMRN2-EMI T67, FUT10/11 DKO (clone 17)
+MS2: m/z 515.8207, 26.95 min, scan# 11480, +2
LVTLLALC(Cam)K
Intens.
X10^6
m/z

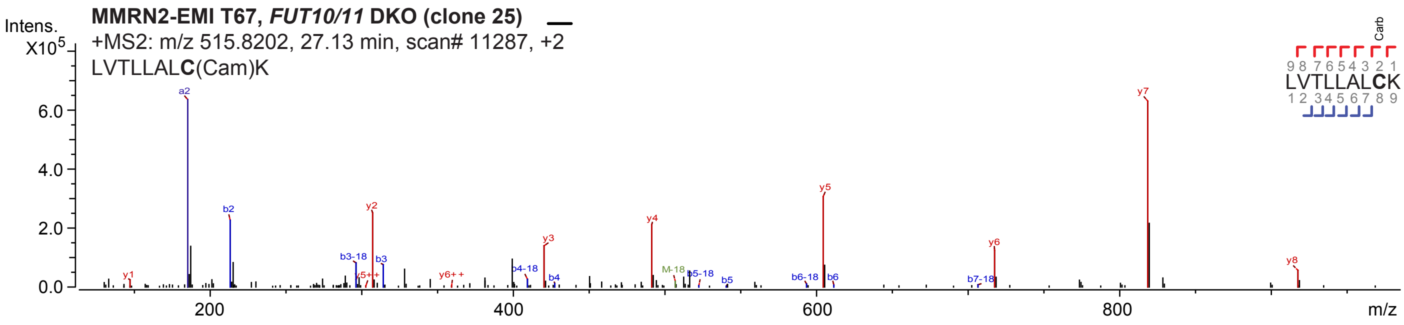
MMRN2-EMI T67, FUT10/11 DKO (clone 25)
+MS2: m/z 515.8202, 27.13 min, scan# 11287, +2
LVTLLALC(Cam)K
Intens.
X10^5
m/z

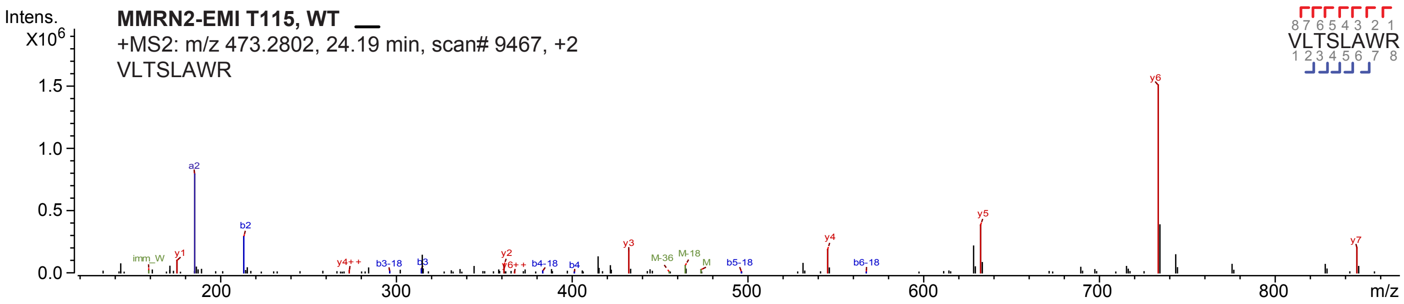
Intens.
X10^6
MMRN2-EMI T115, WT
+MS2: m/z 473.2802, 24.19 min, scan# 9467, +2
VLTSLAWR
m/z

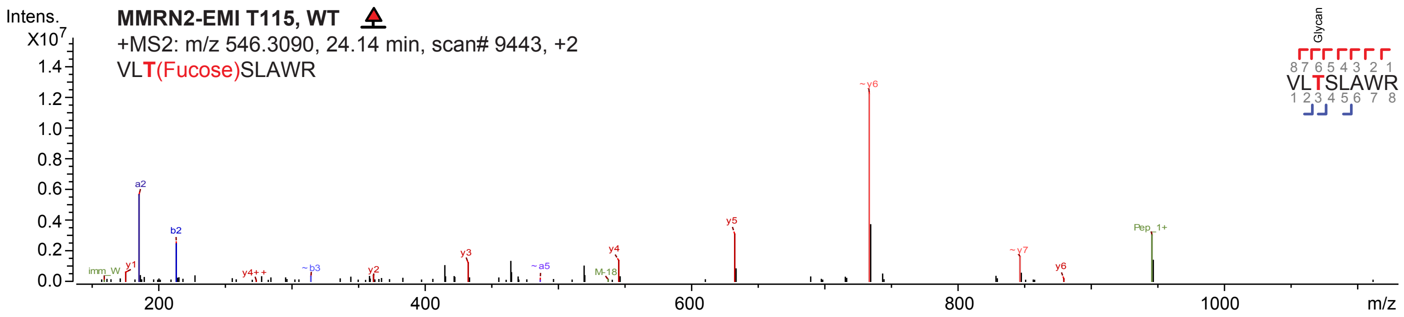
Intens.
X10^7
MMRN2-EMI T115, WT
+MS2: m/z 546.3090, 24.14 min, scan# 9443, +2
VLT(Fucose)SLAWR
Glycan
m/z

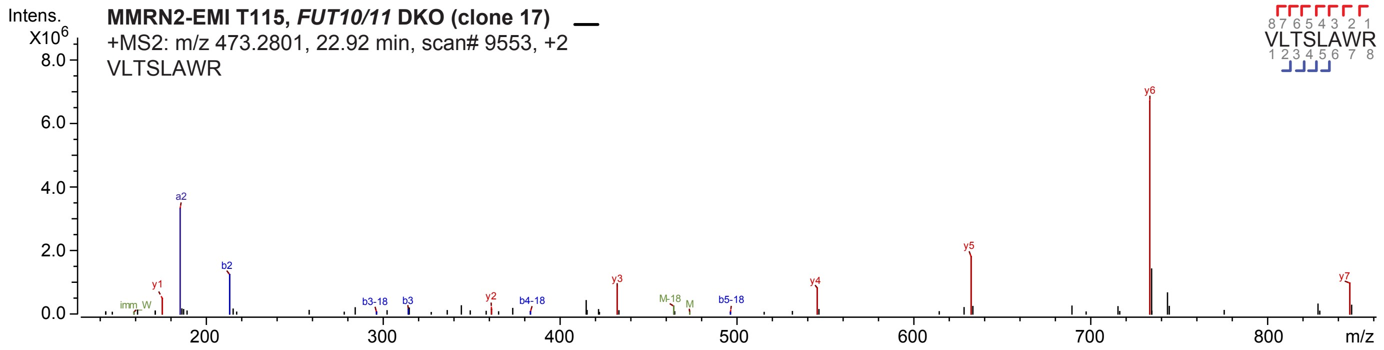
Intens.
X10^6
MMRN2-EMI T115, FUT10/11 DKO (clone 17)
+MS2: m/z 473.2801, 22.92 min, scan# 9553, +2
VLTSLAWR
m/z

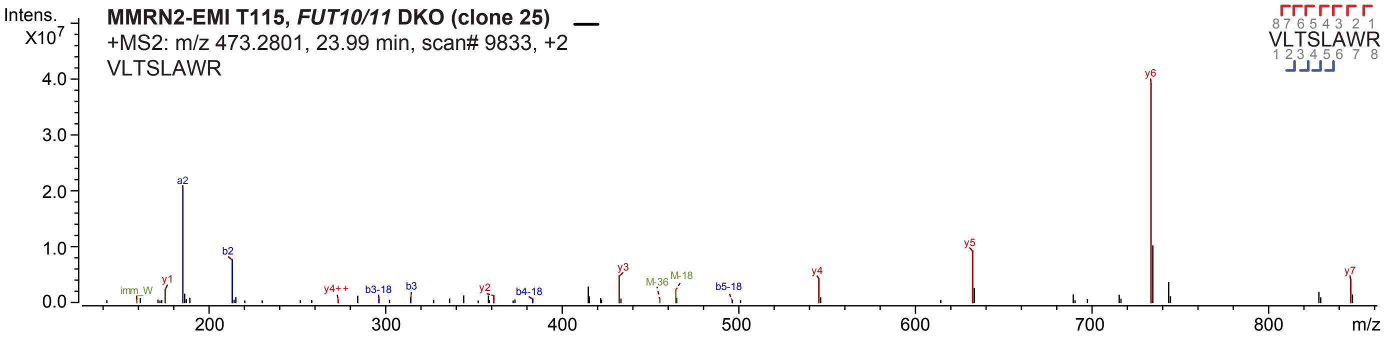
Intens.
X10^7
MMRN2-EMI T115, FUT10/11 DKO (clone 25)
+MS2: m/z 473.2801, 23.99 min, scan# 9833, +2
VLTSLAWR
m/z

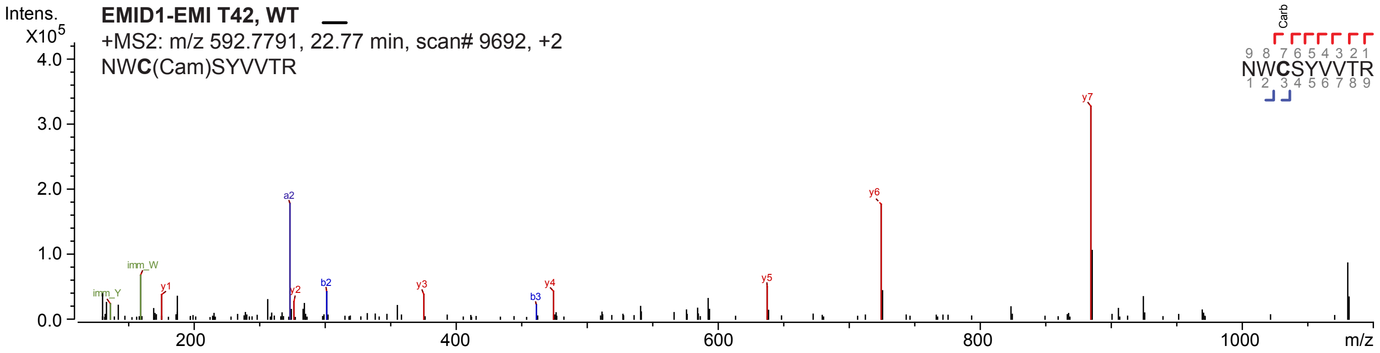
EMID1-EMI T42, WT
+MS2: m/z 592.7791, 22.77 min, scan# 9692, +2
NWC(Cam)SYVVTR
Intens.
X10^5
m/z
NWCSYVVTR
Carb

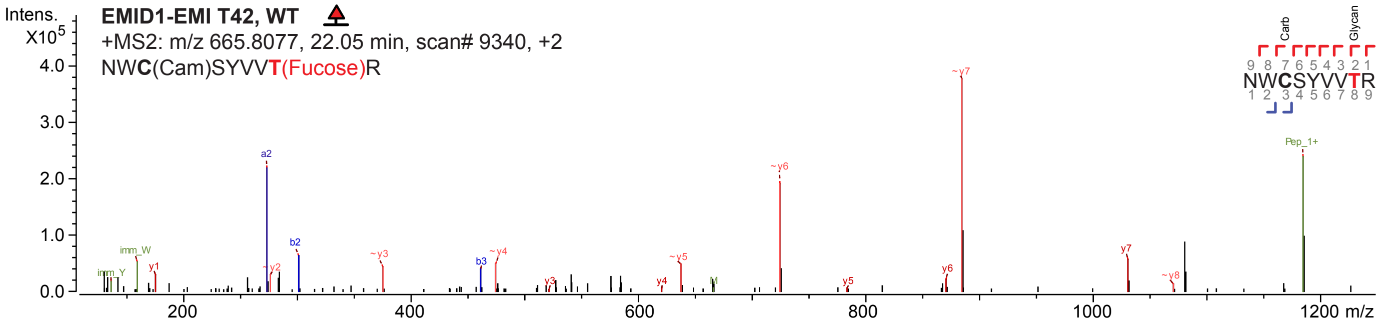
EMID1-EMI T42, WT
+MS2: m/z 665.8077, 22.05 min, scan# 9340, +2
NWC(Cam)SYVVT(Fucose)R
Intens.
X10^5
m/z
NWCSYVVTR
Carb
Glycan
Pep_1+

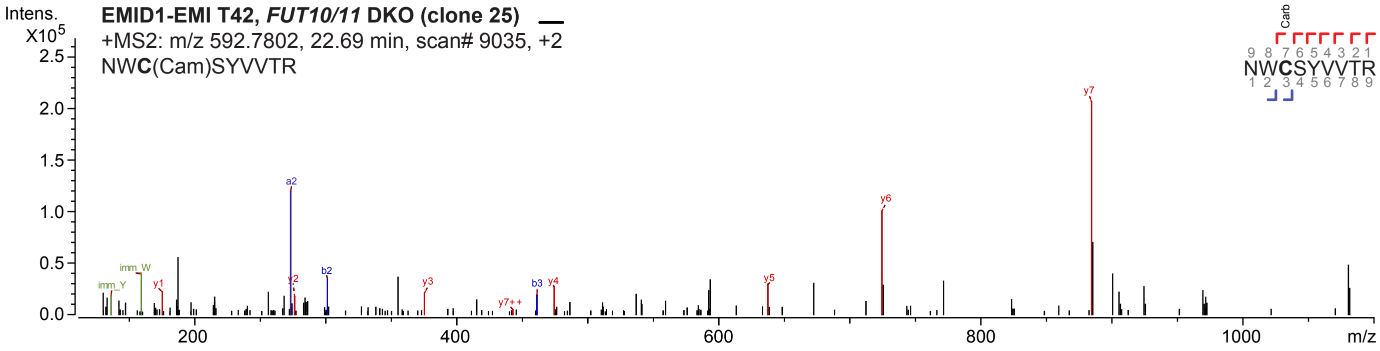
EMID1-EMI T42, FUT10/11 DKO (clone 25)
+MS2: m/z 592.7802, 22.69 min, scan# 9035, +2
NWC(Cam)SYVVTR
Intens.
X10^5
m/z
NWCSYVVTR
Carb

MS2 spectra for peptides used in Supplementary Fig. S2a

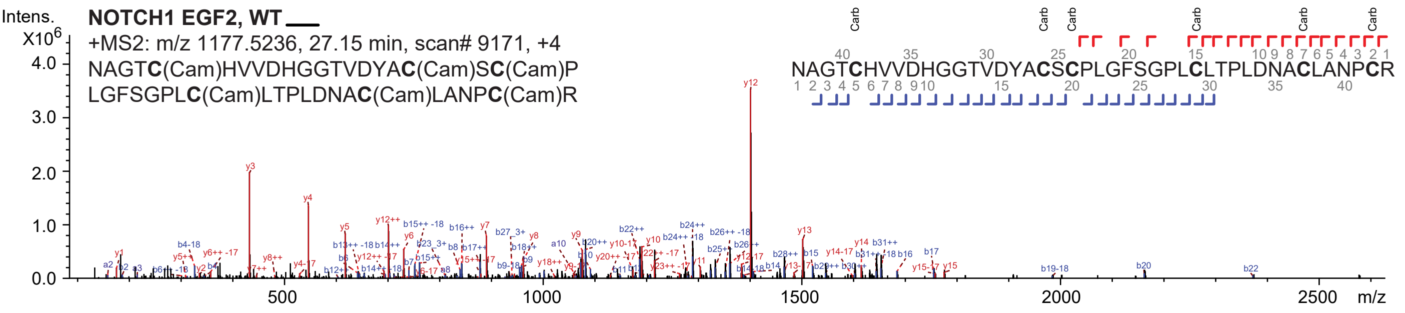

NOTCH1 EGF2, WT
+MS2: m/z 1177.5236, 27.15 min, scan# 9171, +4
NAGT**C**(Cam)HVVDHGGTVDYA**C**(Cam)S**C**(Cam)PLGFSGPL**C**(Cam)LTPLDNA**C**(Cam)LANP**C**(Cam)R

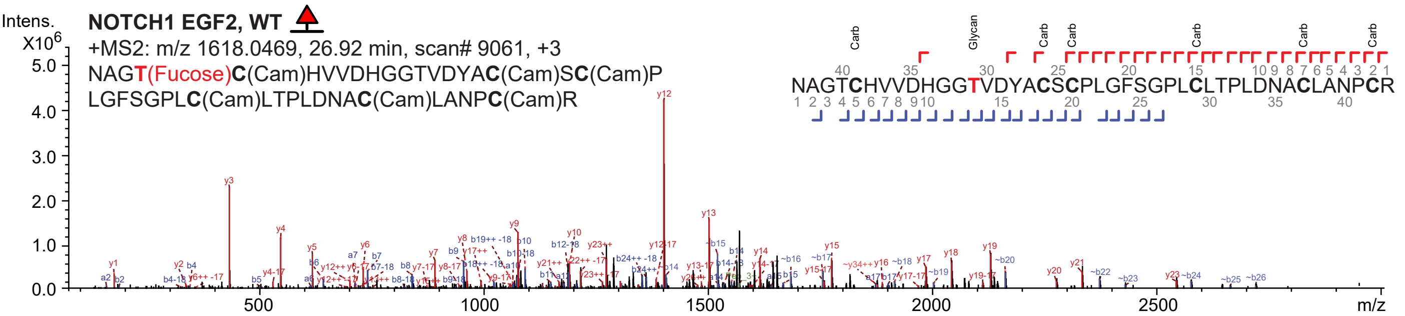

NOTCH1 EGF2, WT
+MS2: m/z 1618.0469, 26.92 min, scan# 9061, +3
NAG**T(Fucose)C**(Cam)HVVDHGGTVDYA**C**(Cam)S**C**(Cam)PLGFSGPL**C**(Cam)LTPLDNA**C**(Cam)LANP**C**(Cam)R

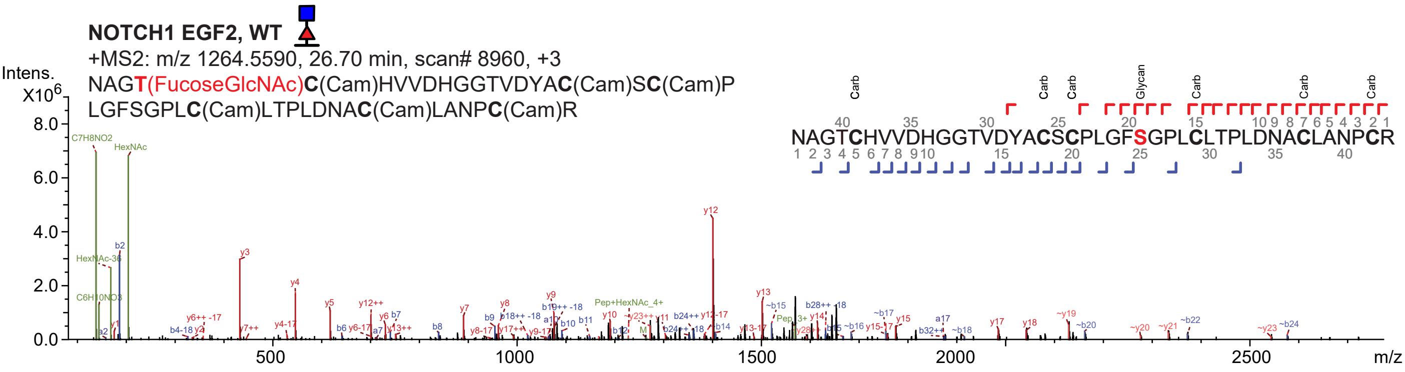

NOTCH1 EGF2, WT
+MS2: m/z 1264.5590, 26.70 min, scan# 8960, +3
NAG**T(FucoseGlcNAc)C**(Cam)HVVDHGGTVDYA**C**(Cam)S**C**(Cam)PLGFSGPL**C**(Cam)LTPLDNA**C**(Cam)LANP**C**(Cam)R

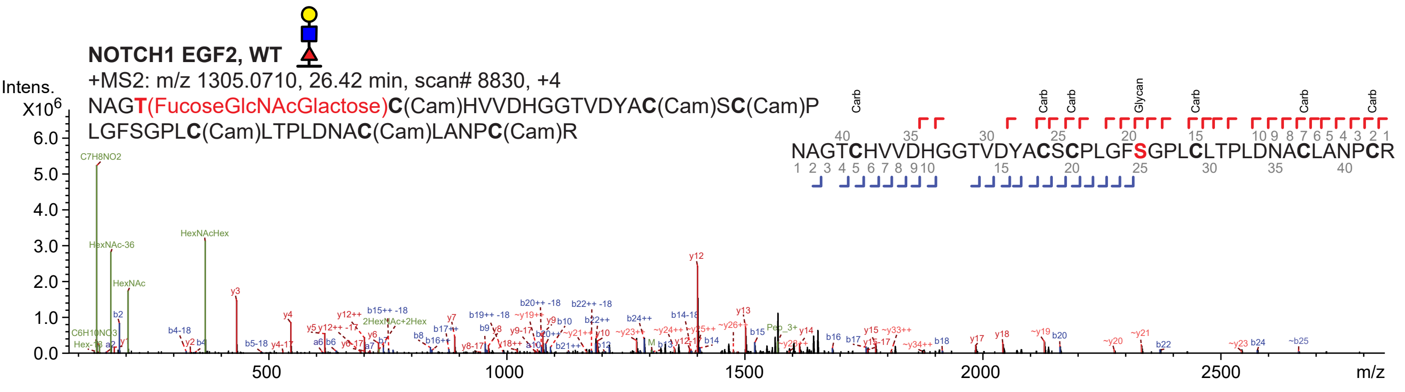

NOTCH1 EGF2, WT
+MS2: m/z 1305.0710, 26.42 min, scan# 8830, +4
NAG**T(FucoseGlcNAcGlactose)C**(Cam)HVVDHGGTVDYA**C**(Cam)S**C**(Cam)PLGFSGPL**C**(Cam)LTPLDNA**C**(Cam)LANP**C**(Cam)R

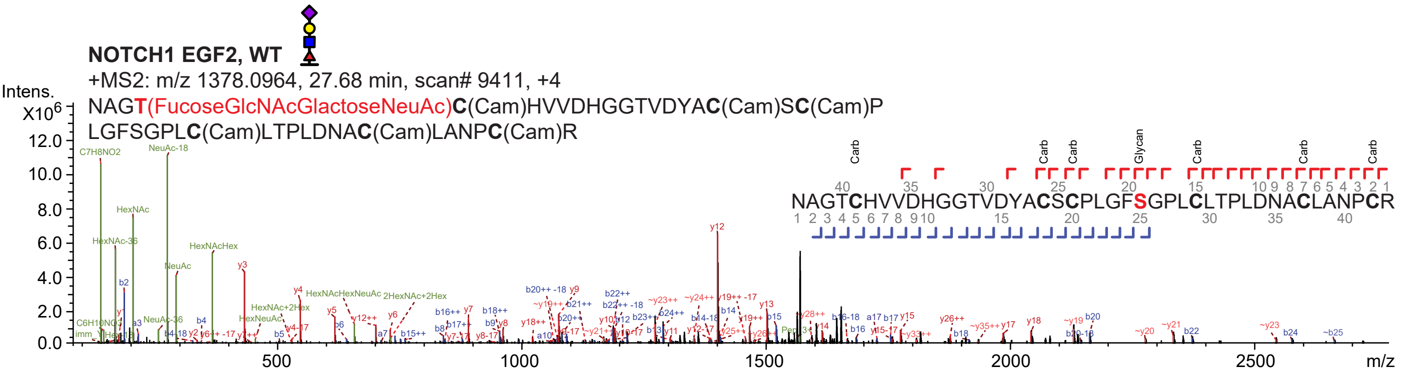

NOTCH1 EGF2, WT
+MS2: m/z 1378.0964, 27.68 min, scan# 9411, +4
NAG**T(FucoseGlcNAcGlactoseNeuAc)C**(Cam)HVVDHGGTVDYA**C**(Cam)S**C**(Cam)PLGFSGPL**C**(Cam)LTPLDNA**C**(Cam)LANP**C**(Cam)R

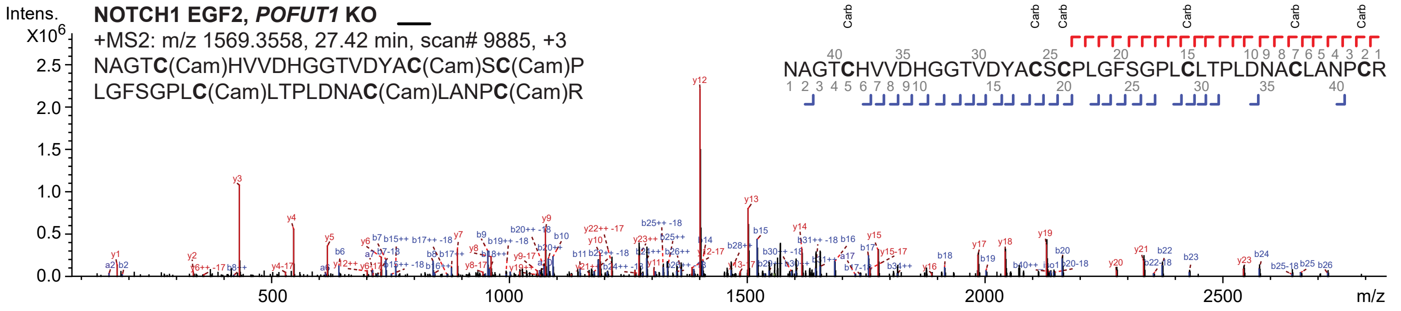

NOTCH1 EGF2, *POFUT1* KO
+MS2: m/z 1569.3558, 27.42 min, scan# 9885, +3
NAGT**C**(Cam)HVVDHGGTVDYA**C**(Cam)S**C**(Cam)P
LGFSGPL**C**(Cam)LTPLDNA**C**(Cam)LANP**C**(Cam)R
Intens.
X10^6
m/z
NAGTCHVVDHGGTVDYACSCPLGFSGPLCLTPLDNACLANPCR

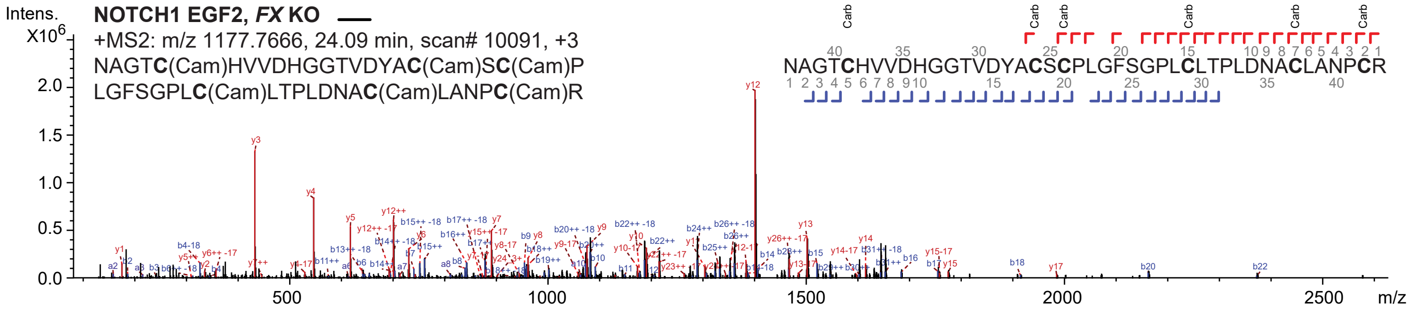

NOTCH1 EGF2, *FX* KO
+MS2: m/z 1177.7666, 24.09 min, scan# 10091, +3
NAGT**C**(Cam)HVVDHGGTVDYA**C**(Cam)S**C**(Cam)P
LGFSGPL**C**(Cam)LTPLDNA**C**(Cam)LANP**C**(Cam)R
Intens.
X10^6
m/z
NAGTCHVVDHGGTVDYACSCPLGFSGPLCLTPLDNACLANPCR

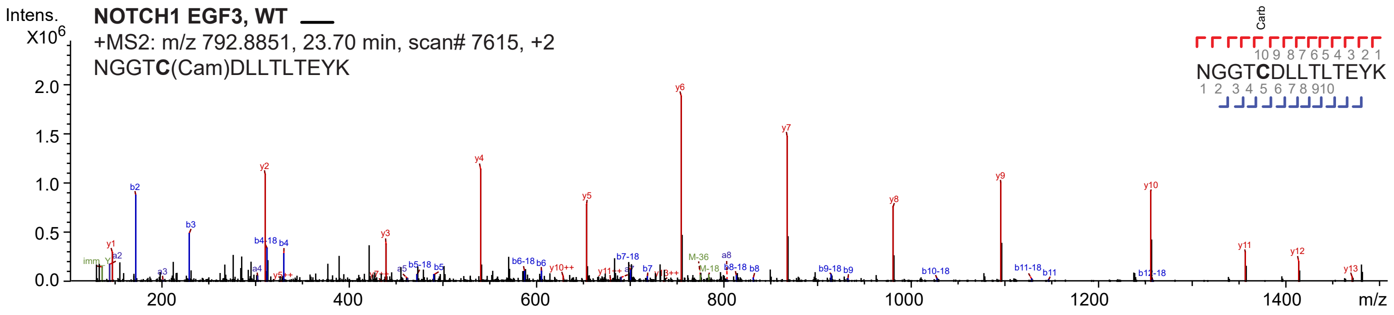

NOTCH1 EGF3, WT
+MS2: m/z 792.8851, 23.70 min, scan# 7615, +2
NGGT**C**(Cam)DLLTLTEYK
Intens.
X10^6
m/z
NGGTCDLLTLTEYK

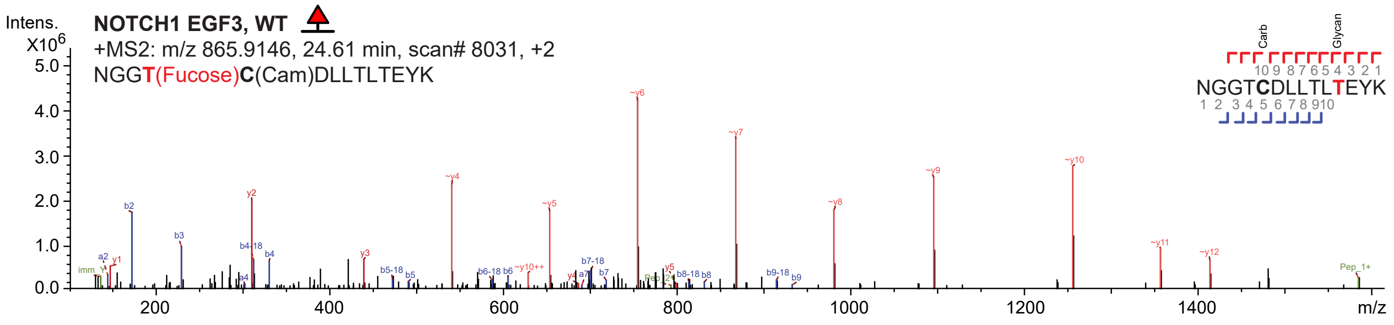

NOTCH1 EGF3, WT
+MS2: m/z 865.9146, 24.61 min, scan# 8031, +2
NGGT(Fucose)**C**(Cam)DLLTLTEYK
Intens.
X10^6
m/z
NGGTCDLLTLTEYK

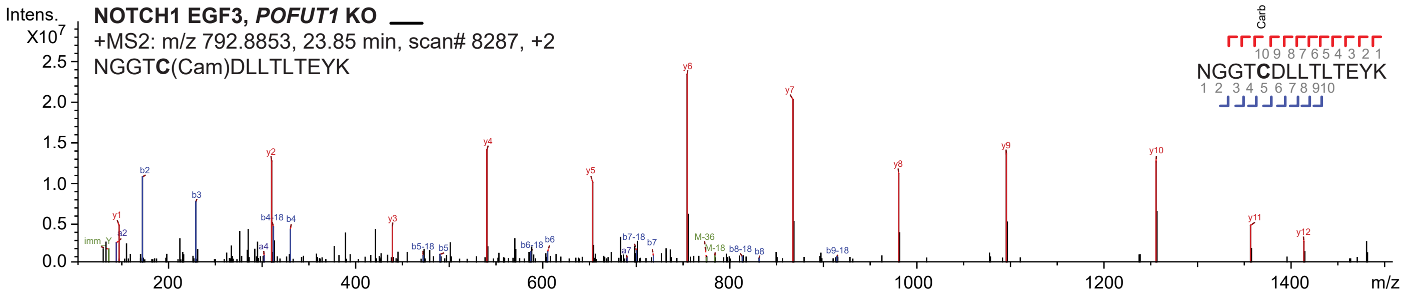

NOTCH1 EGF3, *POFUT1* KO
+MS2: m/z 792.8853, 23.85 min, scan# 8287, +2
NGGT**C**(Cam)DLLTLTEYK
Intens.
X10^7
m/z
NGGTCDLLTLTEYK

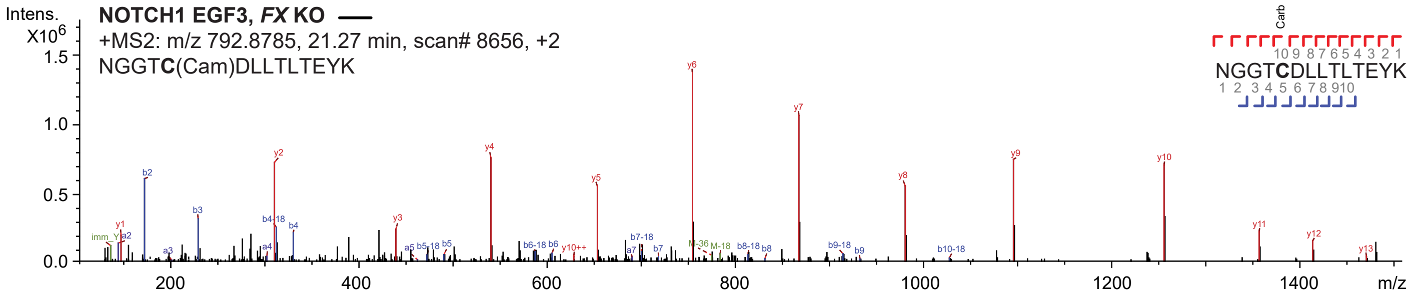
NOTCH1 EGF3, FX KO
+MS2: m/z 792.8785, 21.27 min, scan# 8656, +2
NGGTC(Cam)DLLTLTEYK
Intens. X10^6
m/z
Carb
NGGTCDLLTLTEYK

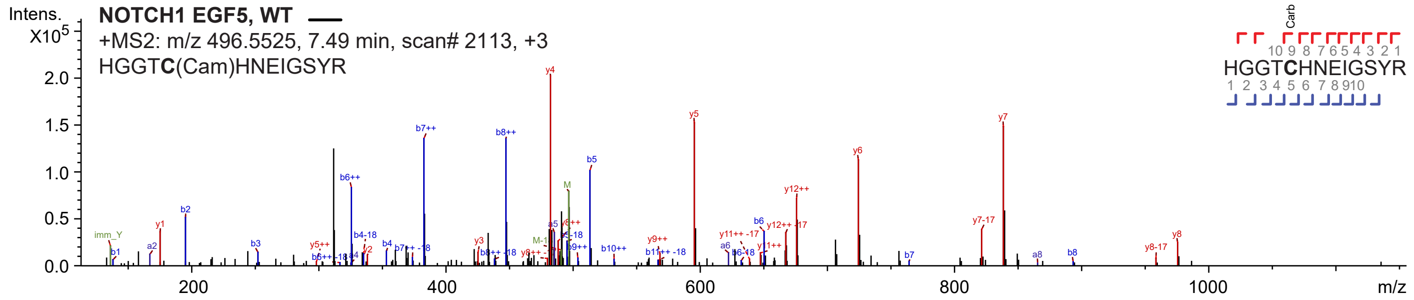
NOTCH1 EGF5, WT
+MS2: m/z 496.5525, 7.49 min, scan# 2113, +3
HGGTC(Cam)HNEIGSYR
Intens. X10^5
m/z
Carb
HGGTCHNEIGSYR

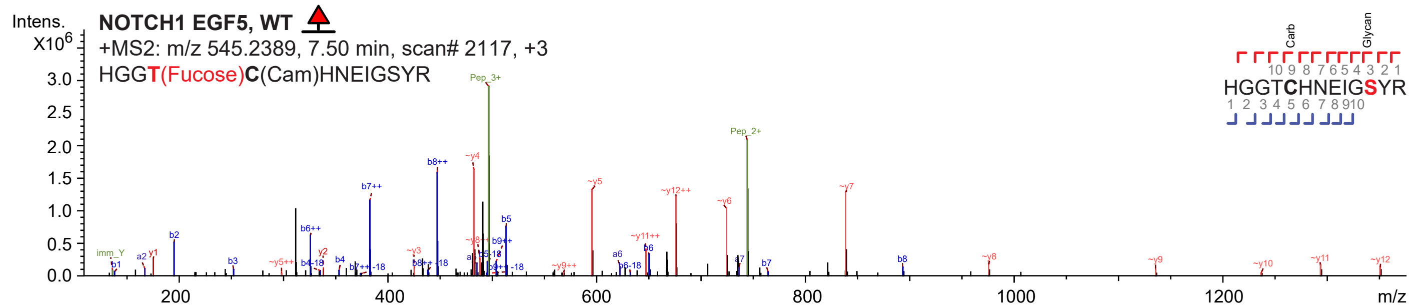
NOTCH1 EGF5, WT
+MS2: m/z 545.2389, 7.50 min, scan# 2117, +3
HGGT(Fucose)C(Cam)HNEIGSYR
Intens. X10^6
m/z
Carb
Glycan
HGGTCHNEIGSYR
Pep_3+
Pep_2+

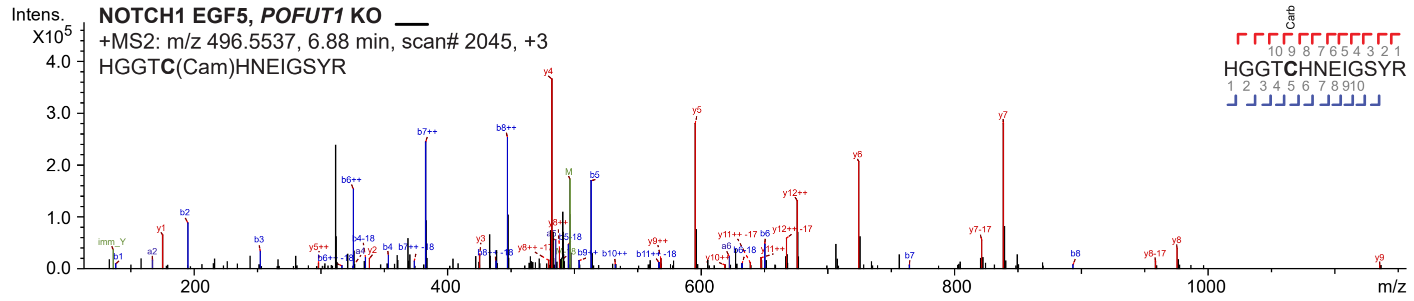
NOTCH1 EGF5, POFUT1 KO
+MS2: m/z 496.5537, 6.88 min, scan# 2045, +3
HGGTC(Cam)HNEIGSYR
Intens. X10^5
m/z
Carb
HGGTCHNEIGSYR

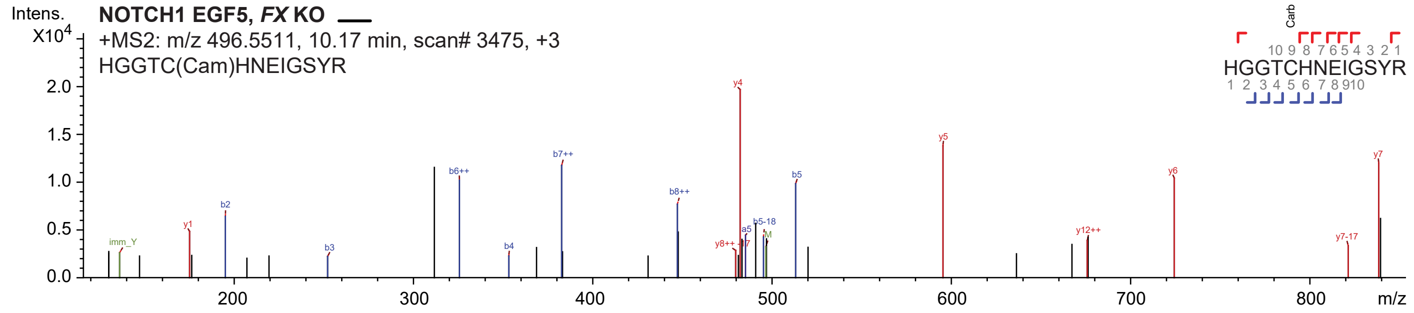
NOTCH1 EGF5, FX KO
+MS2: m/z 496.5511, 10.17 min, scan# 3475, +3
HGGTC(Cam)HNEIGSYR
Intens. X10^4
m/z
Carb
HGGTCHNEIGSYR

MS2 spectra for peptides used in Supplementary Fig. S2b

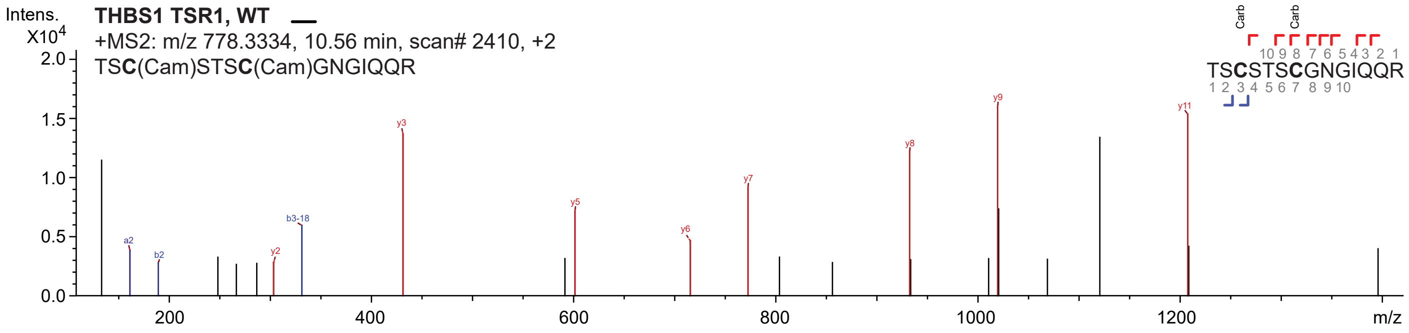

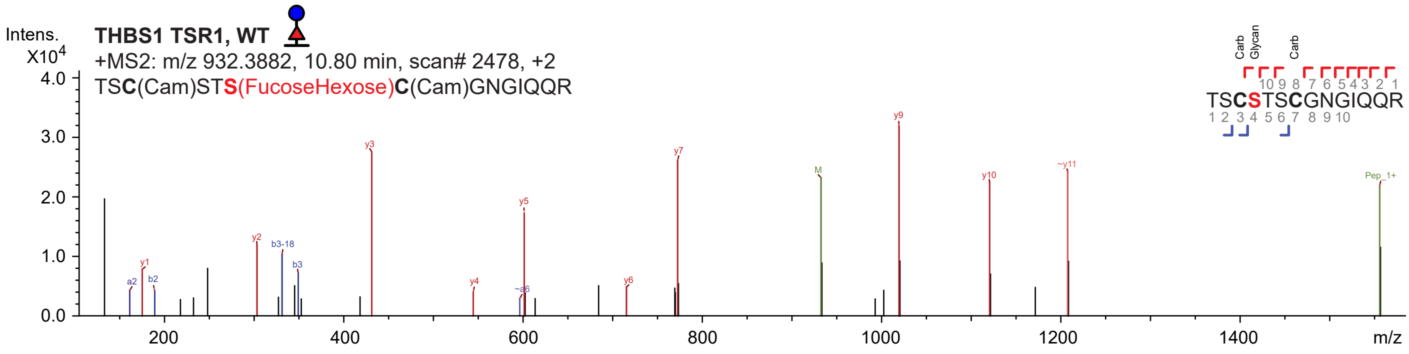

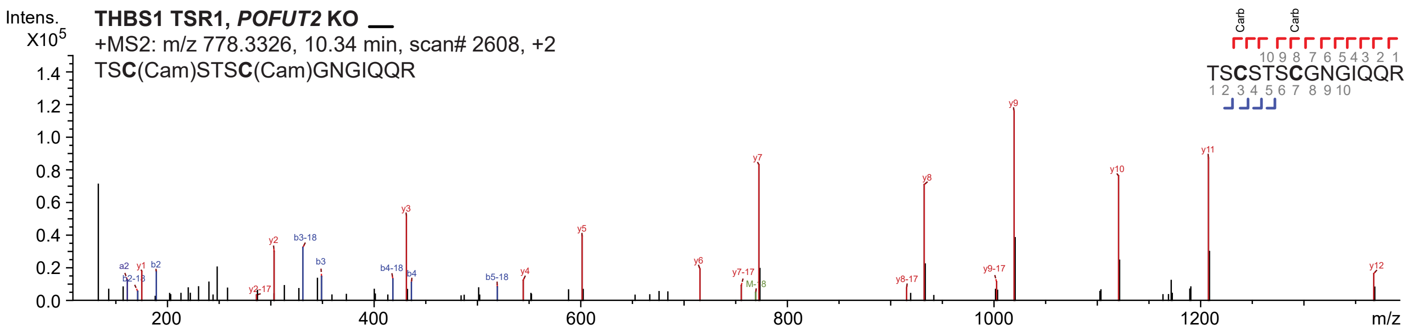

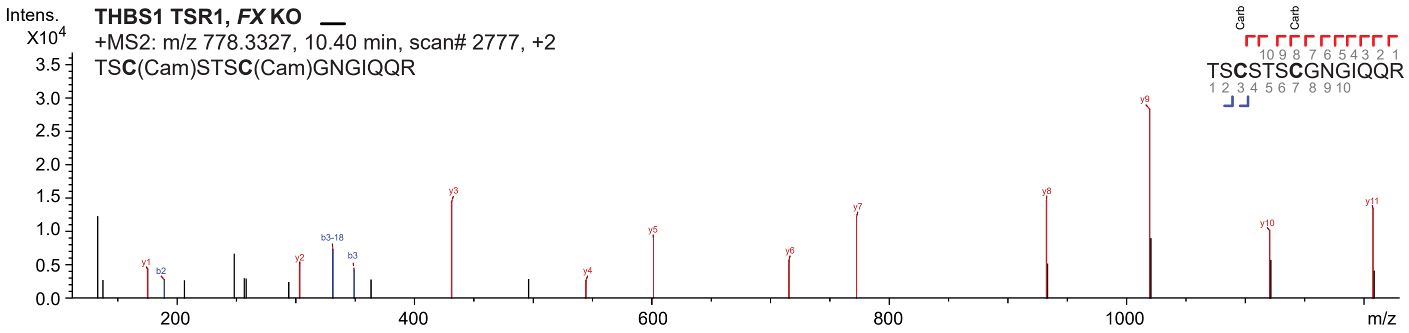

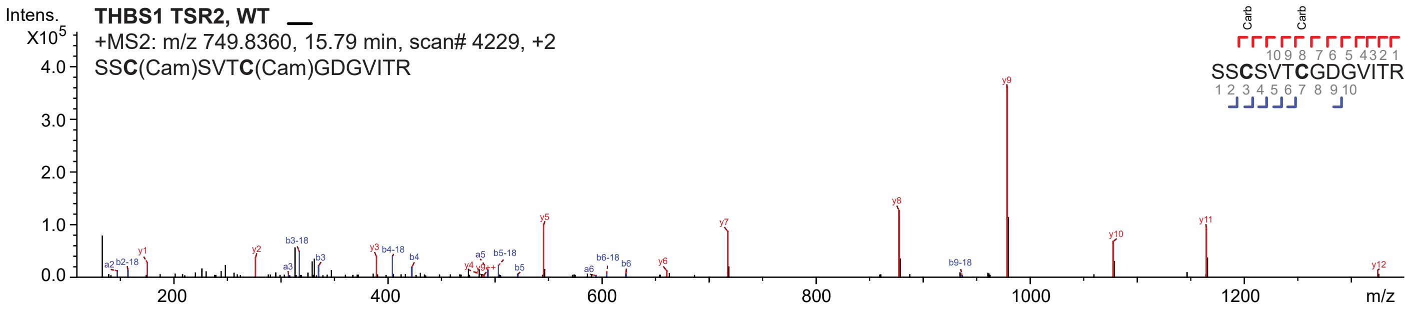

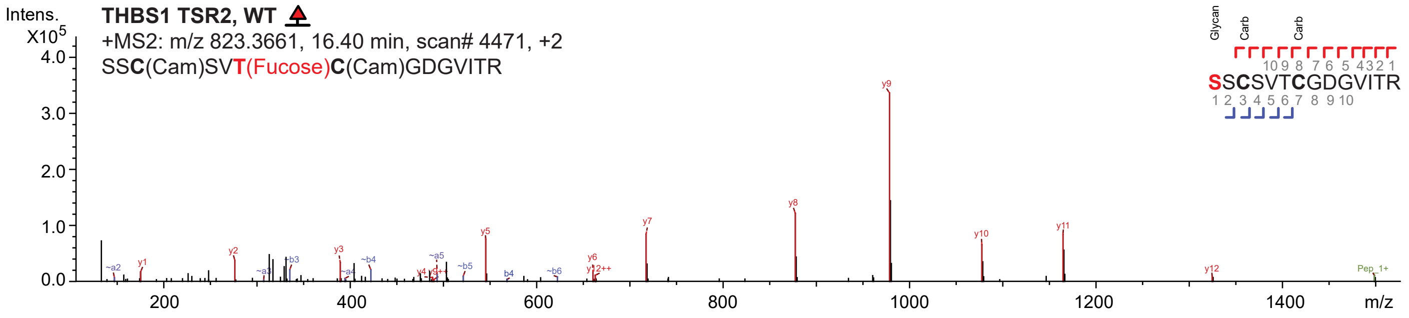
THBS1 TSR2, WT
+MS2: m/z 823.3661, 16.40 min, scan# 4471, +2
SSC(Cam)SVT(Fucose)C(Cam)GDGVITR
Intens.
X10^5
m/z
SSCSVTCGDGVITR

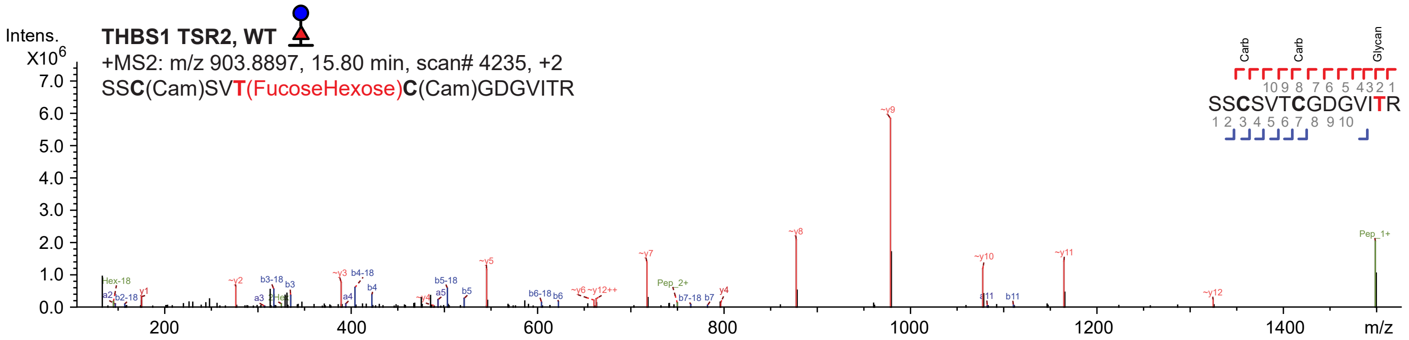
THBS1 TSR2, WT
+MS2: m/z 903.8897, 15.80 min, scan# 4235, +2
SSC(Cam)SVT(FucoseHexose)C(Cam)GDGVITR
Intens.
X10^6
m/z
SSCSVTCGDGVITR

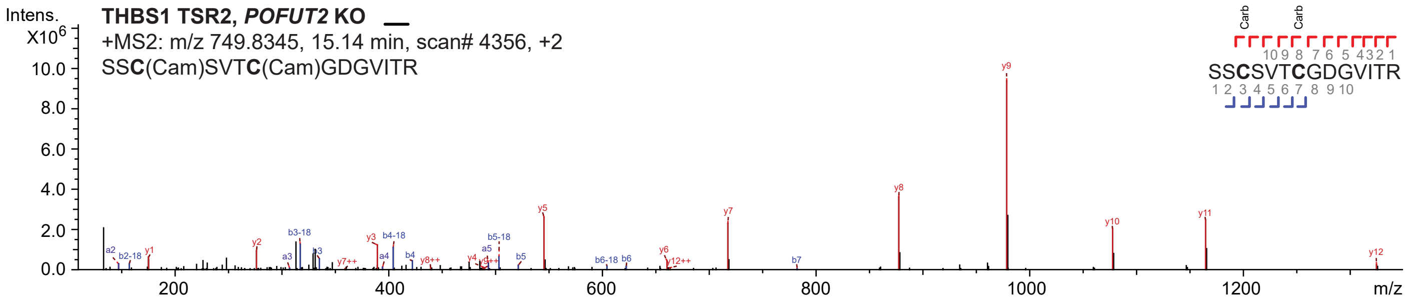
THBS1 TSR2, *POFUT2* KO
+MS2: m/z 749.8345, 15.14 min, scan# 4356, +2
SSC(Cam)SVTC(Cam)GDGVITR
Intens.
X10^6
m/z
SSCSVTCGDGVITR

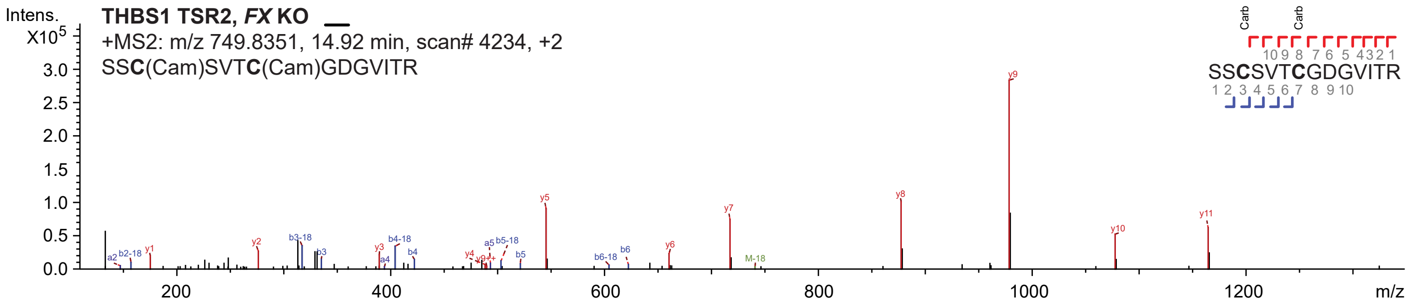
THBS1 TSR2, *FX* KO
+MS2: m/z 749.8351, 14.92 min, scan# 4234, +2
SSC(Cam)SVTC(Cam)GDGVITR
Intens.
X10^5
m/z
SSCSVTCGDGVITR

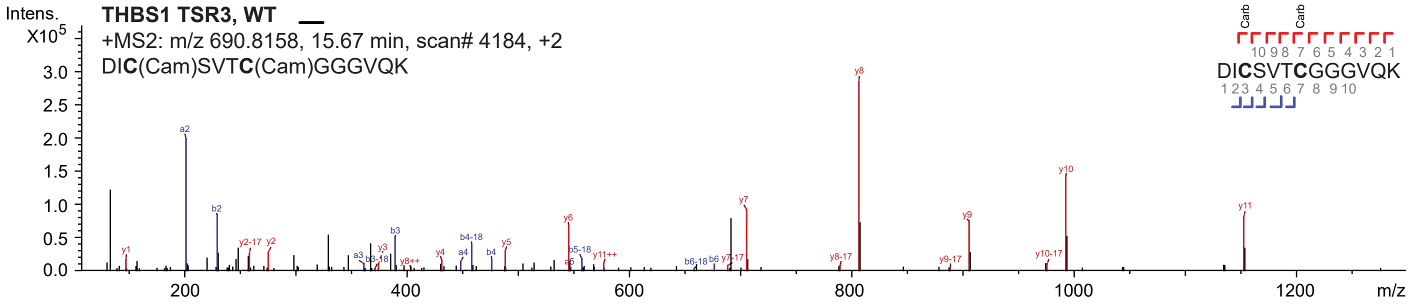
THBS1 TSR3, WT
+MS2: m/z 690.8158, 15.67 min, scan# 4184, +2
DIC(Cam)SVTC(Cam)GGGVQK
Intens.
X10^5
m/z
DICSVTCGGGVQK

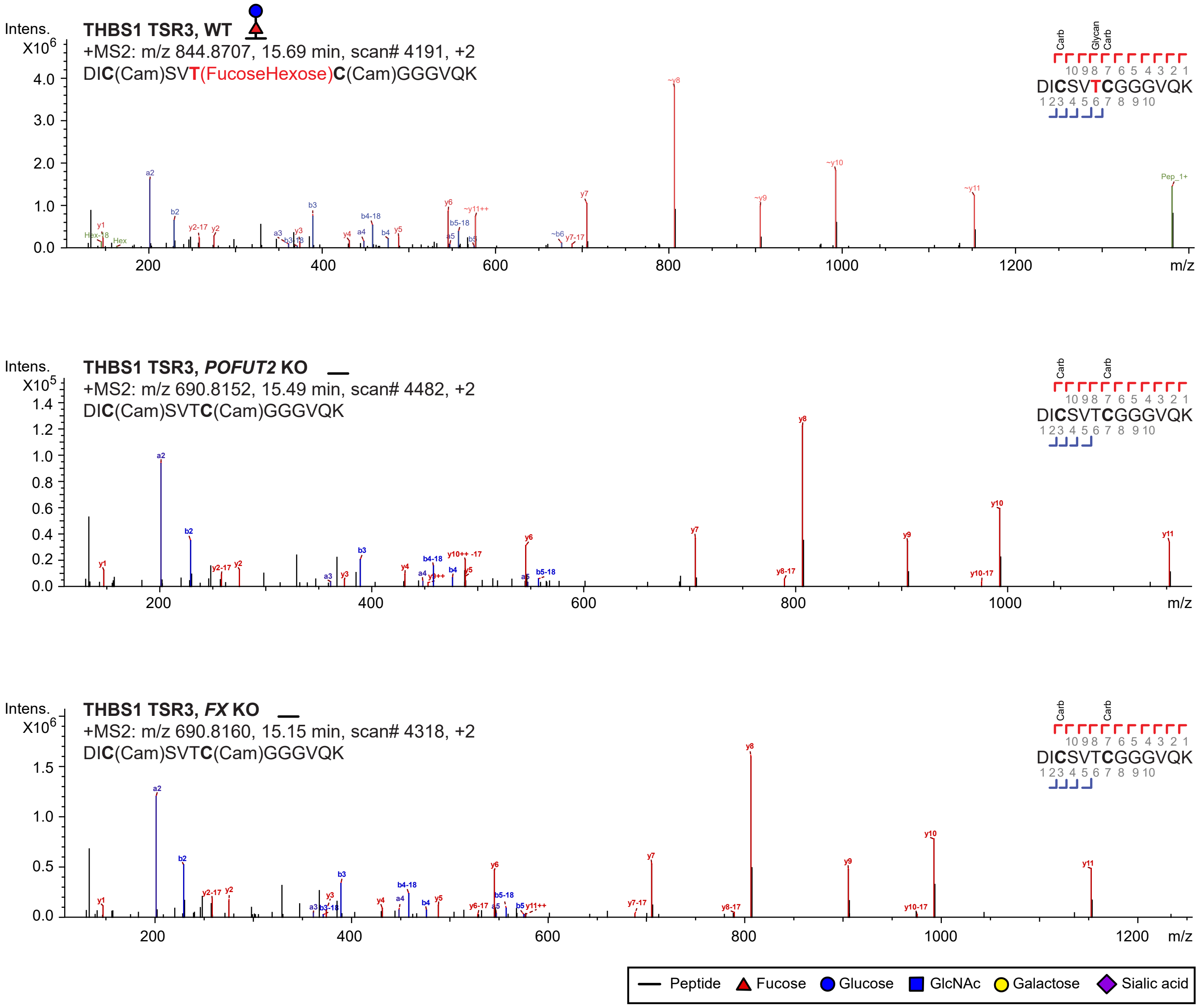
THBS1 TSR3, WT
+MS2: m/z 844.8707, 15.69 min, scan# 4191, +2
DIC(Cam)SVT(FucoseHexose)C(Cam)GGGVQK
Intens.
X10^6
m/z
DICSVTCGGGVQK
Carb
Glycan
Carb
THBS1 TSR3, POFUT2 KO
+MS2: m/z 690.8152, 15.49 min, scan# 4482, +2
DIC(Cam)SVTC(Cam)GGGVQK
Intens.
X10^5
m/z
DICSVTCGGGVQK
THBS1 TSR3, FX KO
+MS2: m/z 690.8160, 15.15 min, scan# 4318, +2
DIC(Cam)SVTC(Cam)GGGVQK
Intens.
X10^6
m/z
DICSVTCGGGVQK
Peptide
Fucose
Glucose
GlcNAc
Galactose
Sialic acid

MS2 spectra for peptides used in Supplementary Fig. S2c

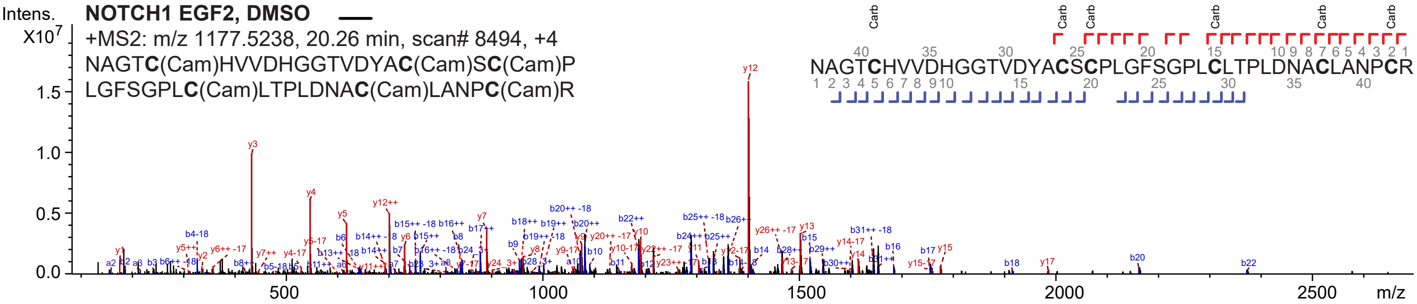

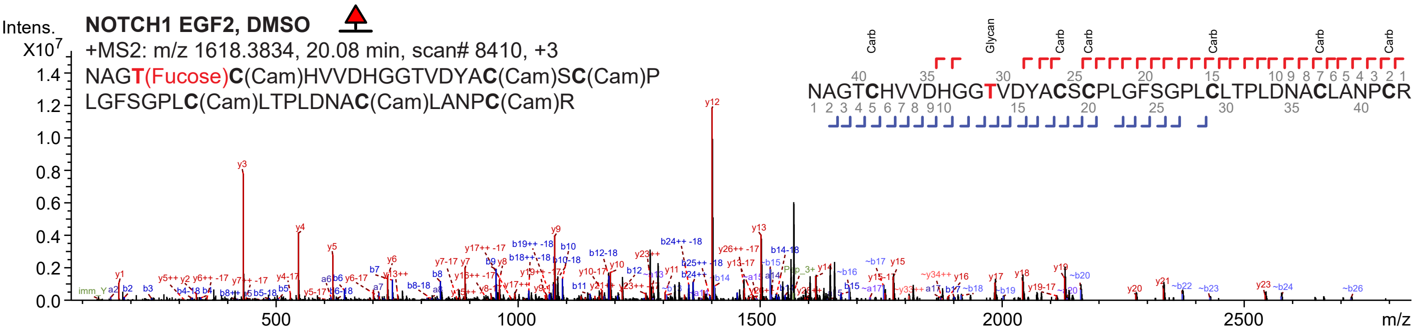

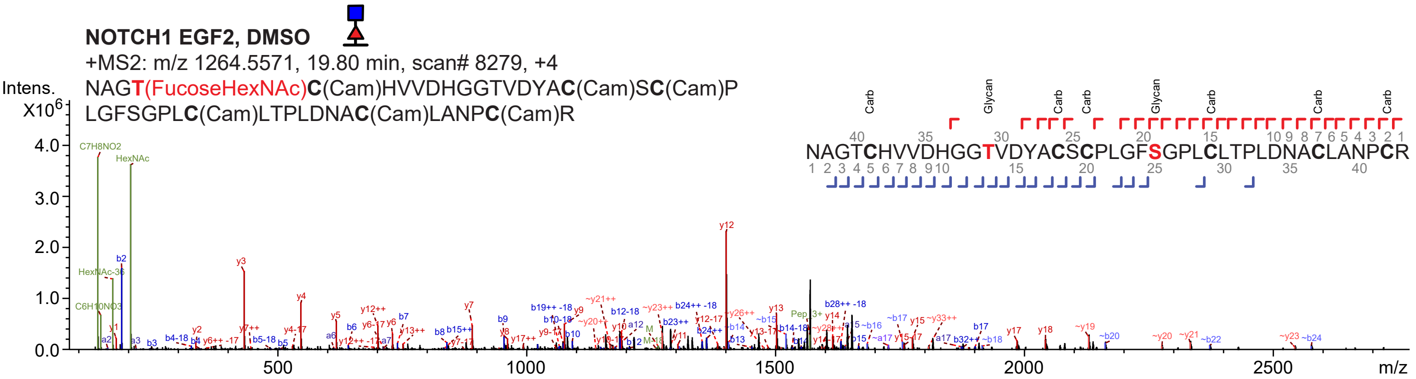

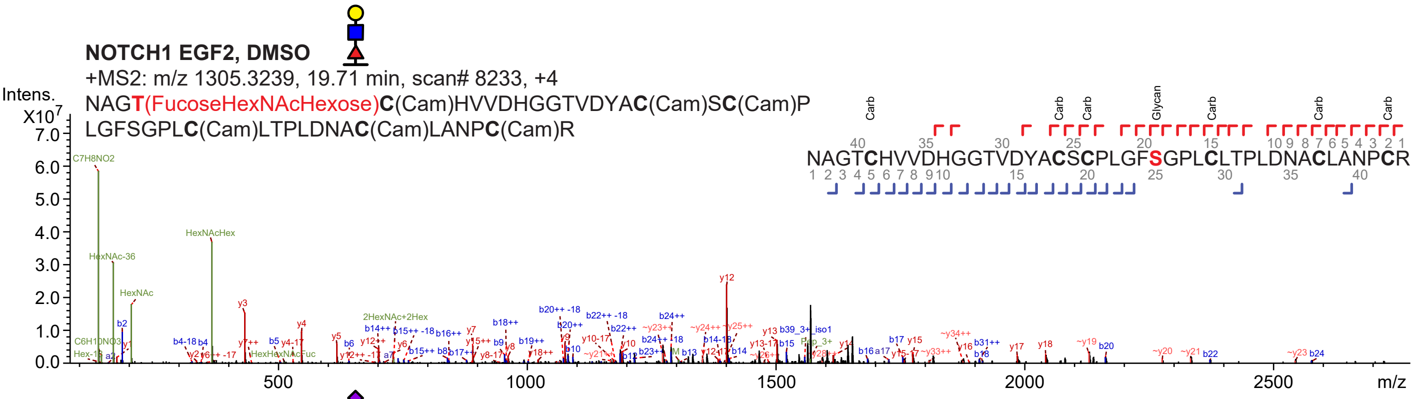

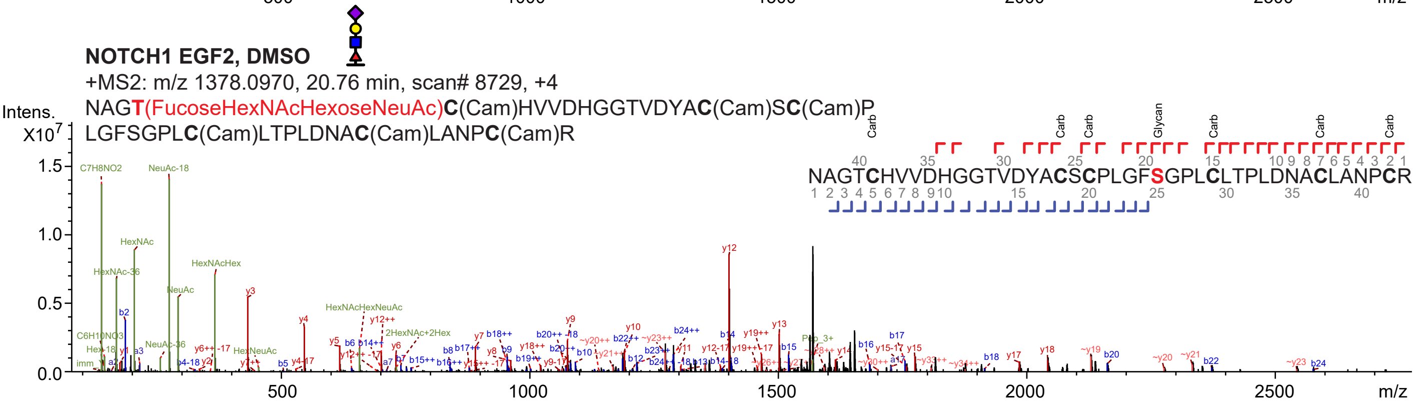

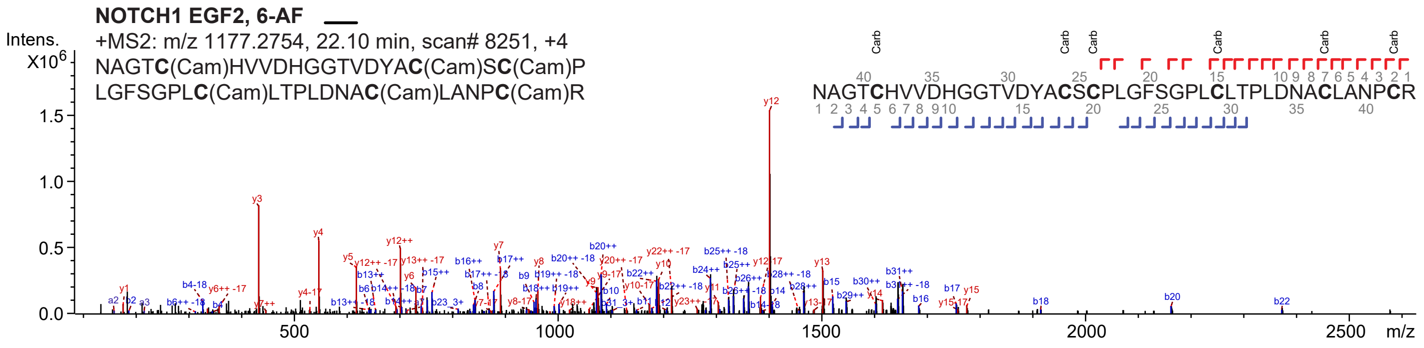
NOTCH1 EGF2, 6-AF
+MS2: m/z 1177.2754, 22.10 min, scan# 8251, +4
NAGTC(Cam)HVVDHGGTVDYAC(Cam)SC(Cam)P
LGFSGPLC(Cam)LTPLDNAC(Cam)LANPC(Cam)R
NAGTCHVVDHGGTVDYACSCPLGFSGPLCLTPLDNACLANPCR
Intens. x10^6
m/z

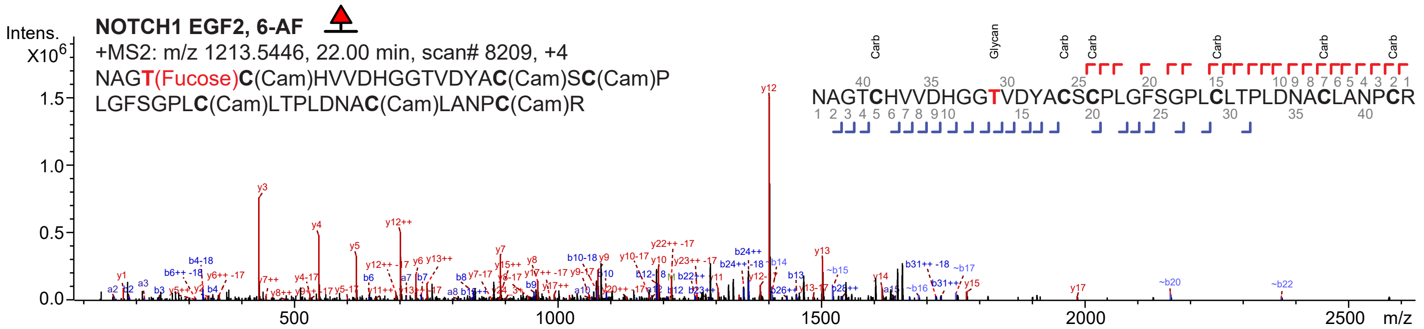
NOTCH1 EGF2, 6-AF
+MS2: m/z 1213.5446, 22.00 min, scan# 8209, +4
NAGT(Fucose)C(Cam)HVVDHGGTVDYAC(Cam)SC(Cam)P
LGFSGPLC(Cam)LTPLDNAC(Cam)LANPC(Cam)R
NAGTCHVVDHGGTVDYACSCPLGFSGPLCLTPLDNACLANPCR
Intens. x10^6
m/z

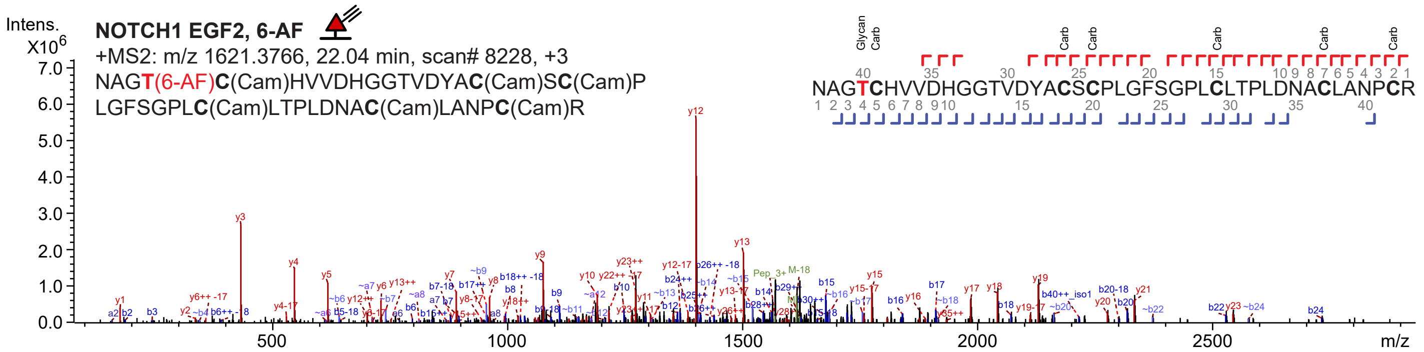
NOTCH1 EGF2, 6-AF
+MS2: m/z 1621.3766, 22.04 min, scan# 8228, +3
NAGT(6-AF)C(Cam)HVVDHGGTVDYAC(Cam)SC(Cam)P
LGFSGPLC(Cam)LTPLDNAC(Cam)LANPC(Cam)R
NAGTCHVVDHGGTVDYACSCPLGFSGPLCLTPLDNACLANPCR
Intens. x10^6
m/z

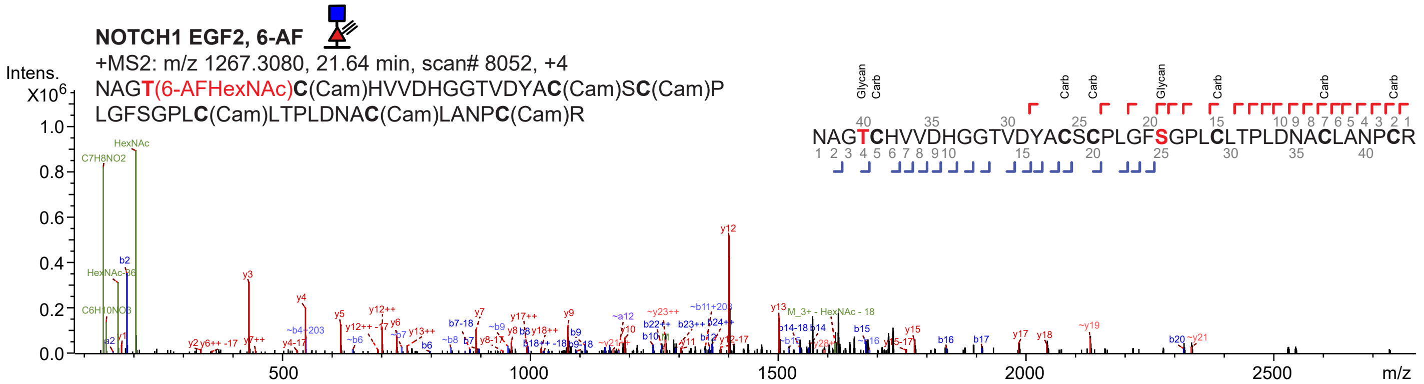
NOTCH1 EGF2, 6-AF
+MS2: m/z 1267.3080, 21.64 min, scan# 8052, +4
NAGT(6-AFHexNAc)C(Cam)HVVDHGGTVDYAC(Cam)SC(Cam)P
LGFSGPLC(Cam)LTPLDNAC(Cam)LANPC(Cam)R
NAGTCHVVDHGGTVDYACSCPLGFSGPLCLTPLDNACLANPCR
Intens. x10^6
m/z

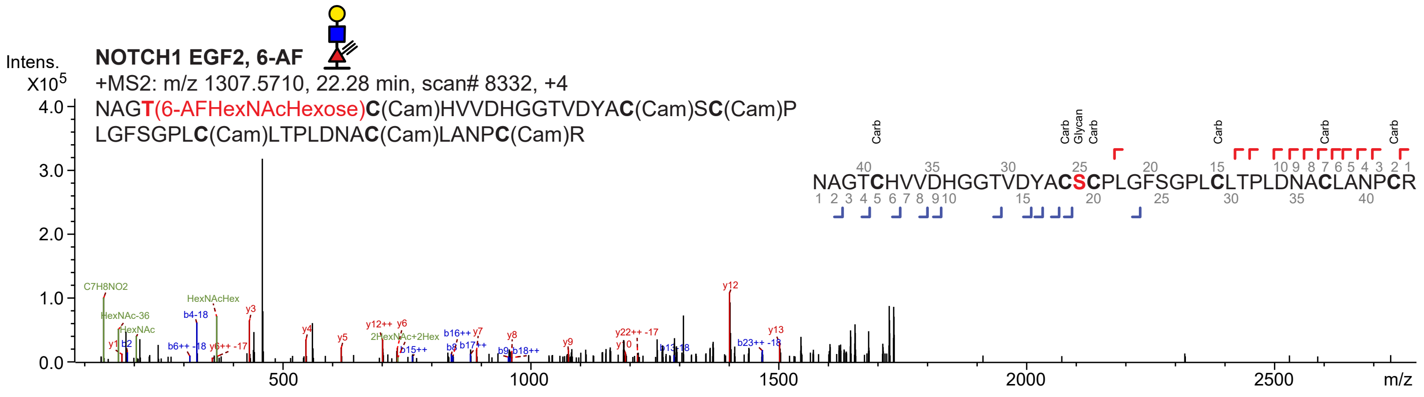
NOTCH1 EGF2, 6-AF
+MS2: m/z 1307.5710, 22.28 min, scan# 8332, +4
NAGT(6-AFHexNAcHexose)C(Cam)HVVDHGGTVDYAC(Cam)SC(Cam)P
LGFSGPLC(Cam)LTPLDNAC(Cam)LANPC(Cam)R
NAGTCHVVDHGGTVDYACSCPLGFSGPLCLTPLDNACLANPCR
Intens. x10^5
m/z

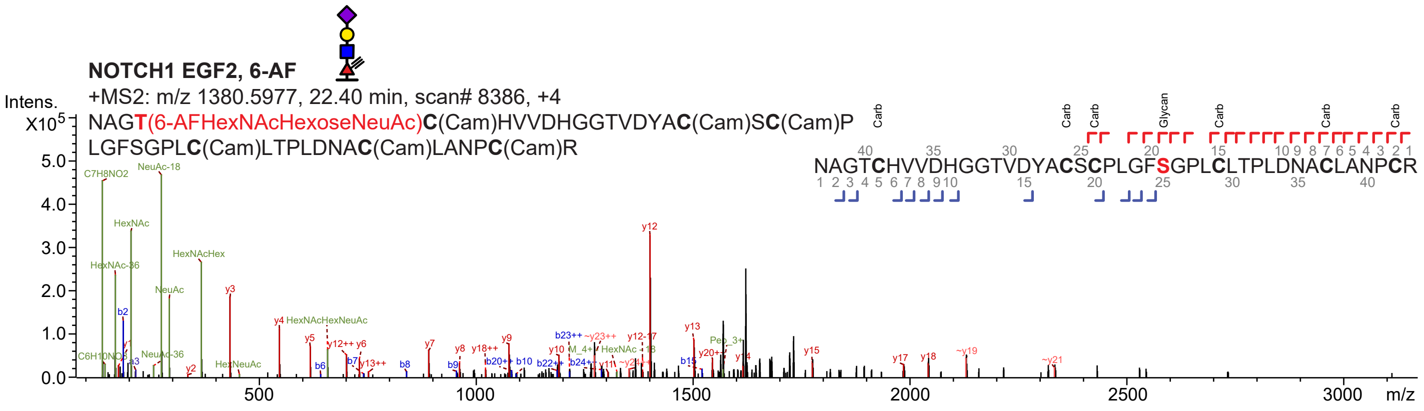
NOTCH1 EGF2, 6-AF
+MS2: m/z 1380.5977, 22.40 min, scan# 8386, +4
NAGT(6-AFHexNAcHexoseNeuAc)C(Cam)HVVDHGGTVDYAC(Cam)SC(Cam)P
LGFSGPLC(Cam)LTPLDNAC(Cam)LANPC(Cam)R
NAGTCHVVDHGGTVDYACSCPLGFSGPLCLTPLDNACLANPCR
Intens.
m/z

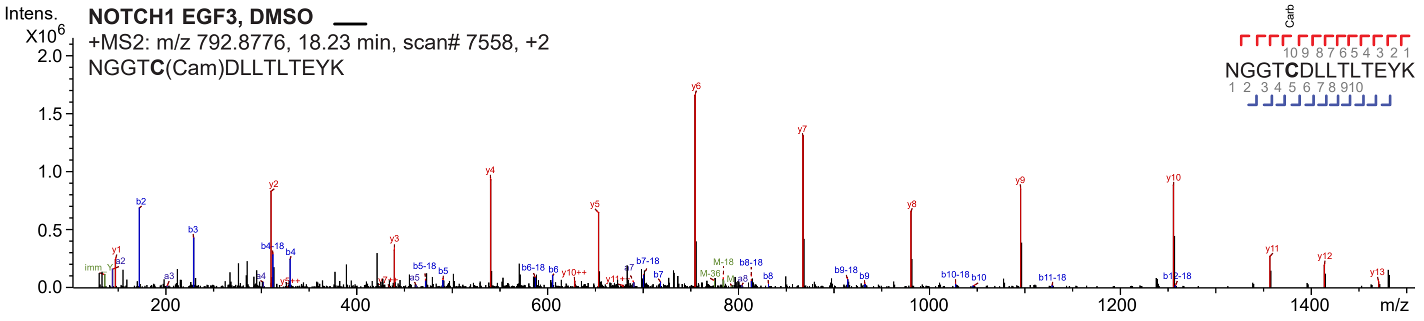
NOTCH1 EGF3, DMSO
+MS2: m/z 792.8776, 18.23 min, scan# 7558, +2
NGGTC(Cam)DLLTLTEYK
NGGTCDLLTLTEYK
Intens.
m/z

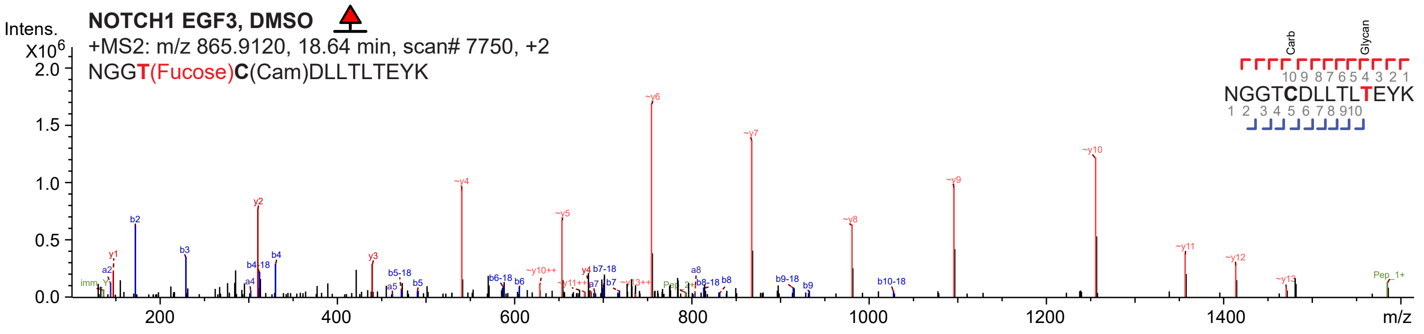
NOTCH1 EGF3, DMSO
+MS2: m/z 865.9120, 18.64 min, scan# 7750, +2
NGGT(Fucose)C(Cam)DLLTLTEYK
NGGTCDLLTLTEYK
Intens.
m/z

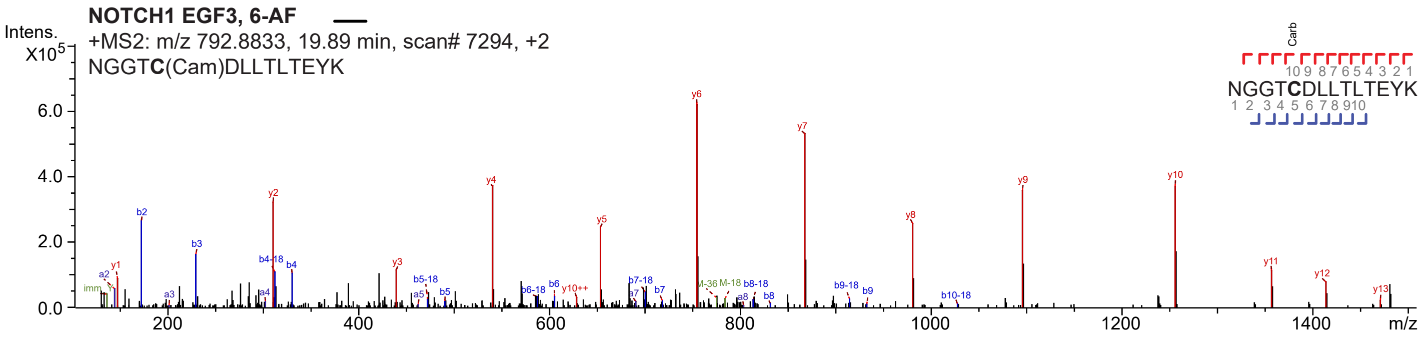
NOTCH1 EGF3, 6-AF
+MS2: m/z 792.8833, 19.89 min, scan# 7294, +2
NGGTC(Cam)DLLTLTEYK
NGGTCDLLTLTEYK
Intens.
m/z

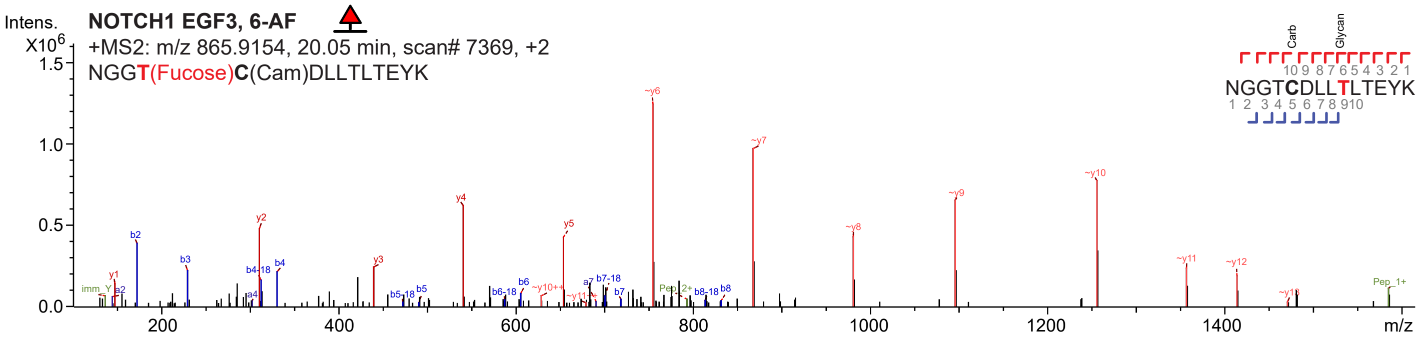
NOTCH1 EGF3, 6-AF
+MS2: m/z 865.9154, 20.05 min, scan# 7369, +2
NGGT(Fucose)C(Cam)DLLTLTEYK
NGGTCDLLTLTEYK
Intens.
m/z

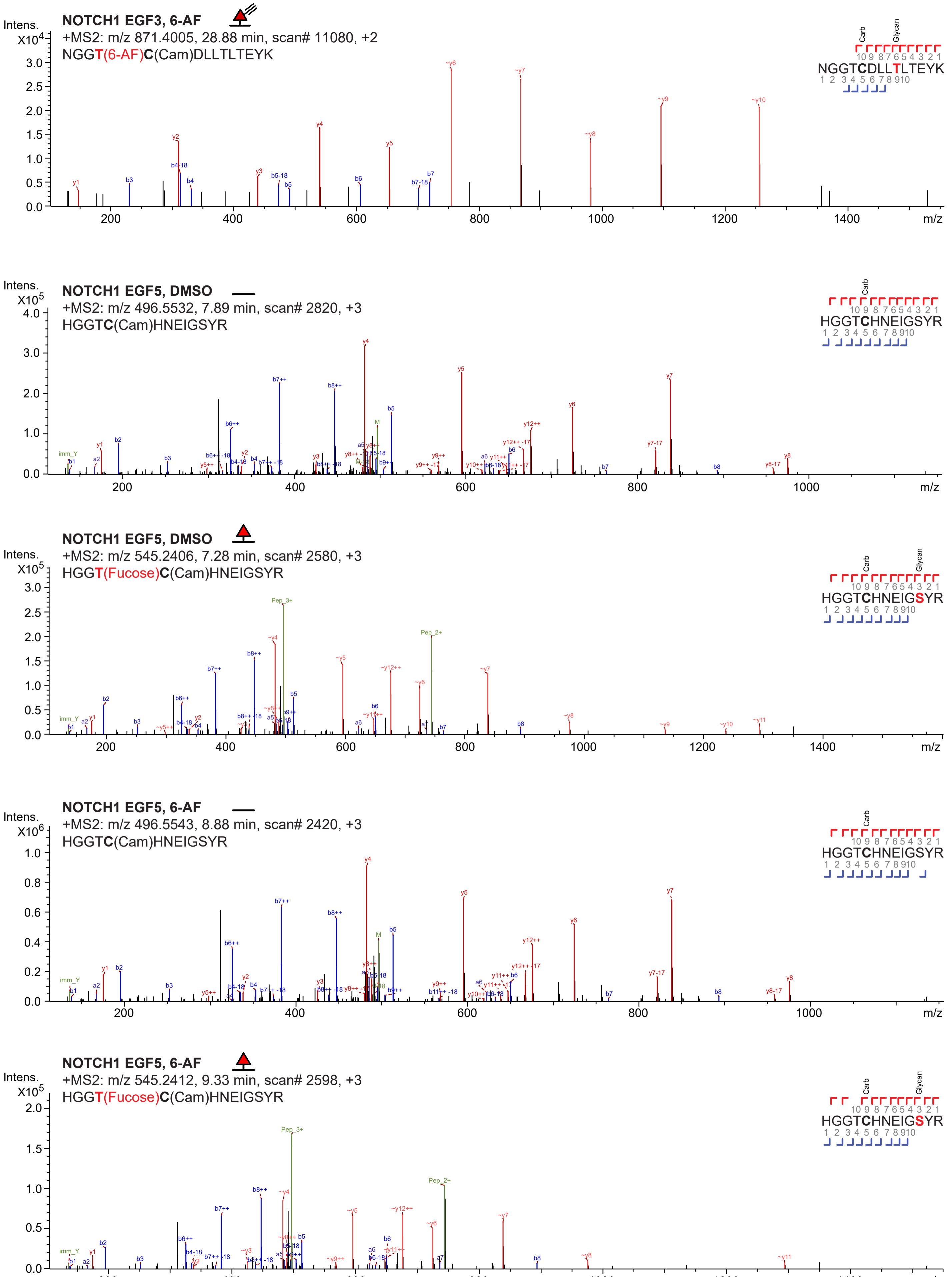
NOTCH1 EGF3, 6-AF
+MS2: m/z 871.4005, 28.88 min, scan# 11080, +2
NGGT(6-AF)C(Cam)DLLTLTEYK
NGGTCDLLTLTEYK
Carb
Glycan
Intens.
X10^4
m/z
NOTCH1 EGF5, DMSO
+MS2: m/z 496.5532, 7.89 min, scan# 2820, +3
HGGTC(Cam)HNEIGSYR
HGGTCHNEIGSYR
Carb
Intens.
X10^5
m/z
NOTCH1 EGF5, DMSO
+MS2: m/z 545.2406, 7.28 min, scan# 2580, +3
HGGT(Fucose)C(Cam)HNEIGSYR
HGGTCHNEIGSYR
Carb
Glycan
Intens.
X10^5
m/z
NOTCH1 EGF5, 6-AF
+MS2: m/z 496.5543, 8.88 min, scan# 2420, +3
HGGTC(Cam)HNEIGSYR
HGGTCHNEIGSYR
Carb
Intens.
X10^6
m/z
NOTCH1 EGF5, 6-AF
+MS2: m/z 545.2412, 9.33 min, scan# 2598, +3
HGGT(Fucose)C(Cam)HNEIGSYR
HGGTCHNEIGSYR
Carb
Glycan
Intens.
X10^5
m/z

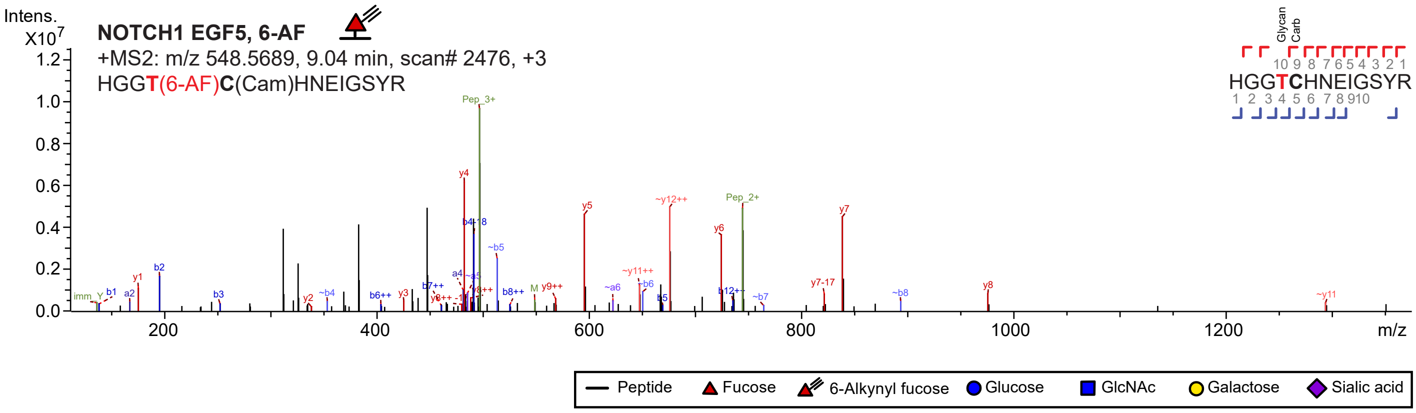

NOTCH1 EGF5, 6-AF
+MS2: m/z 548.5689, 9.04 min, scan# 2476, +3
HGGT(6-AF)C(Cam)HNEIGSYR
Intens.
X10^7
m/z
HGGTCHNEIGSYR
Glycan
Carb
Peptide
Fucose
6-Alkynyl fucose
Glucose
GlcNAc
Galactose
Sialic acid

MS2 spectra for peptides used in Supplementary Fig. S2d

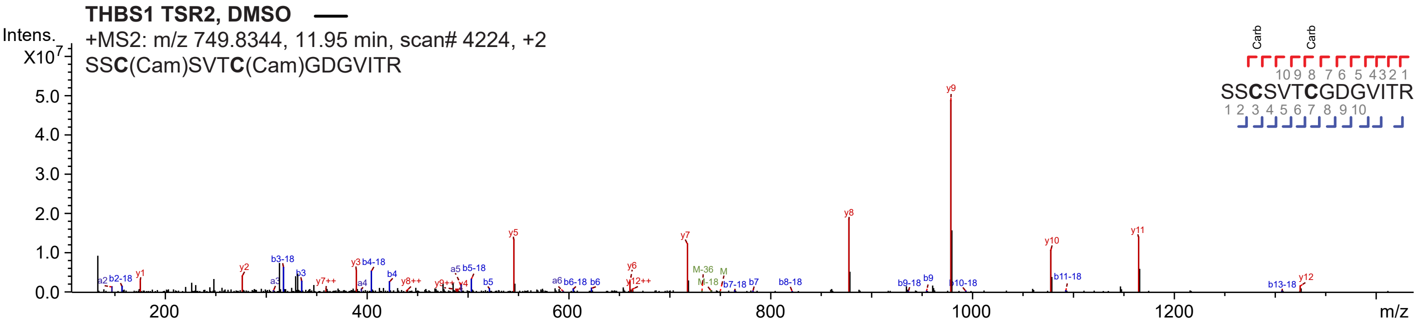

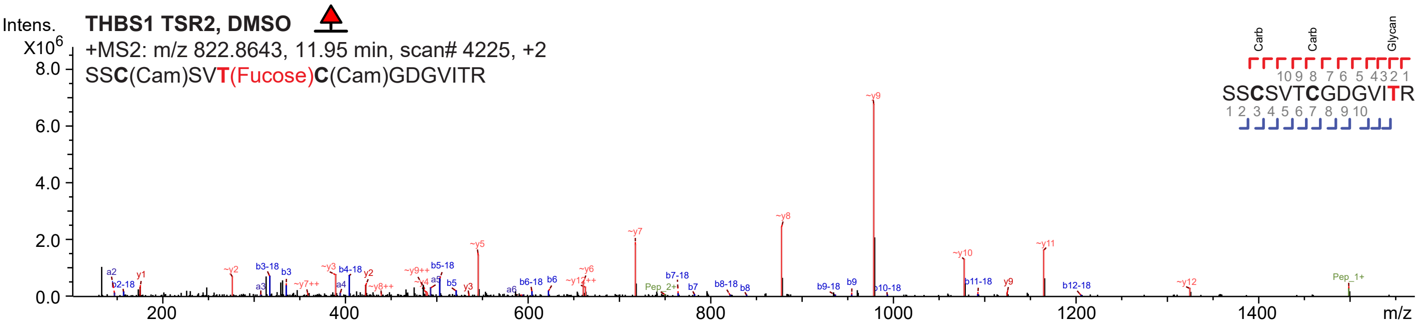

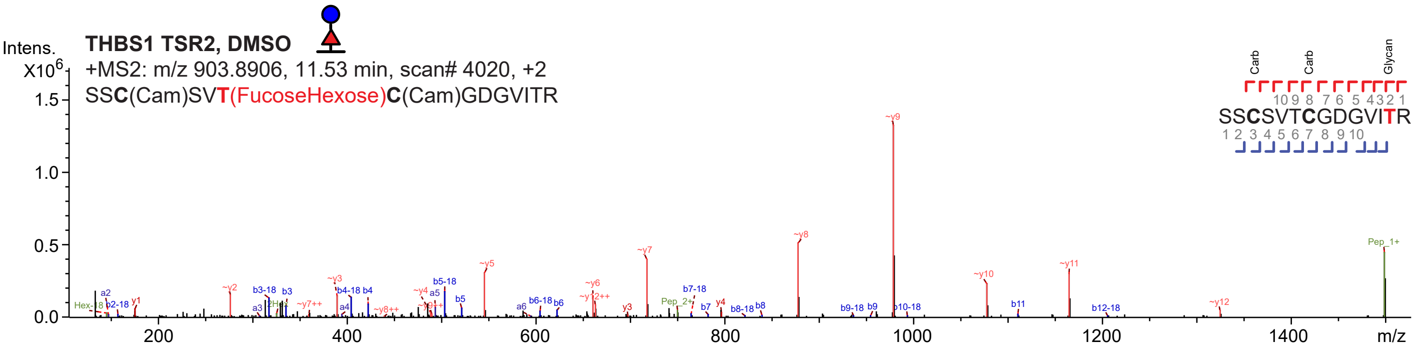

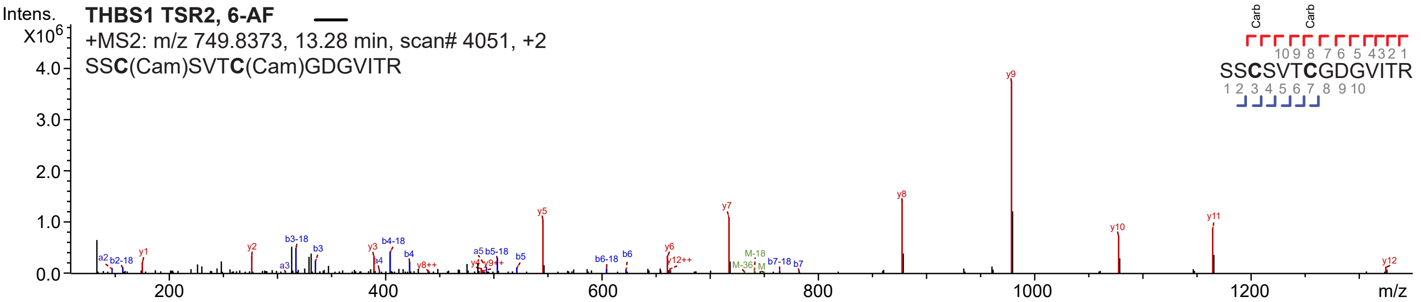

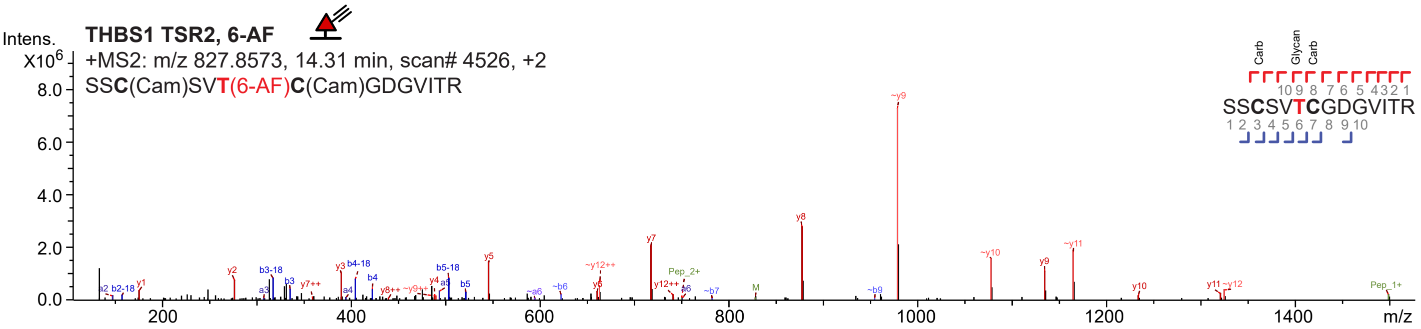

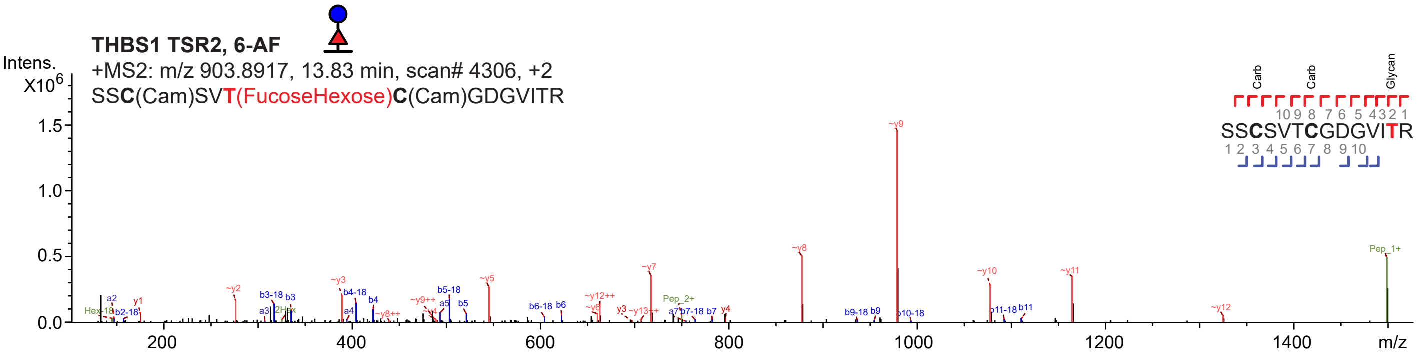
THBS1 TSR2, 6-AF
+MS2: m/z 903.8917, 13.83 min, scan# 4306, +2
SSC(Cam)SVT(FucoseHexose)C(Cam)GDGVITR
Intens.
X10^6
m/z
SSCSVTCGDGVITR
Carb
Carb
Glycan

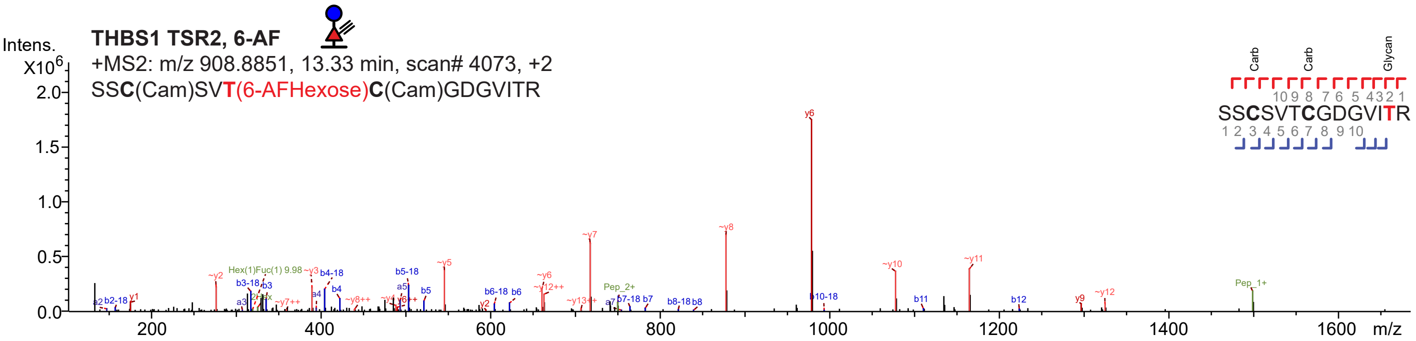
THBS1 TSR2, 6-AF
+MS2: m/z 908.8851, 13.33 min, scan# 4073, +2
SSC(Cam)SVT(6-AFHexose)C(Cam)GDGVITR
Intens.
X10^6
m/z
SSCSVTCGDGVITR
Carb
Carb
Glycan

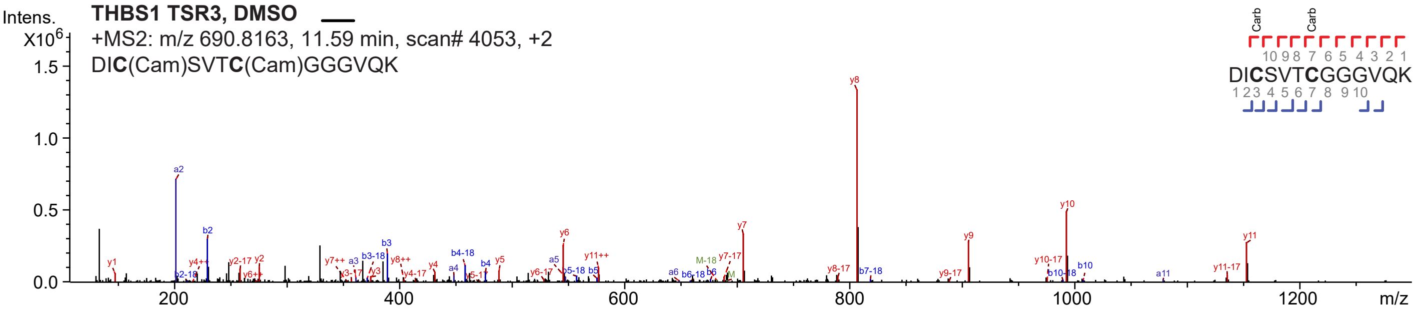
THBS1 TSR3, DMSO
+MS2: m/z 690.8163, 11.59 min, scan# 4053, +2
DIC(Cam)SVTC(Cam)GGGVQK
Intens.
X10^6
m/z
DICSVTCGGGVQK
Carb
Carb

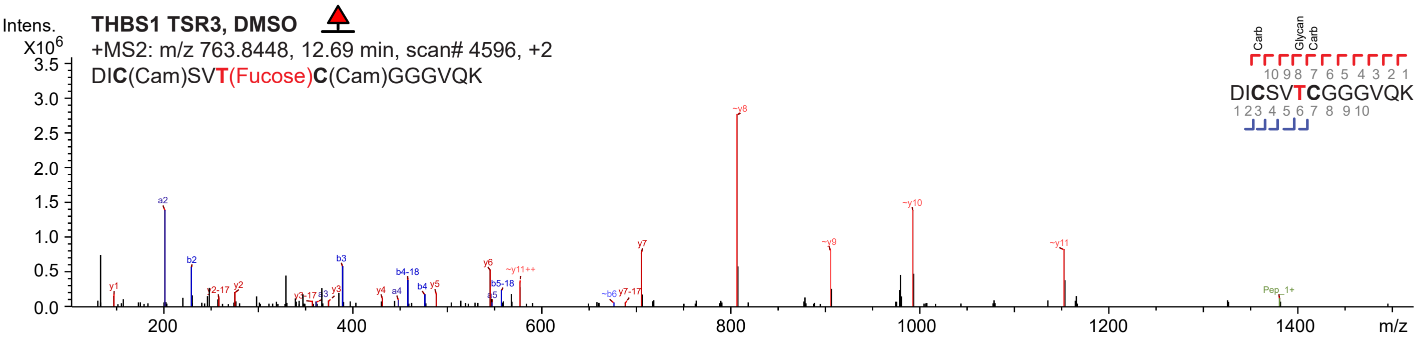
THBS1 TSR3, DMSO
+MS2: m/z 763.8448, 12.69 min, scan# 4596, +2
DIC(Cam)SVT(Fucose)C(Cam)GGGVQK
Intens.
X10^6
m/z
DICSVTCGGGVQK
Carb
Glycan
Carb

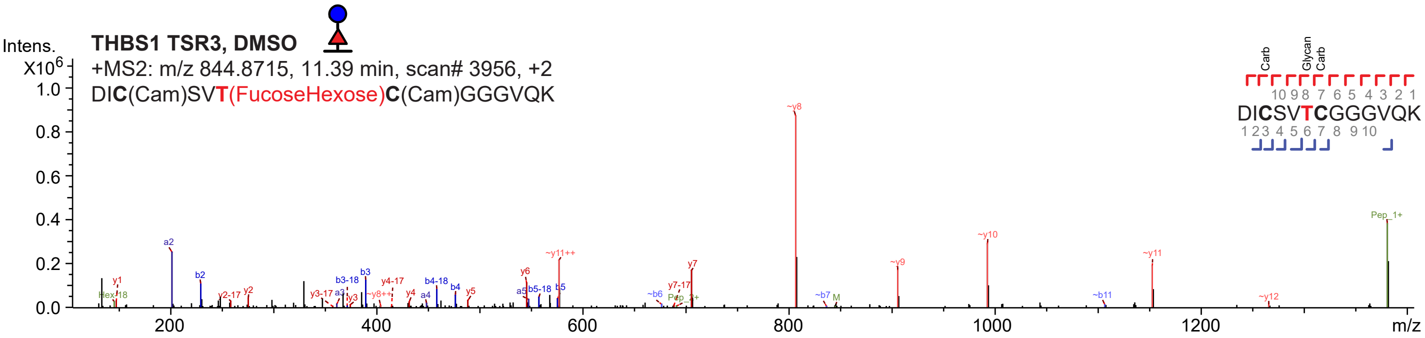
THBS1 TSR3, DMSO
+MS2: m/z 844.8715, 11.39 min, scan# 3956, +2
DIC(Cam)SVT(FucoseHexose)C(Cam)GGGVQK
Intens.
X10^6
m/z
DICSVTCGGGVQK
Carb
Glycan
Carb

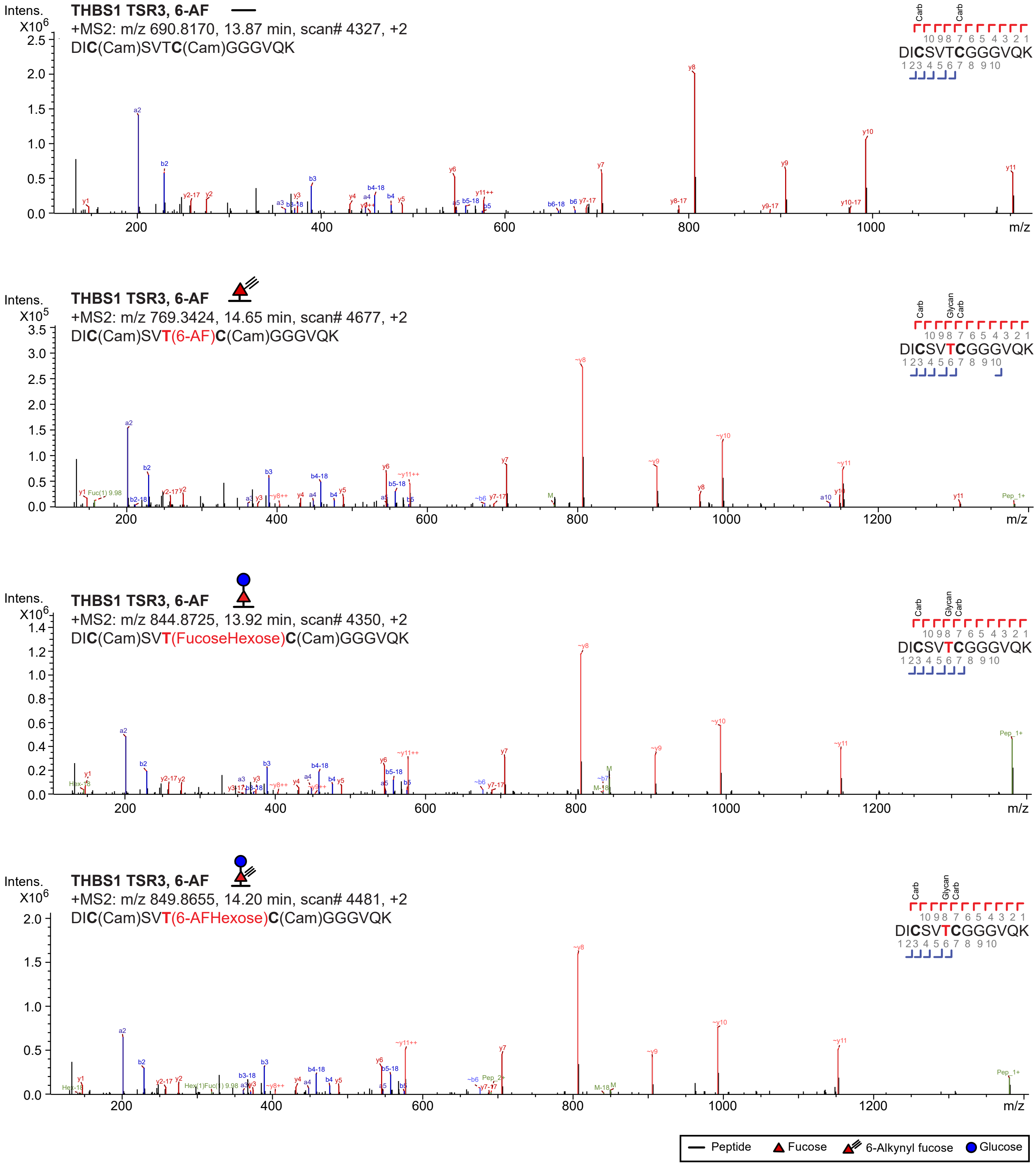
THBS1 TSR3, 6-AF
+MS2: m/z 690.8170, 13.87 min, scan# 4327, +2
DIC(Cam)SVTC(Cam)GGGVQK
Intens. X10^6
m/z
THBS1 TSR3, 6-AF
+MS2: m/z 769.3424, 14.65 min, scan# 4677, +2
DIC(Cam)SVT(6-AF)C(Cam)GGGVQK
Intens. X10^5
m/z
THBS1 TSR3, 6-AF
+MS2: m/z 844.8725, 13.92 min, scan# 4350, +2
DIC(Cam)SVT(FucoseHexose)C(Cam)GGGVQK
Intens. X10^6
m/z
THBS1 TSR3, 6-AF
+MS2: m/z 849.8655, 14.20 min, scan# 4481, +2
DIC(Cam)SVT(6-AFHexose)C(Cam)GGGVQK
Intens. X10^6
m/z
DICSVTCGGGVQK
Carb
Glycan
Peptide
Fucose
6-Alkynyl fucose
Glucose

MS2 spectra for peptides used in Supplementary Fig. S3b

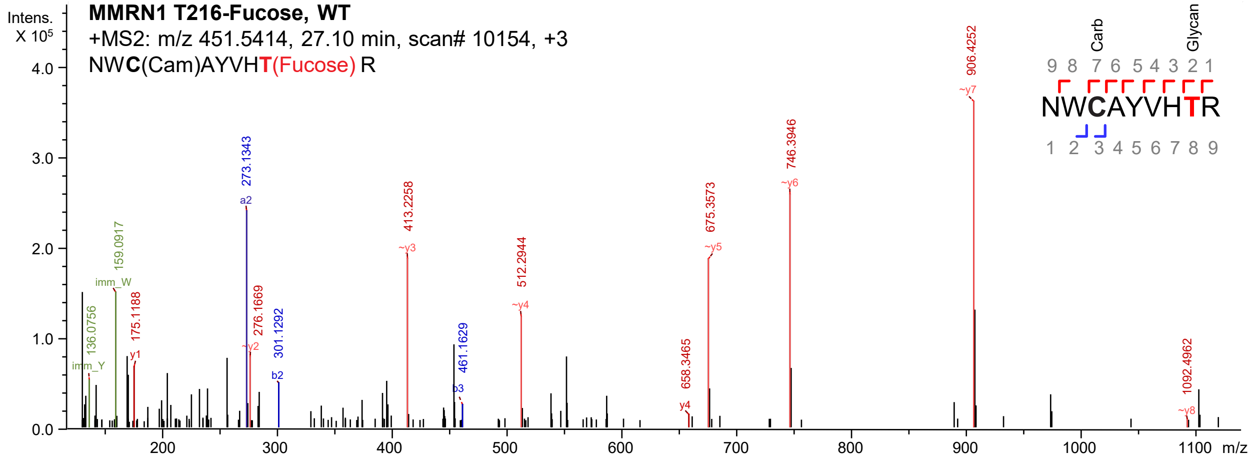

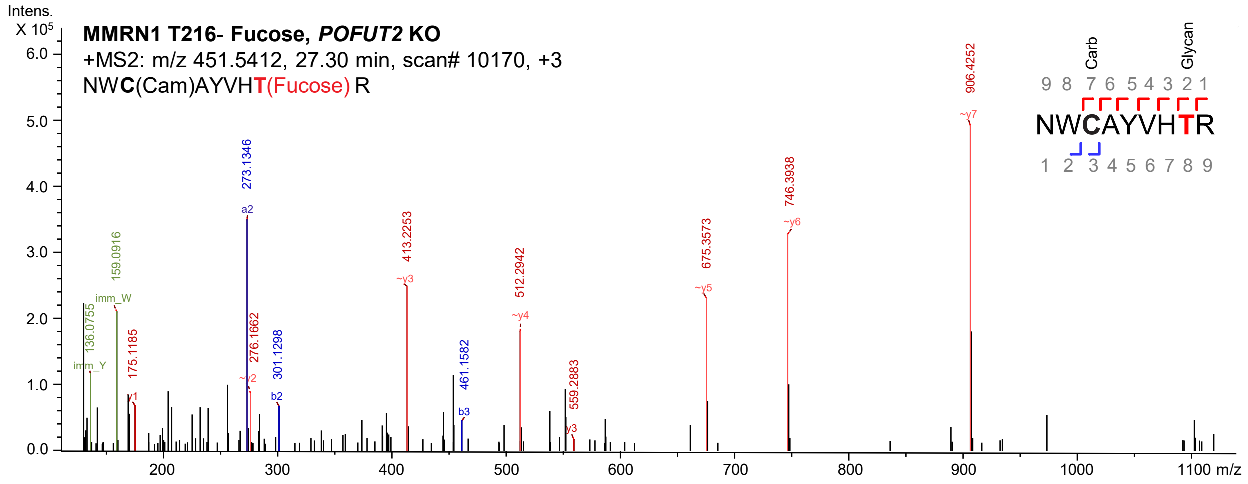

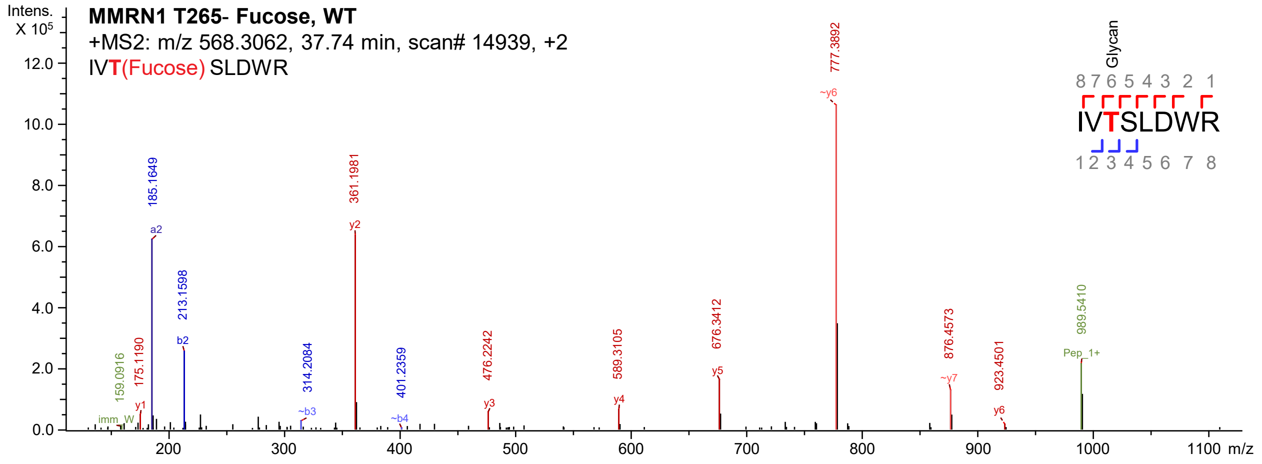

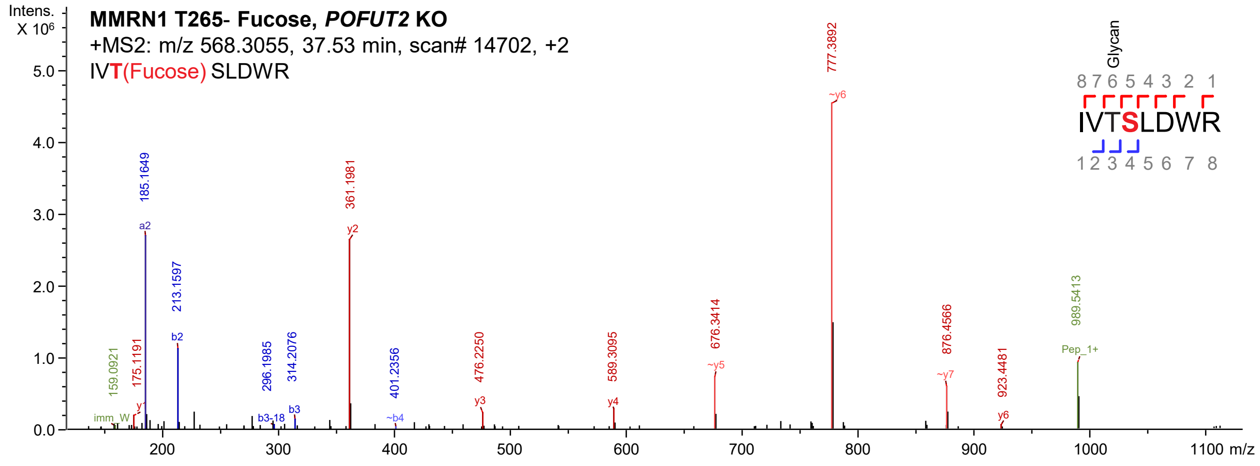

Supplement: Supplementary file 3 — Annotated HCD–MS/MS spectra for peptides used in the generation of EICs in Figs. 1d, 2 and 6b, Extended Data Fig. 7 and Supplementary Figs. 2 and 3. [file 41589_2024_1815_MOESM3_ESM.pdf]
