# Supplementary material for: FUT10 and FUT11 are protein O-fucosyltransferases that modify protein EMI domains
Source: Nat Chem Biol. 2025 Jan 7;21(4):598–610. doi: 10.1038/s41589-024-01815-x (PMC11949838; doi:10.1038/s41589-024-01815-x)
Supplement: Supplementary file 4 — AlphaFold2-multimer PAE plots for the predicted EMI domain and fucosyltransferases structures. [file 41589_2024_1815_MOESM4_ESM.pdf]

Alphafold2-Multimer alignment error (PAE) plots for the predicted EMI domain and fucosyltransferase structures.

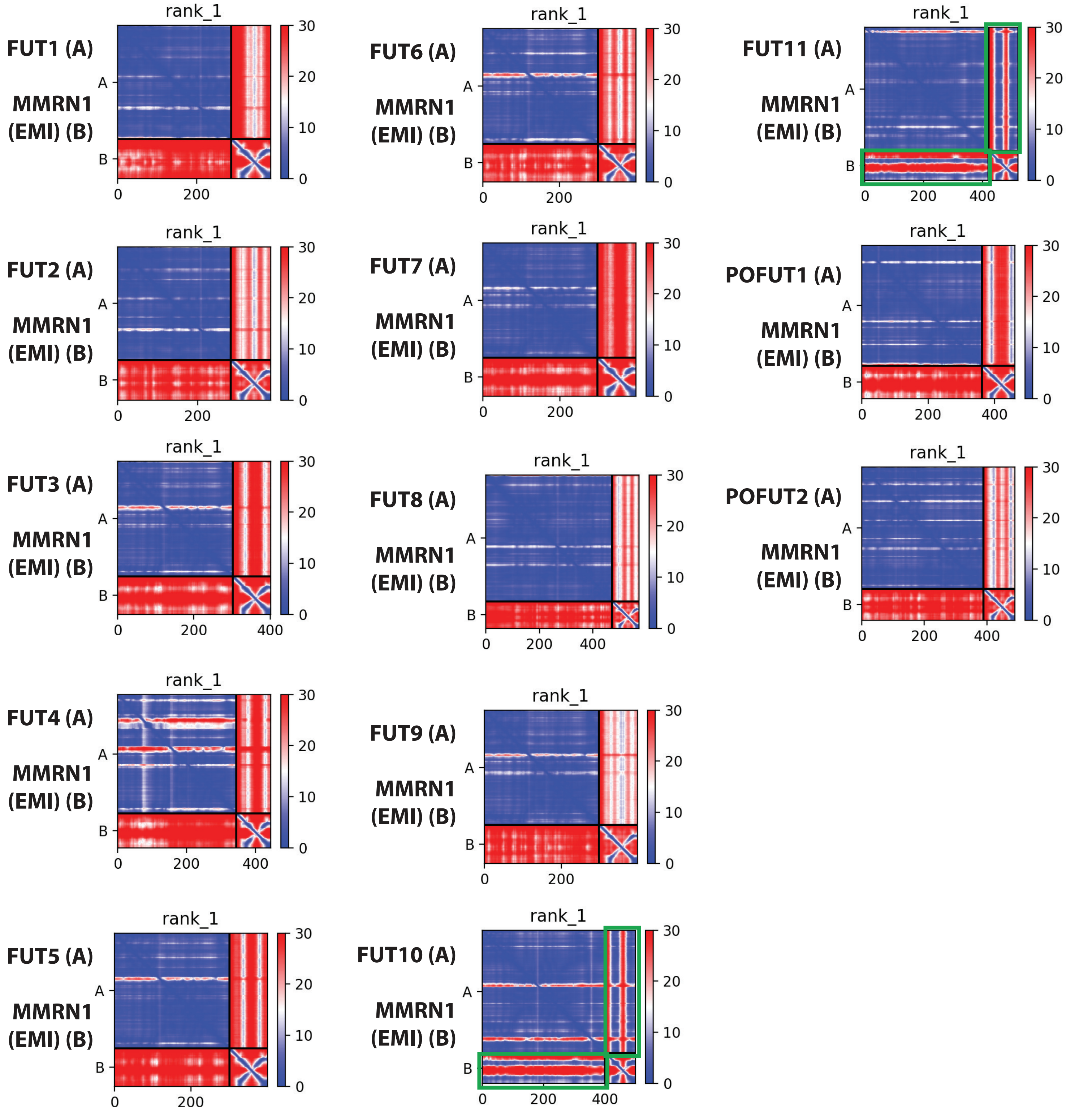

In each plot the fucosyltransferase is molecule “A” and the EMI domain is molecule “B”. The top left and bottom right regions of each plot display the PAE within either “A”, or “B”, respectively. The top right and bottom left regions of each plot display the PAE between “A” and “B”. The PAE units are Angstroms and lower values (blue) in the top right and bottom left regions of each plot indicate higher confidence models for the interaction. These high confidence interactions are highlighted for FUT10-EMI and FUT11-EMI using green bounding boxes.

FUT1 (A)
MMRN1(EMI) (B)

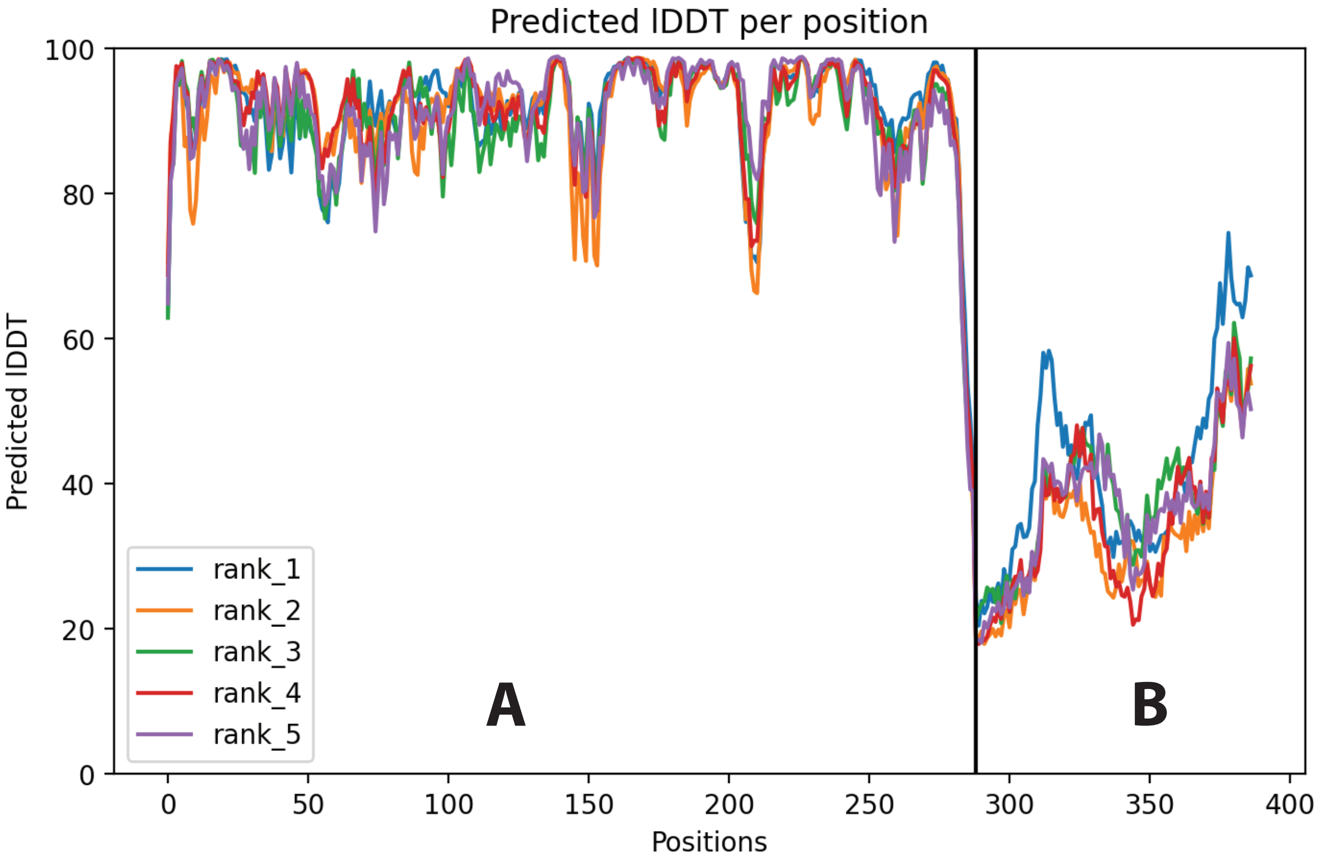

FUT2 (A)
MMRN1(EMI) (B)

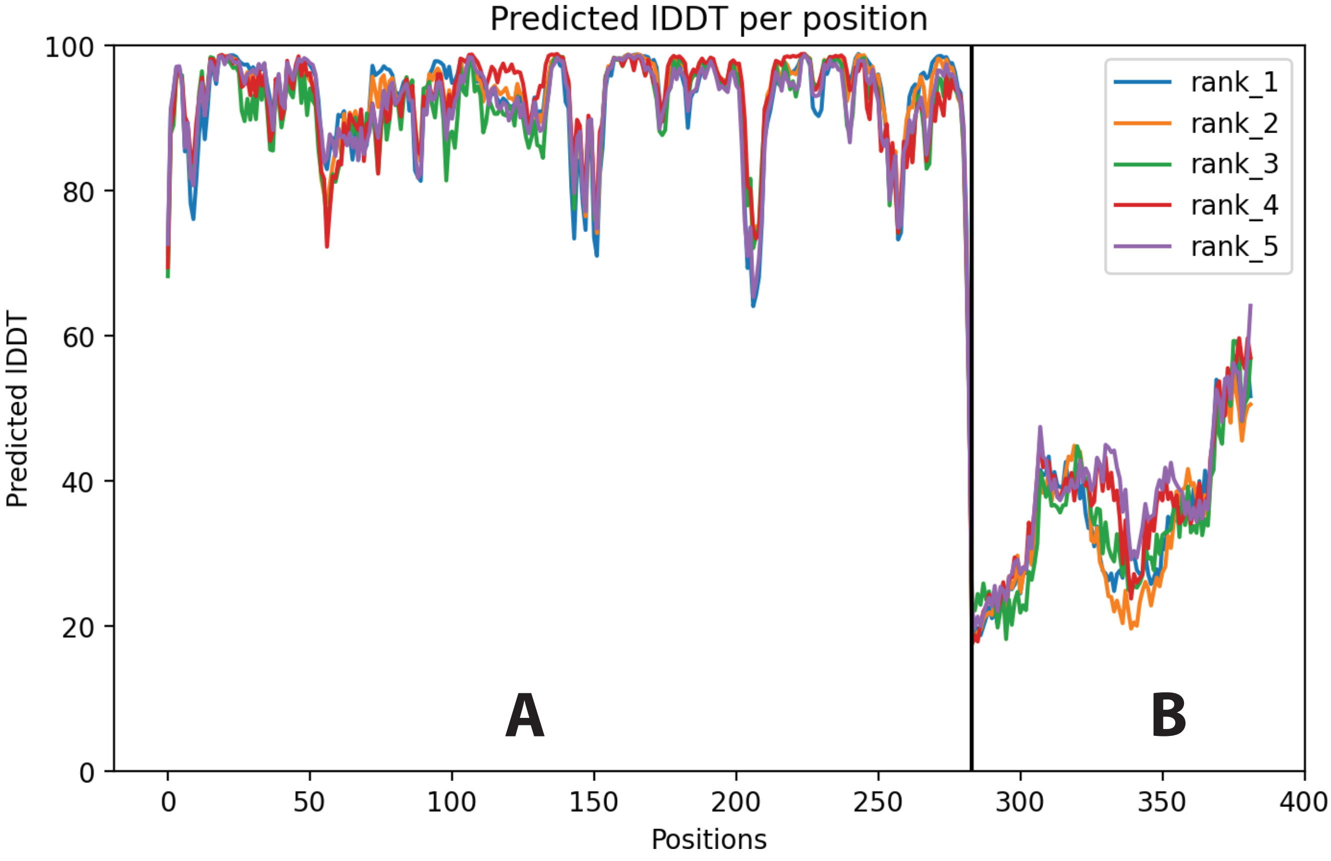

FUT3 (A)
MMRN1(EMI) (B)

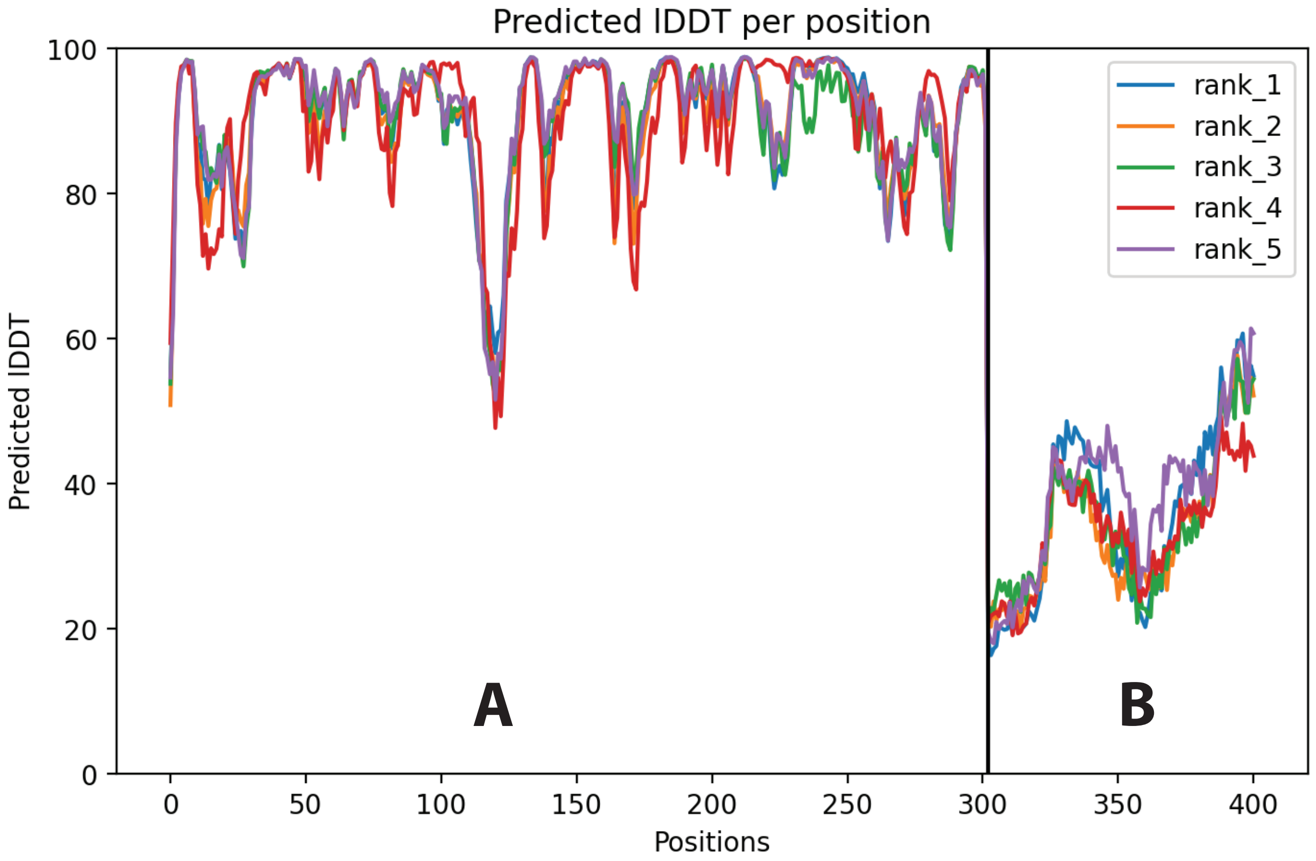

FUT4 (A)
MMRN1(EMI) (B)

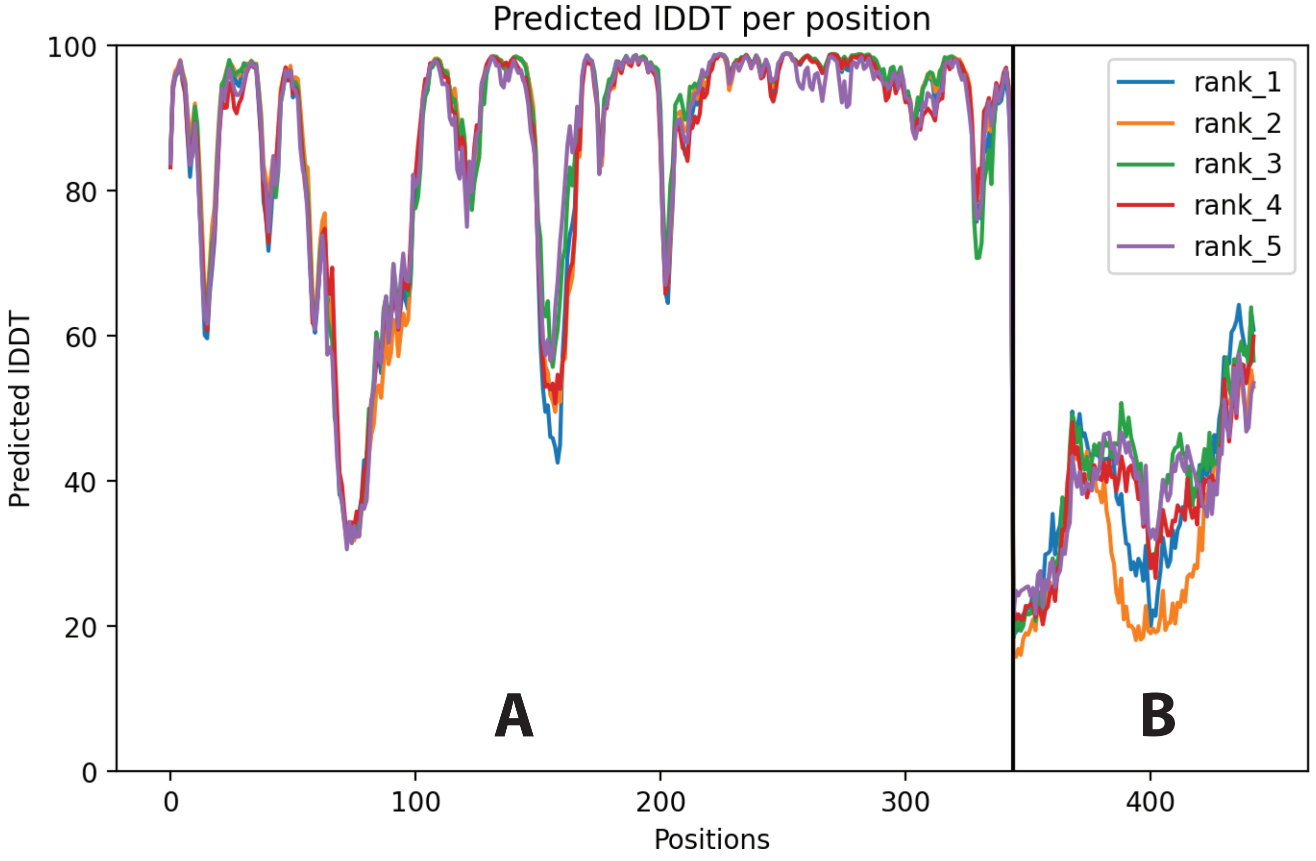

FUT5 (A)
MMRN1(EMI) (B)

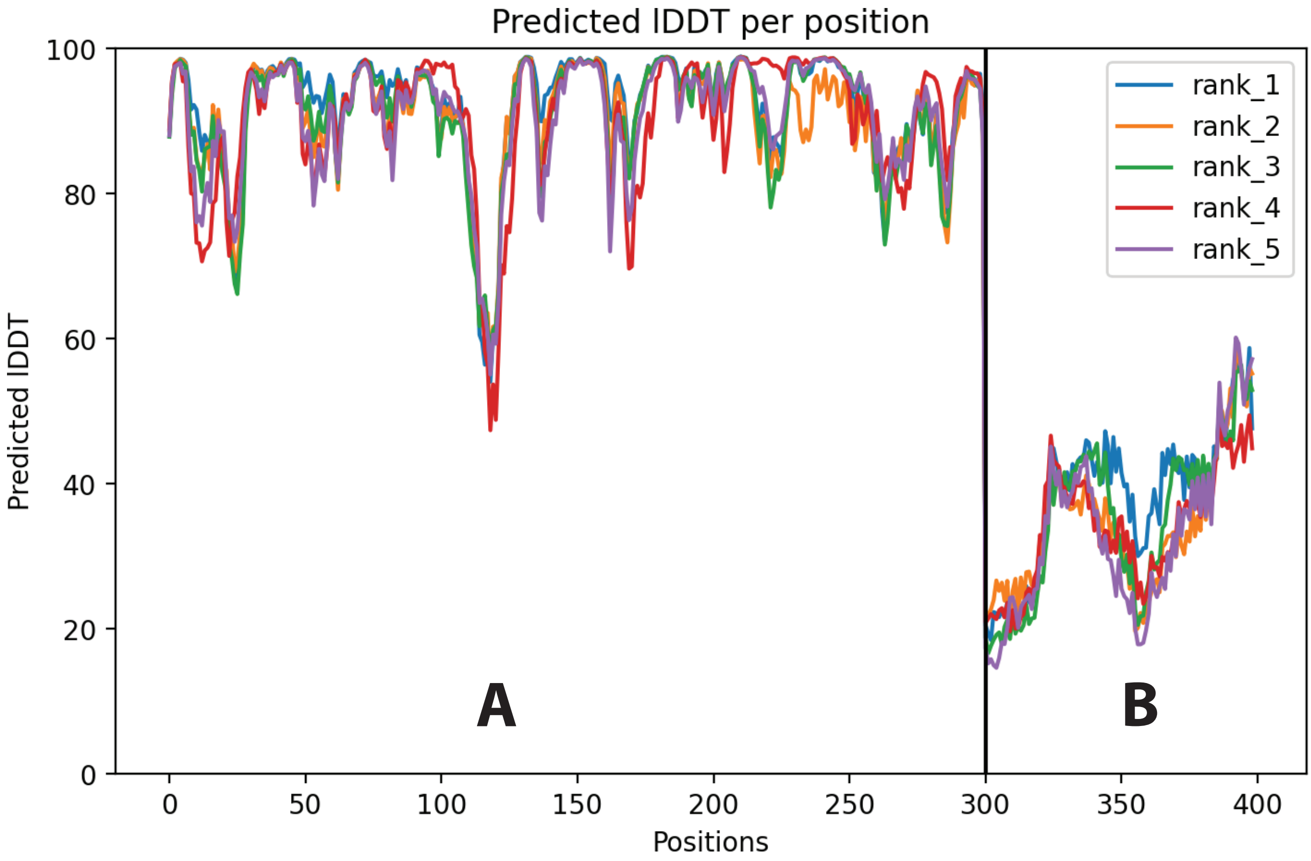

FUT6 (A)
MMRN1(EMI) (B)

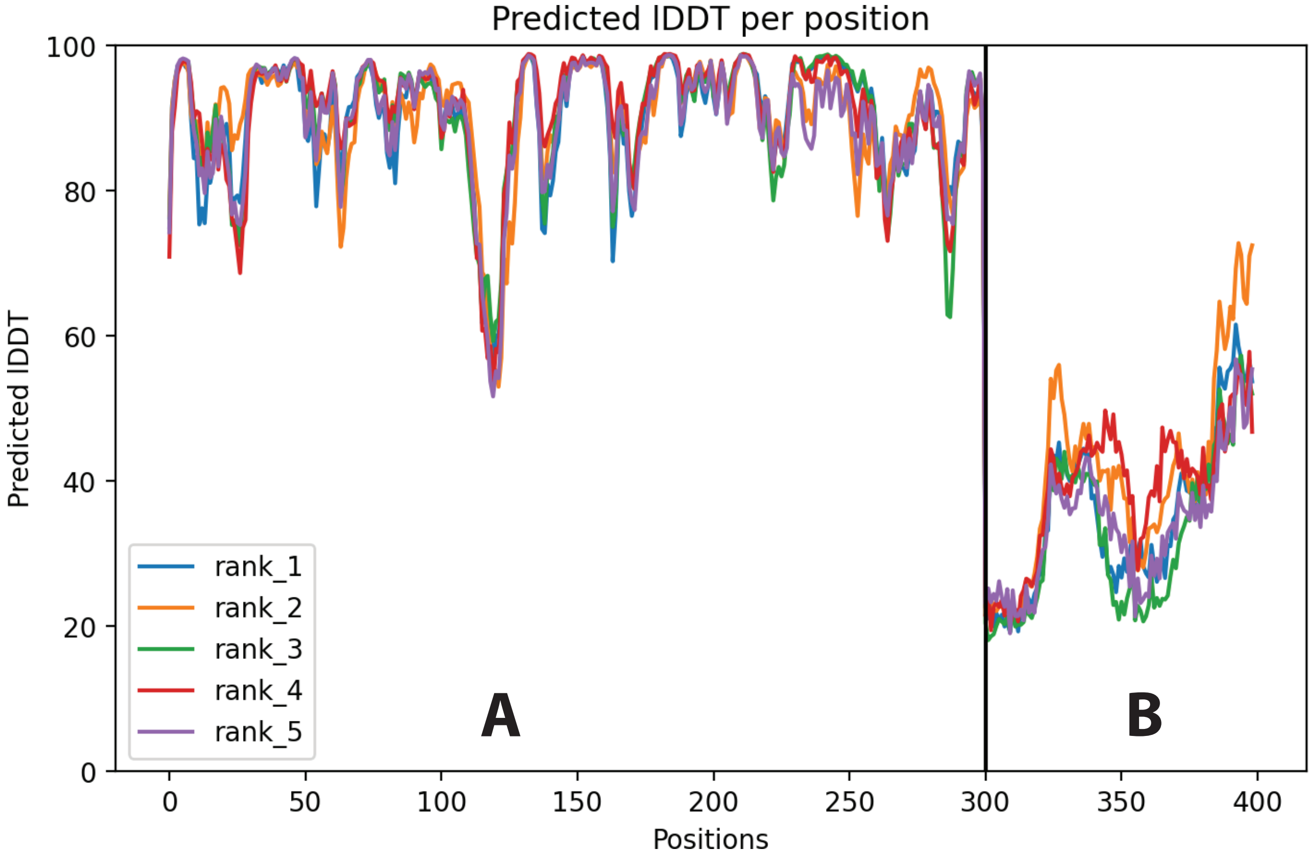

FUT7 (A)
MMRN1(EMI) (B)

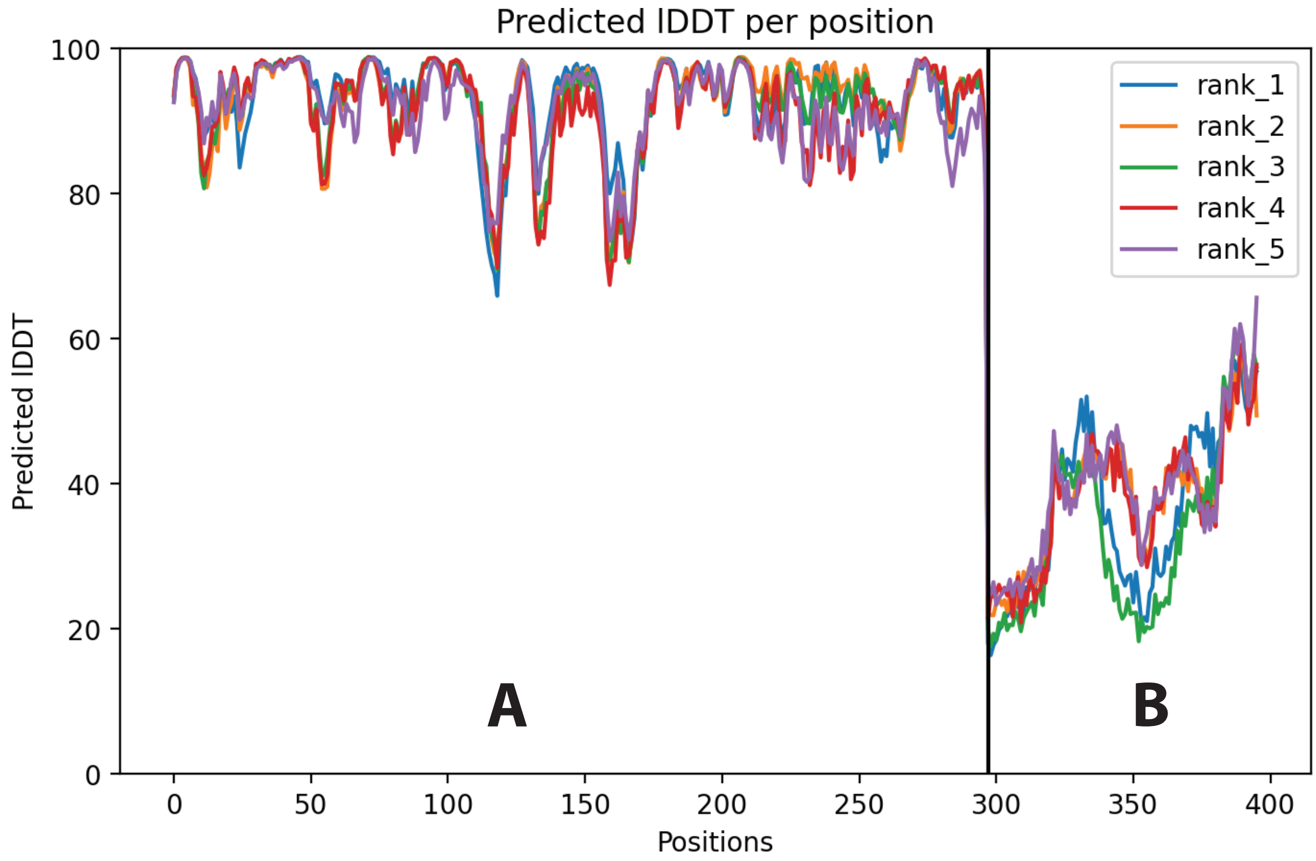

FUT8 (A)
MMRN1(EMI) (B)

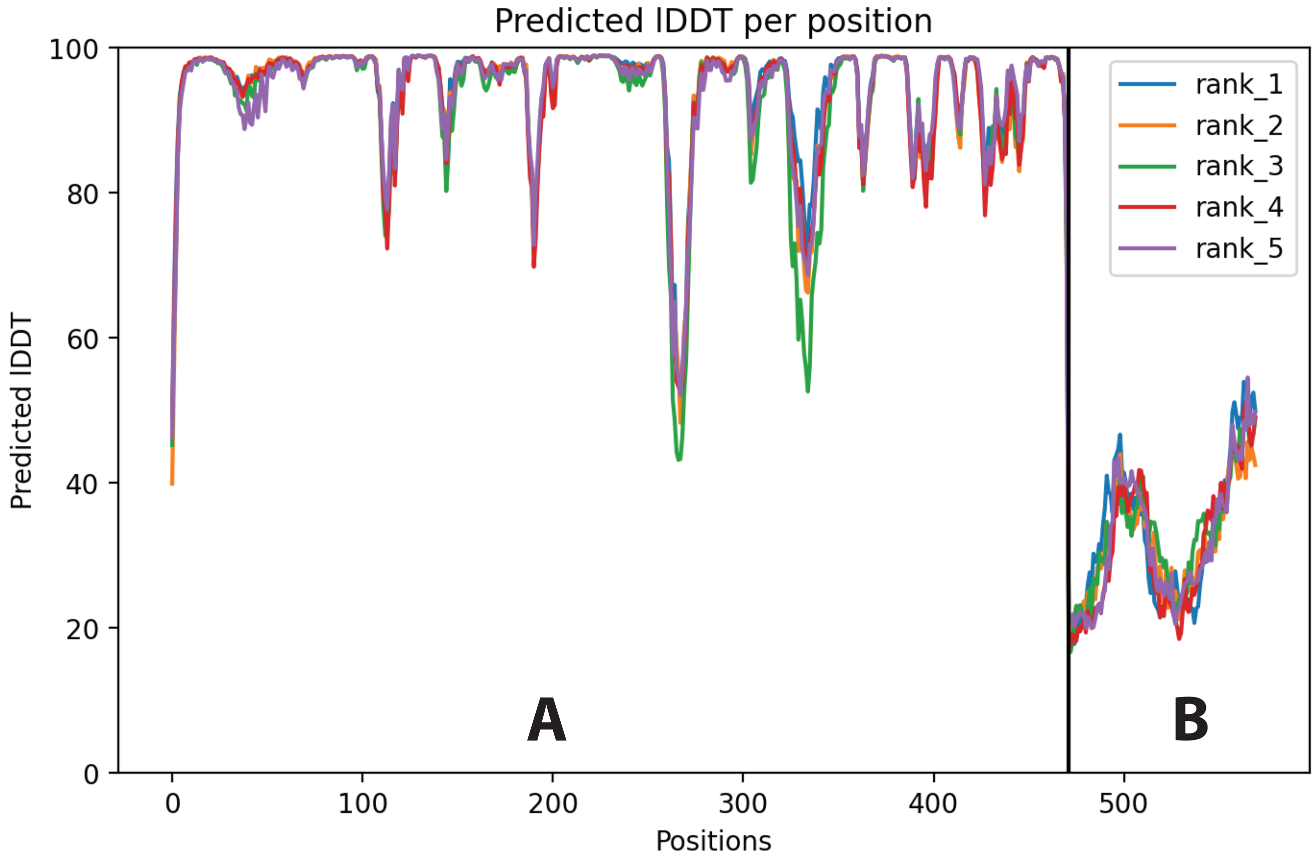

FUT9 (A)
MMRN1(EMI) (B)

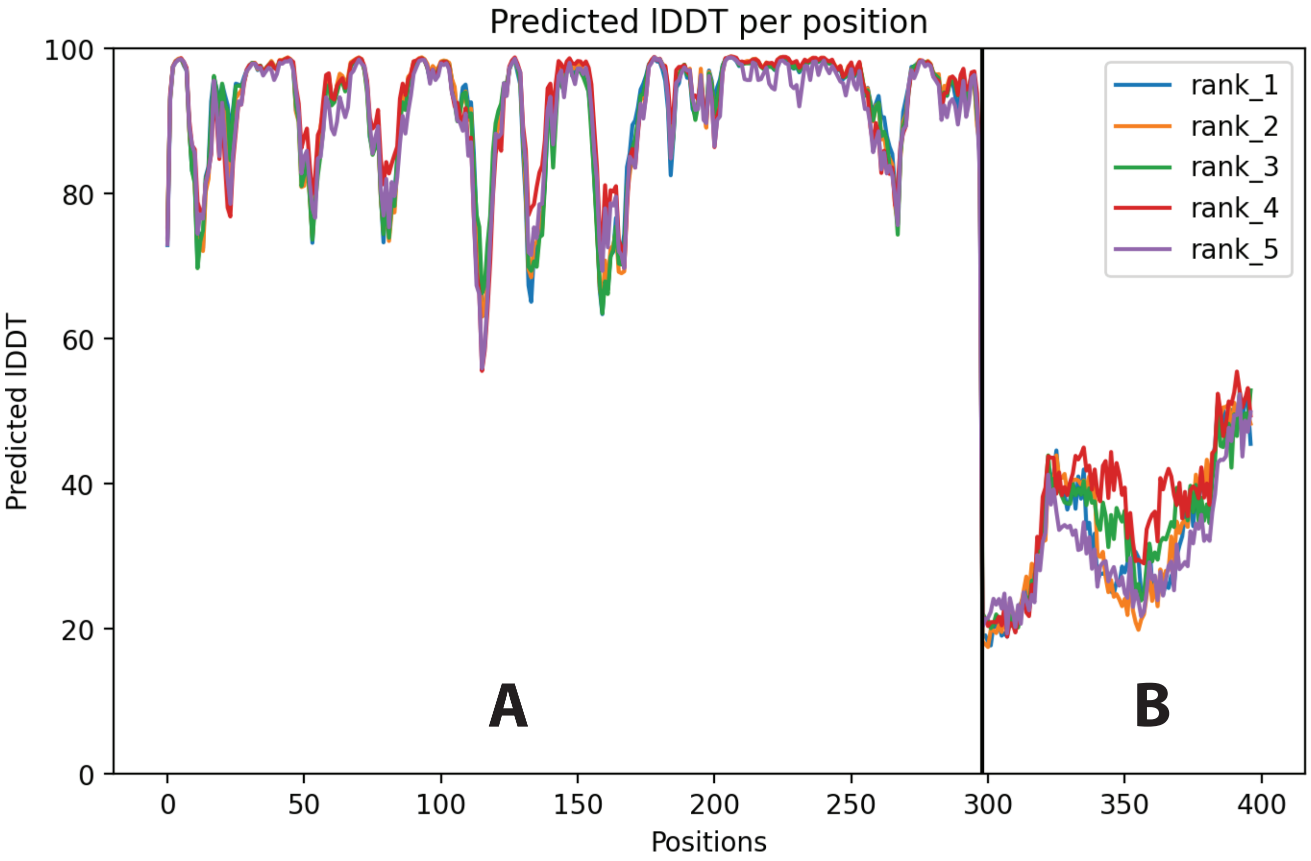

FUT10 (A)
MMRN1(EMI) (B)

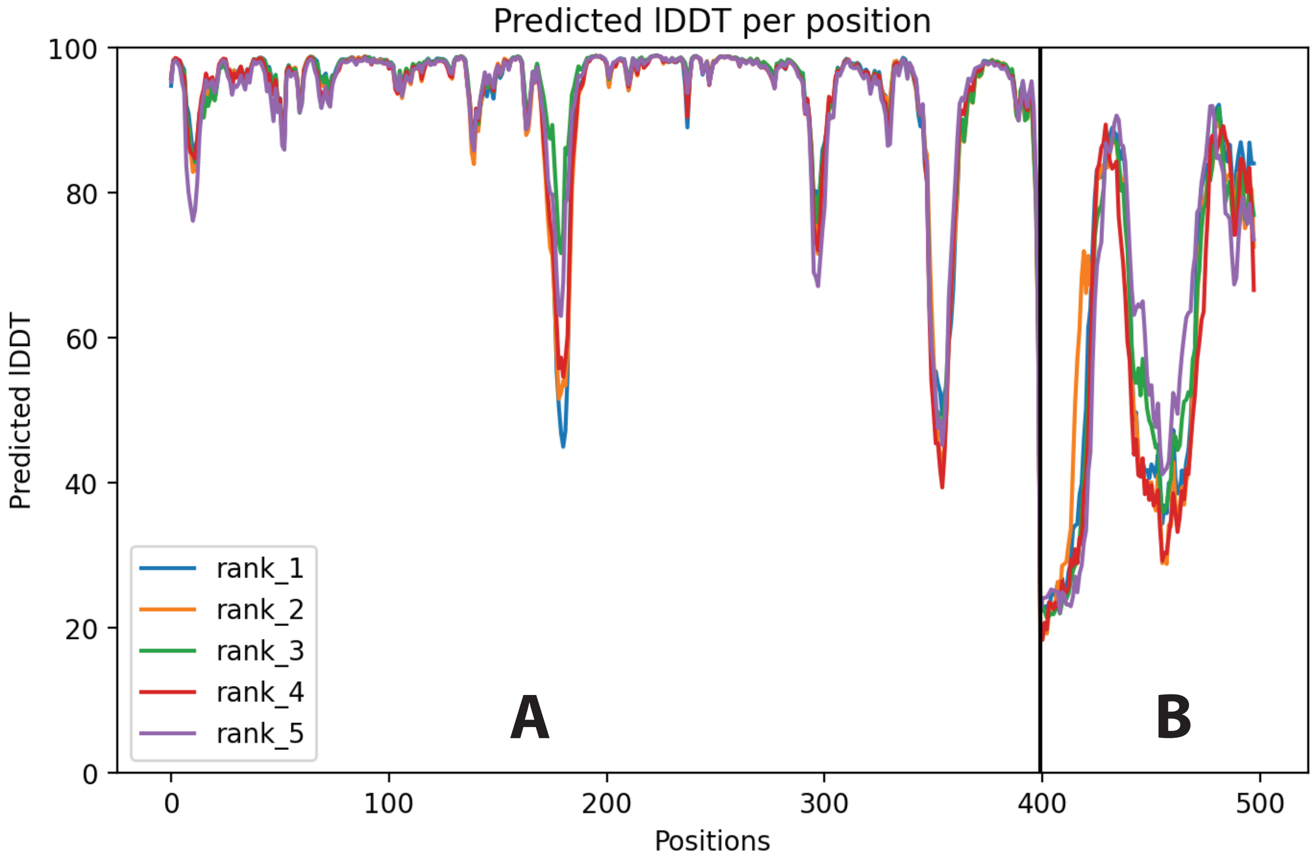

FUT11 (A)
MMRN1(EMI) (B)

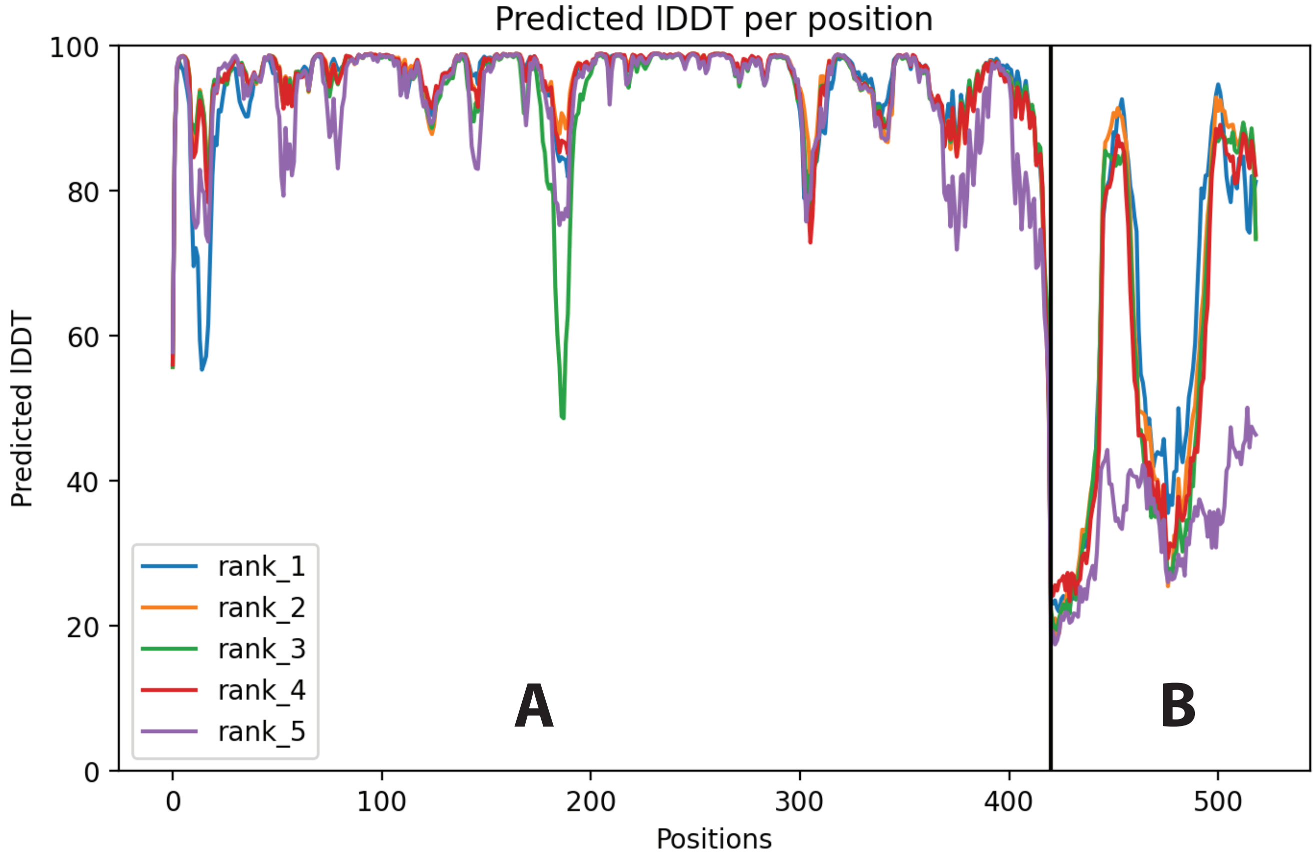

POFUT1 (A)
MMRN1(EMI) (B)

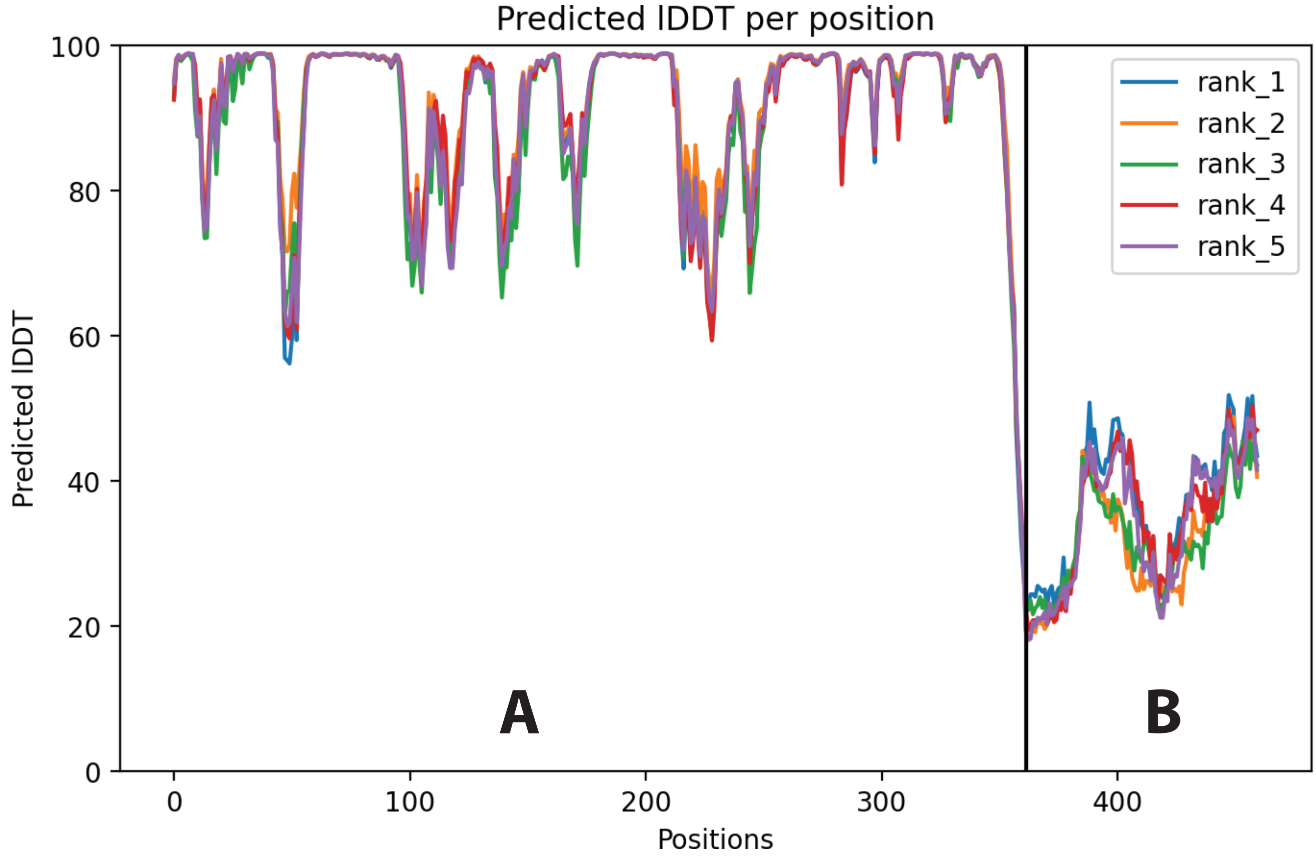

POFUT2 (A)
MMRN1(EMI) (B)

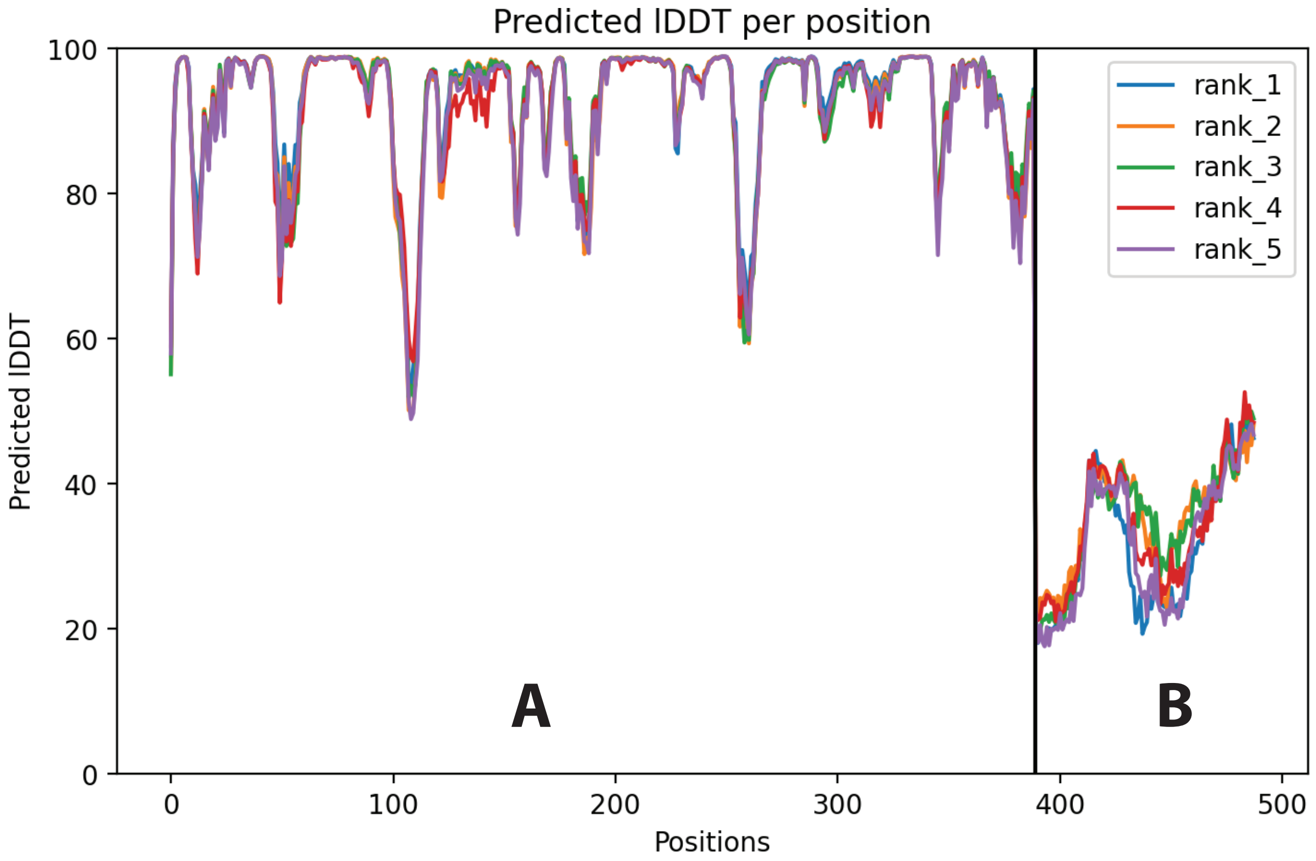

MMRN1_HUMAN : 184-282
FUT1_HUMAN: 78-365
FUT2_HUMAN: 61-343
FUT3_HUMAN: 60-361
FUT4_HUMAN: 187-530
FUT5_HUMAN: 75-374
FUT6_HUMAN: 60-359
FUT7_HUMAN: 46-342
FUT8_HUMAN: 105-575
FUT9_HUMAN: 62-359
FUT10_HUMAN: 81-479
FUT11_HUMAN: 73-492
OFUT1_HUMAN: 28-388
OFUT2_HUMAN: 41-429
