# Supplementary material for: FUT10 and FUT11 are protein O-fucosyltransferases that modify protein EMI domains
Source: Nat Chem Biol. 2025 Jan 7;21(4):598–610. doi: 10.1038/s41589-024-01815-x (PMC11949838; doi:10.1038/s41589-024-01815-x)
Supplement: Supplementary file 10 — Unprocessed western blots. [file 41589_2024_1815_MOESM10_ESM.pdf]

Image Display Values

Channel	Color	Minimum	Maximum	K
700	Red	28.6	258	1
800	Green	0.919	2.03	1

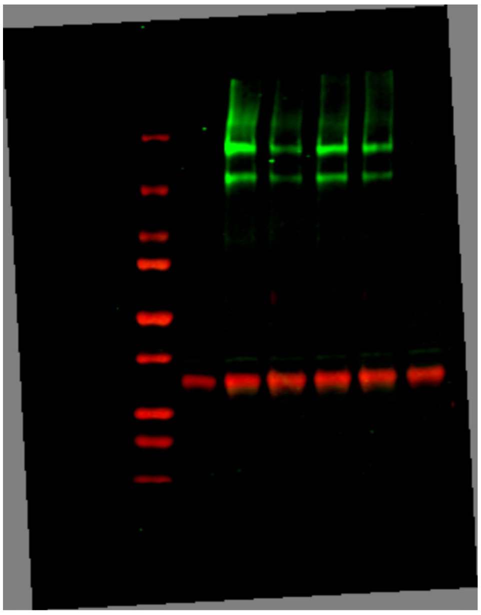

Fig. 6c

Image Display Values

Channel	Color	Minimum	Maximum	K
700	Red	3.74	80.5	1
800	Green	3.23	17.1	1

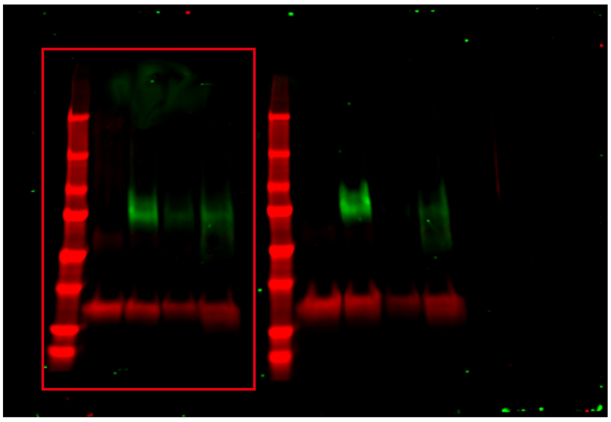

Fig. 6d

Image Display Values

Channel	Color	Minimum	Maximum	K
700	Red	27.0	194	1
800	Green	3.08	149	1

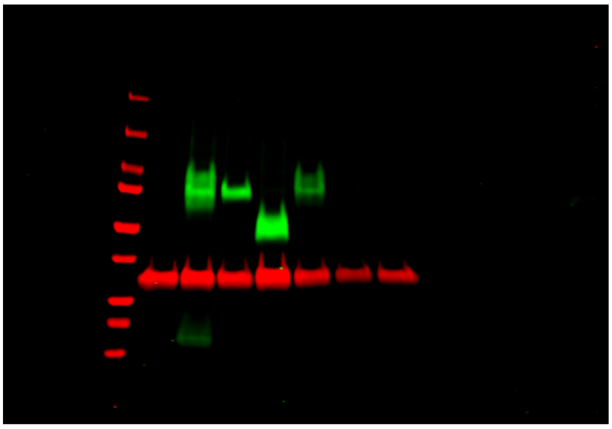

Fig. 6e
